# Applying Corrigan’s progressive model of self-stigma to people with depression

**DOI:** 10.1371/journal.pone.0224418

**Published:** 2019-10-29

**Authors:** Nele Cornelia Göpfert, Steffen Conrad von Heydendorff, Harald Dreßing, Josef Bailer

**Affiliations:** 1 Department of Clinical Psychology, Central Institute of Mental Health, Medical Faculty Mannheim / University Heidelberg, Mannheim, Germany; 2 Department of Psychiatry and Psychotherapy, Central Institute of Mental Health, Medical Faculty Mannheim / University Heidelberg, Mannheim, Germany; 3 Department of Forensic Psychiatry, Central Institute of Mental Health, Medical Faculty Mannheim / University Heidelberg, Mannheim, Germany; University of Vienna, AUSTRIA

## Abstract

**Background:**

The progressive model of self-stigma describes four stages of internalizing stereotypes of mental illness: stereotype awareness, personal agreement, self-concurrence, and harm to self (i.e., self-esteem). Successive stages are postulated to be the most highly related. Endorsement is presumed to decrease by stage. The model has been supported in most but not all elements in various studies. The procedural character has not yet been investigated in one integrative model. The aim of this study was to test the progressive model of self-stigma in three respects: I) successive stages have the strongest associations, II) endorsements decrease with each stage, and III) the procedural character can be represented by one serial mediation model.

**Methods:**

A cross-sectional computer-based survey was conducted in two samples of patients with depression; one online sample (N_A_ = 550; only self-report) and one clinical face-to-face sample (N_B_ = 180; screening by treatment staff). The inclusion criteria were, age of 18–70 years, sufficient cognitive abilities and German language skills. IBM SPSS statistics 24 was used for Cronbach’s alphas, descriptive statistics, Spearman correlations, and Mann-Whitney-U tests. The PROCESS procedure for SPSS Version 3.00 was used for mediation analyses.

**Results:**

The results support the progressive model of self-stigma in people with depression in most respects: Endorsements for stereotype awareness were higher than for personal agreement and self-concurrence, and no relevant difference was found between personal agreement and self-concurrence. Successive stages had the strongest associations, with the exception of the association between stereotype awareness and self-esteem, which was higher than the association between stereotype awareness and personal agreement and self-concurrence. The association between stereotype awareness and self-esteem was mediated via personal agreement and self-concurrence.

**Conclusion:**

The progressive model of self-stigma offers a theoretical foundation for the process research of self-stigma. Longitudinal research may investigate predictive effects and whether different stages of self-stigma require specific consideration in their prediction, consequences, and potential interventions.

## Introduction

Self-stigma appears to be a crucial risk factor in people with mental illness. A higher level of self-stigma in persons with mental illness is negatively related to self-esteem, self-efficacy, quality of life, general functioning, self-clarity, hope, recovery, and professional help-seeking [1–5], and positively related to psychiatric symptoms and suicide ideation [6–8]. Understanding the nature of self-stigma is essential for preventing and overcoming potential consequences.

Goffman was the first to define “stigma as a mark (attribute) that links a person to undesirable characteristics (stereotypes)” [9]. Recent literature adds the perspective of the individual carrying the stigmatized attribute [10, 11]. Therefore, researchers often distinguish between public and self-stigma, both comprising elements of stereotypes, prejudice, and discrimination [12]. Public stigma refers to the general population agreeing with stereotypes and reacting with discrimination against people with mental illness. The literature differentiates two types of public stigma: (a) perceived public stigma (also called stereotype awareness) refers to one’s perception of the attitudes and behaviours of others towards depression, and (b) personal stigma (also called internalised stigma or personal agreement) refers to one’s own attitudes towards depression. Accepting society’s stereotypes and applying them against oneself in relation to having depressive symptoms is described as self-stigma (or self-concurrence). Different conceptualizations of self-stigma have been used in a variety of studies [13, 14]. Most of them consider stigma to be a static rather than a process phenomenon.

### The progressive model of self-stigma

**Fig 1:**
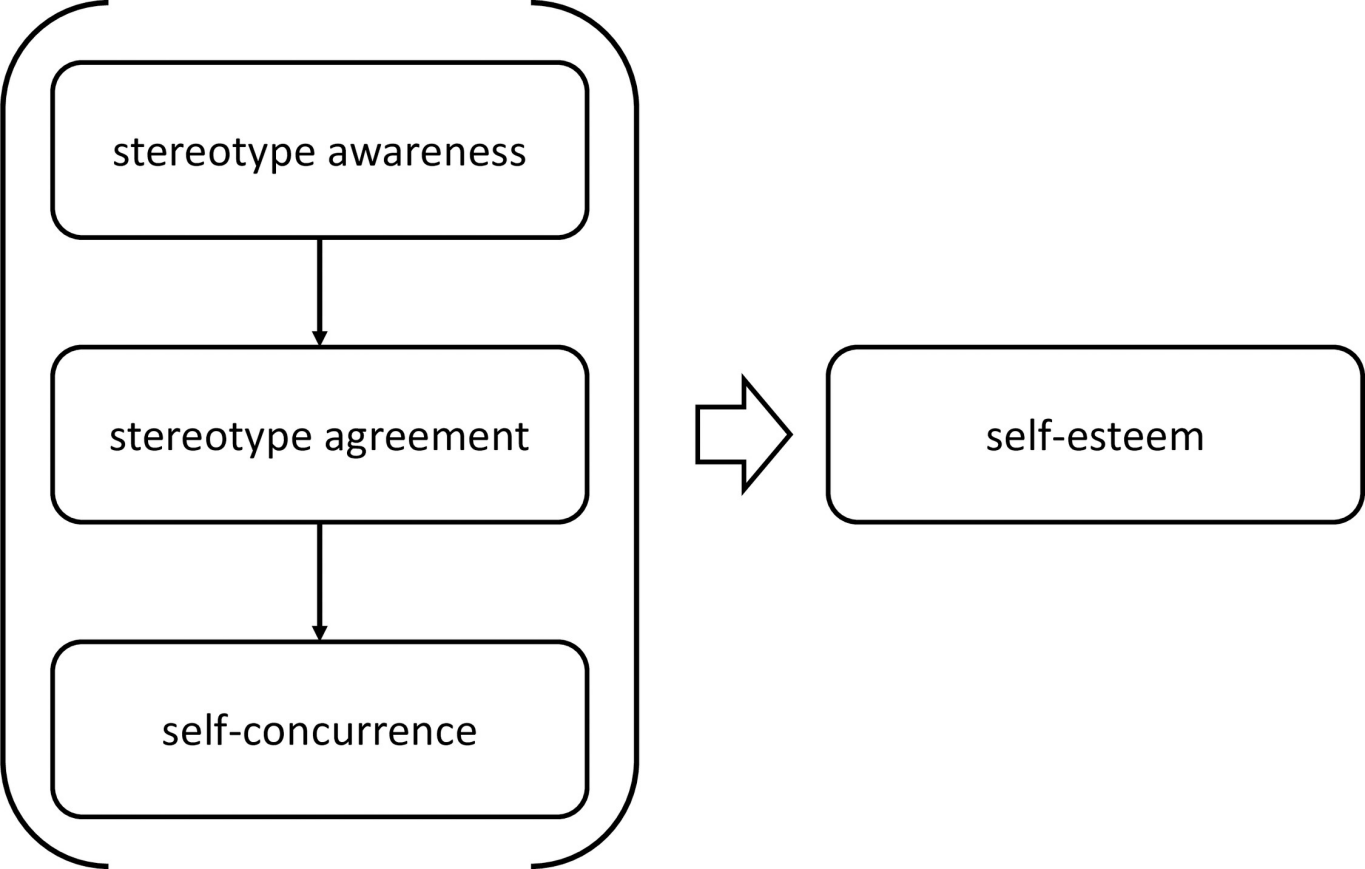
Process-oriented model of self-stigma with self-esteem as an outcome factor adapted from Watson et al. [19].

Corrigan and colleagues integrated several aspects of stigma into a progressive model of self-stigma [15]. As presented in Fig 1, the authors differentiate between three succeeding stages, namely, the awareness of stereotypes (perception of public stigma), followed by personal agreement (believing public stigma to be true), and self-concurrence (internalizing stereotypes and applying them to oneself). Harm to self (such as lower levels of self-esteem) is considered a fourth stage or consequence of self-concurrence. Two main assumptions are postulated by the model: (i) a “trickle down” in nature, i.e., the highest endorsement for stereotype awareness, followed by lower endorsements for each stage, and (ii) proximal stages (e.g. stereotype awareness and personal agreement) are expected to be more strongly associated than more distant stages (e.g., stereotype awareness and self-concurrence). To test this model, a sample of 85 persons with mixed diagnoses (schizophrenia, schizoaffective disorder, bipolar disorder and unipolar major depressive disorder) completed self-report measures. The results indicated a trickle-down in nature for stereotype awareness, personal agreement, and self-concurrence. However, the sums between self-concurrence and harm to self did not differ significantly. Proximal stages were more highly correlated than more distant stages, with the exception of stereotype awareness and harm to self, which were more highly correlated than stereotype awareness with both personal agreement and self-concurrence. As such, the progressive model of self-stigma has been supported in most but not all postulated elements. Replication studies could help to shed light on previous inconsistent research findings.

One implicit but not explicitly investigated aspect of the progressive model of self-stigma is its procedural character of the stages. The conceptual model, as depicted in Fig 1, indicates that self-esteem is a consequence of each of the first three stages. While direct associations have been investigated for both distant and proximal stages of the model with self-esteem, it is also likely that there are indirect associations that explain the effect of distant stages. While higher levels of stereotype awareness are associated with higher levels of stereotype agreement, higher levels of stereotype agreement in turn are associated with higher levels of self-concurrence, which in turn are associated with lower levels of self-esteem. Kao et al. [16] report evidence of the mediating role of self-concurrence between stereotype awareness and psychosocial outcomes (such as self-esteem, depressive symptoms, and subjective quality of life). In addition to correlative associations and score comparisons, specific statistical analyses, such as serial mediation models, can capture this procedural character.

Furthermore, one difficulty in the model of Corrigan et al. was disentangling the effects of self-concurrence on harm to self (i.e. self-esteem decrement) from those of depressive symptoms. Regression analyses revealed no additional variance in harm to self that was explained by awareness, agreement, or self-concurrence after the current level of depression was partialled out [15]. Self-concurrence and harm to self seem to be highly correlated and not significantly different in their scores [15]. One reason may be that depression symptoms themselves are by definition associated with lower levels of self-esteem. Livingston and Boyd [13] discuss conceptual and measurement issues regarding the relationship between stigma measures and psychosocial variables in their review. They note the need to critically investigate specific relations. It is still unclear whether stigma measures are empirically differentiable from symptoms of depression in explaining levels of self-esteem, especially in samples of people with depression.

### Aims and hypotheses

Therefore, the aim of this study is to examine the progressive model of self-stigma by Corrigan et al. [15], which represents the four stages of stereotype awareness, personal agreement, self-concurrence, and self-esteem specifically for people with depression using serial mediation analyses and controlling for current levels of depression in two independent clinical samples. Age in years and gender had been inconsistently correlated with self-concurrence in previous research and are therefore assessed as additional control variables [13].

In line with the model of Corrigan and his colleagues on self-stigma in people with mental illness and the outlined empirical background the following hypotheses are stated for people suffering with depression:

- Stigma attitudes are endorsed decreasingly with the highest endorsement for stereotype awareness, followed by lower endorsements for each stage.
- Proximal stages are more strongly associated than more distant stages.
- Direct associations of distant stages are mediated by proximal stages: While higher levels of stereotype awareness are associated with higher levels of stereotype agreement, higher levels of stereotype agreement in turn are associated with higher levels of self-concurrence, which in turn are associated with lower levels of self-esteem.

## Materials and methods

### Participants and procedure

The present study was designed as a cross-sectional computer-based survey. Data from two independent samples were collected.

Sample A accessed the survey online via www.soscisurvey.de from 8 March 2017 to 9 July 2018. The link was distributed via online platforms (website of Psychologieforum, website of Deutsche Depressionsliga, website of a psychiatric clinic, among others), as well as via representatives of the University of Mannheim and autonomous outpatient groups. Inclusion criteria were at least one pre-diagnosed depressive episode or dysthymia and age of 18–70 years. The following exclusion criteria were not captured in Sample A: insufficient cognitive abilities and German language skills, acute psychotic, manic or hypomanic episode, addiction symptoms, or acute suicidal tendencies. The questionnaire stopped automatically if no lifetime depressive episode was reported. At the beginning of the survey, participants received written informed consent about aims, voluntary participation, and procedure. Confirmation was required to continue. The questionnaire was viewed 849 times, 662 persons completed the questionnaire, and 561 reported lifetime depression. Five data sets were excluded from analyses because of abnormalities in response patterns due to comparably rapid responding behaviour (case 112) or no variance on at least four subscales (cases 4, 43, 211, 470). Six additional data sets were excluded because of gender uncertainty (cases 69, 109, 289, 334, 385, 474). All other data sets did not have any missing data. After the data was cleaned, n = 550 data sets fulfilled the eligibility criteria (age range from 18 to 73 years).

Since online surveys have several limitations, a second face-to-face sample was used to control for online study-specific biases due to the following: the ability of online surveys to reach only participants with internet access, the self-report of limited screening criteria, or the absence of research team members when questions arise. Sample B was therefore taken from the research project “Effects of Media Reports on Self-Stigmatization, Self-esteem and Affectivity in Persons with Depression”, which was conducted from 1 March 2017 to 18 August 2018 [17]. One advantage of the face-to-face sample was a more accurate screening, which was based on not only self-report but also clinical data: potential participants were recruited by the treating psychiatrists and psychotherapists of inpatient and outpatient departments of a psychiatric clinic. The following eligibility criteria were demonstrated by the treating staff based on a screening questionnaire: at least one pre-diagnosed depressive episode or dysthymia, age of 18–70 years, sufficient cognitive abilities and German language skills. The exclusion criteria were acute psychotic, manic or hypomanic episode, addiction symptoms, or acute suicidal tendencies. Both, the clinical staff and research team ensured that patients had the capacity to freely give informed consent. Informed consent was first given orally and second given in writing. All participants completed the computer-based battery of questionnaires. Out of the 202 persons who fulfilled the inclusion criteria, 186 Persons finally decided to participate in the study. Because of technical (cases 117 and 154: interruption of internet; case 178: interruption of server connection) and logistical reasons (case 142: double participation; case 141: interruption because of a medical appointment; case 177: allocated to wrong group), 6 incomplete data sets had to be excluded from analyses. All other data sets did not have any missing data. Participants received a 20€ expense allowance (for study details see [17]).

Ethical approval was provided by the Ethical Committee of the Medical Faculty of Mannheim, Heidelberg University, Germany for both study samples (study: 2016-655N-MA).

### Instruments

#### Self-stigma

The German version of the Self-Stigma of Mental Illness Scale (SSMIS [1]) was used to assess the three phases of self-stigma, namely, stereotype awareness, personal agreement, and self-concurrence [18]. Based on the experience of Watson et al. [19], the fourth subscale of the SSMIS, namely, self-esteem decrement, was not included in this study because of its difficult wording.

Each subscale consisted of 10 items. All items of the stereotype awareness subscale were structured in the same way: “I think *the public* believes most persons with mental illness are… (e.g., dangerous).” Cronbach’s alpha was α = 0.91 in Sample A and α = 0.91 in Sample B. Items of the personal agreement subscale were introduced by “*I* think most persons with mental illness are… (e.g., dangerous).” Internal consistency was α = 0.85 in Sample A and α = 0.87 in Sample B. Self-concurrence implies a causal relation between one’s mental illness and stereotype characteristics: “Because I have a mental illness, I am… (e.g., dangerous).” Cronbach’s alpha was α = 0.84 in Sample A and α = 0.81 in Sample B. Response options ranged from 1 = “I strongly disagree” to 9 = “I strongly agree”. Summed scores were computed for each subscale, with higher scores indicating a higher level of stigma attitudes.

#### Self-esteem

The fourth subscale of the SSMIS was exchanged via a revised version of the German adaptation of the Rosenberg’s well-known self-esteem scale consisting of 10 items [20, 21]. The following is a sample item: “Please indicate the number that applies best to you: On the whole, I am satisfied with myself.” The internal consistency of this scale was α = 0.91 in Sample A and α = 0.87 in Sample B. Response options ranged from 1 = “not at all” to 4 = “completely”. The summed score was computed for analyses, with higher scores indicating higher levels of self-esteem.

#### Socio demographic and clinical characteristics

Age was measured in years. For gender, response options were “male”, “female”, or “others”. The current severity of depression symptoms was measured via the German version of the Patient Health Questionnaire (PHQ-9 [22]). The scale consists of 9 items introduced by “How often have they been bothered by the following over the past 2 weeks?” The following is a sample item: “Little interest or pleasure in doing things?” Internal consistencies were α = 0.86 (Sample A) and α = 0.84 (Sample B). The response scale ranged from 1 = “not at all” to 4 = “nearly every day”. The summed score was computed for analyses, with higher scores indicating higher levels of current depression symptoms.

### Statistical analyses

IBM SPSS statistics 24 was used to examine Cronbach’s alphas, descriptive statistics, and non-parametric Spearman correlations between model variables. For interval variables, group differences regarding all variables were tested with Mann-Whitney-U tests because of non-normal distributions of variables in samples. For nominal variables, Pearson chi-square tests based on cross-tabulations were used.

To test the trickle-down hypothesis, Kruskal-Wallis 1-way ANOVAs were used to compare distributions of the three SSMIS subscales, followed by Dunn-Bonferroni post hoc tests to identify which of the subscales differed significantly. Pearson’s correlation coefficient (*r* = z / (√n)) was computed for effect sizes; r = 0.10 indicated a weak effect, r = 0.30 indicated a medium effect, and r = 0.50 indicated a strong effect [23].

A specific kind of structural equation model–a serial mediation model–was tested using the macro for model 6 of the PROCESS procedure for SPSS Version 3.00 by Andrew F. Hayes [24] to explore the process character of self-stigma, from the independent variable (stereotype awareness) to the outcome (self-esteem) via two potential mediators (personal agreement and self-concurrence) and controlling for age, gender, and current level of depression as covariates. This kind of model is especially well-suited for process-oriented research since several direct and indirect paths can be tested in one single model based on ordinary least squares (OLS) regression analyses. Classic assumptions (linear multicollinearity, multivariate normality of residuals, homoscedasticity) of OLS regression were tested in preliminary analyses. Outliers were identified with stem-and-leaf-plots indicating interquartile ranges computed from Tukey’s hinges.

The PROCESS procedure is described in detail by Hayes [25]. The mediation model included one primary direct path (1: from stereotype awareness to self-esteem), five other direct paths (2: from stereotype awareness to personal agreement; 3: from stereotype awareness to self-concurrence; 4: from personal agreement to self-concurrence; 5: from personal agreement to self-esteem; and 6: from self-concurrence to self-esteem), and three indirect paths (1: from stereotype awareness via personal agreement to self-esteem; 2: from stereotype awareness via self-concurrence to self-esteem; and 3: from stereotype awareness via personal agreement via self-concurrence to self-esteem). Asymmetric bootstrap confidence intervals were used to avoid power problems with asymmetric or other forms of non-normal distributions of variables or outliers: The main assumption of bootstrapping analyses is a representative sample of the population, since bootstrapping artificially recreates a population based on the initial sample. Testing the hypotheses in two independent samples challenges the representativeness of the samples and as such tests the robustness of results. To receive standardized coefficients for the six direct paths, variable scores were z-standardized prior to model fitting.

## Results

### Sample characteristics

Table 1 depicts the socio-demographics, clinical characteristics, and stigma attitudes of both samples. In Sample A, participants were between thirty and forty years old on average, the majority were female, less than half were single, and two-thirds had a relatively high level of education. Most participants were currently in outpatient treatment, only a few were in inpatient treatment, and a quarter was neither in outpatient nor in inpatient treatment. In Sample B, the average age was between thirty and forty years, slightly more than half was female, half were single, and less than half had a higher level of education. Half of the participants were currently in outpatient and half in inpatient treatment.

**Table 1:** Sample characteristics.

	Sample A	Sample B	Group Differences
sample size	550	180	
age in years (mean, standard deviation)	37.3 (13.2)	38.8 (12.6)	z = -1.495, p = 0.135
gender (female) (%)	83.8	58.9	χ^2^ = 48.597; df = 1; p<0.001
marital status (%)			χ^2^ = 0.583; df = 2; p = 0.747.
single	46.7	50.0	
in partnership	26.5	25.0	
others	26.7	25.0	
level of education (%)			χ^2^ = 33.183; df = 1; p<0.001
< 12 years of school education	33.1	57.2	
≥ 12 years of school education	66.9	42.8	
current type of mental health care (%)			χ^2^ = 251.370; df = 2; p<0.001
outpatient	71.5	50.0	
inpatient	3.5	50.0	
not applicable	25.1	0.0	
current severity of depression (PHQ-9) (mean, standard deviation)	14.89 (6.11)	13.92 (5.73)	z = -2.108, p = 0.035
awareness (mean, standard deviation)	52.51 (16.83)	46.81 (18.25)	z = -3.772, p<0.001
agreement (mean, standard deviation)	24.21 (11.48)	24.66 (12.71)	z = -0.133, p = 0.894
self-concurrence (mean, standard deviation)	27.23 (14.26)	25.92 (12.90)	z = -0.706, p = 0.480
self-esteem (mean, standard deviation)	13.12 (7.35)	14.19 (6.18)	z = -2.249, p = 0.025

Group differences were tested with Mann-Whitney-U-Tests for ordinal and interval scaled variables, and with Pearson Chi^2^ Tests based on Crosstables for nominal scaled variables. PHQ-9 = depression scale of the Patient Health Questionnaire; awareness = stereotype awareness; agreement = personal agreement.

Samples differed significantly regarding gender (with more females in Sample A), level of education (with higher levels of education in Sample A), and current type of mental health care (with more inpatient participants in Sample B). While higher levels of stereotype awareness and current depression symptoms were reported by Sample A, a higher level of self-esteem was reported by Sample B. There was only one extreme outlier in Sample A regarding personal agreement. Although its value was more than three interquartile ranges (IQR) from the end of a box, the data set was retained for analyses since bootstrapping procedures are quite robust against these outliers, especially if outliers comprise less than 5% of the data (for data details, please refer to Supporting Information “S1 File” and “S2 File”).

### Preliminary analyses

Kruskal-Wallis 1-way ANOVAs indicated significant differences between the SSMIS subscales in both samples (Sample A: χ^2^(2) = 653.991, *p*<0.001; Sample B: χ^2^(2) = 152.101, *p*<0.001). Dunn-Bonferroni post hoc tests revealed significantly higher levels of stereotype awareness than personal agreement (Sample A: z = 23.307, *p*<0.001, *r* = 0.57; Sample B: z = 11.085, *p*<0.001, *r* = 0.48) and self-concurrence (Sample A: z = 20.768, *p*<0.001, *r* = 0.51; Sample B: z = 10.224, *p*<0.001, *r* = 0.44). At the significance level of *p*<0.05, personal agreement was rated lower than self-concurrence in Sample A (z = -2.540, *p* = 0.033, *r* = 0.06) but not in Sample B (z = -0.861, *p* = 1.000). Table 2 presents bivariate non-parametric Spearman correlations between all variables.

**Table 2:** Bivariate nonparametric Spearman correlations of model variables.

		1	2		3		4		5		6		7	
1	gender	--	0.105	*	0.009		-0.090	*	0.115	**	0.120	**	0.057	
2	age	-0.015	--		-0.114	**	-0.039		-0.089	*	-0.197	***	0.292	***
3	depression	-0.052	-0.001		--		0.170	***	0.196	***	0.439	***	-0.601	***
4	awareness	0.026	-0.143		0.135		--		0.176	***	0.149	***	-0.184	***
5	agreement	0.020	-0.038		0.111		0.228	**	--		0.574	***	-0.200	***
6	self-concurrence	0.020	-0.010		0.470	***	0.163	*	0.401	***	--		-0.469	***
7	self-esteem	0.098	0.155	*	-0.589	***	-0.247	**	-0.205	**	-0.458	***	--	

Sample A above the diagonal, Sample B below the diagonal; depression = current level of depression; awareness = stereotype awareness; agreement = personal agreement

***p<0.001

**p<0.01

*p<0.05.

**Fig 2:**
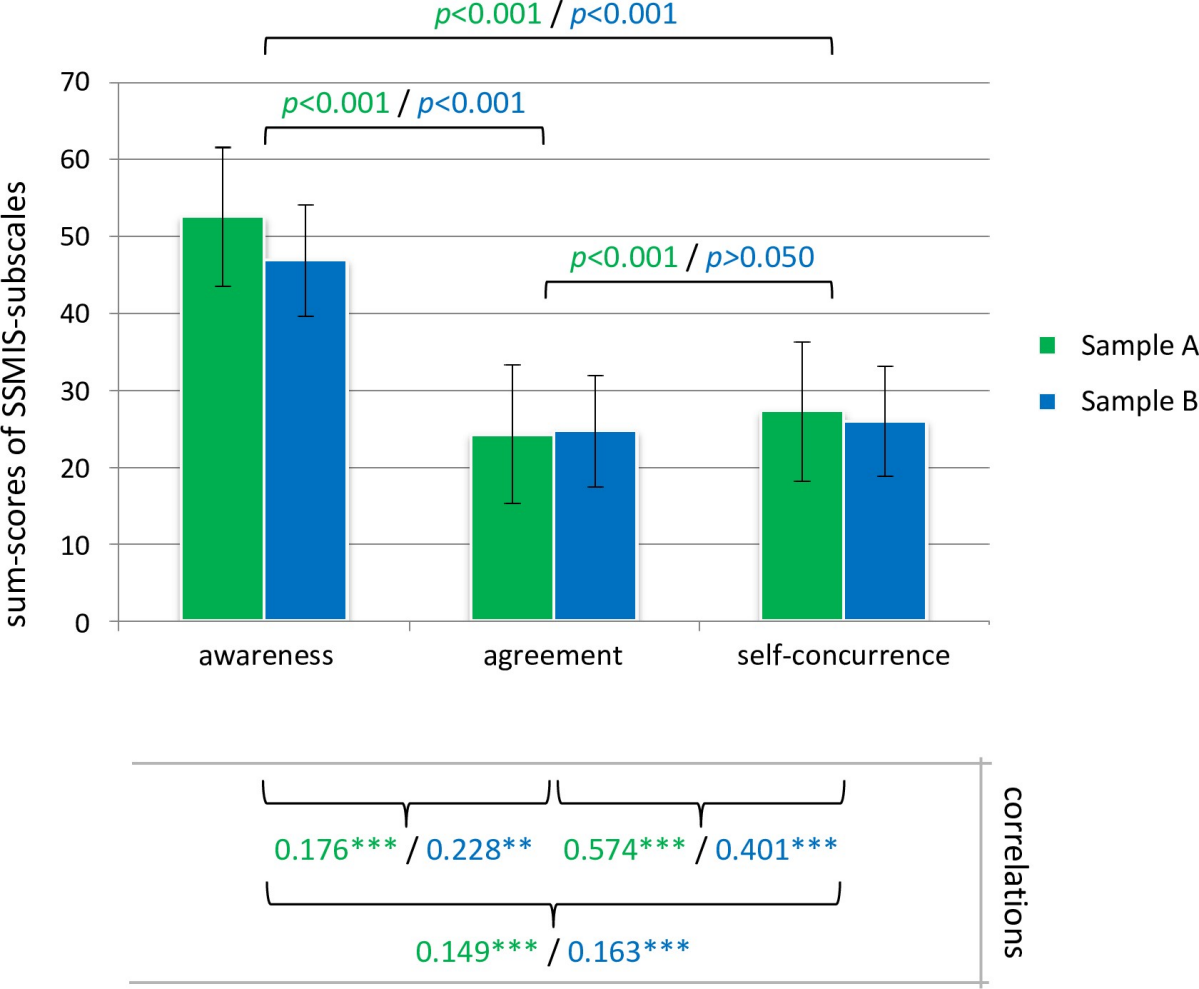
Summed scores and direct associations of stigma attitudes in Sample A and Sample B. The summed scores of SSMIS subscales ranged from 10 to 90. *p* = significance of Dunn-Bonferroni post hoc tests (Sample A/Sample B). Below the graph are bivariate non-parametric Spearman correlations representing associations between the three subscales (Sample A/Sample B). ***p<0.001; **p<0.01; *p<0.05.

Sample A and Sample B showed similar patterns: stereotype awareness was positively correlated with both personal agreement and self-concurrence and negatively with self-esteem; personal agreement was positively correlated with self-concurrence and negatively correlated with self-esteem. Proximal phases were more highly correlated than distal phases, with the exception of one association. While personal agreement and self-concurrence were more highly correlated than were stereotype awareness and personal agreement, stereotype awareness was more highly correlated with self-esteem than with personal agreement and self-concurrence. Compared to stereotype awareness and personal agreement, self-concurrence was most strongly correlated with self-esteem. Please refer to Fig 2 for a summary of the results of hypotheses I and II.

### Mediation analyses

**Fig 3:**
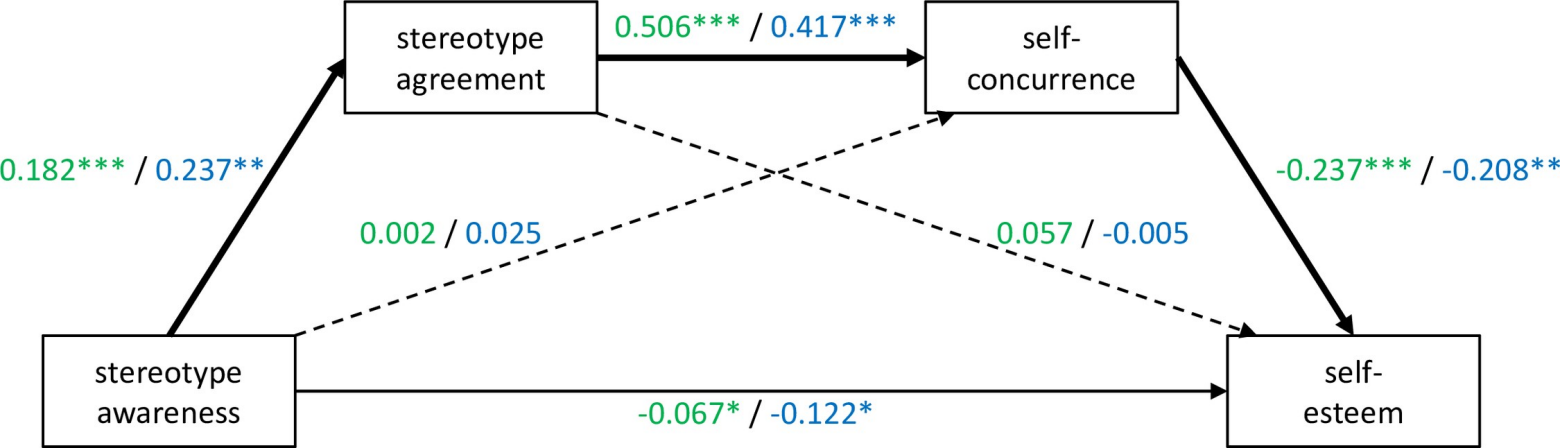
Serial mediation model. Solid lines indicate significant direct paths. Thick solid lines indicate significant indirect paths. Dotted lines represent non-significant direct paths. Standardized coefficients are presented (Sample A/Sample B). ***p<0.001; **p<0.01; *p<0.05.

All OLS assumptions were fulfilled for z-standardized variables in both samples. In Fig 3, the serial mediation model is presented for both samples indicating standardized coefficients of direct paths controlled for age, gender, and current level of depression.

In both samples, four direct pathways were significant: from stereotype awareness to self-esteem; from stereotype awareness to personal agreement; from personal agreement to self-concurrence; and from self-concurrence to self-esteem. Neither the direct effect of stereotype awareness on self-concurrence nor the direct effect of personal agreement on self-esteem was significant.

There was one significant indirect pathway (*p*<0.05, i.e., bootstrap bias-corrected confidence interval does not include zero) in both samples, starting from stereotype awareness via personal agreement and then self-concurrence to self-esteem (estimated indirect effects and 95% bootstrap confidence intervals -0.022 (-0.038, -0.010) for Sample A and -0.021 (-0.045, -0.005) Sample B). This indirect effect implied that an increase of one standard deviation in stereotype awareness was associated with an increase of 0.182 (Sample B: 0.237) standard deviations in personal agreement and then with an increase in self-concurrence of 0.182x0.506 = 0.092 (Sample B: 0.237x0.417 = 0.099) standard deviations and resulted in an increase of 0.182x0.506x0.237 = -0.022 (Sample B: 0.237x0.417x-0.208 = -0.021) standard deviations in self-esteem. All other indirect paths were non-significant (for details on mediation results, please refer to Supporting Information “S4 File”).

## Discussion

The aim of this study was to investigate the progressive model of self-stigma by Corrigan [15], which represented the four stages of stereotype awareness, personal agreement, self-concurrence, and self-esteem. Data were collected from two independent samples of people with depressive symptoms: one online sample (only self-report) and one face-to-face sample (inclusion criteria were screened by the treating staff). Two main assumptions of the model were examined: (I) a trickle-down in nature, i.e., stigma attitudes are endorsed decreasingly, with the highest endorsement for stereotype awareness, followed by lower endorsements for each stage; and (II) proximal stages are more strongly associated than more distant stages. Controlling for age, gender, and current depressive symptoms, the study’s main contribution was (III) serial mediation analyses, which should shed light on the procedural character of the model.

The results of the study indicate a trickle-down effect for the first stage; only endorsements for stereotype awareness were higher than for both personal agreement and self-concurrence in both samples. This result is supported by those of several other studies with students and general population samples (e.g., [26, 27]). The respondents’ beliefs about the stigmatizing attitudes of others seem to be consistently higher than personal beliefs and attitudes towards mental illness. However, the results regarding stages of personal agreement and self-concurrence are less uniform: in the online sample of the present study, there was an unexpected statistically significant difference between endorsements of personal agreement and self-concurrence. Endorsements for personal agreement were lower than those for self-concurrence. The effect size indicated a weak effect and thus not clinically relevant, and in the face-to-face sample, there was no difference in endorsements for personal agreement and self-concurrence at all. At the same time, there were significant differences in endorsements of personal agreement or self-concurrence between the two samples. The Kruskal-Wallis test conducted depends on sample sizes. Statistical differences in results may therefore be due to sample size differences. The results by Corrigan and colleagues, on the other hand, indicate higher endorsements for personal agreement than for self-concurrence [15]. They collected data from a more heterogeneous sample of varying diagnoses, which might explain the differing results of this study as schizophrenia was found to be more strongly associated with self-stigma than with depression [28]. Future research may explore diagnosis-specific associations.

In line with hypothesis II and earlier research [15], all proximal stages were more strongly associated than were more distant stages, with the exception of one association. Stereotype awareness was more strongly associated with self-esteem than with the more proximal stages of personal agreement and self-concurrence. As stereotype awareness refers to others’ rather than one’s own attitudes, associations might be weaker with personal agreement and self-concurrence, which represent one’s opinion rather than perception only. Considering stereotype awareness, a separate construct is in line with other research that differentiates between different kinds of public stigma [29, 30].

The main contribution of this study is the investigation of the procedural character of the stages. As indicated by serial mediation analyses, direct associations of distant stages were mediated by proximal stages. While higher levels of stereotype awareness were associated with higher levels of stereotype agreement, higher levels of stereotype agreement in turn were associated with higher levels of self-concurrence, which in turn were associated with lower levels of self-esteem. Significant correlative associations between stereotype awareness and self-concurrence and between personal agreement and self-esteem became insignificant when integrated into one serial mediation model. The significant primary direct path of stereotype awareness to self-esteem indicates partial but not full mediation. To test the robustness of the results, additional analyses were run for both samples as a whole (for details please refer to Supporting Information “S5 File”). The results do not differ from the results reported above. It can therefore be concluded that the model can be applied to individuals with depression in general.

These results are in line with other research on people with mental illness, supporting the assumption of self-stigma as a multilevel process [16]. Stereotype awareness of mental seems to have independent associations to general self-esteem among self-stigma attitudes. However, because correlations of personal agreement and self-concurrence were very high and summed scores differed only in one sample with a weak effect, the following question remains: in what ways do personal agreement and self-concurrence represent differing constructs? Some intervention studies imply differential effects on specific stigma attitudes. Goepfert et al. investigated direct effects that media reporting could have on people with depression [17]. Their results indicate that even single short film presentations of potentially stigmatizing TV news have an impact on personal agreement but not on other stigma attitudes. Kohls et al. investigated the impact of the Optimizing Suicide Prevention Programs and Their Implementation in Europe (OSPI Europe) in four European regions [31]. They concluded that stigma awareness was more difficult to change than stigma agreement but did not look at self-concurrence. Therefore, compared to the other two stigma-measures, personal agreement appears to be a more readily modifiable factor.

Several other limitations of this study indicate the need for further research in the conceptualization and intervention regarding self-stigma in people with depression. First, the cross-sectional design does not allow for any interpretations of causal effects. Although the results support a procedural character of the model, no predictive conclusions can be drawn from this study. Longitudinal studies could more thoroughly examine the process over time and would be necessary for predictive statements.

Second, the online sample brings along some challenges of any online survey, e.g., it is only based on self-reports. As a result, there is a lack of precise information about diagnoses and the monitoring of participation. The second sample with face-to-face contact and documented clinical data offers a more controlled design of the data collection.

As previously mentioned, Corrigan and colleagues [15] postulated a fourth stage of self-stigma in his progressive model, namely harm to self (i.e. self-esteem decrement). The corresponding subscale in their original SSMIS directly refers to stigma contents (e.g. “I currently respect myself less because I am dangerous.”). As we used a self-esteem scale other than the one from the SSMIS, the content is less related to stigma-attitudes. It can rather be considered as a consequence of the first three phases instead of a fourth phase. This is why a trickle-down could not be investigated for self-stigma in the study under investigation.

Another major limitation is the lack of measures for the external validity of the progressive model of self-stigma. Neither risk nor protective factors (for examples see [12, 19, 32]) nor consequences (such as seeking professional help or adherence to treatments) of stigma-measures were covered. It would be worthwhile to investigate the specificity of predictors and outcomes of different stages of self-stigma both for prevention and treatment programmes.

The results of this study support Corrigan’s progressive model of self-stigma [15] in people with depression in most respects with few exceptions. It offers a theoretical foundation for process research of self-stigma. Different stages of self-stigma may require specific consideration in their prediction, consequences, and potential interventions: the latter the stage is the more grave the effect on harm to self.

## Supporting information

S1 File Data total sample. (PDF)

S2 File Data trickle down. (PDF)

S3 File Syntax. (PDF)

S4 File Results serial mediation models. (PDF)

S5 File Results total sample. (PDF)
