# Supplementary material for: Applying Corrigan’s progressive model of self-stigma to people with depression
Source: PLoS One. 2019 Oct 29;14(10):e0224418. doi: 10.1371/journal.pone.0224418 (PMC6818799; doi:10.1371/journal.pone.0224418)
Supplement: S1 File — (PDF) [file pone.0224418.s001.pdf]

CASE	PUB01_B	PUB02_B	PUB03_B	PUB04_B	PUB05_B	PUB06_B	PUB07_B	PUB08_B
125	7	8	7	6	6	5	5	4
132	8	6	9	5	4	7	6	5
133	9	8	7	2	5	5	3	7
140	2	2	1	1	1	1	1	1
141	3	7	4	4	4	3	5	2
143	6	8	7	5	3	7	4	5
144	8	6	6	7	5	6	5	4
146	4	7	7	5	7	7	3	4
156	6	7	6	4	4	6	4	1
157	2	1	1	1	2	1	1	1
158	8	7	8	6	8	7	8	7
159	9	9	7	7	9	8	9	8
168	8	7	7	2	2	5	6	4
173	9	9	8	7	9	8	4	5
191	6	7	9	3	5	4	2	2
194	4	2	2	3	7	3	2	2
202	2	6	6	3	3	2	6	1
204	6	1	5	7	6	1	2	7
214	6	6	6	4	6	7	6	4
215	8	7	4	2	5	4	7	2
216	7	5	6	3	5	6	4	4
224	7	6	6	5	6	6	5	3
232	3	6	2	1	7	1	5	1
235	7	9	7	5	5	7	7	5
239	6	8	7	5	3	4	2	2
250	7	6	4	5	2	2	1	1
257	7	7	8	1	5	7	9	4
274	1	1	1	1	1	1	1	1
290	1	9	9	1	5	5	1	1
302	9	8	8	1	7	8	1	1
304	8	2	5	6	8	5	5	6
305	6	6	7	6	4	5	5	4
307	7	8	8	4	6	8	6	3
308	4	6	8	6	5	8	5	5
309	6	6	7	3	6	7	3	4
310	1	9	8	4	7	1	7	1
314	9	6	8	5	5	6	2	2
318	1	9	1	1	5	1	1	1
340	6	6	8	5	8	9	5	6
344	7	6	8	6	8	4	4	1
347	5	7	7	1	5	5	1	1
350	6	3	4	1	1	2	3	1
355	2	2	2	1	1	1	1	1
358	8	8	7	7	6	6	6	6
361	2	5	1	1	1	1	1	1
363	7	8	3	3	7	1	2	1
372	9	5	7	8	2	8	7	1
373	8	5	8	7	7	7	7	4
374	6	7	8	1	5	7	6	1

377	7	8	8	6	8	8	5	2
386	9	9	9	9	9	9	9	9
389	7	5	7	5	3	6	6	7
396	8	6	7	2	6	2	2	1
397	7	5	3	1	5	3	1	1
402	2	4	3	1	5	1	1	1
404	8	8	6	6	6	3	3	1
407	8	8	8	1	8	5	5	2
423	7	8	7	2	4	3	1	1
425	2	6	6	1	1	1	1	1
430	5	6	6	5	3	2	5	2
432	7	7	2	1	2	1	1	1
433	4	6	5	2	7	5	6	2
435	6	7	6	6	6	7	5	5
438	9	8	7	3	2	4	7	2
445	5	8	2	1	3	4	2	1
448	7	7	8	5	7	7	6	6
459	7	6	5	4	5	5	5	3
460	8	8	8	6	8	9	9	3
466	7	6	7	5	6	6	7	4
467	1	5	5	3	4	2	1	1
468	7	9	8	6	9	8	4	6
470	6	6	3	3	3	3	2	2
475	6	7	9	4	7	6	5	3
476	8	8	5	2	2	6	1	5
477	6	8	6	4	6	4	5	2
479	9	9	9	6	6	9	9	6
480	9	9	7	9	9	7	9	9
481	6	7	7	6	6	7	5	5
484	7	7	7	6	6	7	6	5
486	2	6	7	7	3	4	6	2
487	2	4	5	3	5	4	3	1
488	7	7	5	7	5	5	5	5
490	8	6	8	4	5	8	6	3
491	6	7	4	2	3	4	1	2
500	6	8	6	7	8	6	6	4
502	6	7	4	4	5	7	5	5
503	8	8	9	6	8	7	7	6
504	1	1	9	5	5	5	1	1
506	7	8	7	6	8	6	3	3
507	6	6	7	3	4	3	1	3
508	5	7	6	4	6	6	3	3
512	8	7	7	5	7	7	4	4
514	8	8	8	8	8	8	4	5
517	7	5	6	5	5	3	3	3
519	3	5	7	3	5	5	5	5
520	5	6	5	4	4	3	3	2
526	8	6	5	3	6	3	3	1
528	7	8	8	5	9	5	6	7
529	8	7	8	5	7	8	7	5

532	5	5	5	5	3	3	3	3
538	5	1	2	1	5	5	1	1
539	9	3	1	1	5	1	1	1
540	9	9	5	7	6	4	6	6
544	3	3	4	3	6	6	3	1
546	8	6	6	1	6	7	3	4
565	6	6	4	4	6	4	3	3
569	7	8	7	7	3	6	6	8
574	6	5	7	5	4	8	5	6
576	8	5	7	5	4	3	2	2
578	8	9	6	1	3	5	5	6
579	7	4	9	6	7	8	9	6
580	4	3	2	2	2	2	1	1
597	6	8	4	5	7	3	3	2
609	7	9	5	3	6	5	6	1
618	8	8	8	8	5	5	7	5
619	8	6	7	6	7	3	1	1
623	7	7	7	6	7	7	5	6
632	6	2	1	1	1	1	1	1
643	8	7	7	5	7	6	5	5
655	5	7	5	6	7	1	1	1
663	2	4	2	1	2	1	1	1
664	7	7	9	6	6	6	7	3
665	7	3	6	2	5	2	3	1
686	6	4	5	3	5	8	5	2
689	6	4	3	2	6	2	1	1
691	7	5	7	6	4	4	5	4
699	9	9	9	6	9	9	1	1
701	7	7	5	4	6	8	8	3
704	7	7	8	8	8	7	7	7
714	9	9	5	3	5	2	7	2
715	8	7	7	5	5	7	3	3
716	1	1	5	1	5	1	1	1
720	8	9	8	3	8	8	7	7
724	3	2	5	3	4	3	3	2
726	8	9	9	3	5	8	7	7
727	6	8	8	1	5	6	1	1
729	5	5	7	5	5	7	5	7
732	7	6	8	5	3	3	4	2
733	7	6	6	5	4	7	4	3
740	8	6	7	7	7	6	4	6
760	2	2	3	3	1	1	1	1
762	7	6	8	7	5	6	7	5
764	7	3	8	6	5	6	6	6
769	2	4	5	2	4	2	2	5
773	7	3	2	2	2	4	1	4
774	9	3	6	1	6	1	1	1
775	8	8	8	7	7	3	3	6
776	6	8	6	5	6	5	6	3
778	6	9	9	6	7	7	7	6

779	7	5	6	5	5	6	6	5
781	9	9	9	9	9	9	5	5
792	1	1	1	1	1	1	1	1
800	9	7	6	6	7	8	7	5
806	9	3	9	5	2	5	5	5
835	3	2	2	1	1	1	1	1
836	7	7	7	7	7	7	5	3
838	8	5	7	4	6	6	4	4
853	7	7	7	1	7	2	1	1
856	9	7	9	5	6	5	5	2
858	7	6	7	7	5	6	5	6
860	3	6	6	6	7	6	6	4
863	8	8	6	5	3	6	7	3
875	7	6	7	7	3	5	5	2
876	7	5	8	2	6	6	1	2
879	9	8	6	7	7	8	5	4
881	6	4	7	2	3	2	4	1
883	9	9	2	1	1	1	1	1
885	7	7	7	6	7	7	3	4
908	1	1	1	1	1	1	1	1
913	8	8	8	6	2	1	1	1
922	4	4	7	1	5	3	4	2
929	8	3	4	3	3	4	6	2
930	4	7	6	4	8	4	5	3
931	5	8	9	7	8	8	5	5
932	5	7	5	2	5	2	5	1
934	6	7	6	7	6	8	8	6
945	8	8	4	6	3	7	5	2
949	8	5	8	7	6	8	6	7
955	6	3	9	2	1	1	1	1
960	6	5	7	4	7	7	5	3
964	8	7	6	3	5	5	3	2
969	8	6	3	2	7	7	1	4
970	8	8	2	1	7	5	2	4
971	6	8	9	6	6	6	7	8
973	4	2	4	2	7	5	1	1
974	8	8	9	5	8	9	3	1
978	9	5	2	5	8	8	7	3
981	9	5	5	5	7	3	3	6
983	5	6	6	1	3	7	1	1
986	7	7	5	3	7	4	5	1
993	7	2	7	5	5	8	6	5
998	5	3	2	2	2	1	1	1
999	8	5	1	1	1	1	1	1
1001	9	9	9	9	9	9	9	9
1008	4	5	6	1	5	5	1	1
1021	7	7	8	8	7	6	6	5
1030	4	4	7	3	2	2	2	3
1033	9	9	5	1	9	7	1	1
1037	7	4	8	6	7	8	7	3

1040	9	9	9	9	9	9	9	9
1046	9	8	6	6	8	6	6	6
1049	5	8	5	5	1	1	5	1
1051	7	3	7	5	5	7	6	5
1054	9	8	1	1	6	1	6	1
1055	2	3	4	1	3	1	1	1
1058	9	9	9	9	6	9	9	7
1060	8	7	7	5	7	7	5	7
1067	4	5	3	5	2	5	5	5
1072	5	9	7	7	7	5	9	4
1076	9	7	8	6	4	6	6	7
1077	7	7	8	6	5	4	5	4
1078	5	8	8	7	3	6	7	5
1079	8	4	8	4	5	6	7	4
1084	8	6	2	1	4	1	1	1
1087	9	9	9	9	9	9	9	9
1094	6	6	7	2	8	3	5	1
1096	5	5	5	5	5	5	5	5
1100	1	1	1	1	1	1	1	1
1105	7	7	7	7	5	7	5	6
1106	7	6	7	4	3	1	1	1
1107	7	5	5	1	1	5	4	1
1109	1	8	5	5	7	5	5	5
1119	7	6	9	3	9	9	4	7
1120	6	6	8	5	7	7	5	5
1123	6	4	2	1	2	2	1	1
1124	9	9	5	5	9	5	5	5
1125	9	7	9	7	8	8	7	9
1126	9	7	9	5	5	8	1	5
1131	7	8	7	6	8	8	8	4
1132	7	5	8	7	6	7	8	4
1133	6	7	7	3	5	6	6	2
1140	1	7	9	5	8	7	4	4
1141	9	5	9	5	9	9	5	7
1144	1	1	1	1	1	1	1	1
1149	7	8	7	3	4	2	4	1
1150	8	5	6	4	5	7	8	5
1152	7	6	2	2	2	1	1	1
1155	8	7	8	6	9	8	2	5
1167	8	8	8	7	6	8	6	7
1169	5	7	3	4	5	4	1	4
1178	8	8	8	8	8	8	8	8
1180	1	5	2	1	5	7	1	1
1181	8	9	9	9	9	9	9	9
1191	7	7	7	5	5	6	2	2
1195	9	9	6	7	1	4	1	2
1206	7	8	9	7	7	6	4	2
1210	9	9	1	1	1	1	1	1
1212	6	5	5	2	2	1	1	1
1224	1	1	2	1	2	1	7	3

1235	8	2	2	1	7	5	2	1
1241	6	6	7	3	6	7	3	5
1242	9	9	9	7	8	9	5	7
1245	3	4	2	1	2	3	2	1
1246	5	2	9	9	9	9	3	5
1247	8	8	6	5	6	5	3	4
1249	7	5	5	1	7	2	1	1
1252	8	7	8	3	4	7	3	4
1253	7	9	6	6	8	2	7	2
1255	7	8	8	5	6	7	6	5
1258	8	8	8	4	4	3	1	1
1261	6	7	7	3	7	5	5	2
1263	9	5	5	9	9	6	7	4
1268	9	9	9	8	9	8	8	8
1274	9	6	5	5	3	3	5	1
1281	6	7	9	5	2	5	6	1
1282	6	7	7	5	8	7	4	5
1289	1	7	1	5	3	1	1	1
1290	8	7	7	6	5	5	6	3
1295	8	8	8	5	6	5	3	5
1309	3	4	8	2	1	6	2	4
1310	6	6	6	5	5	7	6	7
1316	7	6	7	3	4	3	1	3
1319	8	8	5	6	6	6	8	5
1328	2	1	1	1	1	1	1	1
1340	9	8	6	3	8	4	5	1
1363	9	9	9	9	5	5	9	9
1366	7	5	6	5	6	7	4	4
1367	7	3	6	4	6	7	2	5
1368	1	2	3	1	1	1	1	1
1369	3	5	8	2	5	6	2	2
1374	5	6	6	2	2	3	1	1
1376	8	4	4	8	2	7	9	9
1382	1	3	5	1	6	2	1	1
1383	7	8	9	8	9	9	9	7
1393	5	7	8	6	5	6	4	5
1394	1	5	9	1	5	1	1	1
1395	6	3	4	7	9	7	8	7
1401	2	1	3	4	3	2	4	3
1405	3	6	8	6	8	8	4	2
1406	7	8	9	8	6	8	7	6
1409	8	3	6	1	4	1	1	1
1410	7	6	4	5	7	5	4	3
1413	5	4	3	1	1	4	1	2
1416	6	9	9	5	8	8	8	5
1419	7	6	9	7	5	8	5	5
1443	5	6	6	3	3	4	7	1
1445	7	6	6	6	6	3	6	3
1447	8	3	3	6	5	6	2	3
1449	3	2	1	1	2	1	1	4

1451	7	7	4	3	5	5	6	2
1460	8	8	8	8	8	8	8	7
1464	7	9	9	6	9	9	9	6
1477	8	7	7	4	4	4	2	2
1493	4	7	7	6	5	7	4	3
1502	7	9	9	7	7	9	8	7
1503	8	7	8	3	9	5	2	3
1521	1	1	1	1	1	1	1	1
1525	7	7	6	5	5	6	4	3
1527	8	6	6	9	8	7	1	5
1530	8	8	5	4	9	5	5	5
1531	6	8	6	5	6	6	5	5
1533	7	6	4	2	3	5	2	2
1543	7	7	7	8	5	8	9	7
1544	5	7	4	4	6	2	6	3
1548	8	6	6	6	8	6	5	2
1550	7	5	9	5	8	9	3	6
1551	7	6	8	5	2	6	1	6
1553	7	2	2	2	7	1	5	5
1554	7	8	8	6	5	6	7	2
1555	5	7	2	1	7	1	2	1
1556	6	5	7	5	3	5	2	1
1559	5	8	7	2	6	2	7	2
1560	1	5	2	1	1	1	1	1
1579	7	7	7	3	2	2	2	2
1586	9	5	7	7	5	7	8	5
1588	8	7	8	6	5	9	7	5
1609	7	8	6	4	7	8	1	3
1619	8	8	8	7	9	6	6	5
1625	9	9	9	9	5	9	9	5
1626	9	8	6	3	8	2	4	2
1635	1	2	5	1	4	1	1	1
1642	5	7	5	3	7	7	3	1
1646	9	9	9	7	9	7	7	1
1656	8	7	8	7	4	8	5	7
1664	7	7	7	3	7	6	7	5
1672	6	4	3	5	7	3	3	3
1688	8	9	8	2	9	9	8	7
1696	9	9	9	5	9	5	5	5
1703	7	9	9	6	7	7	5	5
1709	6	6	8	5	4	4	1	2
1716	9	8	9	7	6	8	7	6
1718	7	7	5	4	7	6	3	2
1725	7	6	3	5	8	4	9	6
1738	9	9	9	9	8	9	9	7
1740	6	8	9	7	5	6	3	3
1745	7	8	8	4	6	5	7	6
1747	3	3	3	3	3	3	3	3
1759	7	8	7	7	7	8	7	7
1766	7	7	6	7	5	5	6	6

1768	8	9	7	5	5	6	8	6
1769	8	7	8	6	7	7	5	6
1773	8	8	8	8	6	6	7	3
1775	1	5	1	5	1	1	1	1
1776	8	6	5	4	5	4	5	4
1777	7	5	4	4	3	3	2	5
1784	7	9	9	5	5	1	1	1
1788	2	2	3	1	1	2	1	1
1793	7	5	7	4	8	8	3	5
1798	9	7	7	3	8	3	8	2
1818	9	4	4	4	4	4	4	4
1821	9	8	8	6	7	7	7	5
1827	8	8	3	1	6	7	1	1
1828	8	6	7	3	6	6	7	4
1829	3	6	7	3	3	6	3	3
1830	8	3	7	1	6	6	2	7
1831	2	6	5	5	5	2	5	1
1833	9	2	6	2	7	7	7	8
1837	8	9	8	5	7	8	8	9
1866	7	8	6	7	8	7	5	4
1872	8	7	5	5	7	6	4	5
1874	9	7	8	8	8	9	5	6
1875	4	4	4	2	6	2	6	1
1878	7	9	9	2	5	2	5	1
1900	7	6	5	3	6	5	3	5
1910	8	5	6	4	6	6	3	3
1914	7	7	2	1	2	2	2	1
1929	3	4	4	1	2	2	2	1
1930	9	8	1	9	6	5	5	5
1932	9	6	7	8	8	9	6	7
1935	6	5	6	6	6	5	5	3
1936	9	6	7	7	5	6	8	3
1941	2	9	9	8	8	8	9	6
1950	7	7	6	5	6	6	7	4
1981	9	2	7	3	9	1	1	1
1982	6	6	6	6	6	6	5	5
1984	7	7	7	7	7	7	7	5
1987	7	6	7	4	6	3	7	6
1994	8	6	6	4	6	5	4	5
1995	8	2	7	6	9	8	9	7
2001	1	2	2	1	1	1	1	1
2003	7	6	2	1	7	3	4	1
2005	1	3	5	1	1	1	9	3
2006	9	7	6	1	7	7	5	1
2008	4	7	8	5	6	4	5	2
2010	6	4	5	6	7	7	5	6
2013	3	3	2	1	5	3	1	1
2014	8	7	7	5	7	9	5	5
2017	4	4	5	5	5	5	5	5
2021	7	9	8	2	7	9	2	2

2022	7	3	5	3	3	5	3	3
2023	5	5	5	5	2	5	9	5
2024	9	7	7	3	1	3	1	1
2028	6	4	7	1	7	7	1	1
2030	9	9	9	9	9	9	9	9
2032	8	8	6	5	5	6	8	5
2033	4	7	5	6	3	2	3	2
2050	8	9	9	7	5	3	2	1
2052	7	8	8	8	7	9	5	2
2054	7	7	5	3	5	7	7	3
2077	7	4	9	6	3	6	6	1
2081	9	9	5	1	5	5	5	5
2088	8	3	2	2	3	4	2	2
2090	9	5	9	9	1	6	3	1
2093	8	7	9	5	6	9	5	5
2096	9	7	7	5	7	1	1	1
2099	8	6	4	2	6	6	5	1
2108	6	8	6	4	3	6	5	2
2109	9	7	7	6	6	6	8	7
2118	1	1	1	1	5	1	1	1
2123	7	2	7	3	5	7	7	1
2124	5	7	6	1	3	1	1	1
2125	7	5	5	6	9	5	7	1
2127	7	6	7	5	6	4	6	3
2129	9	7	5	3	5	5	2	2
2130	7	6	6	3	5	6	2	3
2132	9	8	9	8	8	8	8	7
2133	5	6	6	6	4	4	2	2
2136	8	8	8	5	7	7	6	5
2137	7	9	8	4	6	6	3	2
2146	8	6	5	3	5	6	5	1
2170	6	5	8	2	5	8	9	2
2180	1	5	1	1	1	1	1	1
2188	5	5	5	2	7	5	2	1
2195	7	7	3	1	5	3	2	3
2204	6	8	8	6	2	8	4	5
2205	3	5	2	2	3	2	1	1
2207	9	9	9	6	6	7	4	8
2222	8	6	6	5	8	5	3	3
2223	6	7	7	2	6	2	3	3
2232	4	3	2	1	3	3	3	1
2235	8	8	7	7	7	7	8	7
2245	8	9	9	2	5	7	7	5
2263	5	5	2	1	5	5	5	5
2264	9	9	6	7	8	8	7	3
2269	9	9	9	9	9	9	9	9
2286	7	7	8	8	6	3	7	5
2287	9	9	9	7	2	5	5	1
2288	5	3	4	5	1	1	1	2
2290	7	7	5	7	7	8	7	6

2293	5	4	8	9	9	7	9	9
2295	9	9	5	5	5	5	7	7
2321	9	7	8	8	9	7	8	7
2331	6	7	4	3	7	4	3	6
2333	7	3	7	7	5	6	6	7
2343	8	5	7	5	6	5	4	3
2375	8	7	7	6	6	5	1	5
2379	8	6	5	6	5	3	6	2
2380	9	9	9	9	9	3	2	2
2427	9	9	9	5	9	9	9	5
2433	8	7	2	2	2	3	1	1
2451	9	7	8	8	9	8	3	5
2453	8	3	9	4	8	8	5	7
2471	6	7	8	7	3	3	6	4
2492	8	8	8	6	6	6	3	3
2503	7	9	9	5	6	8	8	5
2505	9	9	8	7	6	9	9	7
2554	7	2	5	6	2	6	5	5
2556	9	9	9	5	5	9	5	7
2559	8	7	8	5	8	3	5	5
2560	5	5	6	5	6	6	4	2
2563	5	9	7	3	6	9	2	1
2566	8	8	8	7	7	7	5	6
2571	9	5	9	5	5	8	5	1
2595	7	3	7	6	3	2	1	1
2599	6	7	5	7	7	4	4	3
2600	7	5	8	3	5	5	2	1
2602	6	7	3	1	6	1	2	1
2616	8	8	8	8	8	8	5	7
2621	7	6	7	7	7	5	5	5
2624	5	5	5	5	5	5	5	5
2625	7	6	6	3	4	2	3	1
2626	8	8	7	4	7	7	4	3
2639	8	8	7	5	4	7	6	1
2649	8	8	8	3	8	4	9	7
2671	5	8	7	2	4	6	9	4
2672	7	8	7	5	7	7	7	5
2673	9	6	8	6	6	8	5	5
2675	8	7	7	6	5	5	6	4
2680	7	7	7	6	6	8	6	6
2681	8	8	8	6	7	7	5	5
2683	5	9	7	8	8	5	5	5
2686	7	7	7	3	6	6	3	2
2708	9	7	9	1	1	1	1	1
2720	8	9	8	7	8	7	7	8
2724	7	8	8	3	4	7	5	5
2725	7	9	6	1	5	1	5	1
2727	7	7	8	1	8	7	8	5
2738	7	4	6	2	5	4	3	5
2740	7	7	5	5	7	5	2	3

2744	7	7	7	5	9	7	7	5
2745	7	6	6	5	5	6	4	4
2766	7	7	8	6	8	7	6	6
2770	5	7	8	7	7	6	2	1
2771	9	7	6	2	2	5	5	3
2773	6	4	3	6	2	5	4	2
2791	7	4	2	5	7	1	1	1
2793	6	7	7	4	5	5	3	3
2795	8	5	8	7	5	7	8	5
2796	1	5	3	1	4	1	1	1
2806	8	9	7	3	9	9	5	2
2807	4	4	6	2	4	2	3	1
2814	5	3	1	1	2	1	1	1
2834	7	6	6	6	8	8	7	8
2836	5	6	6	3	5	5	5	5
2849	8	7	6	6	6	6	4	3
2853	9	8	8	9	7	8	9	9
2857	9	4	8	6	7	8	7	6
2864	8	7	7	1	5	6	5	2
2873	3	8	3	6	6	4	7	2
2886	8	8	8	5	8	7	5	5
2908	6	3	6	2	5	1	1	1
2913	3	4	1	1	2	1	1	1
2918	9	6	7	2	7	7	2	1
2921	6	7	8	7	6	7	5	7
2922	7	5	3	2	5	3	6	2
2939	7	6	9	5	4	9	8	4
2943	6	7	8	6	9	7	4	2
2960	8	9	9	3	7	2	4	3
2976	5	7	7	7	7	6	5	6
2978	9	8	9	5	7	8	5	5
3027	8	5	7	5	7	7	6	5
3033	8	8	8	8	6	9	6	4
3046	1	5	3	3	1	1	1	1
3064	8	5	5	5	5	5	1	5
3087	8	8	7	6	7	6	4	6
3088	8	8	8	7	8	7	5	5
3123	7	3	6	1	2	6	1	1
3129	6	3	5	2	2	2	2	2
3130	6	7	5	6	5	6	9	7
3133	8	8	9	3	3	9	1	1
3146	8	6	3	2	3	7	4	3
3163	8	8	8	1	3	6	1	1
3181	7	6	6	2	8	2	6	2
3193	9	9	7	3	7	7	4	2
3203	7	6	3	1	2	2	5	1
3208	5	6	6	2	4	3	2	1
3239	8	7	7	5	7	6	9	6
3241	6	6	6	5	6	3	5	1
3289	1	5	6	1	4	2	1	1

3292	6	7	6	5	6	7	3	3
2307	1	7	3	1	5	1	1	1
272	7	2	1	1	1	1	1	1
2178	5	6	7	1	7	6	5	1
1975	8	9	6	7	6	9	7	6
1350	7	8	6	5	6	3	3	2
1912	1	1	1	1	1	1	1	1
2633	1	9	8	5	3	7	1	5
2371	8	9	8	7	4	7	3	2
670	1	1	1	1	1	1	1	1
679	6	6	2	3	5	3	3	4
1756	9	9	5	5	8	5	5	1
1856	8	9	9	9	9	9	7	9
395	9	7	9	5	7	9	5	5
3340	2	1	3	1	6	1	1	1
3305	6	6	5	3	3	3	1	2
2904	9	7	9	9	9	9	9	9
1177	6	6	6	5	5	1	5	1
2891	9	6	9	4	9	4	7	2
1732	5	6	1	1	1	1	1	1
3277	8	7	4	1	6	3	3	1
754	1	2	3	5	3	2	1	1
1218	9	7	7	5	5	5	5	5
2066	7	6	4	7	5	7	2	4
2588	7	6	5	1	6	1	1	1
1592	7	3	8	1	5	8	8	5
1229	7	5	7	7	7	6	1	5
1826	9	9	9	6	9	9	9	5
3051	5	5	5	1	5	5	5	1
2655	5	2	7	5	3	7	6	5
3202	9	7	7	6	4	6	7	3
2957	5	2	5	1	1	1	1	1
1240	8	7	7	1	5	8	1	6
112	3	9	8	6	3	2	2	2
3041	7	3	4	2	5	2	3	2
1442	9	6	7	1	2	5	1	5
2162	9	9	9	8	9	9	8	6
2468	5	6	4	3	2	1	1	1
564	7	7	7	2	5	5	5	5
1585	5	1	1	5	1	1	1	1
3334	7	6	7	6	6	7	7	7
2824	9	7	7	8	8	7	7	1
852	1	2	7	2	1	2	1	1
117	7	7	9	6	4	6	5	5
893	2	2	2	2	2	1	1	1
2539	2	3	2	2	2	2	3	2
1661	7	5	3	2	3	1	1	1
2061	1	5	8	1	4	2	2	1
2697	2	8	7	6	8	8	2	3
1361	8	7	7	8	5	7	8	6

256	4	5	1	6	3	3	1	3
1807	6	6	6	4	4	4	1	1
2734	4	2	1	1	1	1	1	1
1677	8	7	6	6	7	5	6	3
96	7	7	7	5	6	5	6	6
1905	8	7	7	2	6	6	2	1
1026	1	7	1	1	3	1	1	1
2986	6	6	8	7	5	3	5	2
2778	7	7	8	5	7	5	2	7
282	7	8	5	1	8	5	6	5
684	9	8	6	8	7	6	5	5
2937	9	9	6	1	6	7	4	1
952	6	5	8	6	5	6	1	5
927	7	1	1	4	1	1	2	1
590	1	3	7	5	4	3	4	2
827	5	9	9	9	9	9	6	7
1922	6	3	4	1	2	3	2	2
641	5	3	2	2	3	1	5	1
130	6	4	3	6	6	4	2	4
1671	9	9	9	9	9	9	9	5
1638	8	9	8	9	7	8	8	5
249	7	7	7	6	5	8	4	3
992	7	5	7	2	2	5	2	7
2187	1	3	2	1	5	6	1	1
2881	6	6	6	5	4	7	6	4
301	6	5	4	4	5	5	5	5
1188	6	7	4	2	3	3	1	3
1870	5	3	2	1	2	5	5	5
1569	7	5	6	2	1	1	1	1
84	3	6	5	6	6	6	6	5
833	1	1	1	1	1	1	1	1
2044	2	1	3	1	2	1	1	1
920	9	7	1	1	5	1	1	1
1890	4	7	8	8	6	7	6	8
895	7	8	3	2	7	1	1	2
2213	6	6	5	2	6	1	2	2
2490	6	8	3	5	5	3	4	2
2363	7	7	2	2	5	1	2	1
2975	5	5	5	5	5	5	5	5
324	6	8	5	5	8	6	6	6
1019	1	6	5	5	5	5	6	5
1895	7	5	7	3	6	9	8	5
2996	7	7	7	7	7	7	7	7
415	7	8	5	3	7	5	3	1
1541	8	7	8	8	6	7	6	7
1694	8	7	2	1	2	2	8	1
1234	4	7	8	5	2	3	6	4
182	8	8	9	8	7	8	6	1
1813	1	2	1	1	1	1	1	1
2260	2	4	6	4	4	5	1	2

122	7	8	5	6	7	5	7	5
944	4	6	7	1	5	5	1	1
3266	3	8	7	5	5	4	3	3
749	8	4	5	2	3	1	1	1
385	1	1	1	2	1	2	3	6
328	7	7	4	5	6	6	4	1
3214	7	8	7	3	5	7	6	2
608	1	1	1	1	1	1	9	1
2300	7	5	6	4	3	4	5	3
768	6	8	9	4	7	8	6	5
2142	7	8	8	8	9	4	8	2
1013	2	3	7	3	6	7	6	2
1201	6	7	5	4	3	2	2	2
422	7	4	5	4	4	4	2	2
1115	5	8	3	1	1	3	5	1
1436	9	9	9	9	9	9	9	9
208	7	6	6	6	8	7	6	7
2763	6	7	7	3	8	7	7	2
660	1	5	1	1	9	1	1	1
594	7	8	6	4	4	3	3	2
2691	2	3	2	1	1	1	5	2
1783	2	6	5	3	2	1	1	1
1339	6	7	7	3	6	6	3	2
2356	6	5	7	2	4	4	1	1
2594	6	7	6	5	8	4	2	2
1654	1	2	1	1	1	1	1	1
1333	9	4	2	3	8	2	6	6
2228	8	1	1	1	4	1	1	1
2193	7	7	8	1	5	3	5	7
1491	3	6	7	2	6	8	5	3
1949	6	1	2	1	2	2	1	1
3297	6	8	4	6	6	8	9	5
1980	7	8	8	2	9	8	2	2
2218	8	9	5	5	6	5	5	2
2397	9	5	7	6	4	6	5	6
2758	2	2	7	1	3	2	7	3
2484	7	6	7	6	5	5	2	2
2408	7	7	8	7	7	6	5	6
1272	8	5	4	6	5	4	2	2
1865	8	6	7	4	8	6	3	3
1458	7	8	6	3	6	6	5	4
2351	7	6	8	4	7	7	8	7
2533	4	6	9	1	2	6	4	1
1724	8	6	5	1	1	1	1	1
1927	9	9	9	1	9	1	1	1
1485	8	8	8	5	8	5	2	2
2609	9	7	7	9	9	9	1	7
1355	5	3	2	1	2	1	1	1
791	8	9	9	7	8	8	5	6
354	5	9	6	5	6	3	3	5

3109	5	6	6	3	4	4	3	3
2087	3	7	6	2	6	2	8	8
2783	8	5	6	1	1	5	1	1
287	5	5	5	5	5	5	5	5
648	7	5	9	7	6	7	1	5
1850	8	6	6	6	6	6	6	5
1860	7	7	2	1	8	2	5	2
3168	6	3	5	2	4	2	4	3
1327	5	8	9	4	1	1	5	1
244	7	7	8	5	6	2	2	4
193	7	8	7	3	6	7	5	1
166	5	7	2	2	5	5	6	2
1308	7	6	6	4	3	2	1	1
88	7	7	6	4	5	5	5	3
1687	6	5	7	3	3	5	1	1
1702	7	5	6	6	3	5	3	4
2282	6	6	3	3	6	3	3	1
846	7	7	8	7	7	7	7	6
2317	7	7	3	2	3	2	3	2
3178	7	8	7	5	4	8	7	7
101	1	6	9	1	1	9	5	1
2666	7	7	6	3	7	6	2	3
1390	7	7	2	3	5	1	6	1
1970	7	5	8	4	4	6	3	2
1682	9	9	7	9	8	1	9	3
2403	1	1	1	1	1	1	1	1
2157	6	5	6	3	7	3	5	6
2648	5	3	4	5	1	1	1	1
1476	9	6	8	5	7	6	4	5
2327	1	1	1	1	1	1	1	1
2841	3	7	4	6	9	2	3	4

PUB09_B	PUB10_B	PER01_B	PER02_B	PER03_B	PER04_B	PER05_B	PER06_B	PER07_B
4	4	1	4	5	2	6	2	1
9	7	7	2	4	1	6	2	1
6	6	2	4	2	1	3	2	1
1	1	1	1	1	1	1	1	1
3	7	3	6	6	3	3	3	6
6	7	2	1	2	2	1	2	3
5	8	3	6	2	3	3	5	2
7	9	1	2	2	1	3	1	1
2	4	1	2	1	1	1	1	1
1	2	1	1	1	1	1	1	1
8	7	5	2	2	1	2	1	1
5	9	2	1	1	1	1	1	1
7	7	1	7	4	3	6	4	5
9	9	1	6	1	1	7	1	1
6	2	2	3	3	1	3	2	1
3	4	2	1	1	1	4	2	2
1	5	9	8	9	3	8	7	1
6	9	5	6	1	2	3	1	1
7	8	3	3	5	1	4	4	1
2	7	2	1	1	1	1	1	1
7	7	3	4	3	2	5	2	1
5	5	2	2	2	1	3	1	1
1	4	5	3	1	1	1	1	1
6	6	3	3	5	3	5	3	2
6	8	3	3	1	1	6	2	1
3	4	1	1	1	1	5	1	1
7	7	3	3	3	1	3	2	1
1	1	1	1	1	1	1	1	1
1	1	1	9	5	1	1	1	1
1	1	5	6	1	1	1	1	1
8	8	1	1	1	2	5	5	1
6	5	2	3	3	4	1	4	1
6	6	3	1	2	1	6	2	1
9	8	1	2	2	1	7	2	1
6	6	2	6	4	1	3	3	2
6	5	5	9	6	4	6	4	2
7	5	2	2	2	2	2	2	2
1	5	9	9	1	1	5	1	1
7	7	2	1	1	1	3	1	1
1	7	3	5	5	2	4	2	2
1	3	9	9	5	1	9	1	1
2	4	1	2	1	1	1	1	1
1	1	2	2	2	1	1	1	1
4	4	1	3	3	1	2	2	2
1	1	1	1	1	1	1	1	1
2	2	3	2	1	1	1	1	1
6	9	1	4	5	1	7	1	1
5	5	1	4	3	2	4	2	1
7	3	6	6	2	2	8	6	4

5	7	2	1	2	1	2	1	1
9	9	1	5	5	5	5	5	5
7	5	1	2	1	1	5	1	1
1	2	6	6	6	2	7	2	1
1	1	1	1	1	1	1	1	1
1	3	5	3	3	1	2	1	1
3	8	1	5	6	1	3	1	1
2	3	1	1	4	1	1	1	1
5	3	5	7	7	2	2	5	2
2	1	1	6	6	1	1	1	1
3	5	5	5	5	5	5	5	5
1	1	1	1	1	1	1	1	2
3	3	2	2	2	2	3	2	1
6	6	1	3	3	1	4	2	1
5	8	1	7	2	1	9	1	1
2	6	3	1	1	1	3	1	1
8	5	2	5	5	3	5	3	2
3	6	2	3	2	1	2	1	1
9	5	1	3	6	2	5	3	3
4	7	1	1	3	1	3	3	1
1	1	1	4	2	1	4	1	1
8	9	1	1	1	1	1	1	1
3	3	1	1	1	1	1	1	1
4	6	1	3	3	2	5	2	1
2	6	8	8	4	1	8	5	5
4	5	1	1	1	1	1	1	1
7	9	2	2	1	1	1	1	1
7	9	3	1	1	1	1	3	1
6	6	2	4	5	4	4	5	2
8	7	5	6	4	3	4	3	3
3	7	2	2	3	2	1	2	1
2	3	1	4	2	2	6	3	3
5	6	2	5	4	5	7	3	3
7	6	3	3	3	3	3	3	3
7	6	4	6	6	1	2	2	1
3	8	2	5	2	5	3	2	2
6	6	1	1	1	1	1	1	1
6	8	3	1	5	2	2	2	1
1	1	1	1	1	1	1	1	1
7	7	1	1	1	1	1	1	1
4	6	1	1	2	1	2	2	1
5	5	2	3	3	2	3	3	1
4	6	1	2	2	4	3	1	1
8	8	6	6	6	4	5	6	3
3	5	3	3	3	3	5	2	2
7	7	8	8	9	3	2	7	5
2	4	4	6	3	2	2	2	2
3	4	4	7	6	2	4	2	2
8	8	1	8	5	3	5	2	5
8	6	4	5	3	5	4	1	1

7	7	1	3	3	2	1	1	1
1	1	5	1	1	1	3	1	1
3	2	1	5	1	1	5	1	1
5	6	4	3	5	2	5	6	1
4	3	1	1	1	1	1	1	1
7	8	1	1	1	1	4	1	1
6	8	2	2	2	1	2	1	1
8	7	3	4	2	3	4	2	1
7	6	1	3	1	1	2	1	1
5	4	5	3	2	5	2	4	1
5	8	5	2	5	5	7	5	2
7	4	2	4	5	2	6	2	3
1	1	4	3	2	2	2	2	1
4	8	3	7	2	1	3	2	1
6	8	1	2	2	1	1	1	1
7	8	2	2	5	2	5	2	1
1	5	6	6	1	1	2	1	1
7	5	4	7	3	3	3	4	1
1	1	1	1	1	1	1	1	1
7	7	1	3	5	3	3	1	1
1	3	1	3	1	3	6	1	1
1	2	3	3	1	2	1	1	1
5	8	2	3	6	1	1	2	1
2	6	1	3	1	1	1	1	1
5	9	1	3	2	2	2	1	1
5	3	2	2	1	1	1	1	1
6	8	2	2	7	1	3	3	2
1	7	1	1	1	1	7	1	1
3	6	1	2	7	1	5	6	1
9	7	5	5	5	6	2	3	2
2	2	1	1	2	1	1	1	1
6	5	3	3	3	2	6	2	2
1	1	1	1	1	1	1	1	1
6	9	4	5	2	3	3	2	1
4	4	1	1	3	1	2	3	2
7	9	1	4	2	1	5	1	1
4	7	2	2	3	1	1	1	1
7	6	3	2	3	2	2	2	2
4	5	2	3	4	1	2	2	1
6	4	1	1	1	1	1	1	1
7	5	1	3	1	1	5	3	1
1	2	2	2	1	1	1	1	1
7	8	1	4	4	2	3	5	1
7	6	5	1	2	1	3	3	1
3	6	1	9	5	2	4	5	3
3	3	1	1	1	1	1	1	1
1	4	1	3	1	1	5	1	1
7	8	3	9	5	3	7	2	1
5	8	3	2	2	1	2	1	1
8	9	1	4	3	2	4	2	2

6	7	1	1	7	1	1	2	1
6	6	1	1	1	1	1	1	1
1	1	1	1	1	1	1	1	1
6	8	5	3	6	1	3	3	1
7	7	1	5	5	2	1	1	1
1	1	2	2	2	1	1	1	1
4	7	3	2	1	2	3	1	1
6	7	2	1	2	2	2	2	1
1	2	2	1	1	1	1	1	1
6	5	1	1	5	5	2	5	1
6	6	2	4	2	3	5	3	3
6	6	3	3	3	2	3	3	2
4	5	5	3	1	1	1	1	3
6	6	2	1	3	1	6	1	1
5	5	2	2	3	1	1	2	1
7	9	4	7	4	3	8	4	1
7	6	1	1	3	1	6	1	1
1	2	2	9	2	1	1	1	1
3	3	1	6	6	3	7	6	2
1	3	1	1	1	1	1	1	1
8	7	1	1	1	1	1	1	1
5	6	3	5	2	2	4	2	1
2	6	1	2	2	1	3	1	1
4	6	2	1	1	1	1	1	1
6	6	1	8	5	5	4	5	1
2	4	1	6	2	1	5	1	1
8	7	2	3	2	2	1	1	1
7	9	6	7	1	2	6	2	3
8	7	1	3	2	1	3	1	1
1	5	6	4	8	5	4	6	1
6	6	1	3	1	1	1	1	1
5	6	2	2	2	1	1	2	1
6	9	1	5	3	1	5	2	1
5	5	1	7	1	2	7	2	8
7	7	1	2	1	1	1	1	1
5	7	2	4	5	3	8	5	1
4	8	1	4	6	1	6	2	1
5	7	1	5	3	1	3	1	1
7	8	2	2	4	1	3	1	1
1	5	3	6	6	1	7	7	1
5	5	2	5	1	3	1	2	1
6	5	1	3	1	1	1	1	1
2	3	4	3	2	1	2	1	1
1	5	1	1	1	1	1	2	1
9	9	1	1	2	1	2	1	1
3	5	1	3	1	1	5	1	1
7	8	3	8	3	6	7	5	3
6	4	2	6	3	3	8	2	2
5	7	4	1	5	1	5	1	1
8	7	2	5	4	1	2	1	1

9	9	9	1	9	5	9	9	5
4	7	1	1	4	3	5	3	1
1	5	5	8	1	1	8	1	1
7	6	5	5	4	2	4	5	2
1	9	1	9	1	7	8	1	7
1	3	3	2	1	1	2	2	1
9	7	3	7	6	3	8	5	2
7	7	4	4	1	5	5	5	1
6	5	2	7	5	5	6	3	1
4	6	2	1	1	1	1	1	1
8	7	1	5	3	3	7	4	2
4	3	1	6	2	5	6	1	1
8	8	3	5	5	1	2	1	1
5	7	1	4	3	1	6	1	1
1	2	1	1	1	1	3	1	1
9	9	3	3	1	3	3	3	6
4	4	1	1	1	1	2	1	1
5	5	1	1	1	1	1	1	1
1	1	1	1	1	1	1	1	1
6	6	2	6	6	1	8	6	1
6	6	3	1	2	2	1	1	1
7	5	1	7	1	1	1	1	1
5	7	6	9	5	7	9	7	5
8	6	3	7	3	1	3	1	1
7	7	1	5	5	1	6	5	1
2	5	7	4	3	1	3	3	1
9	9	1	8	5	5	7	5	5
8	9	2	3	3	2	1	1	1
8	6	1	2	1	1	6	1	1
7	7	2	5	5	3	7	8	1
6	7	2	3	2	3	6	2	1
7	6	1	4	3	2	3	1	1
7	9	1	2	2	1	1	1	1
9	9	1	5	1	1	4	1	1
1	5	1	6	1	1	1	1	1
2	6	3	2	3	1	2	1	2
7	6	1	3	2	2	5	2	1
2	2	2	2	1	1	2	2	1
7	3	5	1	6	1	7	3	1
9	8	4	2	2	4	2	2	1
5	5	1	5	5	1	7	3	1
8	8	2	2	2	2	2	2	2
1	3	2	7	2	1	3	2	2
6	8	1	6	5	7	6	5	5
5	6	2	7	3	3	2	2	1
8	6	1	2	2	1	9	1	1
5	6	1	2	3	1	5	1	1
1	1	1	5	1	1	5	1	1
2	2	3	3	1	1	2	1	1
7	9	6	1	1	1	7	3	1

6	5	7	1	1	1	7	2	1
4	7	1	2	3	1	3	1	1
9	8	6	5	8	6	5	8	5
1	3	1	1	1	1	1	1	1
7	8	1	2	1	1	5	1	1
6	4	2	2	1	1	3	1	1
3	3	1	1	1	1	1	1	1
8	8	2	4	6	1	3	3	7
6	4	6	5	2	2	5	1	7
7	7	2	4	3	3	2	3	2
5	3	1	3	1	1	1	1	1
5	7	1	2	1	1	2	1	1
6	8	6	7	8	3	3	5	1
6	8	6	3	2	1	1	1	1
3	3	5	5	3	1	1	3	5
8	8	2	2	3	1	2	1	1
6	7	2	2	6	5	3	5	3
1	5	1	5	1	3	3	1	1
5	5	4	5	2	5	6	3	4
6	7	1	1	1	1	1	1	1
3	5	1	1	1	1	1	1	1
7	6	1	1	1	1	1	1	1
3	5	1	6	6	1	2	5	1
5	7	1	1	2	2	1	5	2
1	1	2	1	1	1	1	1	1
1	3	3	1	2	1	6	1	1
9	9	1	5	2	3	3	2	1
3	6	1	2	1	1	3	1	1
5	8	3	4	5	2	2	3	1
1	6	1	1	1	1	1	1	1
5	5	2	5	5	3	7	6	1
6	7	1	1	7	1	6	2	1
4	5	1	5	1	2	5	1	5
1	6	1	1	1	2	3	1	1
8	5	4	5	5	5	5	6	5
5	5	1	2	6	2	7	5	5
1	5	1	1	4	1	6	1	1
4	8	6	7	2	2	7	2	2
3	2	2	6	1	1	2	2	1
6	7	1	4	3	2	3	2	1
7	8	2	3	1	2	1	1	1
2	6	9	1	7	2	8	6	9
4	7	1	3	2	1	5	1	1
1	2	1	4	1	1	3	1	1
8	8	1	2	2	2	2	1	1
8	7	5	6	6	5	3	2	1
3	6	1	3	1	1	3	1	1
3	4	2	3	4	2	3	1	2
3	6	7	5	5	5	5	6	5
1	4	4	2	2	2	2	2	1

2	4	5	2	1	1	5	1	1
7	8	1	1	1	1	2	1	1
8	7	1	3	1	1	1	1	1
4	3	1	4	5	4	7	1	1
6	7	3	7	5	3	5	6	1
7	9	1	2	5	1	2	3	1
2	8	3	4	3	1	2	1	1
1	2	1	1	1	1	1	1	1
5	9	2	2	1	1	2	1	1
5	7	1	3	1	4	3	1	1
5	7	6	6	2	2	4	3	5
5	8	1	2	1	2	1	1	1
6	4	3	7	6	2	4	5	1
7	9	1	9	6	5	5	2	1
3	4	3	6	3	3	3	2	2
5	6	1	1	1	1	1	1	1
8	8	6	2	3	1	5	4	2
6	6	1	6	2	1	5	1	1
2	5	8	5	1	1	5	1	5
6	6	2	2	2	1	3	1	1
4	8	2	2	1	1	2	1	1
3	7	1	3	6	4	2	4	1
7	7	3	2	1	1	1	1	1
1	4	1	6	2	1	2	1	1
2	7	2	8	9	7	2	2	1
5	7	1	5	2	1	5	2	1
5	8	2	7	8	5	7	6	1
4	5	1	1	1	1	1	1	1
7	9	1	1	1	1	1	1	1
9	5	1	1	1	1	1	1	1
2	5	1	1	2	2	1	2	1
1	4	5	8	5	5	6	5	5
3	7	1	4	5	1	5	3	1
5	5	4	2	1	1	1	1	1
7	7	2	2	5	1	6	1	1
7	8	1	2	3	1	2	1	1
3	6	6	3	2	2	7	1	1
8	8	1	7	1	1	1	1	1
5	9	4	9	9	5	9	5	5
7	9	1	7	1	1	6	1	1
6	4	1	3	2	2	2	1	1
7	8	1	2	2	1	2	1	1
6	6	1	1	1	1	2	1	1
3	9	5	6	3	1	4	1	1
8	9	4	2	1	1	1	1	1
4	6	5	2	7	1	2	2	1
5	7	2	5	4	2	4	3	1
3	3	5	5	4	4	4	4	5
7	8	3	6	5	3	6	5	5
6	7	2	1	5	3	5	1	1

6	7	1	1	1	1	1	1	1
7	6	6	4	5	2	7	5	2
7	8	1	6	6	2	2	2	2
1	8	1	6	1	3	1	1	1
4	6	1	4	2	5	4	4	2
5	6	1	2	2	1	1	1	1
8	9	1	5	3	1	1	1	1
2	2	1	2	1	1	1	1	1
8	7	3	4	3	2	1	3	1
5	8	1	1	1	1	1	1	1
6	7	1	1	6	1	1	6	6
6	6	2	1	1	1	3	2	1
6	7	2	3	2	1	6	1	1
7	8	2	6	6	1	2	3	1
6	7	1	5	5	1	1	1	1
6	5	1	2	1	1	7	1	1
1	4	5	2	1	1	1	1	1
5	5	1	1	1	1	1	1	1
9	8	5	6	6	4	4	6	1
5	6	1	4	5	2	6	4	1
6	5	3	4	3	2	6	3	1
9	9	1	4	2	2	3	3	1
1	3	4	4	4	3	4	3	3
1	6	1	7	1	1	5	1	1
5	8	1	4	2	5	4	1	1
7	7	3	5	2	1	1	1	1
1	2	5	2	1	1	3	1	1
2	2	1	4	4	1	3	2	1
6	7	1	5	1	8	5	2	1
9	9	1	1	1	1	2	1	1
3	3	2	2	3	5	2	4	1
6	3	4	3	3	2	3	3	1
8	8	1	1	1	1	1	1	1
5	5	2	6	5	2	2	2	1
1	7	3	7	7	4	6	1	1
6	6	2	1	2	2	1	1	1
5	6	4	3	1	4	4	1	1
6	5	2	1	3	2	3	1	1
7	7	2	3	1	1	3	1	1
8	6	2	8	1	1	2	2	1
1	2	2	1	3	1	3	1	1
2	5	1	2	1	1	2	1	1
7	3	1	1	1	1	1	1	9
3	7	6	4	1	1	2	1	1
6	6	1	6	6	3	6	6	1
6	7	2	2	2	4	4	3	1
5	2	1	3	2	1	5	2	1
7	7	3	3	7	1	7	2	1
6	5	1	1	2	1	1	1	1
7	8	3	4	6	1	2	1	1

5	6	2	7	6	2	3	2	1
5	5	5	5	5	5	2	5	9
1	1	1	1	1	1	7	1	1
6	4	1	1	2	1	6	4	1
9	9	2	3	2	1	1	1	1
5	7	1	2	1	1	1	1	1
2	6	1	8	2	7	3	6	1
1	8	3	9	7	1	8	4	1
5	5	1	1	1	1	2	1	2
3	4	3	5	5	2	2	2	1
2	5	2	1	1	1	5	1	1
5	9	1	9	7	1	3	6	1
4	7	2	3	3	3	4	3	2
6	6	1	1	1	1	1	1	1
7	6	1	2	1	1	2	1	1
1	7	1	6	1	1	3	1	1
4	5	3	5	6	1	2	2	1
3	6	1	4	1	2	3	1	1
8	6	1	5	2	1	4	1	1
1	1	1	1	1	1	1	1	1
5	8	1	1	3	1	5	1	1
1	4	1	3	1	1	3	1	1
5	7	1	1	1	1	1	3	1
5	7	2	2	1	1	2	1	9
6	8	2	7	3	1	7	1	1
5	7	1	1	2	1	1	1	1
9	8	1	1	2	1	1	1	1
2	7	1	5	1	5	1	1	1
6	8	1	3	2	1	2	2	2
6	7	2	4	3	1	1	1	1
8	7	1	2	1	5	3	5	1
6	5	1	2	3	1	1	1	1
1	1	1	5	1	1	1	1	1
1	6	5	7	5	3	7	5	3
5	8	1	6	1	1	5	1	1
8	7	9	8	7	6	2	8	4
2	5	1	2	2	5	2	3	1
8	8	2	7	2	1	3	5	1
3	6	5	2	2	1	6	2	1
5	4	1	7	3	1	3	1	1
2	6	1	1	1	1	1	1	1
8	8	3	2	2	1	4	4	1
7	7	1	1	6	1	2	5	2
5	5	5	5	5	2	5	5	5
3	8	4	3	1	1	1	1	1
9	9	1	2	1	1	1	1	1
6	7	3	2	5	4	3	2	1
7	7	1	5	5	3	7	1	1
2	1	5	1	2	2	1	1	1
8	8	3	4	4	3	2	2	5

8	7	1	5	1	1	1	1	1
1	9	1	7	1	5	4	1	1
6	6	9	5	6	7	9	5	5
3	8	2	8	6	3	7	2	1
7	7	3	3	2	2	3	2	1
9	9	1	4	2	3	2	1	1
5	6	3	8	5	2	5	5	6
2	6	7	1	1	2	1	1	2
9	9	1	1	9	1	6	1	1
6	9	1	3	1	1	3	1	1
2	5	1	2	2	2	1	2	1
7	9	9	9	8	7	8	7	8
9	8	1	2	4	1	6	2	1
4	7	2	6	6	4	3	2	1
5	5	1	3	2	1	1	1	1
7	6	2	3	2	1	1	2	1
3	9	2	1	1	5	2	1	1
5	7	3	5	1	4	6	2	1
7	6	2	5	1	5	5	5	1
5	4	1	1	1	1	8	1	1
7	6	1	2	2	2	1	1	1
8	9	5	3	2	1	5	3	1
7	6	6	4	7	2	7	2	1
5	5	2	1	1	1	1	1	1
4	2	1	1	4	1	4	1	1
3	7	2	4	2	3	3	2	1
6	4	2	2	2	1	2	2	1
3	4	1	7	1	1	8	1	1
7	7	1	4	3	2	2	2	1
5	5	1	1	5	1	1	1	1
5	5	1	8	5	1	3	1	1
2	4	2	3	6	3	2	2	1
7	6	6	4	2	1	4	1	1
6	8	1	2	2	1	2	1	1
5	7	5	2	3	3	5	3	1
7	8	1	1	1	1	1	1	1
7	7	1	7	1	1	1	2	1
6	6	3	1	1	1	3	1	2
4	7	1	6	5	5	5	5	5
7	8	5	7	5	5	7	5	5
6	8	2	5	3	3	4	1	1
6	8	1	7	1	6	6	1	1
3	3	2	5	3	3	5	3	1
1	6	1	1	1	1	1	1	1
9	9	3	2	4	3	5	3	1
7	8	1	3	5	1	3	2	1
4	1	1	1	1	1	5	1	1
8	8	3	6	6	6	9	9	5
3	6	2	1	1	2	2	1	1
4	4	1	3	1	1	1	1	1

7	7	2	3	2	3	5	3	4
6	4	2	3	1	1	1	1	1
6	8	1	1	1	2	2	1	1
3	7	2	3	2	1	2	1	1
3	5	2	3	7	1	7	3	1
3	7	1	3	2	1	1	1	1
2	3	3	2	1	2	4	2	1
4	5	3	6	4	3	6	4	2
7	7	1	3	3	1	1	1	1
1	3	1	5	2	1	4	1	1
9	8	5	2	7	1	3	5	1
6	6	1	5	4	2	4	1	1
1	1	2	1	3	1	3	1	1
9	9	1	2	2	1	1	1	1
5	6	2	5	5	3	2	5	5
5	5	3	2	2	1	3	1	1
7	6	4	1	1	2	2	1	1
7	8	2	6	3	3	3	3	2
2	5	1	1	3	1	5	1	1
3	8	1	7	2	2	7	2	2
7	7	2	1	1	1	1	1	1
1	3	4	4	4	2	6	5	1
1	1	1	1	1	1	3	1	1
7	5	4	6	6	1	6	1	1
7	9	2	5	4	5	1	1	1
2	3	2	2	2	2	2	2	2
8	8	5	5	2	2	3	1	1
7	7	3	2	2	3	1	1	1
5	7	2	4	4	1	5	1	1
5	6	1	3	2	1	2	3	1
3	2	1	1	1	1	1	1	1
6	6	2	2	2	2	5	4	1
8	7	2	2	6	2	3	6	2
1	1	1	1	1	3	2	1	1
4	6	5	3	3	1	4	3	1
7	5	2	2	1	1	7	2	1
6	6	2	2	1	1	4	1	1
2	4	1	3	2	3	1	2	1
5	4	2	3	3	2	7	2	2
6	8	2	2	2	1	2	1	1
7	1	3	3	3	1	4	6	1
3	6	2	7	1	1	3	5	2
1	4	1	7	1	1	3	1	1
3	7	1	1	1	1	1	1	1
6	8	1	1	1	1	1	1	1
5	6	2	2	2	1	3	2	1
2	6	2	7	5	1	3	1	1
3	6	3	2	1	1	2	2	3
5	6	3	5	4	3	5	2	2
1	4	2	4	3	2	4	2	1

6	6	2	5	4	5	3	5	1
2	1	1	2	1	1	2	1	1
1	1	4	1	1	1	1	1	1
5	2	1	2	2	1	1	2	1
7	9	2	5	2	1	1	3	1
6	6	1	2	1	1	2	1	1
2	2	1	1	1	1	1	1	1
7	7	1	7	5	5	2	5	5
5	7	1	4	2	1	1	1	1
1	1	1	1	1	1	1	1	1
7	6	3	5	2	2	5	3	1
7	9	1	2	1	1	1	1	1
9	9	1	1	1	2	1	1	1
7	9	7	1	5	1	6	2	1
1	2	2	1	1	1	2	1	1
7	6	1	3	5	3	2	2	1
9	9	1	1	1	1	1	1	1
5	7	6	6	5	1	5	1	5
9	9	1	1	6	1	4	1	1
2	1	2	2	1	1	1	1	1
2	3	1	1	1	1	2	2	1
2	2	1	3	2	2	2	2	1
6	6	1	1	1	1	1	1	1
7	8	7	7	6	5	6	5	1
1	6	2	3	1	1	6	1	1
7	9	1	3	2	1	1	3	1
8	6	5	2	6	1	4	1	1
9	6	5	3	5	2	7	5	2
1	7	1	1	5	1	1	5	1
6	8	1	7	5	1	5	1	1
7	8	4	5	3	2	1	1	1
1	2	1	1	1	1	1	1	1
9	6	1	3	6	1	1	1	1
7	3	7	9	8	7	7	3	3
2	6	6	1	5	1	6	1	1
5	5	1	5	5	5	5	5	1
5	5	1	1	5	2	5	1	1
2	4	4	3	4	2	3	1	1
5	6	1	3	1	1	1	1	1
1	5	1	1	1	1	1	1	1
8	7	2	1	3	4	5	2	1
1	7	6	2	1	1	5	5	3
2	3	2	4	3	1	1	2	1
8	7	3	4	2	5	5	1	1
1	8	1	1	1	1	1	1	1
2	3	1	2	1	1	2	1	1
3	3	6	2	1	1	2	1	1
6	4	1	6	8	2	3	6	1
8	8	2	5	4	3	3	2	1
8	6	2	1	2	1	1	1	1

1	6	6	5	1	6	2	1	1
1	2	7	1	6	5	8	1	1
1	1	1	7	1	1	1	1	1
3	5	2	3	2	1	4	1	1
5	7	1	1	5	2	2	1	1
6	5	1	1	1	1	1	1	1
1	2	1	3	1	1	2	1	1
6	5	3	1	1	1	3	1	1
5	6	2	2	1	1	1	1	1
5	9	5	9	1	1	7	1	1
9	8	2	4	2	2	3	1	1
8	4	4	6	2	1	8	6	8
5	5	1	1	1	2	5	1	1
1	1	1	1	1	2	3	1	1
4	3	1	4	7	2	4	3	3
5	9	1	5	3	3	5	1	5
6	2	5	3	4	2	4	5	2
5	5	5	3	5	2	3	5	5
4	4	1	2	1	1	2	1	1
8	8	1	1	2	1	2	1	1
8	6	5	5	5	5	7	5	5
7	5	3	8	8	6	2	6	5
4	7	1	5	6	1	1	3	1
1	4	1	3	1	2	3	2	1
6	6	2	3	3	3	6	3	3
5	5	3	3	5	3	5	4	3
2	2	1	3	1	1	3	1	1
5	5	5	5	7	5	5	5	5
5	1	6	5	3	2	1	2	1
5	5	1	1	1	2	1	1	1
1	1	5	1	1	1	1	1	1
5	1	1	1	3	1	2	1	1
1	1	1	5	1	1	1	1	1
8	8	1	1	3	1	2	1	1
2	5	5	7	3	2	7	3	1
2	7	6	5	2	2	5	1	1
2	4	7	8	4	3	5	3	2
1	5	1	2	1	1	5	1	1
5	5	1	1	1	1	1	1	1
8	8	1	6	5	1	5	2	1
4	4	5	6	6	5	6	6	6
9	8	1	3	6	1	7	2	1
7	7	5	1	5	1	1	1	1
4	5	1	4	3	2	2	1	1
8	8	8	7	7	7	6	6	6
1	1	1	8	2	1	1	2	8
7	6	1	2	1	1	2	1	1
7	5	4	4	1	2	4	1	1
1	1	2	2	2	2	2	2	2
2	4	1	5	5	5	5	5	1

5	6	7	7	5	8	9	5	7
6	5	1	2	2	1	2	1	1
6	4	2	4	2	1	2	2	1
1	3	1	1	1	1	2	1	1
7	5	1	1	5	1	1	1	5
2	3	1	2	2	1	2	2	1
8	6	2	3	6	2	6	3	1
1	1	1	9	9	1	1	1	1
4	4	1	6	5	3	3	4	1
7	6	5	1	3	1	2	1	1
8	8	2	1	2	1	3	2	1
7	7	3	2	1	1	6	2	1
2	2	3	5	2	2	2	2	2
4	4	1	1	2	1	2	2	1
5	5	5	4	5	1	3	5	2
9	9	1	1	1	2	2	1	1
6	8	3	2	1	1	2	1	1
8	6	3	2	4	1	2	2	2
1	6	1	4	1	1	9	1	1
3	2	1	3	1	1	2	2	1
1	1	1	5	5	1	1	1	5
2	1	3	7	2	2	3	1	1
5	6	3	3	4	2	5	1	1
3	6	2	3	5	1	3	2	1
4	8	1	6	3	1	2	1	1
1	1	1	1	1	1	1	1	1
6	9	1	1	1	1	3	1	1
1	1	1	1	1	1	3	1	1
9	9	2	2	2	1	1	1	1
7	8	5	3	2	1	2	5	1
1	2	6	1	1	1	1	1	1
7	9	5	6	6	5	3	2	1
7	9	2	7	2	1	2	2	1
7	5	2	9	8	5	5	6	2
7	8	2	1	2	1	1	1	1
5	2	2	4	7	2	4	2	7
3	3	5	3	2	2	6	1	1
7	6	3	4	3	3	5	2	2
5	7	6	6	4	2	5	5	5
3	3	7	8	8	2	8	8	4
5	4	3	5	3	1	4	2	1
7	6	3	6	7	2	4	3	1
6	6	1	1	1	1	5	1	1
1	1	7	5	1	1	1	1	1
1	1	1	9	1	1	9	1	1
2	6	7	8	8	2	7	2	1
7	7	3	2	3	1	7	2	1
1	1	1	1	1	1	5	1	1
5	5	1	3	2	2	2	1	1
5	8	8	1	5	1	1	5	3

6	5	1	1	3	1	3	1	1
8	9	2	6	2	1	2	3	1
7	8	1	1	6	1	5	5	1
5	5	1	1	1	1	1	1	1
9	7	4	2	3	2	1	4	1
5	7	1	3	2	2	5	2	1
1	1	8	6	1	1	1	1	2
6	6	2	1	1	1	2	1	1
1	1	1	5	7	1	1	1	1
5	8	3	3	2	1	1	1	1
7	6	2	1	3	1	1	2	1
4	8	1	6	1	1	7	3	1
8	5	1	1	1	1	1	1	1
4	5	3	2	2	1	1	1	1
3	3	5	1	4	1	1	1	1
6	5	2	4	5	4	2	1	1
2	3	1	6	2	2	1	2	5
7	7	3	5	3	2	2	2	1
3	4	2	3	2	1	3	2	1
9	8	5	7	7	3	5	8	2
9	6	1	5	5	1	1	9	5
5	7	3	3	6	2	3	2	1
5	6	1	1	1	1	1	1	1
4	3	4	3	2	3	7	2	2
7	8	1	1	5	5	3	5	1
1	1	1	1	1	1	1	1	1
6	4	1	1	1	1	1	1	1
1	1	1	1	1	1	1	1	1
9	7	2	1	3	1	4	1	1
1	1	1	1	1	1	1	1	1
3	7	2	8	3	6	9	2	3

PER08_B	PER09_B	PER10_B	SELB01_B	SELB02_B	SELB03_B	SELB04_B	SELB05_B	SELB06_B
1	1	1	1	1	1	1	1	1
1	1	6	6	1	1	1	6	8
2	2	2	8	1	7	1	1	1
1	1	1	3	1	1	1	3	2
3	3	6	4	4	3	2	6	3
1	1	3	5	1	1	4	5	1
2	2	4	5	1	1	1	2	2
1	2	6	5	1	2	1	5	1
1	1	1	2	1	1	1	2	1
1	1	1	3	1	1	1	1	1
1	1	1	3	2	1	1	1	1
1	1	1	1	1	1	1	1	1
3	1	5	8	7	1	1	3	6
1	1	7	7	1	1	4	8	6
1	2	5	1	1	1	1	1	1
1	1	2	3	2	1	1	1	1
1	1	3	1	6	4	1	3	5
3	2	7	3	1	1	2	6	6
2	3	4	3	2	5	1	6	3
1	1	1	1	1	1	1	1	1
2	3	3	2	1	1	1	2	2
1	1	2	5	1	1	1	1	1
1	1	6	5	2	1	1	3	1
2	2	3	7	5	1	3	5	6
1	1	5	7	1	1	7	7	1
1	1	1	4	1	1	1	1	1
1	3	3	3	1	1	3	5	1
1	1	1	1	1	1	1	1	1
1	1	1	5	1	1	1	1	1
1	1	1	1	1	1	1	1	1
1	1	6	8	1	1	2	7	9
2	3	6	1	1	2	2	3	3
1	2	1	7	1	1	2	3	1
1	2	2	9	8	1	4	3	1
1	2	3	6	1	6	3	6	5
1	5	9	7	6	1	1	2	1
2	3	2	1	1	7	1	1	1
1	1	1	9	1	1	1	8	1
1	1	2	3	1	1	2	2	1
1	1	3	3	2	1	1	1	1
2	1	5	5	1	1	2	2	1
1	1	1	1	1	1	1	2	1
1	1	2	1	1	1	1	1	1
2	1	1	7	1	1	1	1	1
1	1	1	1	2	1	1	1	1
1	1	1	6	1	1	1	3	1
1	1	6	5	1	1	1	6	1
1	2	2	5	1	1	1	2	1
1	7	5	8	1	8	1	6	5

1	4	3	7	6	2	1	3	1
5	5	2	5	1	1	5	3	1
1	2	4	5	3	1	1	1	1
1	1	5	9	1	5	6	6	8
1	1	1	1	9	9	9	9	9
1	1	2	6	1	1	1	2	1
1	1	6	6	1	1	1	7	1
1	1	1	1	6	1	1	1	1
5	5	7	3	2	1	1	2	1
1	2	1	7	7	1	1	5	3
5	5	5	5	7	3	2	4	2
1	1	1	5	1	1	1	3	1
1	1	2	2	1	1	1	3	1
1	1	1	4	1	1	1	2	1
1	6	8	9	6	6	1	3	8
1	1	2	3	1	1	1	2	1
2	2	3	2	1	1	1	1	1
1	1	2	7	2	1	1	7	2
2	1	2	1	4	1	1	3	3
1	1	3	1	1	1	1	3	1
1	1	1	2	1	1	1	1	1
1	1	1	1	1	1	1	1	1
1	1	1	1	1	1	1	1	1
1	1	3	3	1	1	1	3	1
5	4	2	8	5	2	1	5	4
1	1	1	1	1	1	1	1	1
1	1	1	5	1	1	1	1	1
3	3	1	7	7	1	1	3	1
1	4	5	2	1	1	1	1	1
2	4	5	4	3	2	2	5	2
1	2	1	1	1	1	2	1	1
1	3	3	7	1	3	2	4	1
3	2	2	5	1	1	1	3	1
3	3	3	6	1	1	1	1	1
1	4	5	3	1	1	1	4	1
1	2	3	2	1	1	2	1	1
1	1	1	1	1	1	1	1	1
1	2	1	3	3	2	2	2	2
1	1	1	1	1	1	1	1	1
1	1	1	1	1	1	1	1	1
1	1	3	3	1	1	1	3	3
1	2	3	3	1	1	1	3	1
1	1	1	2	1	4	2	1	1
3	4	4	2	1	1	1	1	1
2	2	5	3	3	3	2	5	5
2	8	7	7	1	7	7	7	7
2	2	2	6	3	3	2	6	2
1	2	2	8	1	1	2	3	1
3	3	6	6	6	7	2	7	8
1	4	4	7	5	1	2	7	6

1	1	3	1	1	1	2	2	1
1	1	1	1	1	1	1	1	1
1	1	1	5	1	1	1	1	1
1	2	6	6	2	1	2	3	1
1	1	1	1	1	1	1	1	1
1	1	3	2	1	1	1	2	1
1	2	2	5	1	1	1	1	1
1	2	2	1	5	1	1	1	2
1	1	2	2	1	1	1	3	1
2	5	3	1	1	5	3	2	1
5	5	4	6	1	5	1	2	6
1	3	4	3	1	2	1	3	1
1	1	1	3	1	1	1	3	1
1	5	2	2	1	2	3	2	1
1	4	2	2	1	1	1	1	1
1	5	2	5	1	2	1	1	1
1	1	2	9	1	1	1	2	1
2	3	3	7	1	2	4	1	3
1	1	1	1	1	1	1	1	1
1	2	2	5	1	1	2	3	1
1	1	1	8	1	1	1	2	1
1	1	2	2	1	1	1	2	1
1	1	6	1	1	2	1	3	1
1	1	3	1	1	1	1	2	1
1	1	2	3	1	3	1	5	1
1	2	2	2	1	1	1	7	6
1	2	5	1	1	1	1	2	1
1	1	7	2	1	1	1	2	1
1	3	4	6	1	1	1	1	1
2	2	3	7	3	2	2	5	5
1	1	1	1	1	1	1	1	1
2	3	3	3	1	1	1	3	1
1	1	1	1	1	1	1	1	1
2	1	5	1	1	1	1	7	1
1	1	2	2	1	1	1	1	1
1	1	3	2	1	1	1	8	9
1	1	2	1	1	1	1	1	1
2	3	2	2	2	6	3	2	1
1	1	2	3	1	1	1	1	1
1	1	1	3	1	1	1	6	1
1	1	1	1	1	1	3	3	6
1	1	2	1	1	1	1	1	1
1	2	5	1	1	6	7	8	2
1	2	2	3	5	3	1	2	6
5	4	8	8	6	6	4	8	7
1	1	1	1	1	1	1	1	1
1	1	1	2	1	1	1	1	1
4	6	8	7	1	1	2	8	6
1	1	2	2	1	1	1	1	1
2	2	3	8	1	1	3	7	2

1	1	1	3	1	1	1	1	1
1	1	1	1	2	1	2	1	1
1	1	1	1	1	1	1	1	1
1	2	6	8	1	6	1	4	6
1	2	2	5	1	1	1	6	1
1	1	1	2	1	1	1	1	1
1	1	2	1	1	1	1	1	1
1	1	2	2	1	1	1	1	1
1	1	2	1	1	1	1	1	1
1	5	2	1	1	1	1	1	1
1	2	3	3	2	4	4	6	1
3	3	3	6	4	2	6	7	6
1	2	6	1	1	1	1	6	1
1	3	3	6	1	1	1	1	1
1	1	1	1	1	1	1	1	1
3	2	4	3	5	2	3	6	6
1	3	1	3	1	1	1	1	1
1	1	2	6	1	1	1	1	1
5	5	6	8	2	2	1	6	1
1	1	1	5	3	6	5	4	7
1	2	2	3	1	2	1	1	1
1	2	2	6	1	1	6	6	6
1	2	3	6	4	1	1	3	5
1	1	1	1	1	1	1	2	1
1	1	1	8	5	6	1	1	6
1	1	3	5	1	1	1	1	1
1	1	2	2	2	1	1	3	2
1	2	3	2	2	2	2	6	4
1	1	2	3	1	1	6	7	3
1	6	8	5	1	3	6	6	6
1	1	1	2	1	1	1	1	1
1	1	2	7	2	2	2	6	4
1	5	6	3	1	2	2	6	2
2	2	2	7	2	1	1	2	1
1	1	2	2	1	2	1	7	1
1	4	6	7	1	4	3	6	1
1	1	3	3	1	1	1	3	6
1	1	1	6	2	1	1	1	1
2	3	6	4	1	3	1	3	1
2	2	6	5	1	1	1	1	1
1	1	4	2	1	1	3	4	1
1	1	1	3	1	1	1	1	1
1	2	3	7	2	3	8	5	6
1	1	1	1	3	1	1	1	1
1	2	1	9	1	2	1	5	1
1	1	5	7	2	1	1	1	1
5	2	3	7	1	1	7	7	1
3	4	6	8	2	7	2	6	6
1	5	1	3	1	1	1	1	1
1	3	4	6	3	4	2	1	4

5	5	5	9	6	9	6	6	9
2	2	3	7	3	1	1	2	4
1	1	3	8	1	1	1	1	1
2	5	2	3	1	1	6	1	2
1	1	5	7	6	1	1	7	1
1	1	1	4	2	4	1	5	4
1	2	2	7	1	4	2	2	2
1	1	1	6	1	1	1	2	1
2	2	7	8	7	8	6	8	5
1	1	1	1	1	1	1	1	1
1	6	6	6	3	2	2	6	1
1	2	3	5	1	1	3	2	1
1	2	5	1	1	3	2	8	1
2	3	4	8	1	3	4	6	9
1	1	2	2	1	1	1	2	1
1	1	6	5	1	1	1	5	5
1	1	1	1	1	1	1	2	1
1	1	1	3	1	1	1	5	1
1	1	1	1	1	1	1	2	1
1	3	6	8	1	5	5	1	1
1	3	1	1	1	1	1	1	1
1	1	1	8	1	1	3	5	1
1	6	7	9	5	1	6	7	3
1	1	4	1	1	1	1	2	1
1	1	1	2	2	1	1	1	6
1	2	6	4	1	2	1	4	1
1	3	7	5	1	1	1	9	1
1	2	6	4	2	2	2	6	8
1	5	2	6	1	1	1	1	1
1	4	6	3	1	5	5	5	1
1	1	5	6	1	3	3	2	1
1	3	3	3	1	1	1	1	1
1	1	3	2	1	1	1	1	1
1	1	1	5	1	3	6	6	6
1	1	4	5	1	1	1	5	6
1	2	3	2	1	1	1	2	1
1	2	4	4	1	1	2	1	1
1	2	2	5	5	2	2	2	1
1	5	7	3	1	4	1	2	1
1	2	2	8	5	2	2	6	7
5	4	6	7	1	3	1	7	5
2	2	2	1	1	1	1	1	1
1	1	5	4	1	1	1	1	1
5	5	7	4	1	1	1	1	1
1	2	6	7	1	1	1	7	1
1	1	2	2	1	2	2	4	1
1	1	1	2	2	1	1	3	1
1	1	1	1	1	1	1	1	1
1	1	1	3	1	1	1	1	1
1	5	5	5	1	1	1	2	1

1	2	4	6	1	1	1	1	1
1	1	2	3	1	1	2	5	1
5	8	5	7	5	5	5	5	5
1	1	1	1	1	1	1	1	1
1	1	1	2	1	1	1	1	1
1	2	3	1	1	1	1	6	1
1	1	1	1	1	1	1	2	1
1	3	8	2	8	4	1	9	2
1	1	1	1	8	1	1	1	1
2	3	4	3	2	2	3	4	2
1	1	1	1	1	1	1	1	1
1	1	2	2	1	1	1	1	1
1	1	7	7	1	1	1	3	1
2	1	2	5	1	1	1	7	2
1	3	3	9	9	1	3	3	9
1	2	3	6	2	1	2	1	1
3	3	2	1	5	1	1	1	1
1	1	3	1	1	1	5	5	1
2	4	5	4	5	1	1	2	4
1	1	1	1	1	1	1	1	1
1	6	7	1	1	1	1	1	1
1	1	1	8	5	1	5	7	5
3	2	3	3	2	2	4	4	4
5	5	1	1	1	1	1	1	1
1	1	1	2	5	1	1	1	1
1	1	1	7	1	1	1	6	1
1	5	5	1	1	7	6	7	1
1	1	2	6	1	3	1	7	3
1	5	3	1	1	2	1	1	1
1	1	7	6	7	1	1	4	6
3	3	5	7	1	3	7	8	8
1	5	6	7	3	6	3	6	8
1	1	5	2	5	5	5	6	5
1	1	5	5	1	1	1	5	1
5	6	5	4	5	5	5	6	6
1	1	3	7	1	1	6	6	6
1	1	5	5	1	1	1	5	1
6	3	7	7	3	1	2	8	2
1	1	2	2	1	1	2	2	1
1	2	2	8	1	1	1	1	1
1	1	2	3	3	3	5	7	3
1	1	2	7	9	1	1	1	1
1	1	4	7	1	3	2	8	4
1	1	1	5	1	1	1	1	1
1	2	2	1	1	2	2	2	1
1	5	5	2	1	1	5	7	1
1	1	3	5	3	1	1	6	1
1	1	1	3	4	1	1	1	1
2	2	4	5	1	2	6	6	6
3	3	2	6	1	2	2	7	4

1	1	2	4	1	1	1	1	1
1	1	2	7	1	1	1	1	1
1	1	1	3	5	6	5	4	1
1	4	2	8	1	3	3	6	6
1	3	7	5	1	1	3	7	1
3	3	4	1	1	1	1	3	3
1	1	4	2	1	1	1	2	1
1	1	1	5	5	1	1	1	1
1	2	3	1	1	1	1	6	3
1	1	3	6	1	1	7	7	1
3	3	3	5	1	1	1	6	1
1	1	3	1	1	1	1	2	1
2	3	2	3	7	1	2	4	7
1	5	7	7	1	5	2	7	1
2	1	5	5	2	6	5	6	3
1	1	1	1	1	1	1	1	1
7	4	4	8	3	6	2	3	9
1	1	3	6	1	1	1	1	1
1	1	5	2	5	5	7	6	5
5	2	2	9	1	1	1	1	5
1	2	2	2	1	1	1	2	1
1	3	5	3	1	2	2	1	1
1	1	2	1	1	1	1	2	1
1	1	5	7	2	2	1	1	1
1	1	8	5	1	6	5	7	1
1	1	2	4	1	1	1	2	1
2	2	2	7	1	2	6	4	6
1	1	1	6	1	1	1	3	1
1	1	1	1	1	1	1	2	1
1	1	1	1	1	1	1	1	1
1	1	1	1	1	1	1	1	1
5	5	7	6	1	1	1	7	1
1	2	5	2	1	1	1	4	1
1	1	1	1	1	1	1	1	1
1	3	4	6	1	6	1	6	2
1	1	1	1	1	1	1	1	1
1	1	3	7	3	3	2	1	1
1	1	3	1	1	1	1	2	1
5	5	9	9	5	5	5	9	5
1	1	7	7	5	1	1	9	1
1	1	1	1	1	1	1	1	1
1	1	2	6	2	1	2	4	1
1	2	3	6	1	2	1	3	1
1	2	4	3	1	1	1	3	1
1	2	4	4	1	1	1	2	1
2	1	5	7	1	1	6	5	1
1	1	4	5	1	1	1	4	6
6	5	6	3	3	6	3	6	6
3	4	7	7	5	1	1	7	1
1	2	2	8	1	1	6	6	5

1	1	1	1	1	1	1	5	1
2	5	6	4	2	2	2	4	6
2	2	6	2	1	1	1	1	1
1	1	7	8	9	1	9	7	9
3	3	6	6	6	5	1	7	1
1	1	2	5	1	1	1	1	1
1	2	3	7	1	1	1	1	5
1	1	1	1	1	1	1	1	1
1	2	5	2	1	2	3	4	1
1	1	4	1	1	1	1	2	1
1	3	1	3	1	1	1	1	1
1	1	1	5	1	1	1	1	5
1	2	4	3	1	1	1	7	1
1	3	6	4	1	2	1	2	1
1	1	5	1	1	1	6	7	1
1	1	2	6	1	1	1	6	1
1	1	1	2	6	1	1	4	1
1	1	1	6	6	1	1	1	1
3	3	7	5	1	1	6	7	3
1	2	6	8	3	1	6	8	5
1	2	6	9	3	1	1	7	3
1	2	3	6	1	6	4	6	4
2	2	3	4	4	4	1	4	7
1	1	1	7	1	1	3	3	1
1	3	5	4	1	1	2	3	2
1	1	1	1	1	1	1	2	4
1	1	1	2	1	1	1	1	1
1	3	2	2	5	6	2	2	3
1	1	3	5	1	1	1	1	1
1	1	1	5	3	1	1	1	1
3	1	5	2	1	1	1	1	1
2	3	2	9	5	2	5	7	6
1	1	1	1	1	1	1	1	1
1	1	1	5	1	1	1	1	1
1	1	7	9	9	1	4	4	8
1	2	2	6	2	2	1	1	1
1	1	5	2	1	1	2	4	1
1	1	1	1	2	1	1	1	1
1	2	2	3	1	1	1	2	1
1	2	3	2	1	1	1	3	1
1	1	2	3	1	1	1	1	1
1	1	2	2	1	1	1	6	1
3	4	3	1	1	1	1	6	6
1	1	2	1	1	1	1	2	1
2	1	5	6	1	1	1	4	1
1	2	2	7	5	3	1	2	8
1	2	3	5	5	2	1	1	1
1	6	5	7	3	5	2	2	6
1	1	1	1	1	3	1	1	1
1	2	2	1	1	1	1	2	1

1	2	3	1	1	1	1	1	1
5	5	5	1	5	5	5	5	5
1	1	1	7	1	1	3	3	1
2	1	1	8	1	1	1	1	3
1	1	1	2	5	1	2	2	1
1	1	1	2	1	1	1	1	1
1	3	6	4	1	1	7	7	3
1	3	7	9	1	2	1	8	1
1	1	1	9	1	7	1	3	1
1	1	1	3	1	1	1	3	1
1	2	5	5	1	1	1	5	1
6	6	6	1	1	1	1	1	1
2	3	4	3	1	2	2	3	1
1	1	7	7	1	1	7	7	1
1	1	1	2	1	1	1	2	1
1	1	8	1	1	1	1	1	1
1	4	5	2	1	2	3	2	1
1	1	4	6	1	3	2	7	1
1	2	3	3	2	1	1	5	1
1	1	1	5	1	1	1	1	1
1	1	3	5	1	1	1	5	6
1	1	3	2	1	1	1	3	1
1	1	1	1	1	1	1	1	1
1	1	2	3	2	1	1	3	1
1	2	7	7	1	1	5	6	4
1	1	2	1	1	1	1	1	1
2	1	1	1	1	1	1	1	1
1	1	5	1	1	1	1	1	1
2	2	2	7	1	1	1	8	1
1	2	2	1	1	1	1	1	1
1	1	3	1	1	1	1	3	1
1	2	3	1	1	1	1	1	1
1	1	1	1	1	1	1	1	1
5	2	3	8	5	5	6	7	7
1	1	5	1	1	1	1	2	1
2	8	6	7	7	1	3	3	1
1	2	3	2	2	1	2	2	1
2	2	7	7	3	4	1	9	9
1	3	3	6	1	1	1	6	1
1	1	1	3	1	1	1	1	1
1	1	1	1	1	1	1	1	1
1	2	2	4	1	1	1	2	1
1	1	7	1	1	1	1	1	1
5	5	5	5	1	1	1	1	1
1	1	3	1	1	1	1	3	1
1	1	1	2	1	1	1	1	1
1	1	3	2	5	1	2	3	1
1	1	6	7	1	1	8	6	1
2	2	2	1	1	1	1	1	1
2	2	4	4	5	1	1	2	1

1	1	1	1	1	1	1	1	1
5	1	3	5	5	1	6	8	5
7	9	7	9	5	9	5	5	9
3	2	7	6	1	1	1	3	1
1	1	2	1	1	1	1	2	1
1	3	3	5	3	3	2	2	1
2	5	5	7	1	5	3	5	3
1	1	1	5	1	1	1	2	1
1	9	1	7	1	9	1	1	1
1	1	2	1	1	1	1	1	1
1	2	5	5	1	1	1	6	1
9	8	7	8	2	8	8	7	8
1	4	3	6	1	7	1	7	1
1	1	6	3	1	1	6	7	1
1	2	2	1	1	1	4	4	1
1	2	2	1	1	1	1	1	1
1	1	2	5	5	1	1	6	6
1	1	8	3	1	1	6	7	1
1	1	5	5	1	1	6	7	6
1	2	2	8	7	1	1	2	8
1	1	2	1	1	1	1	2	1
1	2	7	7	2	1	2	6	5
5	3	4	6	1	2	4	3	2
1	1	1	5	5	1	1	1	1
1	2	1	4	5	2	2	2	8
1	2	3	3	2	2	2	4	3
1	2	2	3	1	1	1	3	1
1	1	6	8	1	1	1	4	1
1	1	3	9	1	1	1	8	1
1	2	1	1	1	1	1	1	1
1	1	1	2	1	1	7	1	1
1	3	4	3	1	4	1	6	1
1	1	3	1	1	1	1	1	1
1	2	6	5	1	1	1	5	6
1	2	6	7	2	2	8	3	1
1	1	1	1	1	1	1	1	1
2	2	3	3	1	1	1	1	1
1	1	4	5	1	1	1	6	1
5	5	7	1	1	1	6	8	1
5	5	5	8	5	5	5	7	5
1	2	3	5	1	2	1	4	2
1	1	3	8	1	1	1	5	1
1	2	3	7	1	2	2	4	1
1	1	1	1	1	1	7	2	1
5	5	4	3	2	1	3	4	1
1	2	2	3	2	6	1	4	4
1	1	6	7	1	1	1	9	1
5	7	7	6	7	1	4	6	1
1	1	3	2	1	1	1	3	1
1	1	1	1	1	1	1	1	1

3	3	5	1	1	1	1	2	2
1	1	1	1	1	1	1	1	1
2	1	2	1	1	1	1	2	1
1	1	4	2	1	1	2	3	2
1	2	7	2	1	1	1	6	1
1	1	2	5	2	2	2	7	3
1	2	4	6	1	1	2	1	1
2	2	6	7	1	1	1	8	2
1	3	7	1	1	1	1	6	1
1	1	3	2	1	1	1	1	1
1	1	2	1	1	1	2	2	1
1	2	3	6	1	1	1	6	1
1	1	1	1	1	1	1	1	1
1	1	3	4	4	5	5	8	6
5	5	6	5	5	7	3	6	5
1	1	2	5	1	1	1	1	1
1	4	1	9	8	1	2	8	9
1	3	2	8	3	6	6	6	6
1	1	1	7	1	1	1	1	1
2	1	4	7	1	1	1	6	1
1	1	1	1	1	1	1	3	1
1	1	3	9	5	1	1	7	5
1	1	1	6	1	1	1	1	1
1	3	4	7	1	3	1	5	1
1	2	4	4	1	1	2	3	3
2	2	2	6	1	1	1	6	1
1	2	4	1	1	1	1	2	1
1	2	3	2	2	1	1	2	1
1	2	5	7	1	1	1	7	1
3	2	3	7	1	1	3	3	1
1	1	1	4	1	1	1	1	1
1	1	4	3	2	1	1	3	1
4	6	6	6	1	7	5	8	6
1	1	2	7	1	1	2	2	1
1	1	3	5	2	2	1	1	1
1	1	1	7	2	1	1	2	1
1	1	2	2	1	1	1	1	1
1	1	4	7	1	1	2	2	1
2	3	5	8	1	6	1	5	6
1	1	2	4	5	1	1	1	8
1	2	1	2	1	3	1	1	1
1	1	6	3	2	1	2	2	1
1	1	2	1	1	1	1	4	1
1	1	1	4	1	5	1	7	2
1	1	1	5	1	1	1	3	1
1	2	1	1	1	1	1	1	1
1	1	6	1	1	1	1	3	1
1	1	2	3	1	1	1	3	1
1	2	5	5	2	2	1	3	2
1	1	4	8	1	1	1	4	2

1	5	4	1	1	1	1	2	1
1	2	1	2	1	1	1	1	1
1	1	1	1	1	1	1	1	1
1	1	1	2	1	1	1	1	1
1	3	3	2	1	1	7	8	5
1	1	1	5	1	1	5	3	1
1	2	2	6	2	1	1	2	1
5	4	6	3	2	6	3	3	2
1	2	3	1	1	1	1	2	1
1	1	1	7	4	1	1	1	1
2	4	4	4	2	2	2	3	2
1	5	3	1	1	1	1	1	1
1	1	1	1	1	1	2	3	1
1	6	6	4	6	5	5	6	1
1	1	2	4	1	1	1	1	1
1	2	3	1	1	1	1	1	1
1	1	1	4	5	1	1	5	5
1	5	7	5	5	5	5	5	6
1	6	6	3	1	1	1	2	3
3	2	1	7	1	1	1	1	1
1	1	2	5	1	1	1	1	1
1	2	2	2	5	1	2	2	1
1	2	2	1	1	1	1	1	1
5	5	7	3	1	1	7	8	8
1	1	3	6	1	1	1	1	1
1	1	2	1	1	1	1	8	8
1	3	2	3	1	6	1	3	1
5	3	4	4	1	2	1	2	2
1	5	1	1	1	1	1	1	1
1	1	5	2	1	1	2	2	1
1	1	1	1	6	1	1	1	1
1	1	3	1	1	1	1	2	1
1	1	1	1	1	3	1	1	1
3	7	7	1	1	1	1	1	1
1	1	4	4	1	1	1	3	1
5	5	5	1	1	1	1	1	1
1	5	5	9	5	1	6	6	2
1	3	3	8	2	3	1	4	1
1	1	1	1	1	1	1	7	1
1	1	1	7	5	2	1	9	1
3	3	2	6	1	3	3	3	3
1	1	5	7	1	1	1	1	1
2	2	2	1	1	3	1	1	2
2	4	4	5	3	1	5	5	2
1	1	8	7	3	1	3	4	1
1	1	2	2	5	5	5	5	5
1	3	1	6	1	2	2	2	1
1	2	6	2	2	6	2	6	1
1	5	5	4	1	2	2	5	1
1	1	1	5	3	1	2	1	2

1	1	6	5	1	1	1	4	3
1	1	4	8	1	1	3	7	1
1	1	1	4	1	1	1	1	1
1	2	2	4	1	1	1	2	1
1	1	1	1	1	1	1	1	1
1	1	1	1	1	1	3	6	1
1	1	2	7	5	1	5	5	6
1	1	1	1	1	1	1	1	1
1	1	1	2	2	1	1	1	1
1	1	6	7	1	1	1	5	1
1	1	3	5	2	1	1	1	1
1	7	3	7	9	1	1	2	1
1	1	2	1	1	1	1	2	1
1	1	1	2	5	1	1	1	1
2	7	5	6	2	2	2	2	3
2	2	5	5	5	2	2	2	1
2	4	2	3	1	2	2	1	3
5	5	3	5	2	5	5	8	1
1	1	2	2	1	1	1	2	1
1	1	2	2	1	1	1	1	1
8	5	8	9	9	5	5	8	8
3	7	7	4	1	5	3	2	1
1	7	5	1	6	1	1	1	1
1	1	3	7	6	1	2	3	1
3	4	5	7	1	2	1	5	3
8	8	8	5	1	1	1	1	1
1	1	2	1	5	1	1	1	1
5	5	7	5	5	5	5	7	5
1	3	2	1	1	5	1	2	1
1	1	2	5	5	1	1	1	1
1	1	1	1	1	1	1	1	1
1	3	1	2	5	5	1	2	2
1	1	1	1	5	1	1	1	1
1	1	1	2	1	1	1	4	2
3	2	3	7	2	3	7	6	9
1	2	6	5	2	1	2	4	1
3	2	3	8	6	1	2	7	2
1	1	2	5	5	1	6	1	1
1	1	1	5	1	1	1	1	1
5	5	5	3	1	1	1	1	2
7	6	5	5	5	5	5	1	1
1	1	3	3	1	9	9	1	1
1	1	5	1	1	1	1	1	1
2	3	2	2	2	1	1	2	1
7	7	8	5	5	5	5	5	6
1	2	2	2	1	1	1	1	1
1	2	2	1	1	2	1	2	1
1	1	2	3	6	1	2	4	1
2	2	2	1	1	1	1	1	1
1	4	5	1	1	1	1	1	1

5	5	5	7	2	2	7	7	5
1	1	5	2	1	1	1	3	1
1	1	5	2	1	1	1	1	1
1	1	3	8	1	1	1	1	1
1	2	1	1	5	1	1	1	1
1	2	2	1	1	1	1	1	1
2	4	3	3	1	3	1	2	6
1	1	9	1	9	1	1	9	1
2	4	5	2	1	2	3	4	1
1	2	3	3	1	1	3	4	1
1	3	2	1	1	1	1	1	1
1	1	1	7	5	1	1	1	1
2	2	2	2	1	1	1	1	1
1	1	2	3	1	2	1	1	1
1	1	2	1	6	1	1	1	1
2	1	1	5	5	1	1	1	1
1	1	3	7	4	2	2	7	4
1	1	2	1	1	1	1	1	1
1	1	7	1	5	1	1	1	1
2	3	6	2	1	1	1	4	1
1	1	2	1	1	1	1	1	3
2	1	1	2	1	2	2	1	1
1	1	3	7	1	2	1	7	2
1	2	5	2	1	1	1	2	1
1	2	6	5	1	2	2	9	1
1	1	1	6	1	1	1	1	1
1	2	6	3	2	1	1	3	1
1	1	1	1	1	1	1	1	1
1	3	1	1	1	1	1	1	1
1	2	3	2	1	2	1	2	1
1	1	2	2	2	1	1	1	1
1	3	4	4	2	5	1	3	1
1	1	7	3	2	1	1	7	1
1	8	7	7	5	6	4	3	2
1	1	1	7	2	5	1	1	2
2	5	4	2	2	1	1	1	2
1	1	1	2	1	1	1	1	1
1	1	2	2	1	1	1	1	1
2	4	4	3	3	2	1	2	1
3	3	3	7	1	1	1	1	1
1	1	5	5	1	1	1	2	1
1	2	2	2	2	1	1	2	1
1	1	6	5	1	1	1	8	1
1	1	1	1	1	1	1	1	1
1	1	1	9	1	1	1	1	9
1	2	6	8	2	1	2	8	4
4	4	3	5	5	1	1	1	1
1	1	1	2	1	1	1	1	1
1	1	2	1	1	1	2	2	1
8	8	2	8	5	1	1	9	1

1	3	3	7	3	2	2	2	1
1	2	7	1	1	1	1	2	1
1	1	1	1	1	1	1	5	3
1	1	1	2	1	1	1	1	1
1	3	2	3	2	1	1	2	1
1	1	4	3	2	1	1	9	1
2	1	1	1	4	1	1	1	1
1	1	2	1	1	1	1	3	1
1	1	1	1	1	1	1	1	1
1	1	6	3	2	1	2	3	5
1	1	2	1	1	1	1	5	1
1	3	4	3	1	1	1	7	6
1	1	1	8	2	2	1	6	7
1	1	2	1	1	1	1	1	1
1	3	5	2	2	6	1	3	1
1	2	5	4	1	5	5	6	4
1	1	3	1	1	1	1	1	1
1	2	2	6	2	1	1	1	1
1	2	3	2	2	1	1	2	1
6	8	8	5	2	7	1	8	5
1	9	2	5	9	9	1	9	5
1	3	6	3	1	1	6	7	1
1	1	1	5	3	1	3	3	6
3	4	3	4	5	2	2	1	1
1	5	5	2	1	1	1	5	1
1	1	1	5	5	1	1	1	1
1	1	1	1	1	1	1	1	1
1	1	1	3	3	1	1	1	1
1	2	2	2	5	1	1	5	7
1	1	1	9	1	1	1	1	1
1	6	7	7	4	1	2	4	3

SELB07_B	SELB08_B	SELB09_B	SELB10_B	SW02_B	SW05_B	SW06_B	SW08_B	SW09_B
1	1	1	8	3,00	0,00	2,00	2,00	2,00
6	9	1	5	0,00	0,00	0,00	0,00	0,00
1	1	1	1	3,00	3,00	3,00	3,00	3,00
1	4	3	1	1,00	2,00	0,00	0,00	0,00
5	3	3	6	1,00	1,00	1,00	0,00	1,00
3	1	1	3	2,00	1,00	2,00	1,00	3,00
1	3	1	2	1,00	0,00	1,00	0,00	1,00
2	5	1	1	1,00	1,00	0,00	1,00	1,00
1	4	3	3	2,00	2,00	0,00	1,00	2,00
1	2	1	1	3,00	3,00	3,00	3,00	3,00
1	3	1	1	2,00	2,00	2,00	2,00	3,00
1	3	1	3	0,00	0,00	0,00	1,00	1,00
5	8	5	9	0,00	0,00	0,00	1,00	0,00
1	7	1	9	0,00	0,00	0,00	0,00	0,00
2	2	3	2	3,00	3,00	3,00	1,00	3,00
1	3	1	1	1,00	0,00	1,00	0,00	0,00
9	8	2	4	0,00	1,00	0,00	0,00	1,00
1	5	1	6	0,00	0,00	1,00	0,00	0,00
4	4	5	5	1,00	3,00	1,00	0,00	2,00
1	1	1	1	3,00	3,00	3,00	2,00	3,00
3	3	1	3	1,00	2,00	1,00	0,00	1,00
1	2	1	3	3,00	2,00	3,00	2,00	2,00
1	5	5	5	2,00	2,00	2,00	0,00	1,00
5	9	1	7	3,00	1,00	0,00	2,00	2,00
1	7	3	5	1,00	1,00	1,00	0,00	1,00
1	2	1	2	2,00	1,00	2,00	0,00	1,00
1	3	1	1	2,00	1,00	2,00	0,00	2,00
1	1	1	1	3,00	2,00	3,00	1,00	3,00
1	1	1	9	3,00	0,00	0,00	2,00	2,00
1	9	1	5	0,00	3,00	1,00	0,00	0,00
1	5	1	2	1,00	2,00	0,00	0,00	1,00
3	3	3	6	3,00	3,00	3,00	0,00	1,00
1	8	4	3	0,00	1,00	0,00	0,00	1,00
1	2	1	1	0,00	1,00	0,00	1,00	0,00
2	7	7	7	1,00	1,00	1,00	1,00	1,00
1	1	1	9	1,00	2,00	2,00	1,00	2,00
1	1	1	1	2,00	2,00	2,00	1,00	3,00
1	1	1	8	1,00	1,00	1,00	0,00	0,00
1	1	1	1	2,00	1,00	2,00	2,00	2,00
1	4	3	1	2,00	1,00	1,00	0,00	1,00
2	7	5	7	2,00	0,00	0,00	0,00	1,00
1	1	1	1	2,00	3,00	2,00	2,00	3,00
1	1	1	1	2,00	3,00	3,00	3,00	3,00
1	3	1	1	1,00	3,00	1,00	2,00	2,00
1	2	1	1	3,00	3,00	2,00	2,00	1,00
1	3	1	1	0,00	2,00	1,00	0,00	1,00
7	5	1	2	2,00	2,00	2,00	0,00	3,00
2	1	2	5	1,00	1,00	1,00	0,00	1,00
9	7	5	6	1,00	3,00	0,00	3,00	3,00

1	1	3	3	3,00	1,00	2,00	1,00	2,00
5	5	1	5	3,00	2,00	2,00	0,00	2,00
2	1	1	1	1,00	0,00	0,00	0,00	0,00
8	8	6	9	3,00	0,00	0,00	2,00	1,00
5	1	1	1	3,00	3,00	3,00	0,00	3,00
1	6	1	2	2,00	2,00	2,00	2,00	3,00
1	1	1	4	3,00	3,00	2,00	2,00	2,00
1	1	1	5	1,00	1,00	0,00	0,00	1,00
1	5	1	5	2,00	2,00	2,00	2,00	1,00
3	3	8	7	1,00	0,00	0,00	1,00	0,00
5	8	5	6	1,00	0,00	0,00	1,00	0,00
1	2	1	1	0,00	1,00	1,00	1,00	0,00
3	3	2	2	2,00	3,00	2,00	0,00	2,00
1	1	1	1	3,00	2,00	1,00	3,00	2,00
7	9	2	1	1,00	0,00	0,00	1,00	0,00
2	3	2	2	2,00	1,00	1,00	0,00	1,00
1	1	1	1	3,00	3,00	3,00	3,00	3,00
1	2	1	7	1,00	1,00	1,00	0,00	1,00
2	6	2	1	0,00	1,00	0,00	1,00	0,00
3	1	2	1	3,00	2,00	3,00	0,00	2,00
1	1	1	1	1,00	1,00	1,00	1,00	2,00
1	1	1	1	0,00	2,00	0,00	1,00	3,00
1	1	1	1	3,00	2,00	3,00	2,00	3,00
6	1	2	2	1,00	2,00	2,00	2,00	2,00
3	6	5	8	1,00	0,00	0,00	0,00	1,00
1	1	1	1	3,00	3,00	0,00	1,00	3,00
1	9	1	9	0,00	0,00	0,00	0,00	0,00
1	9	1	6	0,00	0,00	0,00	0,00	0,00
1	1	1	1	2,00	2,00	1,00	1,00	2,00
2	5	2	5	2,00	2,00	2,00	2,00	2,00
1	2	1	1	0,00	1,00	1,00	1,00	0,00
3	2	1	1	0,00	1,00	0,00	1,00	1,00
1	2	1	3	1,00	3,00	0,00	0,00	0,00
1	3	1	3	0,00	2,00	0,00	1,00	1,00
6	6	1	2	1,00	2,00	1,00	0,00	1,00
1	2	1	1	3,00	2,00	3,00	3,00	3,00
1	1	1	1	3,00	0,00	3,00	3,00	3,00
2	2	1	2	2,00	0,00	1,00	2,00	1,00
1	1	1	1	2,00	1,00	2,00	1,00	1,00
1	2	1	1	2,00	2,00	2,00	1,00	2,00
2	5	2	2	1,00	1,00	1,00	0,00	0,00
1	2	2	1	1,00	2,00	1,00	0,00	2,00
3	1	3	3	0,00	2,00	0,00	1,00	1,00
3	1	1	4	0,00	1,00	0,00	2,00	2,00
5	5	5	6	1,00	2,00	1,00	1,00	1,00
8	7	6	8	3,00	2,00	0,00	0,00	3,00
2	6	6	7	0,00	1,00	1,00	1,00	1,00
2	3	2	3	3,00	2,00	1,00	0,00	1,00
8	1	5	9	1,00	0,00	0,00	1,00	0,00
2	8	1	1	0,00	0,00	0,00	2,00	0,00

1	1	1	2	2,00	3,00	1,00	3,00	2,00
2	6	6	2	1,00	2,00	0,00	0,00	0,00
1	3	1	2	2,00	3,00	2,00	0,00	1,00
1	7	5	3	1,00	1,00	1,00	1,00	1,00
1	1	1	1	3,00	3,00	3,00	1,00	3,00
2	3	1	1	1,00	2,00	2,00	0,00	1,00
1	5	1	1	2,00	1,00	1,00	0,00	1,00
1	4	1	1	1,00	2,00	1,00	1,00	1,00
2	6	3	4	0,00	1,00	0,00	0,00	0,00
3	4	2	3	3,00	2,00	2,00	2,00	2,00
5	3	1	2	1,00	2,00	0,00	2,00	2,00
2	1	4	2	2,00	1,00	1,00	1,00	1,00
1	2	1	2	2,00	2,00	3,00	0,00	2,00
1	1	1	6	3,00	2,00	1,00	1,00	2,00
1	6	1	4	0,00	0,00	0,00	2,00	1,00
5	3	1	1	3,00	3,00	2,00	2,00	3,00
2	3	2	9	1,00	2,00	3,00	1,00	1,00
1	3	3	3	2,00	2,00	2,00	2,00	2,00
1	1	1	1	3,00	3,00	3,00	2,00	2,00
2	1	1	3	2,00	2,00	3,00	0,00	1,00
1	5	2	1	0,00	3,00	0,00	0,00	1,00
1	2	1	2	2,00	2,00	1,00	0,00	2,00
9	6	6	5	0,00	1,00	1,00	0,00	0,00
2	4	1	3	1,00	2,00	1,00	0,00	2,00
3	1	3	7	1,00	0,00	1,00	0,00	1,00
6	7	2	1	0,00	1,00	0,00	1,00	1,00
1	2	2	1	0,00	0,00	0,00	0,00	1,00
1	1	1	1	1,00	3,00	3,00	0,00	2,00
2	2	1	1	1,00	2,00	2,00	3,00	3,00
2	5	3	5	0,00	0,00	0,00	0,00	0,00
1	1	1	1	1,00	2,00	2,00	1,00	1,00
3	2	1	3	1,00	3,00	3,00	2,00	2,00
1	1	1	1	1,00	3,00	2,00	0,00	1,00
1	6	1	6	3,00	3,00	3,00	3,00	3,00
1	2	1	1	1,00	2,00	1,00	0,00	1,00
7	6	8	9	0,00	0,00	0,00	0,00	0,00
1	2	1	1	3,00	3,00	2,00	3,00	3,00
6	3	3	2	2,00	2,00	0,00	1,00	2,00
3	2	1	1	1,00	0,00	0,00	0,00	1,00
1	1	1	6	2,00	2,00	1,00	2,00	3,00
6	5	1	3	2,00	1,00	3,00	0,00	2,00
1	1	1	6	3,00	3,00	3,00	3,00	2,00
4	9	6	5	1,00	3,00	0,00	0,00	1,00
2	9	7	2	0,00	0,00	1,00	1,00	0,00
6	9	6	9	2,00	1,00	1,00	1,00	0,00
1	1	1	1	1,00	2,00	1,00	0,00	0,00
1	1	1	1	1,00	1,00	3,00	2,00	3,00
6	8	2	9	0,00	0,00	0,00	1,00	1,00
1	2	1	2	2,00	2,00	2,00	2,00	2,00
2	1	1	9	3,00	2,00	1,00	2,00	3,00

1	1	1	8	3,00	3,00	3,00	2,00	3,00
1	8	1	1	3,00	3,00	2,00	3,00	3,00
3	1	1	1	3,00	1,00	2,00	1,00	2,00
7	8	6	1	1,00	1,00	1,00	1,00	1,00
1	1	1	6	3,00	3,00	3,00	3,00	3,00
2	1	1	1	0,00	1,00	0,00	1,00	1,00
2	1	1	1	1,00	2,00	1,00	2,00	2,00
1	2	1	1	1,00	2,00	1,00	1,00	1,00
1	2	1	1	3,00	3,00	2,00	3,00	3,00
2	1	1	1	2,00	1,00	3,00	1,00	2,00
2	4	4	6	1,00	1,00	1,00	1,00	1,00
3	7	6	6	1,00	0,00	1,00	0,00	2,00
1	5	1	1	2,00	2,00	1,00	1,00	3,00
6	1	1	1	3,00	3,00	3,00	2,00	3,00
1	1	1	1	2,00	3,00	2,00	1,00	2,00
6	9	8	8	0,00	1,00	0,00	0,00	1,00
1	7	1	1	2,00	1,00	1,00	0,00	1,00
1	3	1	9	0,00	2,00	0,00	0,00	1,00
2	1	2	2	1,00	1,00	1,00	0,00	1,00
6	7	7	2	0,00	0,00	0,00	0,00	0,00
2	9	1	1	0,00	1,00	2,00	1,00	0,00
7	5	1	6	2,00	3,00	0,00	1,00	1,00
1	7	1	9	0,00	0,00	0,00	1,00	0,00
1	3	1	2	0,00	1,00	0,00	2,00	0,00
7	5	7	8	3,00	1,00	2,00	2,00	2,00
1	1	1	1	3,00	3,00	2,00	3,00	2,00
4	2	4	2	1,00	0,00	1,00	0,00	1,00
1	7	6	6	1,00	1,00	1,00	3,00	0,00
3	1	1	9	0,00	1,00	0,00	0,00	0,00
9	7	6	1	1,00	3,00	0,00	0,00	0,00
3	1	1	1	3,00	3,00	3,00	3,00	3,00
4	6	2	2	1,00	1,00	1,00	1,00	1,00
6	3	3	1	0,00	1,00	0,00	0,00	2,00
1	2	1	7	2,00	1,00	2,00	2,00	2,00
4	1	1	4	2,00	3,00	3,00	1,00	3,00
7	4	7	2	0,00	1,00	2,00	0,00	0,00
7	3	2	1	1,00	2,00	0,00	0,00	1,00
1	2	1	5	0,00	1,00	1,00	0,00	1,00
2	5	4	3	0,00	1,00	0,00	0,00	0,00
1	7	1	1	2,00	1,00	2,00	0,00	0,00
2	2	3	4	1,00	2,00	1,00	0,00	2,00
1	3	1	2	2,00	2,00	2,00	0,00	0,00
7	7	5	7	0,00	1,00	0,00	0,00	0,00
1	1	1	1	0,00	0,00	0,00	0,00	0,00
2	1	1	2	1,00	0,00	0,00	0,00	0,00
1	1	1	5	3,00	2,00	1,00	1,00	2,00
1	1	1	1	3,00	3,00	3,00	1,00	3,00
7	1	6	8	0,00	0,00	1,00	1,00	0,00
1	6	1	1	2,00	3,00	1,00	0,00	3,00
1	8	1	8	0,00	0,00	0,00	1,00	0,00

9	9	9	9	0,00	0,00	0,00	0,00	0,00
1	6	6	1	0,00	0,00	0,00	0,00	1,00
1	2	1	6	2,00	1,00	1,00	3,00	2,00
2	8	2	8	0,00	2,00	2,00	1,00	1,00
1	2	1	9	0,00	0,00	0,00	1,00	0,00
4	6	4	4	1,00	1,00	1,00	2,00	1,00
7	6	1	7	0,00	1,00	0,00	1,00	0,00
1	6	1	9	1,00	1,00	1,00	1,00	1,00
7	8	5	9	1,00	1,00	1,00	0,00	0,00
1	5	1	1	1,00	2,00	0,00	0,00	1,00
5	2	6	6	0,00	1,00	0,00	1,00	0,00
4	1	3	8	3,00	3,00	2,00	2,00	3,00
8	2	1	8	0,00	0,00	0,00	0,00	1,00
3	1	1	6	0,00	2,00	1,00	1,00	0,00
1	3	1	1	0,00	1,00	0,00	0,00	0,00
5	5	5	6	1,00	2,00	2,00	0,00	0,00
1	1	1	1	2,00	2,00	2,00	0,00	2,00
1	1	1	3	0,00	1,00	0,00	1,00	0,00
1	1	1	1	2,00	2,00	1,00	3,00	3,00
6	4	6	1	1,00	0,00	0,00	1,00	1,00
1	2	1	1	2,00	3,00	2,00	1,00	2,00
1	5	1	7	0,00	1,00	0,00	1,00	0,00
2	8	7	9	2,00	3,00	3,00	0,00	3,00
2	2	2	1	0,00	2,00	1,00	0,00	1,00
7	1	6	1	1,00	2,00	2,00	0,00	1,00
3	7	3	5	1,00	2,00	1,00	1,00	2,00
1	6	1	8	0,00	0,00	0,00	0,00	0,00
8	2	2	7	1,00	1,00	1,00	0,00	2,00
1	1	1	1	1,00	3,00	1,00	1,00	2,00
6	3	5	9	1,00	2,00	1,00	3,00	0,00
7	1	1	5	1,00	2,00	1,00	1,00	2,00
1	3	4	4	2,00	3,00	2,00	1,00	2,00
1	1	1	1	3,00	3,00	3,00	1,00	2,00
3	3	1	6	1,00	2,00	1,00	2,00	2,00
1	1	1	2	0,00	0,00	0,00	1,00	0,00
2	3	1	2	2,00	2,00	1,00	0,00	1,00
1	3	1	2	1,00	2,00	1,00	1,00	2,00
1	6	1	2	2,00	3,00	3,00	0,00	2,00
7	5	4	2	0,00	1,00	0,00	0,00	0,00
2	7	2	9	1,00	1,00	1,00	0,00	0,00
5	2	3	8	0,00	0,00	0,00	3,00	1,00
1	1	3	1	1,00	2,00	0,00	0,00	1,00
1	1	1	7	2,00	2,00	3,00	1,00	3,00
1	1	1	7	2,00	3,00	1,00	3,00	2,00
1	1	1	6	1,00	1,00	0,00	1,00	1,00
1	1	1	6	0,00	2,00	1,00	3,00	3,00
3	9	1	3	0,00	3,00	0,00	0,00	0,00
1	1	1	1	0,00	2,00	2,00	1,00	1,00
1	2	1	1	1,00	2,00	0,00	0,00	1,00
1	3	1	1	2,00	1,00	1,00	0,00	0,00

2	5	1	1	0,00	1,00	1,00	0,00	1,00
3	1	3	2	2,00	3,00	2,00	1,00	2,00
5	5	5	9	1,00	1,00	2,00	1,00	1,00
1	1	1	1	3,00	2,00	3,00	2,00	3,00
1	1	1	1	3,00	3,00	3,00	3,00	3,00
1	1	1	1	3,00	2,00	2,00	0,00	2,00
1	1	1	1	1,00	2,00	0,00	1,00	1,00
7	2	6	7	1,00	2,00	1,00	0,00	1,00
1	8	1	6	0,00	0,00	0,00	0,00	0,00
2	2	2	3	2,00	2,00	2,00	1,00	2,00
7	1	1	1	0,00	1,00	0,00	1,00	0,00
1	6	1	6	3,00	2,00	3,00	3,00	2,00
9	7	7	8	2,00	2,00	3,00	0,00	0,00
1	5	3	1	2,00	1,00	0,00	0,00	0,00
3	3	3	9	0,00	0,00	0,00	1,00	0,00
1	7	1	1	2,00	0,00	1,00	0,00	0,00
1	4	1	1	1,00	1,00	2,00	2,00	1,00
1	1	3	7	0,00	1,00	2,00	1,00	1,00
1	3	1	1	1,00	2,00	1,00	1,00	2,00
1	5	1	1	0,00	1,00	0,00	1,00	1,00
1	1	1	1	1,00	2,00	2,00	0,00	1,00
5	9	2	9	0,00	0,00	0,00	2,00	0,00
3	7	7	9	0,00	1,00	0,00	0,00	0,00
1	1	1	1	2,00	2,00	2,00	2,00	2,00
1	1	1	1	2,00	2,00	1,00	1,00	1,00
1	1	1	5	2,00	2,00	0,00	1,00	3,00
6	1	1	9	0,00	1,00	0,00	0,00	0,00
6	6	4	8	0,00	1,00	0,00	1,00	1,00
1	3	2	2	1,00	2,00	1,00	1,00	1,00
1	3	2	4	0,00	1,00	2,00	0,00	0,00
8	5	5	6	0,00	1,00	0,00	0,00	0,00
7	5	6	1	1,00	0,00	0,00	1,00	1,00
5	7	7	6	0,00	0,00	0,00	1,00	1,00
1	1	1	5	2,00	2,00	2,00	0,00	2,00
5	7	5	6	0,00	2,00	3,00	1,00	0,00
7	2	6	1	0,00	3,00	0,00	1,00	1,00
1	1	1	8	3,00	3,00	3,00	3,00	3,00
2	6	4	7	0,00	2,00	2,00	2,00	2,00
1	2	1	8	1,00	2,00	1,00	0,00	1,00
1	1	1	1	1,00	1,00	1,00	1,00	1,00
1	3	2	4	1,00	2,00	1,00	1,00	1,00
9	9	8	1	0,00	1,00	1,00	0,00	0,00
6	6	7	8	1,00	1,00	0,00	1,00	1,00
1	1	1	3	3,00	2,00	2,00	1,00	2,00
2	1	2	2	3,00	3,00	2,00	3,00	3,00
1	5	1	9	1,00	2,00	0,00	3,00	2,00
1	7	6	9	0,00	0,00	0,00	1,00	0,00
7	6	1	1	2,00	2,00	1,00	0,00	1,00
6	9	7	9	0,00	0,00	1,00	1,00	1,00
2	3	3	1	2,00	1,00	0,00	0,00	1,00

1	5	1	1	3,00	1,00	2,00	0,00	0,00
1	1	1	1	1,00	2,00	2,00	1,00	1,00
6	5	1	8	3,00	0,00	0,00	0,00	0,00
3	1	1	6	2,00	3,00	1,00	0,00	2,00
3	3	1	5	2,00	0,00	1,00	0,00	0,00
6	1	6	6	2,00	2,00	0,00	1,00	0,00
2	3	1	2	0,00	1,00	0,00	0,00	0,00
1	1	1	1	0,00	1,00	0,00	2,00	0,00
1	7	2	2	1,00	2,00	1,00	1,00	2,00
1	1	1	7	3,00	3,00	3,00	3,00	3,00
1	7	1	1	1,00	2,00	0,00	1,00	1,00
1	1	1	2	2,00	3,00	0,00	3,00	2,00
8	7	3	8	0,00	1,00	1,00	0,00	0,00
5	1	5	8	1,00	1,00	1,00	1,00	1,00
6	5	3	9	1,00	0,00	0,00	0,00	1,00
1	1	1	1	0,00	2,00	0,00	1,00	0,00
7	3	5	3	0,00	0,00	0,00	1,00	0,00
3	1	1	1	1,00	1,00	1,00	0,00	1,00
5	7	1	4	1,00	1,00	0,00	3,00	1,00
1	1	1	6	0,00	2,00	3,00	3,00	0,00
1	1	1	1	1,00	3,00	1,00	2,00	2,00
3	2	2	1	2,00	2,00	2,00	3,00	2,00
1	3	1	3	1,00	3,00	0,00	0,00	1,00
1	1	1	4	2,00	2,00	2,00	2,00	2,00
9	2	7	8	0,00	1,00	0,00	0,00	0,00
1	6	1	5	3,00	1,00	2,00	0,00	0,00
7	3	3	5	3,00	0,00	0,00	0,00	0,00
1	1	1	1	2,00	3,00	2,00	3,00	3,00
1	1	1	2	2,00	3,00	2,00	0,00	1,00
1	1	1	1	0,00	0,00	0,00	0,00	0,00
1	4	1	1	3,00	1,00	2,00	1,00	2,00
1	5	2	9	1,00	0,00	2,00	3,00	1,00
1	2	2	4	2,00	3,00	1,00	2,00	2,00
1	4	1	1	2,00	2,00	2,00	0,00	1,00
7	2	1	4	1,00	1,00	0,00	0,00	1,00
1	1	1	2	1,00	2,00	1,00	0,00	0,00
1	6	1	1	1,00	1,00	1,00	0,00	1,00
1	7	1	8	0,00	0,00	0,00	0,00	0,00
6	5	5	9	0,00	1,00	0,00	0,00	0,00
1	6	1	9	0,00	2,00	0,00	0,00	0,00
1	1	1	3	1,00	2,00	1,00	0,00	2,00
2	2	1	4	2,00	1,00	2,00	1,00	2,00
1	6	1	2	1,00	1,00	3,00	0,00	1,00
6	1	2	2	0,00	1,00	1,00	1,00	1,00
1	6	1	1	0,00	0,00	1,00	2,00	2,00
2	3	2	7	2,00	2,00	1,00	0,00	2,00
6	8	2	5	0,00	2,00	0,00	0,00	1,00
3	5	6	6	2,00	2,00	2,00	2,00	2,00
3	9	2	7	0,00	1,00	0,00	1,00	1,00
2	5	3	3	1,00	0,00	1,00	0,00	0,00

1	1	1	1	2,00	2,00	2,00	0,00	2,00
2	7	1	5	0,00	1,00	0,00	1,00	0,00
1	1	1	2	1,00	1,00	0,00	0,00	1,00
1	2	1	8	3,00	3,00	0,00	2,00	3,00
1	8	7	7	0,00	0,00	1,00	1,00	0,00
1	1	1	1	2,00	1,00	2,00	1,00	2,00
1	9	1	6	0,00	0,00	0,00	0,00	0,00
1	1	1	2	3,00	3,00	3,00	3,00	3,00
1	3	1	5	1,00	3,00	3,00	1,00	3,00
1	1	1	1	2,00	3,00	2,00	3,00	3,00
3	1	1	1	1,00	1,00	1,00	1,00	2,00
1	1	1	1	2,00	1,00	1,00	0,00	1,00
1	1	1	5	1,00	2,00	1,00	2,00	1,00
2	2	1	2	2,00	1,00	0,00	0,00	1,00
6	1	1	7	1,00	3,00	1,00	1,00	1,00
1	1	1	6	2,00	2,00	0,00	1,00	1,00
3	5	2	4	1,00	1,00	1,00	1,00	1,00
1	1	1	1	2,00	3,00	0,00	0,00	0,00
1	8	3	5	1,00	1,00	0,00	0,00	1,00
6	2	6	9	1,00	0,00	1,00	0,00	2,00
6	8	1	6	1,00	1,00	1,00	0,00	0,00
7	3	5	4	0,00	0,00	0,00	1,00	0,00
6	4	4	7	1,00	2,00	2,00	1,00	2,00
2	1	1	9	0,00	2,00	0,00	0,00	2,00
1	3	1	2	1,00	1,00	0,00	0,00	0,00
3	1	3	3	1,00	1,00	1,00	0,00	1,00
1	3	2	1	2,00	3,00	3,00	1,00	1,00
4	2	2	6	1,00	0,00	0,00	1,00	1,00
1	1	1	9	3,00	3,00	2,00	2,00	3,00
1	2	1	1	0,00	0,00	3,00	1,00	0,00
1	1	2	1	1,00	2,00	3,00	1,00	3,00
6	7	3	9	0,00	1,00	0,00	0,00	0,00
1	3	1	1	3,00	3,00	3,00	1,00	3,00
1	1	1	1	1,00	2,00	0,00	1,00	3,00
8	9	9	9	0,00	0,00	0,00	1,00	1,00
1	1	1	1	1,00	2,00	1,00	2,00	1,00
1	3	1	1	0,00	1,00	0,00	2,00	1,00
1	1	1	1	3,00	3,00	3,00	3,00	3,00
1	5	1	3	1,00	2,00	1,00	1,00	1,00
2	2	1	9	0,00	0,00	1,00	2,00	1,00
1	1	1	1	2,00	2,00	1,00	2,00	3,00
1	1	1	6	1,00	3,00	2,00	1,00	2,00
1	6	1	2	0,00	0,00	0,00	0,00	0,00
1	3	1	1	2,00	3,00	3,00	2,00	3,00
3	1	1	2	1,00	2,00	2,00	0,00	3,00
5	4	1	7	0,00	0,00	0,00	1,00	0,00
2	2	1	3	1,00	1,00	1,00	0,00	1,00
7	5	2	2	2,00	3,00	2,00	0,00	1,00
4	6	7	7	1,00	0,00	2,00	0,00	1,00
3	2	1	2	1,00	0,00	0,00	0,00	0,00

6	8	1	8	0,00	1,00	0,00	0,00	0,00
5	5	5	5	3,00	3,00	2,00	3,00	3,00
1	5	1	1	2,00	3,00	3,00	0,00	1,00
4	8	2	8	0,00	0,00	0,00	0,00	0,00
2	6	2	6	0,00	0,00	0,00	0,00	0,00
1	1	1	1	1,00	1,00	1,00	0,00	0,00
3	1	3	8	1,00	2,00	0,00	0,00	1,00
3	7	1	9	0,00	0,00	0,00	0,00	0,00
8	1	5	7	3,00	2,00	3,00	3,00	3,00
3	7	1	7	0,00	0,00	0,00	0,00	0,00
1	1	1	5	2,00	2,00	1,00	3,00	3,00
1	1	1	1	3,00	3,00	3,00	3,00	3,00
4	2	7	6	1,00	2,00	0,00	0,00	1,00
1	6	1	9	0,00	0,00	2,00	0,00	1,00
1	1	1	1	2,00	2,00	3,00	1,00	2,00
1	1	1	3	3,00	3,00	3,00	3,00	3,00
5	5	5	5	2,00	2,00	0,00	1,00	2,00
3	2	2	8	1,00	2,00	1,00	1,00	1,00
3	3	1	5	1,00	0,00	0,00	0,00	1,00
1	1	1	1	3,00	2,00	3,00	3,00	3,00
3	6	1	1	1,00	0,00	0,00	0,00	0,00
1	4	1	2	2,00	2,00	2,00	1,00	2,00
1	1	1	4	1,00	0,00	0,00	0,00	2,00
1	2	1	2	2,00	2,00	2,00	0,00	1,00
5	9	3	9	0,00	1,00	0,00	0,00	0,00
1	1	1	2	2,00	3,00	2,00	2,00	3,00
2	1	1	1	0,00	2,00	0,00	1,00	1,00
1	1	1	1	3,00	3,00	3,00	3,00	3,00
1	1	1	1	2,00	2,00	1,00	1,00	1,00
1	3	2	2	2,00	2,00	2,00	1,00	3,00
1	1	1	2	3,00	3,00	2,00	2,00	3,00
1	1	1	1	1,00	0,00	1,00	0,00	2,00
1	1	1	1	3,00	3,00	3,00	3,00	3,00
6	2	2	9	1,00	0,00	0,00	1,00	1,00
2	1	1	2	1,00	1,00	1,00	1,00	1,00
1	1	1	1	0,00	0,00	0,00	0,00	0,00
2	2	2	2	2,00	2,00	2,00	2,00	1,00
1	4	3	9	0,00	0,00	0,00	2,00	0,00
1	7	1	1	2,00	2,00	2,00	0,00	2,00
1	3	1	1	0,00	3,00	1,00	2,00	2,00
1	1	1	3	1,00	2,00	0,00	0,00	2,00
1	2	1	2	1,00	2,00	2,00	0,00	1,00
1	1	1	1	1,00	1,00	0,00	0,00	1,00
1	1	1	1	1,00	2,00	2,00	0,00	2,00
1	7	1	1	3,00	3,00	3,00	3,00	3,00
1	1	1	2	0,00	0,00	0,00	0,00	0,00
1	1	1	1	3,00	3,00	2,00	0,00	3,00
6	6	1	5	1,00	1,00	2,00	0,00	1,00
1	3	1	2	0,00	0,00	0,00	0,00	1,00
2	1	1	1	1,00	2,00	2,00	1,00	2,00

1	1	1	1	0,00	0,00	0,00	3,00	0,00
5	8	8	8	0,00	0,00	0,00	0,00	0,00
9	9	9	7	0,00	0,00	0,00	2,00	0,00
1	1	1	5	2,00	3,00	1,00	0,00	1,00
2	1	2	2	1,00	2,00	2,00	0,00	2,00
3	5	1	9	0,00	1,00	0,00	0,00	0,00
6	5	3	8	1,00	0,00	0,00	2,00	1,00
1	8	1	1	1,00	3,00	1,00	0,00	0,00
9	2	1	1	0,00	0,00	0,00	0,00	0,00
1	1	1	1	2,00	2,00	2,00	1,00	2,00
1	1	1	6	1,00	3,00	3,00	3,00	3,00
8	7	9	8	2,00	2,00	2,00	0,00	2,00
9	5	6	7	0,00	0,00	0,00	1,00	2,00
7	4	3	7	1,00	0,00	2,00	0,00	0,00
1	1	1	2	1,00	1,00	2,00	0,00	1,00
1	1	1	1	1,00	0,00	0,00	1,00	1,00
1	7	1	7	0,00	3,00	0,00	1,00	0,00
6	7	1	5	1,00	1,00	0,00	0,00	2,00
2	4	1	9	1,00	1,00	2,00	0,00	1,00
3	6	2	8	0,00	0,00	1,00	1,00	0,00
1	1	1	9	2,00	3,00	1,00	2,00	2,00
7	7	3	2	0,00	0,00	0,00	0,00	0,00
4	7	3	1	1,00	2,00	1,00	1,00	1,00
1	1	1	1	1,00	2,00	2,00	0,00	2,00
5	7	2	6	0,00	0,00	1,00	1,00	1,00
3	3	2	3	1,00	1,00	2,00	0,00	1,00
2	2	1	2	2,00	1,00	1,00	0,00	2,00
1	3	1	7	3,00	2,00	2,00	3,00	3,00
6	3	3	6	0,00	0,00	0,00	0,00	0,00
1	5	1	1	0,00	3,00	0,00	0,00	0,00
1	1	1	9	3,00	3,00	3,00	3,00	3,00
7	6	1	7	0,00	0,00	0,00	1,00	0,00
1	1	4	1	1,00	2,00	2,00	0,00	2,00
6	3	6	7	0,00	2,00	0,00	0,00	1,00
1	3	7	1	1,00	2,00	0,00	0,00	1,00
1	1	1	1	3,00	3,00	3,00	3,00	3,00
1	1	1	1	2,00	2,00	2,00	0,00	2,00
1	1	1	6	3,00	3,00	1,00	0,00	2,00
1	1	1	7	0,00	0,00	0,00	0,00	0,00
5	6	5	9	0,00	1,00	0,00	0,00	0,00
2	1	1	3	1,00	0,00	1,00	0,00	1,00
1	1	1	9	0,00	1,00	0,00	1,00	0,00
5	2	3	6	1,00	2,00	1,00	0,00	1,00
3	1	5	3	0,00	0,00	0,00	0,00	0,00
3	2	2	1	2,00	2,00	1,00	2,00	1,00
5	8	3	8	0,00	2,00	0,00	0,00	0,00
1	5	1	1	1,00	1,00	0,00	0,00	0,00
1	7	1	6	3,00	0,00	0,00	3,00	1,00
1	1	1	1	2,00	3,00	1,00	0,00	2,00
1	8	1	1	1,00	2,00	3,00	0,00	2,00

2	2	2	1	1,00	3,00	1,00	0,00	1,00
2	2	1	1	3,00	3,00	3,00	3,00	3,00
1	1	1	2	2,00	2,00	2,00	2,00	2,00
2	1	1	2	2,00	3,00	2,00	2,00	3,00
8	6	5	6	0,00	1,00	1,00	1,00	1,00
3	7	6	8	0,00	0,00	0,00	2,00	1,00
1	6	1	1	1,00	2,00	3,00	0,00	1,00
7	3	2	5	1,00	1,00	0,00	0,00	1,00
4	1	3	5	2,00	1,00	1,00	1,00	2,00
1	5	1	4	0,00	2,00	1,00	0,00	0,00
5	8	3	5	3,00	3,00	3,00	0,00	0,00
3	4	1	1	1,00	1,00	1,00	0,00	1,00
3	1	2	1	3,00	3,00	3,00	3,00	3,00
7	5	2	8	0,00	0,00	0,00	0,00	0,00
6	2	5	5	2,00	2,00	2,00	2,00	2,00
1	5	1	2	0,00	2,00	1,00	0,00	0,00
2	8	9	9	0,00	0,00	0,00	2,00	0,00
7	4	2	4	2,00	2,00	1,00	0,00	2,00
3	1	1	1	1,00	2,00	1,00	0,00	1,00
1	1	1	8	0,00	1,00	0,00	2,00	0,00
1	1	1	6	0,00	1,00	1,00	0,00	1,00
6	6	6	8	0,00	0,00	0,00	0,00	0,00
1	1	1	2	3,00	2,00	2,00	2,00	2,00
6	5	5	7	1,00	0,00	0,00	0,00	0,00
1	2	1	1	0,00	0,00	1,00	0,00	0,00
1	1	1	2	2,00	2,00	1,00	2,00	2,00
1	1	2	8	0,00	1,00	0,00	0,00	0,00
1	2	2	2	2,00	2,00	2,00	2,00	2,00
3	2	1	6	0,00	0,00	0,00	0,00	0,00
3	1	1	7	2,00	2,00	2,00	0,00	2,00
1	1	1	1	0,00	0,00	0,00	1,00	0,00
1	3	3	7	1,00	2,00	1,00	2,00	1,00
6	5	6	2	1,00	0,00	2,00	1,00	1,00
1	1	1	1	2,00	2,00	1,00	3,00	2,00
5	5	1	5	2,00	2,00	1,00	0,00	1,00
6	7	5	8	0,00	0,00	0,00	1,00	1,00
1	1	1	1	3,00	2,00	1,00	3,00	3,00
1	1	1	4	2,00	2,00	1,00	2,00	1,00
5	6	3	4	0,00	1,00	1,00	1,00	1,00
2	7	1	6	0,00	0,00	0,00	1,00	0,00
5	2	5	5	0,00	1,00	3,00	0,00	1,00
1	7	5	3	2,00	1,00	2,00	1,00	2,00
1	1	1	6	0,00	1,00	1,00	1,00	0,00
7	7	5	2	0,00	1,00	2,00	2,00	0,00
2	9	1	8	2,00	1,00	0,00	3,00	2,00
1	3	2	2	1,00	2,00	1,00	0,00	1,00
3	5	1	3	3,00	3,00	3,00	3,00	3,00
2	2	2	7	1,00	3,00	2,00	3,00	3,00
2	3	2	3	1,00	1,00	2,00	1,00	2,00
2	4	3	8	1,00	1,00	1,00	1,00	1,00

1	1	1	1	3,00	3,00	3,00	3,00	1,00
1	1	1	1	2,00	2,00	3,00	2,00	2,00
1	3	1	1	3,00	2,00	1,00	3,00	3,00
2	3	1	2	2,00	3,00	2,00	3,00	2,00
2	6	1	2	1,00	2,00	1,00	1,00	1,00
3	2	1	1	1,00	1,00	1,00	0,00	2,00
2	3	1	5	0,00	2,00	2,00	1,00	1,00
5	2	2	7	2,00	1,00	0,00	1,00	0,00
1	1	1	1	3,00	3,00	2,00	2,00	3,00
1	5	5	5	1,00	0,00	1,00	1,00	0,00
2	3	2	3	2,00	1,00	1,00	1,00	2,00
2	5	1	1	3,00	2,00	3,00	3,00	2,00
1	1	1	1	2,00	3,00	2,00	1,00	3,00
7	9	7	1	1,00	2,00	0,00	0,00	1,00
1	8	1	1	1,00	1,00	1,00	0,00	2,00
1	2	1	1	2,00	0,00	1,00	1,00	2,00
5	1	9	5	3,00	2,00	3,00	3,00	3,00
6	6	8	9	1,00	2,00	0,00	1,00	0,00
1	7	7	8	0,00	0,00	0,00	0,00	1,00
1	3	1	2	1,00	1,00	2,00	1,00	2,00
1	2	1	5	2,00	1,00	2,00	3,00	3,00
1	1	1	2	2,00	2,00	3,00	3,00	2,00
1	1	1	1	1,00	3,00	1,00	3,00	3,00
5	9	2	2	3,00	0,00	2,00	2,00	1,00
3	3	6	1	1,00	2,00	0,00	1,00	2,00
2	1	2	7	0,00	0,00	0,00	0,00	0,00
7	3	1	5	2,00	2,00	2,00	3,00	2,00
4	8	2	6	0,00	2,00	3,00	1,00	0,00
1	9	1	1	3,00	1,00	3,00	2,00	3,00
4	1	1	7	0,00	1,00	0,00	0,00	1,00
1	6	1	2	1,00	1,00	1,00	0,00	0,00
2	2	1	3	3,00	2,00	3,00	3,00	2,00
4	1	1	6	3,00	2,00	3,00	1,00	3,00
1	1	1	1	1,00	3,00	3,00	1,00	3,00
3	3	1	2	1,00	0,00	1,00	2,00	1,00
1	1	2	2	2,00	2,00	2,00	2,00	2,00
6	9	9	9	0,00	0,00	0,00	0,00	0,00
2	6	2	8	1,00	1,00	1,00	1,00	1,00
1	1	1	7	1,00	3,00	1,00	1,00	3,00
9	1	1	9	2,00	0,00	0,00	0,00	0,00
4	3	3	7	0,00	1,00	1,00	0,00	1,00
1	6	3	1	0,00	0,00	1,00	1,00	3,00
6	6	1	6	2,00	1,00	2,00	0,00	1,00
1	7	4	7	1,00	1,00	2,00	1,00	1,00
1	5	1	8	3,00	2,00	2,00	3,00	3,00
5	5	5	5	3,00	0,00	1,00	3,00	3,00
1	5	1	2	3,00	3,00	2,00	3,00	3,00
8	7	7	6	2,00	1,00	2,00	0,00	2,00
3	2	1	1	0,00	1,00	1,00	0,00	0,00
2	5	1	1	2,00	1,00	1,00	1,00	2,00

1	6	1	5	1,00	2,00	2,00	1,00	2,00
7	7	1	1	2,00	2,00	1,00	0,00	3,00
1	7	1	1	1,00	2,00	2,00	0,00	1,00
1	5	1	7	0,00	1,00	0,00	2,00	0,00
1	1	1	1	3,00	3,00	1,00	3,00	3,00
1	1	2	7	0,00	1,00	0,00	0,00	0,00
2	5	5	8	1,00	1,00	1,00	0,00	1,00
1	6	1	1	2,00	3,00	3,00	0,00	2,00
1	2	1	1	2,00	2,00	1,00	1,00	2,00
1	8	1	8	2,00	0,00	1,00	0,00	1,00
6	5	1	1	1,00	2,00	1,00	0,00	0,00
1	9	1	1	2,00	0,00	0,00	0,00	0,00
1	2	1	3	2,00	2,00	2,00	1,00	2,00
1	5	1	1	3,00	3,00	2,00	0,00	3,00
2	2	2	7	1,00	2,00	1,00	1,00	1,00
1	1	1	2	0,00	0,00	0,00	1,00	0,00
3	9	2	1	1,00	1,00	1,00	1,00	0,00
1	1	5	5	2,00	2,00	2,00	2,00	2,00
1	2	1	1	1,00	2,00	2,00	0,00	0,00
1	1	1	1	2,00	1,00	1,00	1,00	2,00
8	1	5	8	0,00	0,00	0,00	1,00	0,00
6	3	7	2	2,00	2,00	3,00	1,00	2,00
1	5	1	1	3,00	3,00	1,00	3,00	3,00
1	1	2	3	0,00	1,00	0,00	0,00	0,00
3	4	2	2	3,00	2,00	2,00	2,00	2,00
1	1	1	1	0,00	0,00	0,00	1,00	1,00
1	3	2	1	2,00	2,00	3,00	3,00	2,00
5	5	5	7	0,00	1,00	0,00	1,00	1,00
2	7	1	6	2,00	2,00	2,00	1,00	2,00
1	1	1	5	2,00	2,00	3,00	1,00	2,00
1	1	1	1	2,00	2,00	2,00	1,00	1,00
2	1	1	1	2,00	2,00	1,00	2,00	1,00
1	1	1	1	3,00	3,00	3,00	3,00	3,00
5	6	3	8	1,00	2,00	0,00	1,00	1,00
7	8	1	9	0,00	1,00	0,00	1,00	1,00
1	4	3	6	2,00	1,00	1,00	1,00	2,00
1	9	2	8	1,00	1,00	2,00	1,00	2,00
1	3	1	5	2,00	2,00	1,00	1,00	1,00
1	5	1	1	3,00	3,00	3,00	2,00	3,00
1	1	1	1	0,00	1,00	0,00	0,00	0,00
3	5	1	7	2,00	1,00	1,00	1,00	1,00
2	1	1	6	0,00	0,00	0,00	0,00	1,00
1	1	1	1	1,00	3,00	0,00	1,00	1,00
1	2	2	2	2,00	2,00	2,00	2,00	3,00
6	8	7	3	2,00	2,00	3,00	1,00	3,00
1	1	1	2	2,00	2,00	1,00	1,00	2,00
1	5	1	2	2,00	2,00	2,00	2,00	2,00
2	2	1	7	3,00	0,00	0,00	1,00	1,00
1	1	1	1	1,00	2,00	1,00	1,00	1,00
1	6	1	6	1,00	2,00	2,00	2,00	1,00

6	9	2	8	2,00	2,00	2,00	1,00	2,00
1	3	1	3	2,00	2,00	2,00	1,00	2,00
1	2	1	1	2,00	1,00	2,00	3,00	3,00
6	3	3	4	0,00	3,00	1,00	1,00	3,00
1	3	2	2	2,00	1,00	3,00	3,00	3,00
1	1	1	1	3,00	1,00	3,00	0,00	3,00
7	6	3	6	1,00	1,00	1,00	0,00	2,00
1	9	1	1	3,00	3,00	3,00	1,00	3,00
4	1	3	3	1,00	1,00	2,00	1,00	1,00
2	4	1	4	1,00	1,00	1,00	0,00	2,00
1	4	1	1	2,00	2,00	2,00	0,00	2,00
2	6	1	5	1,00	1,00	0,00	1,00	1,00
1	1	1	1	3,00	3,00	3,00	1,00	3,00
2	1	1	1	1,00	2,00	1,00	1,00	1,00
1	2	1	1	2,00	2,00	3,00	3,00	3,00
1	1	1	3	3,00	1,00	2,00	2,00	3,00
2	5	2	7	0,00	0,00	0,00	0,00	0,00
6	3	1	1	1,00	2,00	1,00	0,00	1,00
1	1	1	1	1,00	1,00	1,00	1,00	1,00
4	2	1	1	1,00	1,00	0,00	0,00	1,00
1	2	1	1	3,00	3,00	3,00	3,00	3,00
2	1	1	7	0,00	1,00	0,00	0,00	0,00
3	3	3	7	1,00	1,00	0,00	0,00	0,00
1	1	1	1	2,00	3,00	2,00	1,00	2,00
2	2	1	8	0,00	1,00	0,00	0,00	3,00
1	3	1	3	1,00	2,00	1,00	2,00	1,00
2	3	1	2	1,00	1,00	3,00	1,00	2,00
1	1	1	1	1,00	1,00	1,00	3,00	3,00
1	3	1	1	3,00	1,00	2,00	0,00	3,00
6	6	1	5	1,00	2,00	1,00	1,00	1,00
2	2	1	1	2,00	2,00	1,00	1,00	1,00
2	5	1	9	2,00	2,00	1,00	1,00	2,00
2	3	3	7	1,00	2,00	1,00	1,00	1,00
8	7	3	2	1,00	1,00	0,00	2,00	1,00
1	5	1	1	2,00	1,00	0,00	0,00	1,00
2	9	1	2	1,00	2,00	1,00	2,00	1,00
1	5	5	2	0,00	1,00	0,00	0,00	1,00
1	5	1	2	3,00	2,00	2,00	2,00	3,00
2	2	1	3	1,00	2,00	1,00	2,00	1,00
1	2	1	2	1,00	2,00	1,00	0,00	0,00
2	2	1	5	1,00	1,00	1,00	1,00	2,00
6	3	1	1	1,00	2,00	1,00	1,00	2,00
6	6	5	8	0,00	1,00	0,00	0,00	0,00
1	6	1	1	0,00	1,00	1,00	1,00	0,00
1	9	1	9	0,00	1,00	0,00	0,00	1,00
4	8	2	8	1,00	1,00	0,00	1,00	0,00
1	9	1	1	1,00	2,00	0,00	0,00	0,00
1	5	1	1	1,00	2,00	1,00	1,00	2,00
1	1	1	6	0,00	1,00	0,00	0,00	0,00
1	4	5	5	1,00	2,00	1,00	0,00	1,00

2	2	1	2	1,00	2,00	1,00	2,00	3,00
1	2	1	2	2,00	2,00	2,00	2,00	3,00
6	5	5	1	0,00	1,00	0,00	1,00	3,00
1	1	1	1	3,00	3,00	3,00	2,00	3,00
1	1	1	1	1,00	2,00	2,00	0,00	1,00
2	5	1	1	1,00	2,00	1,00	1,00	1,00
1	8	2	8	2,00	2,00	2,00	1,00	1,00
1	2	1	2	3,00	2,00	1,00	2,00	3,00
8	7	1	5	2,00	0,00	1,00	0,00	1,00
7	6	5	1	0,00	1,00	0,00	1,00	0,00
1	8	1	5	2,00	0,00	0,00	0,00	0,00
7	5	5	6	1,00	1,00	1,00	0,00	0,00
6	9	6	8	0,00	1,00	0,00	0,00	0,00
1	3	1	1	1,00	2,00	1,00	2,00	2,00
7	5	2	9	1,00	2,00	2,00	1,00	1,00
6	6	6	5	0,00	2,00	0,00	2,00	2,00
1	1	1	1	3,00	3,00	3,00	3,00	3,00
2	2	2	2	3,00	1,00	1,00	1,00	2,00
1	4	1	2	1,00	2,00	3,00	1,00	3,00
5	9	5	3	1,00	2,00	1,00	1,00	1,00
9	1	9	5	3,00	2,00	3,00	0,00	2,00
3	6	2	6	1,00	1,00	1,00	0,00	1,00
3	7	2	6	0,00	2,00	0,00	0,00	0,00
1	8	2	2	1,00	2,00	2,00	2,00	2,00
1	1	1	1	1,00	2,00	0,00	0,00	1,00
1	1	1	1	1,00	3,00	1,00	1,00	3,00
1	1	1	2	2,00	2,00	2,00	0,00	2,00
1	1	1	1	1,00	1,00	2,00	1,00	1,00
2	1	3	5	0,00	2,00	0,00	1,00	0,00
1	1	1	1	0,00	1,00	3,00	0,00	0,00
1	1	1	9	0,00	2,00	0,00	0,00	1,00

SW01_B	SW03_B	SW04_B	SW07_B	SW10_B	PH01	PH02	PH03	PH04
1,00	2,00	2,00	2,00	3,00	1,00	0,00	0,00	1,00
0,00	2,00	1,00	0,00	0,00	2,00	2,00	3,00	2,00
1,00	2,00	2,00	3,00	2,00	1,00	3,00	0,00	0,00
1,00	2,00	2,00	1,00	1,00	1,00	1,00	2,00	2,00
1,00	2,00	2,00	2,00	1,00	1,00	2,00	1,00	2,00
3,00	3,00	3,00	3,00	3,00	2,00	1,00	1,00	3,00
0,00	1,00	1,00	1,00	0,00	1,00	1,00	2,00	1,00
1,00	3,00	3,00	2,00	1,00	3,00	2,00	3,00	3,00
1,00	2,00	2,00	2,00	1,00	1,00	2,00	3,00	2,00
1,00	3,00	3,00	3,00	1,00	1,00	2,00	1,00	1,00
2,00	2,00	2,00	2,00	1,00	0,00	0,00	1,00	1,00
0,00	1,00	1,00	1,00	0,00	2,00	3,00	3,00	3,00
0,00	0,00	1,00	0,00	0,00	3,00	2,00	2,00	3,00
0,00	1,00	0,00	1,00	0,00	3,00	3,00	3,00	3,00
3,00	3,00	3,00	3,00	3,00	0,00	0,00	1,00	3,00
1,00	1,00	1,00	1,00	1,00	1,00	2,00	1,00	0,00
1,00	2,00	3,00	1,00	0,00	2,00	1,00	1,00	1,00
0,00	1,00	0,00	3,00	0,00	3,00	3,00	3,00	3,00
2,00	3,00	3,00	3,00	1,00	1,00	2,00	1,00	2,00
2,00	3,00	3,00	3,00	3,00	1,00	0,00	0,00	1,00
2,00	2,00	2,00	2,00	1,00	1,00	1,00	1,00	1,00
3,00	3,00	3,00	3,00	2,00	1,00	1,00	2,00	1,00
2,00	3,00	2,00	2,00	3,00	2,00	3,00	3,00	2,00
0,00	1,00	1,00	1,00	0,00	3,00	3,00	3,00	2,00
0,00	2,00	2,00	1,00	0,00	3,00	1,00	2,00	3,00
0,00	1,00	2,00	1,00	1,00	1,00	2,00	3,00	3,00
1,00	2,00	2,00	2,00	1,00	1,00	1,00	0,00	1,00
2,00	2,00	2,00	3,00	2,00	0,00	0,00	0,00	2,00
1,00	1,00	1,00	1,00	1,00	2,00	3,00	3,00	3,00
1,00	2,00	2,00	2,00	1,00	2,00	2,00	1,00	1,00
0,00	2,00	2,00	1,00	0,00	1,00	3,00	3,00	2,00
1,00	2,00	1,00	1,00	1,00	1,00	1,00	2,00	3,00
1,00	2,00	3,00	1,00	1,00	1,00	1,00	2,00	3,00
0,00	0,00	0,00	0,00	0,00	3,00	3,00	1,00	3,00
1,00	2,00	2,00	1,00	1,00	2,00	2,00	3,00	3,00
3,00	2,00	1,00	2,00	1,00	2,00	3,00	3,00	3,00
2,00	3,00	3,00	2,00	2,00	1,00	0,00	2,00	1,00
0,00	2,00	2,00	2,00	0,00	3,00	3,00	3,00	3,00
1,00	1,00	2,00	1,00	1,00	0,00	1,00	0,00	1,00
2,00	2,00	1,00	1,00	1,00	1,00	1,00	3,00	2,00
1,00	1,00	1,00	1,00	1,00	1,00	2,00	0,00	2,00
3,00	3,00	3,00	2,00	2,00	1,00	1,00	2,00	1,00
3,00	2,00	2,00	3,00	3,00	0,00	0,00	1,00	1,00
1,00	3,00	3,00	2,00	3,00	1,00	2,00	2,00	2,00
2,00	3,00	2,00	3,00	1,00	1,00	1,00	3,00	1,00
2,00	2,00	2,00	2,00	1,00	1,00	0,00	1,00	1,00
2,00	3,00	3,00	2,00	2,00	0,00	0,00	1,00	1,00
0,00	2,00	1,00	1,00	1,00	3,00	2,00	3,00	3,00
1,00	3,00	3,00	1,00	1,00	1,00	3,00	3,00	1,00

1,00	2,00	2,00	2,00	2,00	1,00	1,00	3,00	2,00
1,00	3,00	3,00	3,00	1,00	1,00	3,00	3,00	3,00
0,00	2,00	1,00	0,00	0,00	3,00	3,00	3,00	3,00
0,00	1,00	1,00	0,00	1,00	3,00	3,00	3,00	3,00
2,00	3,00	3,00	3,00	3,00	0,00	0,00	0,00	1,00
1,00	2,00	2,00	2,00	2,00	1,00	1,00	1,00	2,00
2,00	2,00	3,00	3,00	1,00	1,00	1,00	3,00	3,00
1,00	2,00	3,00	3,00	2,00	1,00	1,00	2,00	2,00
1,00	2,00	1,00	2,00	2,00	2,00	1,00	3,00	1,00
0,00	2,00	3,00	0,00	0,00	2,00	3,00	3,00	3,00
0,00	1,00	1,00	0,00	0,00	2,00	1,00	2,00	3,00
2,00	3,00	3,00	2,00	1,00	3,00	2,00	1,00	3,00
2,00	2,00	3,00	2,00	2,00	1,00	1,00	1,00	2,00
2,00	3,00	2,00	3,00	3,00	2,00	3,00	2,00	2,00
2,00	0,00	0,00	0,00	0,00	3,00	2,00	3,00	2,00
1,00	2,00	3,00	2,00	1,00	1,00	3,00	3,00	0,00
2,00	2,00	2,00	2,00	2,00	1,00	0,00	0,00	1,00
1,00	2,00	1,00	0,00	1,00	2,00	2,00	2,00	1,00
0,00	1,00	1,00	0,00	1,00	1,00	1,00	3,00	3,00
1,00	3,00	2,00	3,00	2,00	1,00	1,00	2,00	1,00
1,00	2,00	2,00	2,00	1,00	3,00	3,00	3,00	3,00
3,00	3,00	3,00	3,00	2,00	0,00	0,00	0,00	1,00
2,00	3,00	2,00	2,00	3,00	1,00	0,00	2,00	2,00
1,00	1,00	1,00	1,00	2,00	1,00	1,00	2,00	3,00
2,00	2,00	2,00	2,00	1,00	3,00	2,00	2,00	3,00
2,00	3,00	3,00	3,00	2,00	1,00	1,00	0,00	2,00
0,00	1,00	1,00	0,00	0,00	3,00	3,00	3,00	3,00
0,00	0,00	0,00	3,00	0,00	2,00	3,00	2,00	3,00
3,00	3,00	3,00	3,00	3,00	1,00	1,00	0,00	0,00
2,00	2,00	2,00	3,00	2,00	1,00	1,00	3,00	2,00
1,00	2,00	1,00	1,00	1,00	2,00	1,00	2,00	1,00
1,00	2,00	2,00	1,00	0,00	1,00	1,00	3,00	2,00
1,00	1,00	0,00	1,00	0,00	3,00	3,00	3,00	3,00
2,00	2,00	2,00	2,00	1,00	1,00	0,00	1,00	1,00
1,00	2,00	2,00	1,00	1,00	2,00	2,00	3,00	3,00
3,00	3,00	3,00	3,00	3,00	0,00	0,00	1,00	1,00
2,00	3,00	3,00	3,00	2,00	1,00	0,00	1,00	1,00
0,00	1,00	1,00	0,00	0,00	3,00	3,00	3,00	3,00
2,00	3,00	2,00	1,00	2,00	1,00	1,00	3,00	2,00
1,00	2,00	1,00	1,00	1,00	1,00	1,00	1,00	2,00
0,00	1,00	1,00	0,00	0,00	1,00	1,00	1,00	2,00
1,00	2,00	2,00	2,00	2,00	2,00	2,00	0,00	1,00
1,00	2,00	2,00	1,00	1,00	2,00	1,00	3,00	3,00
1,00	2,00	1,00	1,00	1,00	1,00	1,00	2,00	1,00
1,00	2,00	2,00	2,00	1,00	2,00	2,00	3,00	3,00
0,00	2,00	1,00	1,00	0,00	2,00	2,00	2,00	3,00
1,00	2,00	1,00	1,00	1,00	2,00	2,00	3,00	3,00
1,00	2,00	1,00	1,00	0,00	2,00	3,00	1,00	3,00
0,00	0,00	0,00	0,00	0,00	2,00	0,00	3,00	3,00
0,00	0,00	1,00	0,00	0,00	2,00	1,00	2,00	3,00

2,00	2,00	2,00	0,00	1,00	1,00	1,00	1,00	1,00
2,00	2,00	2,00	2,00	1,00	2,00	1,00	3,00	3,00
1,00	3,00	2,00	3,00	1,00	3,00	3,00	3,00	3,00
0,00	1,00	1,00	1,00	0,00	2,00	2,00	3,00	3,00
1,00	3,00	3,00	3,00	2,00	1,00	2,00	2,00	1,00
2,00	2,00	2,00	2,00	1,00	2,00	1,00	0,00	1,00
1,00	2,00	1,00	2,00	2,00	2,00	2,00	3,00	3,00
1,00	3,00	2,00	1,00	2,00	1,00	2,00	1,00	2,00
1,00	1,00	1,00	1,00	0,00	2,00	3,00	1,00	3,00
2,00	3,00	2,00	3,00	2,00	1,00	1,00	2,00	1,00
1,00	1,00	1,00	0,00	1,00	3,00	2,00	3,00	3,00
2,00	1,00	1,00	1,00	1,00	3,00	2,00	2,00	3,00
3,00	2,00	2,00	2,00	2,00	0,00	0,00	1,00	1,00
2,00	2,00	3,00	1,00	2,00	1,00	1,00	2,00	2,00
0,00	0,00	0,00	0,00	0,00	1,00	3,00	3,00	1,00
0,00	3,00	3,00	2,00	1,00	3,00	2,00	3,00	3,00
1,00	2,00	2,00	2,00	2,00	1,00	1,00	1,00	2,00
1,00	2,00	2,00	2,00	2,00	2,00	1,00	1,00	1,00
1,00	3,00	3,00	3,00	2,00	0,00	1,00	0,00	1,00
2,00	2,00	3,00	3,00	1,00	3,00	3,00	2,00	3,00
2,00	3,00	3,00	2,00	2,00	3,00	1,00	1,00	3,00
2,00	3,00	2,00	2,00	2,00	2,00	3,00	3,00	3,00
1,00	2,00	3,00	1,00	0,00	2,00	3,00	3,00	3,00
1,00	3,00	3,00	2,00	0,00	3,00	2,00	3,00	3,00
0,00	2,00	1,00	1,00	0,00	2,00	3,00	2,00	3,00
0,00	1,00	1,00	1,00	1,00	2,00	3,00	3,00	3,00
1,00	1,00	0,00	0,00	1,00	3,00	3,00	2,00	1,00
1,00	2,00	3,00	2,00	2,00	1,00	1,00	0,00	3,00
2,00	3,00	1,00	3,00	2,00	1,00	0,00	1,00	1,00
1,00	0,00	0,00	0,00	0,00	3,00	3,00	3,00	3,00
0,00	1,00	0,00	1,00	1,00	1,00	1,00	1,00	2,00
2,00	2,00	2,00	2,00	2,00	1,00	1,00	3,00	1,00
3,00	3,00	2,00	3,00	2,00	1,00	1,00	0,00	1,00
3,00	3,00	3,00	3,00	3,00	0,00	0,00	1,00	0,00
1,00	2,00	2,00	2,00	1,00	1,00	1,00	2,00	2,00
0,00	1,00	1,00	0,00	1,00	1,00	1,00	1,00	1,00
3,00	3,00	3,00	3,00	3,00	0,00	0,00	0,00	1,00
3,00	3,00	3,00	2,00	2,00	0,00	0,00	1,00	1,00
0,00	1,00	2,00	0,00	0,00	2,00	2,00	3,00	2,00
2,00	3,00	2,00	3,00	2,00	1,00	1,00	2,00	1,00
1,00	2,00	1,00	2,00	1,00	1,00	1,00	2,00	3,00
2,00	3,00	2,00	3,00	2,00	0,00	0,00	1,00	0,00
2,00	3,00	1,00	0,00	2,00	1,00	2,00	1,00	3,00
0,00	1,00	0,00	0,00	0,00	2,00	2,00	3,00	3,00
0,00	2,00	2,00	1,00	1,00	2,00	2,00	2,00	2,00
0,00	2,00	1,00	0,00	0,00	1,00	3,00	3,00	2,00
0,00	3,00	3,00	2,00	0,00	3,00	3,00	3,00	2,00
1,00	2,00	2,00	1,00	1,00	3,00	2,00	2,00	3,00
1,00	2,00	2,00	2,00	2,00	2,00	0,00	0,00	1,00
2,00	3,00	3,00	3,00	2,00	1,00	1,00	3,00	3,00

2,00	3,00	2,00	2,00	2,00	1,00	1,00	1,00	1,00
2,00	2,00	3,00	0,00	1,00	0,00	0,00	3,00	1,00
2,00	2,00	2,00	2,00	2,00	1,00	1,00	0,00	1,00
1,00	2,00	1,00	0,00	1,00	0,00	1,00	3,00	1,00
2,00	3,00	2,00	3,00	3,00	2,00	2,00	2,00	2,00
1,00	2,00	1,00	1,00	1,00	2,00	1,00	1,00	2,00
2,00	3,00	3,00	2,00	2,00	0,00	0,00	0,00	1,00
0,00	1,00	1,00	1,00	1,00	2,00	3,00	3,00	3,00
2,00	2,00	3,00	3,00	2,00	1,00	1,00	2,00	3,00
2,00	2,00	2,00	2,00	2,00	1,00	1,00	1,00	2,00
1,00	3,00	1,00	2,00	1,00	1,00	1,00	0,00	1,00
1,00	1,00	2,00	2,00	0,00	2,00	2,00	1,00	1,00
2,00	3,00	3,00	1,00	2,00	1,00	0,00	2,00	1,00
2,00	2,00	2,00	3,00	1,00	2,00	2,00	2,00	3,00
1,00	3,00	1,00	2,00	1,00	1,00	2,00	2,00	2,00
0,00	1,00	1,00	0,00	0,00	3,00	3,00	3,00	2,00
1,00	3,00	2,00	2,00	0,00	2,00	1,00	1,00	3,00
3,00	2,00	2,00	2,00	1,00	3,00	3,00	2,00	3,00
1,00	2,00	1,00	1,00	1,00	3,00	1,00	1,00	1,00
0,00	0,00	0,00	0,00	0,00	2,00	3,00	1,00	3,00
0,00	2,00	0,00	1,00	0,00	1,00	2,00	1,00	3,00
2,00	2,00	2,00	1,00	1,00	1,00	1,00	1,00	3,00
0,00	1,00	1,00	1,00	3,00	1,00	3,00	3,00	3,00
0,00	2,00	1,00	1,00	1,00	1,00	1,00	1,00	2,00
1,00	2,00	1,00	1,00	1,00	3,00	2,00	2,00	2,00
3,00	3,00	3,00	3,00	1,00	3,00	3,00	1,00	2,00
1,00	1,00	3,00	1,00	1,00	1,00	2,00	1,00	3,00
1,00	3,00	2,00	1,00	0,00	3,00	2,00	3,00	3,00
1,00	2,00	1,00	1,00	1,00	3,00	1,00	3,00	3,00
0,00	3,00	3,00	3,00	1,00	1,00	3,00	3,00	3,00
2,00	3,00	2,00	3,00	3,00	0,00	1,00	1,00	1,00
0,00	1,00	1,00	1,00	1,00	2,00	1,00	3,00	3,00
1,00	1,00	1,00	0,00	1,00	2,00	1,00	1,00	3,00
1,00	2,00	1,00	2,00	2,00	1,00	1,00	0,00	2,00
2,00	1,00	2,00	2,00	2,00	1,00	1,00	2,00	2,00
2,00	2,00	2,00	1,00	2,00	1,00	0,00	1,00	1,00
1,00	2,00	2,00	1,00	1,00	1,00	1,00	2,00	3,00
0,00	0,00	1,00	0,00	0,00	3,00	2,00	1,00	2,00
1,00	2,00	2,00	0,00	1,00	3,00	2,00	0,00	2,00
2,00	2,00	2,00	1,00	1,00	2,00	2,00	2,00	3,00
2,00	2,00	1,00	2,00	2,00	1,00	1,00	1,00	2,00
1,00	2,00	1,00	2,00	1,00	1,00	1,00	3,00	2,00
0,00	1,00	3,00	1,00	2,00	3,00	3,00	2,00	3,00
0,00	1,00	2,00	1,00	0,00	3,00	2,00	3,00	3,00
0,00	1,00	1,00	0,00	0,00	3,00	3,00	1,00	3,00
2,00	3,00	3,00	3,00	2,00	3,00	2,00	1,00	3,00
0,00	3,00	3,00	3,00	1,00	3,00	3,00	2,00	2,00
1,00	1,00	1,00	1,00	1,00	2,00	2,00	3,00	3,00
2,00	3,00	1,00	2,00	1,00	2,00	1,00	3,00	3,00
0,00	1,00	0,00	0,00	0,00	2,00	3,00	3,00	3,00

0,00	0,00	0,00	0,00	0,00	3,00	3,00	3,00	3,00
1,00	1,00	1,00	1,00	1,00	2,00	3,00	2,00	2,00
0,00	2,00	1,00	2,00	0,00	2,00	3,00	3,00	3,00
1,00	2,00	2,00	1,00	1,00	1,00	1,00	1,00	3,00
0,00	0,00	1,00	1,00	0,00	1,00	3,00	2,00	3,00
0,00	1,00	0,00	1,00	0,00	2,00	1,00	3,00	2,00
1,00	2,00	2,00	1,00	0,00	1,00	2,00	3,00	3,00
1,00	2,00	2,00	1,00	1,00	1,00	1,00	2,00	1,00
1,00	2,00	1,00	1,00	1,00	2,00	2,00	3,00	3,00
2,00	1,00	1,00	1,00	0,00	3,00	1,00	3,00	3,00
1,00	2,00	0,00	0,00	0,00	3,00	3,00	3,00	3,00
2,00	2,00	3,00	3,00	2,00	1,00	1,00	1,00	2,00
0,00	1,00	0,00	1,00	0,00	3,00	2,00	3,00	3,00
1,00	1,00	2,00	0,00	0,00	2,00	1,00	3,00	3,00
0,00	0,00	0,00	0,00	0,00	2,00	3,00	2,00	3,00
0,00	2,00	2,00	1,00	0,00	3,00	3,00	3,00	3,00
1,00	2,00	2,00	1,00	1,00	2,00	1,00	2,00	2,00
1,00	2,00	2,00	1,00	1,00	2,00	2,00	2,00	2,00
3,00	3,00	3,00	3,00	3,00	1,00	1,00	3,00	2,00
0,00	0,00	1,00	1,00	0,00	2,00	2,00	3,00	2,00
3,00	3,00	3,00	3,00	2,00	0,00	1,00	1,00	1,00
0,00	1,00	2,00	0,00	0,00	3,00	3,00	3,00	3,00
0,00	1,00	1,00	0,00	1,00	1,00	2,00	3,00	3,00
1,00	2,00	1,00	1,00	1,00	2,00	1,00	2,00	2,00
1,00	1,00	2,00	2,00	1,00	1,00	0,00	1,00	1,00
1,00	2,00	1,00	1,00	1,00	2,00	2,00	3,00	3,00
0,00	2,00	2,00	3,00	0,00	3,00	3,00	3,00	2,00
1,00	1,00	1,00	1,00	0,00	2,00	3,00	3,00	3,00
0,00	1,00	1,00	1,00	0,00	2,00	1,00	3,00	3,00
1,00	2,00	1,00	2,00	0,00	3,00	3,00	3,00	3,00
1,00	2,00	1,00	2,00	2,00	2,00	2,00	3,00	2,00
2,00	2,00	3,00	2,00	2,00	0,00	0,00	1,00	1,00
2,00	2,00	3,00	3,00	2,00	1,00	1,00	2,00	1,00
2,00	2,00	2,00	2,00	2,00	1,00	0,00	1,00	1,00
0,00	1,00	0,00	0,00	0,00	3,00	3,00	3,00	3,00
1,00	2,00	1,00	1,00	1,00	1,00	2,00	3,00	1,00
1,00	2,00	2,00	2,00	1,00	2,00	2,00	3,00	3,00
2,00	1,00	1,00	2,00	0,00	1,00	1,00	1,00	1,00
1,00	1,00	2,00	1,00	1,00	2,00	1,00	2,00	3,00
1,00	2,00	2,00	1,00	0,00	3,00	3,00	3,00	3,00
0,00	1,00	0,00	1,00	0,00	1,00	3,00	3,00	3,00
1,00	1,00	1,00	1,00	0,00	1,00	2,00	1,00	2,00
0,00	2,00	1,00	2,00	2,00	1,00	1,00	2,00	2,00
2,00	3,00	1,00	3,00	2,00	1,00	1,00	3,00	3,00
1,00	2,00	2,00	2,00	1,00	3,00	3,00	3,00	3,00
1,00	3,00	3,00	3,00	1,00	2,00	3,00	3,00	3,00
0,00	1,00	1,00	1,00	0,00	3,00	3,00	3,00	3,00
2,00	1,00	2,00	2,00	1,00	1,00	1,00	1,00	1,00
1,00	1,00	2,00	2,00	1,00	2,00	1,00	1,00	2,00
2,00	1,00	0,00	1,00	1,00	1,00	3,00	2,00	3,00

1,00	1,00	1,00	1,00	2,00	1,00	1,00	2,00	1,00
2,00	3,00	3,00	3,00	2,00	3,00	3,00	3,00	2,00
2,00	2,00	2,00	2,00	3,00	1,00	1,00	3,00	3,00
2,00	3,00	3,00	3,00	2,00	0,00	0,00	1,00	1,00
2,00	3,00	3,00	3,00	2,00	3,00	3,00	3,00	3,00
2,00	2,00	2,00	2,00	1,00	3,00	3,00	2,00	3,00
1,00	2,00	2,00	2,00	0,00	1,00	1,00	2,00	3,00
1,00	3,00	1,00	1,00	1,00	2,00	3,00	2,00	3,00
1,00	1,00	1,00	0,00	0,00	1,00	1,00	2,00	2,00
2,00	2,00	2,00	1,00	1,00	1,00	1,00	2,00	2,00
0,00	1,00	0,00	0,00	0,00	2,00	3,00	3,00	2,00
2,00	3,00	3,00	2,00	2,00	1,00	0,00	1,00	1,00
0,00	2,00	2,00	2,00	1,00	2,00	1,00	3,00	2,00
0,00	2,00	0,00	1,00	0,00	2,00	2,00	3,00	3,00
0,00	0,00	0,00	0,00	0,00	2,00	2,00	2,00	2,00
0,00	2,00	1,00	1,00	1,00	2,00	2,00	1,00	2,00
2,00	2,00	1,00	1,00	2,00	0,00	0,00	2,00	1,00
1,00	2,00	2,00	2,00	1,00	2,00	1,00	1,00	1,00
1,00	1,00	1,00	1,00	1,00	2,00	2,00	1,00	2,00
1,00	3,00	2,00	1,00	2,00	1,00	1,00	3,00	3,00
2,00	2,00	2,00	2,00	1,00	0,00	0,00	0,00	0,00
0,00	0,00	0,00	0,00	0,00	3,00	3,00	3,00	3,00
1,00	2,00	2,00	1,00	0,00	2,00	2,00	2,00	3,00
1,00	2,00	2,00	1,00	2,00	0,00	0,00	2,00	2,00
1,00	2,00	2,00	1,00	1,00	2,00	1,00	3,00	1,00
1,00	3,00	3,00	3,00	1,00	3,00	3,00	3,00	2,00
1,00	2,00	0,00	1,00	0,00	3,00	3,00	2,00	3,00
1,00	1,00	1,00	1,00	1,00	1,00	2,00	2,00	3,00
1,00	2,00	3,00	3,00	1,00	1,00	1,00	3,00	2,00
2,00	2,00	2,00	2,00	1,00	1,00	1,00	1,00	3,00
1,00	2,00	2,00	2,00	1,00	3,00	3,00	3,00	3,00
1,00	1,00	1,00	1,00	0,00	2,00	1,00	2,00	2,00
3,00	1,00	0,00	2,00	1,00	2,00	1,00	3,00	1,00
0,00	2,00	2,00	1,00	0,00	1,00	2,00	2,00	2,00
0,00	1,00	0,00	0,00	1,00	3,00	3,00	3,00	3,00
2,00	2,00	2,00	1,00	1,00	1,00	1,00	2,00	1,00
2,00	3,00	1,00	3,00	2,00	3,00	3,00	3,00	3,00
2,00	3,00	1,00	3,00	2,00	2,00	3,00	3,00	3,00
1,00	2,00	2,00	2,00	1,00	2,00	2,00	1,00	1,00
1,00	2,00	1,00	1,00	1,00	1,00	3,00	3,00	1,00
1,00	3,00	3,00	3,00	2,00	1,00	1,00	1,00	1,00
1,00	3,00	0,00	0,00	1,00	2,00	3,00	3,00	2,00
0,00	1,00	1,00	1,00	0,00	2,00	2,00	3,00	3,00
2,00	3,00	3,00	3,00	2,00	3,00	3,00	1,00	3,00
2,00	3,00	3,00	3,00	2,00	1,00	0,00	1,00	0,00
1,00	2,00	1,00	2,00	1,00	3,00	3,00	3,00	3,00
0,00	0,00	0,00	0,00	0,00	3,00	3,00	3,00	3,00
1,00	2,00	1,00	1,00	1,00	2,00	2,00	1,00	2,00
1,00	2,00	0,00	2,00	1,00	2,00	2,00	3,00	3,00
0,00	1,00	2,00	0,00	0,00	2,00	3,00	1,00	2,00

1,00	2,00	2,00	1,00	1,00	1,00	1,00	2,00	1,00
1,00	2,00	2,00	1,00	0,00	2,00	3,00	3,00	3,00
1,00	1,00	0,00	1,00	0,00	2,00	3,00	3,00	3,00
2,00	2,00	1,00	2,00	2,00	3,00	3,00	3,00	3,00
0,00	2,00	2,00	2,00	0,00	1,00	1,00	2,00	3,00
1,00	2,00	0,00	2,00	2,00	0,00	1,00	1,00	0,00
3,00	2,00	1,00	1,00	1,00	1,00	1,00	2,00	3,00
0,00	1,00	0,00	1,00	1,00	0,00	3,00	2,00	1,00
2,00	3,00	2,00	2,00	2,00	2,00	3,00	2,00	3,00
1,00	3,00	3,00	2,00	2,00	2,00	2,00	3,00	3,00
1,00	1,00	2,00	1,00	1,00	3,00	2,00	2,00	2,00
1,00	2,00	2,00	2,00	2,00	1,00	1,00	3,00	2,00
1,00	1,00	0,00	1,00	0,00	1,00	1,00	1,00	2,00
1,00	2,00	1,00	1,00	1,00	3,00	1,00	2,00	3,00
0,00	1,00	1,00	1,00	1,00	2,00	3,00	2,00	3,00
1,00	2,00	2,00	0,00	1,00	2,00	2,00	1,00	2,00
1,00	0,00	0,00	0,00	0,00	3,00	1,00	2,00	3,00
1,00	3,00	2,00	2,00	1,00	0,00	1,00	0,00	1,00
3,00	2,00	2,00	3,00	3,00	1,00	1,00	1,00	1,00
0,00	1,00	1,00	2,00	0,00	3,00	2,00	2,00	3,00
2,00	3,00	3,00	2,00	2,00	1,00	2,00	2,00	3,00
2,00	3,00	3,00	3,00	2,00	1,00	1,00	1,00	1,00
1,00	1,00	1,00	2,00	1,00	3,00	1,00	2,00	3,00
1,00	2,00	2,00	1,00	1,00	2,00	1,00	3,00	3,00
0,00	1,00	2,00	1,00	0,00	3,00	3,00	3,00	3,00
0,00	2,00	2,00	1,00	0,00	3,00	3,00	2,00	3,00
0,00	0,00	0,00	0,00	0,00	3,00	3,00	3,00	3,00
1,00	3,00	2,00	3,00	2,00	3,00	3,00	2,00	2,00
1,00	2,00	1,00	1,00	1,00	1,00	1,00	2,00	1,00
0,00	1,00	0,00	0,00	0,00	2,00	3,00	3,00	3,00
1,00	3,00	2,00	3,00	2,00	1,00	1,00	2,00	3,00
0,00	1,00	1,00	1,00	1,00	1,00	1,00	3,00	1,00
1,00	2,00	2,00	2,00	1,00	2,00	3,00	3,00	3,00
1,00	2,00	2,00	1,00	1,00	2,00	2,00	3,00	3,00
2,00	1,00	1,00	1,00	2,00	2,00	3,00	3,00	3,00
1,00	3,00	2,00	1,00	1,00	1,00	1,00	1,00	2,00
1,00	2,00	2,00	2,00	0,00	3,00	3,00	2,00	2,00
0,00	0,00	0,00	0,00	0,00	3,00	3,00	2,00	3,00
0,00	1,00	1,00	0,00	0,00	3,00	3,00	3,00	3,00
1,00	2,00	1,00	1,00	0,00	2,00	3,00	1,00	3,00
1,00	2,00	2,00	1,00	1,00	3,00	3,00	1,00	3,00
2,00	2,00	1,00	2,00	1,00	1,00	0,00	1,00	2,00
1,00	1,00	1,00	1,00	1,00	2,00	1,00	3,00	3,00
1,00	1,00	3,00	3,00	1,00	1,00	2,00	1,00	3,00
0,00	1,00	1,00	0,00	0,00	3,00	3,00	2,00	3,00
0,00	2,00	1,00	2,00	1,00	3,00	3,00	3,00	3,00
1,00	2,00	2,00	0,00	2,00	1,00	1,00	3,00	1,00
1,00	1,00	1,00	1,00	1,00	2,00	1,00	2,00	1,00
0,00	1,00	1,00	1,00	0,00	3,00	3,00	2,00	3,00
0,00	1,00	0,00	0,00	0,00	1,00	1,00	3,00	2,00

1,00	3,00	2,00	2,00	2,00	1,00	1,00	1,00	1,00
1,00	1,00	1,00	1,00	1,00	2,00	3,00	2,00	3,00
2,00	1,00	2,00	1,00	1,00	2,00	2,00	3,00	3,00
3,00	3,00	2,00	3,00	2,00	1,00	1,00	3,00	1,00
0,00	1,00	1,00	1,00	1,00	2,00	2,00	0,00	3,00
1,00	2,00	2,00	2,00	0,00	1,00	0,00	3,00	3,00
0,00	0,00	0,00	0,00	0,00	3,00	3,00	3,00	3,00
3,00	3,00	3,00	3,00	3,00	0,00	0,00	1,00	1,00
2,00	2,00	2,00	2,00	2,00	1,00	2,00	1,00	2,00
1,00	3,00	3,00	2,00	2,00	3,00	3,00	2,00	3,00
1,00	1,00	1,00	1,00	2,00	1,00	1,00	3,00	3,00
1,00	2,00	2,00	2,00	1,00	1,00	3,00	1,00	2,00
1,00	1,00	0,00	2,00	2,00	1,00	1,00	1,00	3,00
1,00	2,00	1,00	1,00	0,00	2,00	1,00	2,00	2,00
3,00	3,00	3,00	1,00	2,00	1,00	1,00	2,00	1,00
1,00	3,00	3,00	3,00	1,00	2,00	2,00	2,00	3,00
0,00	2,00	2,00	2,00	2,00	2,00	1,00	1,00	1,00
0,00	0,00	0,00	0,00	1,00	3,00	2,00	1,00	3,00
1,00	1,00	1,00	1,00	1,00	1,00	2,00	2,00	3,00
1,00	1,00	1,00	1,00	0,00	2,00	2,00	1,00	3,00
0,00	1,00	1,00	1,00	0,00	2,00	2,00	3,00	3,00
0,00	1,00	1,00	0,00	0,00	3,00	3,00	3,00	3,00
1,00	1,00	1,00	1,00	1,00	2,00	2,00	3,00	1,00
1,00	3,00	3,00	3,00	1,00	2,00	2,00	1,00	3,00
0,00	1,00	1,00	1,00	1,00	3,00	1,00	3,00	3,00
0,00	1,00	2,00	1,00	1,00	2,00	2,00	3,00	2,00
2,00	3,00	3,00	2,00	0,00	1,00	1,00	2,00	2,00
1,00	0,00	1,00	1,00	0,00	3,00	2,00	3,00	3,00
1,00	3,00	3,00	3,00	1,00	1,00	0,00	1,00	3,00
0,00	0,00	0,00	0,00	0,00	3,00	3,00	3,00	3,00
1,00	2,00	2,00	3,00	1,00	2,00	2,00	2,00	3,00
0,00	1,00	1,00	1,00	0,00	3,00	3,00	3,00	3,00
2,00	2,00	3,00	0,00	0,00	2,00	2,00	3,00	3,00
2,00	2,00	1,00	2,00	2,00	1,00	1,00	3,00	1,00
0,00	0,00	0,00	0,00	0,00	2,00	2,00	2,00	3,00
1,00	1,00	1,00	1,00	1,00	1,00	1,00	1,00	1,00
1,00	1,00	1,00	1,00	1,00	0,00	0,00	0,00	3,00
3,00	3,00	3,00	3,00	3,00	1,00	0,00	1,00	1,00
0,00	1,00	2,00	1,00	0,00	3,00	3,00	2,00	2,00
0,00	2,00	0,00	1,00	1,00	3,00	2,00	2,00	2,00
2,00	3,00	1,00	3,00	2,00	3,00	3,00	3,00	3,00
2,00	3,00	3,00	2,00	2,00	3,00	3,00	2,00	3,00
0,00	1,00	1,00	0,00	0,00	3,00	2,00	3,00	3,00
2,00	2,00	2,00	2,00	2,00	1,00	1,00	3,00	2,00
2,00	3,00	3,00	2,00	2,00	1,00	1,00	1,00	1,00
0,00	0,00	0,00	0,00	0,00	3,00	3,00	2,00	3,00
0,00	2,00	1,00	0,00	3,00	2,00	2,00	3,00	3,00
1,00	2,00	1,00	1,00	0,00	1,00	2,00	2,00	3,00
0,00	1,00	1,00	1,00	0,00	2,00	2,00	3,00	3,00
0,00	1,00	1,00	1,00	1,00	1,00	2,00	3,00	3,00

0,00	1,00	1,00	0,00	0,00	3,00	3,00	3,00	3,00
2,00	3,00	2,00	3,00	3,00	1,00	1,00	3,00	1,00
2,00	2,00	2,00	2,00	2,00	1,00	1,00	1,00	1,00
0,00	1,00	1,00	3,00	0,00	3,00	3,00	3,00	3,00
0,00	1,00	3,00	0,00	0,00	3,00	3,00	3,00	2,00
0,00	1,00	1,00	1,00	0,00	2,00	3,00	3,00	3,00
1,00	3,00	3,00	3,00	1,00	3,00	1,00	3,00	3,00
0,00	1,00	2,00	3,00	0,00	3,00	3,00	3,00	3,00
3,00	3,00	1,00	3,00	3,00	3,00	3,00	3,00	3,00
0,00	1,00	1,00	0,00	0,00	3,00	3,00	0,00	3,00
2,00	2,00	3,00	3,00	3,00	3,00	2,00	1,00	2,00
3,00	3,00	3,00	3,00	3,00	0,00	0,00	0,00	1,00
2,00	2,00	2,00	2,00	1,00	2,00	3,00	2,00	2,00
1,00	3,00	0,00	0,00	0,00	2,00	1,00	2,00	3,00
2,00	3,00	1,00	3,00	2,00	0,00	0,00	1,00	1,00
3,00	3,00	3,00	3,00	3,00	0,00	1,00	1,00	1,00
2,00	2,00	2,00	2,00	2,00	3,00	2,00	2,00	3,00
1,00	1,00	1,00	2,00	1,00	2,00	1,00	2,00	1,00
0,00	1,00	0,00	0,00	0,00	2,00	2,00	3,00	3,00
0,00	2,00	1,00	1,00	2,00	3,00	3,00	0,00	3,00
0,00	1,00	1,00	1,00	0,00	2,00	2,00	1,00	3,00
2,00	2,00	2,00	3,00	2,00	1,00	1,00	1,00	0,00
1,00	2,00	3,00	2,00	2,00	3,00	2,00	0,00	3,00
1,00	2,00	2,00	2,00	1,00	3,00	2,00	3,00	2,00
0,00	1,00	1,00	0,00	0,00	2,00	3,00	1,00	2,00
1,00	3,00	3,00	3,00	3,00	1,00	0,00	0,00	1,00
1,00	2,00	1,00	1,00	1,00	1,00	1,00	2,00	1,00
3,00	3,00	3,00	3,00	3,00	0,00	0,00	0,00	0,00
1,00	2,00	1,00	1,00	1,00	2,00	2,00	2,00	2,00
2,00	3,00	3,00	2,00	1,00	1,00	1,00	2,00	2,00
2,00	3,00	2,00	3,00	2,00	0,00	1,00	2,00	1,00
1,00	0,00	0,00	0,00	0,00	2,00	0,00	3,00	1,00
3,00	3,00	3,00	3,00	3,00	2,00	1,00	1,00	3,00
0,00	0,00	0,00	0,00	0,00	3,00	3,00	3,00	3,00
1,00	2,00	2,00	1,00	1,00	1,00	1,00	1,00	2,00
0,00	0,00	1,00	0,00	0,00	2,00	2,00	3,00	3,00
1,00	2,00	2,00	2,00	1,00	1,00	1,00	1,00	1,00
0,00	0,00	0,00	0,00	0,00	3,00	3,00	3,00	3,00
2,00	1,00	1,00	1,00	1,00	1,00	1,00	3,00	3,00
2,00	3,00	3,00	2,00	2,00	1,00	1,00	1,00	1,00
1,00	2,00	1,00	1,00	1,00	2,00	2,00	3,00	1,00
1,00	2,00	2,00	2,00	1,00	1,00	1,00	1,00	1,00
2,00	3,00	2,00	3,00	2,00	1,00	1,00	2,00	2,00
0,00	2,00	3,00	2,00	0,00	3,00	2,00	1,00	2,00
2,00	3,00	3,00	3,00	3,00	2,00	2,00	2,00	3,00
0,00	1,00	0,00	0,00	0,00	3,00	3,00	3,00	3,00
2,00	3,00	3,00	2,00	2,00	1,00	1,00	1,00	2,00
1,00	3,00	3,00	1,00	0,00	3,00	1,00	1,00	3,00
2,00	1,00	1,00	1,00	1,00	3,00	3,00	2,00	3,00
1,00	1,00	1,00	1,00	1,00	2,00	2,00	3,00	3,00

0,00	0,00	0,00	0,00	0,00	2,00	3,00	1,00	3,00
0,00	0,00	1,00	1,00	0,00	3,00	3,00	3,00	3,00
0,00	0,00	0,00	0,00	0,00	3,00	3,00	3,00	3,00
1,00	2,00	2,00	1,00	1,00	2,00	1,00	1,00	3,00
1,00	3,00	3,00	3,00	3,00	3,00	2,00	0,00	3,00
0,00	2,00	2,00	1,00	1,00	2,00	3,00	3,00	3,00
0,00	2,00	2,00	2,00	1,00	3,00	2,00	3,00	3,00
2,00	3,00	3,00	2,00	2,00	2,00	2,00	1,00	3,00
0,00	1,00	1,00	0,00	0,00	3,00	3,00	3,00	3,00
1,00	2,00	1,00	2,00	2,00	1,00	0,00	0,00	3,00
1,00	3,00	3,00	0,00	2,00	3,00	3,00	0,00	2,00
2,00	1,00	1,00	2,00	1,00	1,00	1,00	0,00	1,00
0,00	1,00	1,00	0,00	0,00	2,00	3,00	3,00	3,00
1,00	2,00	1,00	1,00	1,00	2,00	3,00	3,00	3,00
0,00	3,00	3,00	1,00	0,00	3,00	3,00	3,00	3,00
1,00	2,00	2,00	1,00	1,00	2,00	3,00	2,00	2,00
1,00	1,00	1,00	0,00	0,00	3,00	2,00	3,00	3,00
1,00	1,00	1,00	0,00	0,00	2,00	1,00	3,00	3,00
1,00	2,00	1,00	2,00	0,00	3,00	2,00	3,00	3,00
0,00	1,00	3,00	3,00	0,00	2,00	3,00	1,00	3,00
2,00	2,00	2,00	3,00	2,00	1,00	0,00	2,00	1,00
0,00	1,00	2,00	0,00	0,00	3,00	2,00	2,00	1,00
2,00	2,00	3,00	1,00	1,00	2,00	2,00	0,00	3,00
0,00	2,00	3,00	0,00	0,00	3,00	3,00	3,00	3,00
0,00	0,00	0,00	0,00	0,00	3,00	3,00	1,00	3,00
1,00	1,00	1,00	2,00	1,00	3,00	2,00	1,00	2,00
2,00	2,00	3,00	1,00	1,00	1,00	1,00	1,00	1,00
2,00	1,00	1,00	3,00	2,00	2,00	2,00	1,00	1,00
0,00	1,00	0,00	0,00	0,00	1,00	3,00	2,00	3,00
1,00	2,00	2,00	0,00	1,00	0,00	0,00	2,00	1,00
3,00	3,00	2,00	3,00	3,00	3,00	2,00	3,00	3,00
1,00	1,00	1,00	0,00	0,00	3,00	3,00	3,00	3,00
0,00	2,00	2,00	2,00	1,00	1,00	2,00	3,00	1,00
1,00	1,00	2,00	1,00	1,00	3,00	3,00	2,00	3,00
1,00	1,00	1,00	1,00	1,00	2,00	3,00	1,00	3,00
2,00	3,00	3,00	3,00	2,00	1,00	1,00	3,00	3,00
1,00	1,00	1,00	2,00	1,00	1,00	1,00	3,00	3,00
2,00	3,00	1,00	3,00	2,00	1,00	2,00	2,00	3,00
0,00	1,00	1,00	1,00	0,00	3,00	3,00	3,00	3,00
0,00	1,00	0,00	0,00	0,00	3,00	3,00	3,00	3,00
1,00	2,00	1,00	1,00	1,00	1,00	2,00	3,00	2,00
0,00	2,00	0,00	1,00	0,00	3,00	3,00	3,00	3,00
1,00	3,00	2,00	3,00	1,00	2,00	3,00	3,00	3,00
0,00	1,00	3,00	1,00	1,00	3,00	2,00	3,00	3,00
1,00	2,00	2,00	1,00	1,00	2,00	2,00	2,00	3,00
1,00	1,00	1,00	1,00	0,00	1,00	2,00	2,00	3,00
1,00	1,00	2,00	1,00	1,00	1,00	1,00	2,00	2,00
0,00	1,00	1,00	1,00	0,00	3,00	3,00	2,00	2,00
1,00	3,00	2,00	3,00	1,00	1,00	1,00	0,00	2,00
1,00	2,00	1,00	1,00	2,00	0,00	0,00	2,00	2,00

2,00	2,00	1,00	1,00	1,00	1,00	2,00	3,00	2,00
3,00	3,00	2,00	3,00	3,00	0,00	1,00	1,00	1,00
2,00	2,00	2,00	2,00	2,00	0,00	0,00	1,00	1,00
2,00	3,00	1,00	3,00	3,00	0,00	1,00	0,00	1,00
1,00	2,00	2,00	0,00	1,00	1,00	1,00	3,00	2,00
1,00	0,00	0,00	0,00	0,00	3,00	3,00	2,00	3,00
1,00	1,00	1,00	2,00	1,00	1,00	1,00	0,00	2,00
1,00	1,00	1,00	2,00	0,00	3,00	3,00	3,00	3,00
2,00	3,00	3,00	3,00	2,00	3,00	2,00	2,00	2,00
1,00	3,00	2,00	2,00	0,00	3,00	3,00	1,00	1,00
1,00	2,00	2,00	2,00	1,00	2,00	3,00	3,00	2,00
1,00	2,00	1,00	1,00	1,00	1,00	2,00	1,00	1,00
2,00	3,00	2,00	3,00	3,00	1,00	0,00	2,00	1,00
0,00	1,00	1,00	1,00	0,00	2,00	3,00	3,00	3,00
1,00	3,00	2,00	1,00	1,00	3,00	2,00	3,00	3,00
0,00	2,00	2,00	1,00	1,00	2,00	2,00	3,00	2,00
0,00	1,00	0,00	1,00	0,00	2,00	3,00	3,00	3,00
1,00	2,00	2,00	3,00	2,00	1,00	1,00	3,00	3,00
2,00	2,00	2,00	1,00	0,00	2,00	1,00	1,00	3,00
0,00	2,00	1,00	1,00	1,00	3,00	3,00	3,00	3,00
2,00	1,00	1,00	1,00	1,00	1,00	0,00	3,00	2,00
0,00	1,00	0,00	0,00	0,00	3,00	3,00	1,00	3,00
2,00	3,00	2,00	3,00	1,00	1,00	1,00	2,00	2,00
0,00	2,00	1,00	0,00	0,00	3,00	3,00	2,00	3,00
0,00	1,00	1,00	1,00	0,00	2,00	2,00	3,00	3,00
2,00	2,00	2,00	1,00	2,00	1,00	1,00	2,00	2,00
0,00	2,00	1,00	0,00	0,00	1,00	1,00	3,00	1,00
2,00	2,00	2,00	2,00	2,00	1,00	1,00	3,00	1,00
0,00	1,00	0,00	0,00	1,00	3,00	3,00	2,00	3,00
1,00	1,00	1,00	1,00	1,00	3,00	2,00	3,00	3,00
1,00	2,00	1,00	1,00	1,00	3,00	3,00	3,00	2,00
2,00	3,00	2,00	2,00	3,00	3,00	3,00	3,00	3,00
2,00	3,00	0,00	2,00	1,00	1,00	1,00	2,00	1,00
2,00	3,00	2,00	2,00	2,00	2,00	1,00	2,00	2,00
1,00	2,00	2,00	2,00	1,00	2,00	2,00	3,00	3,00
0,00	0,00	0,00	0,00	0,00	3,00	2,00	3,00	2,00
2,00	3,00	3,00	3,00	2,00	1,00	1,00	1,00	3,00
2,00	2,00	2,00	2,00	2,00	3,00	3,00	1,00	2,00
0,00	2,00	2,00	1,00	1,00	2,00	2,00	1,00	2,00
0,00	0,00	0,00	0,00	0,00	1,00	3,00	3,00	2,00
0,00	1,00	1,00	1,00	0,00	2,00	3,00	3,00	3,00
2,00	2,00	2,00	1,00	2,00	1,00	1,00	3,00	2,00
0,00	2,00	3,00	0,00	1,00	3,00	3,00	1,00	2,00
1,00	3,00	1,00	0,00	0,00	1,00	2,00	3,00	3,00
1,00	3,00	1,00	0,00	1,00	2,00	2,00	3,00	3,00
1,00	2,00	1,00	1,00	0,00	1,00	1,00	2,00	2,00
3,00	3,00	3,00	3,00	3,00	0,00	0,00	1,00	1,00
1,00	3,00	2,00	3,00	1,00	1,00	1,00	0,00	3,00
0,00	1,00	1,00	1,00	1,00	3,00	2,00	3,00	3,00
1,00	2,00	1,00	1,00	1,00	2,00	2,00	2,00	1,00

1,00	2,00	2,00	3,00	2,00	1,00	1,00	1,00	1,00
1,00	2,00	1,00	1,00	2,00	2,00	1,00	1,00	2,00
1,00	2,00	2,00	0,00	2,00	2,00	1,00	0,00	1,00
1,00	2,00	2,00	3,00	1,00	1,00	0,00	0,00	1,00
2,00	2,00	1,00	1,00	2,00	2,00	1,00	1,00	2,00
1,00	2,00	2,00	0,00	1,00	1,00	1,00	3,00	2,00
2,00	2,00	1,00	0,00	1,00	1,00	2,00	2,00	2,00
0,00	2,00	1,00	2,00	2,00	3,00	3,00	2,00	3,00
2,00	3,00	1,00	2,00	2,00	1,00	1,00	3,00	0,00
2,00	2,00	2,00	2,00	2,00	1,00	2,00	2,00	2,00
1,00	1,00	1,00	2,00	1,00	1,00	1,00	0,00	2,00
1,00	2,00	2,00	3,00	2,00	1,00	1,00	1,00	0,00
2,00	2,00	1,00	3,00	3,00	0,00	1,00	1,00	1,00
2,00	3,00	2,00	2,00	1,00	1,00	2,00	2,00	2,00
0,00	2,00	1,00	1,00	1,00	3,00	3,00	2,00	3,00
2,00	2,00	1,00	2,00	1,00	1,00	1,00	0,00	1,00
0,00	1,00	0,00	0,00	0,00	3,00	3,00	3,00	3,00
1,00	3,00	1,00	1,00	3,00	1,00	1,00	3,00	1,00
0,00	1,00	0,00	0,00	0,00	3,00	3,00	2,00	3,00
2,00	1,00	2,00	1,00	1,00	1,00	1,00	1,00	2,00
2,00	3,00	2,00	3,00	2,00	1,00	1,00	3,00	2,00
2,00	2,00	1,00	3,00	2,00	1,00	1,00	1,00	1,00
2,00	2,00	2,00	2,00	2,00	1,00	1,00	1,00	1,00
2,00	2,00	1,00	2,00	2,00	1,00	1,00	2,00	1,00
1,00	2,00	2,00	2,00	1,00	2,00	3,00	2,00	1,00
0,00	1,00	0,00	0,00	0,00	3,00	3,00	2,00	3,00
0,00	2,00	1,00	1,00	1,00	1,00	2,00	2,00	3,00
0,00	2,00	2,00	1,00	1,00	2,00	1,00	2,00	2,00
1,00	2,00	2,00	2,00	2,00	2,00	1,00	3,00	2,00
1,00	3,00	1,00	2,00	0,00	3,00	2,00	2,00	2,00
1,00	2,00	1,00	0,00	1,00	2,00	1,00	1,00	2,00
2,00	2,00	2,00	2,00	1,00	2,00	1,00	3,00	2,00
1,00	2,00	1,00	2,00	1,00	1,00	1,00	2,00	3,00
2,00	2,00	2,00	0,00	2,00	2,00	1,00	1,00	2,00
1,00	2,00	2,00	2,00	1,00	3,00	2,00	3,00	3,00
2,00	2,00	2,00	2,00	2,00	0,00	1,00	1,00	1,00
0,00	1,00	0,00	0,00	0,00	3,00	2,00	3,00	3,00
1,00	1,00	1,00	1,00	0,00	1,00	3,00	3,00	2,00
1,00	3,00	3,00	3,00	2,00	3,00	3,00	3,00	3,00
1,00	1,00	1,00	2,00	0,00	3,00	3,00	0,00	3,00
1,00	2,00	2,00	1,00	1,00	2,00	2,00	1,00	3,00
0,00	1,00	1,00	0,00	0,00	1,00	2,00	0,00	1,00
1,00	1,00	1,00	0,00	1,00	1,00	1,00	3,00	2,00
2,00	1,00	0,00	1,00	1,00	2,00	2,00	3,00	3,00
3,00	2,00	2,00	2,00	3,00	3,00	3,00	3,00	3,00
2,00	0,00	0,00	3,00	3,00	3,00	3,00	3,00	2,00
3,00	3,00	3,00	3,00	3,00	1,00	1,00	1,00	1,00
2,00	2,00	2,00	2,00	2,00	1,00	2,00	2,00	2,00
1,00	1,00	1,00	1,00	1,00	3,00	3,00	2,00	3,00
1,00	1,00	2,00	2,00	1,00	1,00	1,00	2,00	2,00

1,00	1,00	1,00	1,00	1,00	2,00	2,00	1,00	1,00
0,00	2,00	0,00	1,00	0,00	2,00	3,00	3,00	3,00
2,00	3,00	1,00	1,00	1,00	2,00	1,00	3,00	2,00
0,00	1,00	1,00	1,00	1,00	3,00	3,00	3,00	3,00
2,00	3,00	3,00	3,00	3,00	3,00	3,00	0,00	3,00
0,00	2,00	1,00	2,00	1,00	2,00	2,00	1,00	2,00
0,00	1,00	1,00	2,00	1,00	3,00	3,00	3,00	3,00
1,00	1,00	1,00	2,00	1,00	1,00	1,00	3,00	2,00
1,00	2,00	3,00	2,00	2,00	1,00	1,00	2,00	2,00
0,00	1,00	0,00	0,00	0,00	2,00	3,00	3,00	3,00
2,00	3,00	1,00	1,00	2,00	1,00	0,00	0,00	1,00
0,00	2,00	1,00	1,00	0,00	1,00	3,00	3,00	3,00
0,00	1,00	1,00	1,00	1,00	1,00	1,00	1,00	1,00
1,00	3,00	3,00	2,00	1,00	3,00	3,00	2,00	3,00
1,00	1,00	1,00	2,00	1,00	3,00	3,00	1,00	3,00
1,00	1,00	0,00	0,00	0,00	3,00	2,00	3,00	3,00
2,00	3,00	2,00	2,00	1,00	1,00	2,00	3,00	2,00
2,00	2,00	1,00	2,00	2,00	1,00	1,00	0,00	1,00
1,00	2,00	2,00	1,00	1,00	3,00	2,00	3,00	1,00
1,00	2,00	1,00	1,00	1,00	2,00	2,00	1,00	3,00
0,00	0,00	0,00	0,00	0,00	3,00	3,00	3,00	3,00
2,00	2,00	3,00	2,00	2,00	1,00	1,00	1,00	1,00
1,00	3,00	2,00	2,00	2,00	2,00	1,00	1,00	2,00
0,00	1,00	1,00	1,00	0,00	3,00	3,00	1,00	3,00
2,00	2,00	2,00	2,00	1,00	1,00	1,00	2,00	1,00
0,00	1,00	1,00	1,00	0,00	3,00	3,00	1,00	2,00
1,00	3,00	2,00	3,00	2,00	1,00	2,00	3,00	3,00
1,00	2,00	1,00	2,00	1,00	3,00	2,00	1,00	2,00
1,00	2,00	2,00	2,00	1,00	1,00	1,00	1,00	2,00
1,00	1,00	1,00	2,00	1,00	2,00	1,00	1,00	2,00
1,00	2,00	2,00	2,00	2,00	1,00	1,00	2,00	2,00
2,00	2,00	2,00	1,00	2,00	1,00	1,00	2,00	3,00
2,00	2,00	2,00	2,00	2,00	1,00	2,00	3,00	2,00
1,00	2,00	2,00	2,00	1,00	1,00	1,00	1,00	2,00
0,00	1,00	1,00	0,00	0,00	3,00	3,00	1,00	3,00
1,00	2,00	2,00	2,00	1,00	1,00	2,00	1,00	3,00
1,00	2,00	2,00	1,00	2,00	1,00	2,00	3,00	3,00
1,00	2,00	1,00	1,00	1,00	2,00	3,00	2,00	2,00
3,00	2,00	2,00	2,00	2,00	2,00	1,00	2,00	2,00
1,00	2,00	2,00	1,00	1,00	1,00	1,00	1,00	3,00
2,00	2,00	2,00	2,00	1,00	1,00	2,00	2,00	2,00
1,00	1,00	1,00	1,00	1,00	1,00	2,00	3,00	3,00
0,00	1,00	1,00	1,00	0,00	3,00	2,00	2,00	2,00
1,00	2,00	2,00	2,00	2,00	0,00	1,00	2,00	1,00
1,00	1,00	1,00	1,00	0,00	2,00	3,00	2,00	3,00
0,00	2,00	2,00	2,00	1,00	3,00	3,00	3,00	3,00
2,00	3,00	2,00	3,00	1,00	2,00	1,00	3,00	2,00
0,00	1,00	1,00	0,00	0,00	3,00	3,00	3,00	3,00
1,00	3,00	3,00	1,00	1,00	3,00	3,00	1,00	2,00
1,00	3,00	2,00	2,00	1,00	2,00	2,00	1,00	1,00

0,00	1,00	2,00	0,00	0,00	3,00	3,00	2,00	3,00
1,00	3,00	1,00	2,00	1,00	2,00	2,00	1,00	3,00
2,00	1,00	2,00	1,00	1,00	1,00	1,00	2,00	1,00
2,00	2,00	3,00	3,00	2,00	3,00	2,00	3,00	3,00
1,00	1,00	1,00	2,00	3,00	1,00	1,00	1,00	1,00
1,00	3,00	1,00	2,00	2,00	1,00	1,00	1,00	2,00
2,00	2,00	2,00	1,00	1,00	1,00	2,00	3,00	3,00
1,00	2,00	3,00	3,00	3,00	0,00	1,00	1,00	3,00
1,00	1,00	1,00	2,00	1,00	1,00	0,00	1,00	1,00
1,00	2,00	1,00	1,00	1,00	2,00	2,00	1,00	1,00
2,00	2,00	1,00	1,00	1,00	1,00	1,00	2,00	1,00
1,00	2,00	2,00	1,00	1,00	2,00	3,00	2,00	3,00
2,00	3,00	3,00	3,00	2,00	1,00	0,00	2,00	1,00
1,00	2,00	2,00	1,00	1,00	1,00	1,00	1,00	1,00
2,00	2,00	2,00	2,00	2,00	1,00	1,00	0,00	1,00
0,00	1,00	1,00	2,00	1,00	2,00	2,00	3,00	2,00
0,00	1,00	1,00	0,00	0,00	2,00	2,00	3,00	3,00
1,00	2,00	2,00	3,00	1,00	3,00	2,00	1,00	3,00
1,00	1,00	1,00	1,00	1,00	1,00	0,00	0,00	1,00
1,00	3,00	2,00	2,00	1,00	3,00	2,00	1,00	3,00
3,00	2,00	3,00	3,00	3,00	1,00	3,00	0,00	3,00
1,00	2,00	1,00	0,00	1,00	2,00	2,00	3,00	3,00
1,00	2,00	2,00	1,00	1,00	3,00	3,00	1,00	3,00
2,00	3,00	3,00	3,00	2,00	1,00	1,00	2,00	1,00
0,00	2,00	0,00	2,00	0,00	3,00	3,00	3,00	3,00
2,00	1,00	1,00	1,00	1,00	1,00	1,00	2,00	2,00
1,00	2,00	1,00	1,00	1,00	1,00	1,00	0,00	1,00
3,00	2,00	2,00	3,00	3,00	1,00	1,00	1,00	3,00
1,00	2,00	2,00	2,00	0,00	3,00	3,00	3,00	3,00
2,00	3,00	3,00	2,00	2,00	1,00	1,00	2,00	1,00
1,00	1,00	2,00	3,00	1,00	1,00	1,00	1,00	2,00
1,00	3,00	2,00	2,00	2,00	2,00	1,00	1,00	2,00
1,00	1,00	1,00	1,00	1,00	2,00	2,00	1,00	2,00
1,00	2,00	1,00	1,00	0,00	2,00	2,00	1,00	2,00
0,00	1,00	1,00	0,00	0,00	2,00	2,00	2,00	3,00
1,00	2,00	2,00	1,00	1,00	1,00	1,00	3,00	2,00
1,00	1,00	1,00	1,00	1,00	1,00	3,00	3,00	3,00
2,00	3,00	3,00	3,00	2,00	1,00	0,00	1,00	1,00
1,00	2,00	1,00	2,00	1,00	2,00	2,00	0,00	3,00
1,00	1,00	1,00	1,00	1,00	1,00	3,00	3,00	3,00
1,00	2,00	2,00	1,00	0,00	3,00	2,00	2,00	2,00
2,00	2,00	2,00	1,00	2,00	1,00	1,00	1,00	2,00
1,00	1,00	0,00	0,00	1,00	3,00	3,00	3,00	2,00
2,00	2,00	0,00	0,00	1,00	2,00	1,00	0,00	0,00
0,00	1,00	2,00	0,00	0,00	2,00	3,00	3,00	3,00
1,00	1,00	1,00	0,00	0,00	3,00	2,00	3,00	2,00
0,00	0,00	1,00	0,00	0,00	3,00	3,00	3,00	3,00
1,00	2,00	2,00	1,00	2,00	1,00	2,00	2,00	2,00
1,00	2,00	2,00	2,00	1,00	2,00	1,00	3,00	2,00
2,00	1,00	1,00	3,00	1,00	1,00	2,00	2,00	3,00

2,00	2,00	2,00	2,00	2,00	3,00	2,00	0,00	2,00
2,00	2,00	1,00	2,00	2,00	0,00	0,00	0,00	1,00
2,00	2,00	3,00	0,00	2,00	3,00	3,00	3,00	2,00
2,00	3,00	3,00	3,00	2,00	1,00	1,00	2,00	1,00
1,00	2,00	1,00	1,00	1,00	2,00	1,00	3,00	2,00
1,00	3,00	3,00	2,00	1,00	2,00	2,00	3,00	2,00
1,00	3,00	2,00	2,00	1,00	2,00	2,00	1,00	1,00
2,00	3,00	2,00	3,00	2,00	1,00	1,00	3,00	2,00
1,00	2,00	2,00	1,00	3,00	3,00	2,00	3,00	1,00
1,00	1,00	2,00	1,00	0,00	2,00	1,00	1,00	2,00
1,00	1,00	1,00	0,00	0,00	3,00	2,00	2,00	3,00
1,00	1,00	1,00	1,00	1,00	2,00	2,00	2,00	2,00
0,00	2,00	1,00	0,00	0,00	3,00	3,00	3,00	3,00
1,00	2,00	2,00	2,00	1,00	1,00	1,00	1,00	1,00
2,00	2,00	1,00	1,00	1,00	1,00	1,00	0,00	2,00
2,00	2,00	2,00	1,00	2,00	1,00	1,00	1,00	1,00
2,00	3,00	3,00	3,00	3,00	0,00	0,00	0,00	0,00
1,00	2,00	2,00	2,00	1,00	3,00	3,00	3,00	3,00
2,00	2,00	2,00	2,00	2,00	1,00	1,00	1,00	0,00
2,00	3,00	2,00	1,00	1,00	2,00	2,00	3,00	2,00
0,00	2,00	0,00	1,00	2,00	0,00	1,00	2,00	0,00
1,00	3,00	1,00	3,00	2,00	1,00	1,00	3,00	2,00
1,00	2,00	2,00	1,00	2,00	2,00	2,00	3,00	3,00
2,00	2,00	1,00	1,00	3,00	2,00	2,00	3,00	2,00
1,00	2,00	3,00	1,00	0,00	1,00	2,00	3,00	3,00
2,00	2,00	2,00	2,00	2,00	1,00	1,00	0,00	1,00
2,00	2,00	3,00	3,00	2,00	1,00	2,00	1,00	0,00
2,00	2,00	2,00	1,00	2,00	1,00	1,00	3,00	1,00
1,00	2,00	0,00	0,00	2,00	1,00	1,00	2,00	2,00
2,00	0,00	0,00	2,00	2,00	1,00	1,00	1,00	1,00
0,00	1,00	1,00	1,00	0,00	3,00	2,00	2,00	3,00

PH05	PH06	PH07	PH08	PH09	fam	part	schul	PHQ_kat
1,00	0,00	0,00	0,00	0,00	1,00	0,00	1,00	1,00
3,00	3,00	2,00	0,00	1,00	1,00	0,00	1,00	4,00
3,00	0,00	1,00	0,00	2,00	1,00	0,00	1,00	3,00
2,00	2,00	2,00	1,00	0,00	0,00	1,00	1,00	3,00
1,00	1,00	1,00	1,00	1,00	1,00	0,00	1,00	3,00
3,00	1,00	3,00	1,00	1,00	1,00	0,00	0,00	4,00
1,00	2,00	0,00	0,00	0,00	1,00	0,00	1,00	2,00
0,00	3,00	1,00	0,00	1,00	0,00	0,00	1,00	4,00
2,00	2,00	3,00	1,00	1,00	1,00	0,00	1,00	4,00
0,00	0,00	0,00	0,00	0,00	1,00	0,00	1,00	2,00
1,00	0,00	1,00	0,00	0,00	1,00	0,00	0,00	1,00
3,00	2,00	3,00	1,00	0,00	2,00	1,00	0,00	5,00
2,00	2,00	1,00	0,00	0,00	1,00	0,00	1,00	4,00
1,00	3,00	3,00	0,00	2,00	1,00	0,00	1,00	5,00
1,00	1,00	1,00	2,00	0,00	1,00	0,00	1,00	2,00
1,00	3,00	1,00	0,00	1,00	1,00	0,00	1,00	3,00
2,00	3,00	0,00	0,00	0,00	2,00	1,00	1,00	3,00
2,00	3,00	2,00	1,00	3,00	1,00	0,00	0,00	5,00
2,00	1,00	2,00	1,00	1,00	1,00	0,00	1,00	3,00
0,00	0,00	0,00	0,00	0,00	1,00	0,00	1,00	1,00
2,00	1,00	1,00	1,00	0,00	0,00	1,00	1,00	2,00
0,00	0,00	1,00	1,00	0,00	2,00	1,00	1,00	2,00
2,00	1,00	3,00	1,00	2,00	0,00	0,00	0,00	4,00
1,00	3,00	1,00	0,00	3,00	2,00	1,00	0,00	4,00
0,00	1,00	1,00	0,00	1,00	0,00	0,00	1,00	3,00
0,00	1,00	3,00	0,00	0,00	0,00	1,00	1,00	3,00
0,00	1,00	0,00	1,00	1,00	1,00	0,00	0,00	2,00
0,00	0,00	0,00	0,00	0,00	1,00	0,00	1,00	1,00
3,00	2,00	2,00	2,00	1,00	2,00	1,00	0,00	5,00
1,00	3,00	2,00	0,00	0,00	1,00	0,00	1,00	3,00
2,00	3,00	1,00	1,00	2,00	2,00	1,00	0,00	4,00
2,00	1,00	3,00	1,00	1,00	2,00	1,00	1,00	4,00
1,00	1,00	2,00	3,00	1,00	2,00	1,00	0,00	4,00
3,00	3,00	2,00	2,00	3,00	0,00	0,00	0,00	5,00
3,00	2,00	1,00	2,00	2,00	1,00	0,00	1,00	5,00
1,00	1,00	3,00	1,00	1,00	2,00	1,00	0,00	4,00
2,00	0,00	0,00	0,00	1,00	1,00	0,00	1,00	2,00
3,00	3,00	3,00	0,00	3,00	1,00	0,00	0,00	5,00
2,00	1,00	1,00	0,00	1,00	0,00	0,00	1,00	2,00
0,00	2,00	3,00	0,00	0,00	2,00	1,00	1,00	3,00
2,00	1,00	1,00	1,00	1,00	2,00	1,00	1,00	3,00
0,00	0,00	0,00	0,00	0,00	2,00	1,00	1,00	2,00
0,00	0,00	0,00	0,00	0,00	2,00	1,00	1,00	1,00
3,00	2,00	3,00	2,00	0,00	0,00	0,00	1,00	4,00
0,00	1,00	1,00	0,00	0,00	0,00	0,00	0,00	2,00
0,00	1,00	1,00	0,00	0,00	2,00	1,00	1,00	2,00
2,00	0,00	1,00	0,00	0,00	2,00	1,00	0,00	2,00
2,00	3,00	3,00	3,00	0,00	0,00	0,00	1,00	5,00
2,00	0,00	2,00	3,00	3,00	0,00	0,00	1,00	4,00

1,00	0,00	2,00	2,00	0,00	1,00	0,00	0,00	3,00
3,00	0,00	1,00	0,00	0,00	0,00	0,00	0,00	3,00
2,00	3,00	3,00	3,00	3,00	1,00	0,00	0,00	5,00
2,00	2,00	2,00	0,00	2,00	2,00	1,00	1,00	5,00
0,00	0,00	1,00	0,00	0,00	2,00	1,00	1,00	1,00
1,00	1,00	2,00	0,00	0,00	0,00	0,00	1,00	2,00
3,00	0,00	3,00	3,00	0,00	2,00	1,00	1,00	4,00
0,00	2,00	1,00	0,00	1,00	0,00	1,00	1,00	3,00
2,00	1,00	1,00	1,00	0,00	0,00	0,00	0,00	3,00
1,00	3,00	3,00	2,00	1,00	1,00	0,00	1,00	5,00
2,00	2,00	3,00	2,00	0,00	1,00	0,00	1,00	4,00
2,00	3,00	1,00	0,00	2,00	1,00	0,00	1,00	4,00
3,00	0,00	1,00	0,00	0,00	0,00	1,00	1,00	2,00
2,00	1,00	1,00	0,00	2,00	1,00	0,00	1,00	4,00
1,00	3,00	2,00	0,00	3,00	1,00	0,00	0,00	4,00
0,00	2,00	0,00	0,00	1,00	2,00	1,00	1,00	3,00
0,00	0,00	0,00	0,00	0,00	2,00	1,00	1,00	1,00
1,00	2,00	3,00	0,00	0,00	2,00	1,00	1,00	3,00
1,00	3,00	1,00	0,00	0,00	0,00	0,00	0,00	3,00
2,00	2,00	2,00	1,00	0,00	2,00	1,00	1,00	3,00
1,00	2,00	3,00	0,00	0,00	0,00	0,00	1,00	4,00
0,00	1,00	0,00	0,00	0,00	2,00	1,00	1,00	1,00
2,00	0,00	0,00	0,00	0,00	2,00	1,00	1,00	2,00
1,00	1,00	3,00	2,00	0,00	2,00	1,00	1,00	3,00
3,00	1,00	2,00	1,00	1,00	1,00	0,00	0,00	4,00
3,00	1,00	1,00	0,00	0,00	0,00	1,00	1,00	2,00
3,00	3,00	1,00	0,00	3,00	1,00	0,00	0,00	5,00
3,00	3,00	3,00	3,00	3,00	0,00	1,00	0,00	5,00
0,00	1,00	1,00	0,00	0,00	0,00	1,00	1,00	1,00
1,00	1,00	2,00	0,00	0,00	1,00	0,00	1,00	3,00
2,00	3,00	1,00	1,00	1,00	1,00	0,00	1,00	3,00
2,00	2,00	1,00	1,00	2,00	1,00	0,00	1,00	4,00
3,00	2,00	2,00	2,00	1,00	0,00	0,00	1,00	5,00
1,00	1,00	1,00	0,00	0,00	2,00	1,00	1,00	2,00
2,00	1,00	1,00	0,00	2,00	1,00	0,00	1,00	4,00
1,00	0,00	0,00	0,00	0,00	0,00	1,00	1,00	1,00
0,00	0,00	0,00	0,00	0,00	0,00	1,00	1,00	1,00
2,00	3,00	3,00	2,00	1,00	1,00	0,00	0,00	5,00
2,00	1,00	1,00	1,00	1,00	2,00	1,00	0,00	3,00
2,00	1,00	1,00	1,00	0,00	2,00	1,00	1,00	3,00
2,00	3,00	0,00	0,00	1,00	1,00	0,00	0,00	3,00
2,00	1,00	1,00	0,00	1,00	1,00	0,00	1,00	3,00
2,00	1,00	2,00	1,00	1,00	0,00	1,00	0,00	4,00
1,00	2,00	2,00	0,00	0,00	0,00	0,00	0,00	3,00
3,00	2,00	2,00	1,00	1,00	0,00	0,00	0,00	4,00
3,00	3,00	2,00	2,00	3,00	1,00	0,00	1,00	5,00
3,00	3,00	3,00	2,00	1,00	2,00	1,00	1,00	5,00
1,00	1,00	3,00	2,00	1,00	0,00	1,00	1,00	4,00
1,00	3,00	1,00	1,00	3,00	1,00	0,00	0,00	4,00
3,00	3,00	2,00	1,00	1,00	1,00	0,00	1,00	4,00

1,00	1,00	2,00	2,00	0,00	2,00	1,00	1,00	3,00
3,00	2,00	2,00	1,00	1,00	1,00	0,00	1,00	4,00
3,00	3,00	3,00	1,00	0,00	1,00	0,00	0,00	5,00
2,00	2,00	2,00	0,00	1,00	1,00	0,00	0,00	4,00
2,00	0,00	1,00	1,00	1,00	0,00	0,00	1,00	3,00
1,00	2,00	2,00	0,00	1,00	1,00	0,00	0,00	3,00
1,00	2,00	2,00	2,00	1,00	2,00	1,00	1,00	4,00
3,00	2,00	1,00	1,00	0,00	1,00	0,00	0,00	3,00
1,00	3,00	1,00	0,00	2,00	1,00	0,00	1,00	4,00
2,00	1,00	2,00	3,00	1,00	2,00	1,00	1,00	3,00
2,00	2,00	3,00	1,00	3,00	1,00	0,00	0,00	5,00
1,00	1,00	2,00	1,00	0,00	1,00	0,00	0,00	4,00
1,00	1,00	1,00	0,00	0,00	0,00	0,00	0,00	2,00
0,00	1,00	2,00	0,00	0,00	0,00	1,00	1,00	2,00
2,00	3,00	1,00	0,00	3,00	1,00	0,00	0,00	4,00
1,00	2,00	2,00	1,00	2,00	2,00	1,00	0,00	4,00
2,00	1,00	1,00	0,00	1,00	0,00	0,00	1,00	3,00
2,00	1,00	1,00	0,00	0,00	1,00	0,00	1,00	2,00
0,00	1,00	1,00	0,00	0,00	2,00	1,00	1,00	1,00
1,00	3,00	3,00	2,00	2,00	1,00	0,00	1,00	5,00
1,00	1,00	1,00	0,00	1,00	0,00	0,00	0,00	3,00
2,00	1,00	3,00	0,00	2,00	1,00	0,00	1,00	4,00
3,00	2,00	3,00	2,00	1,00	0,00	0,00	1,00	5,00
2,00	2,00	3,00	3,00	1,00	0,00	0,00	1,00	5,00
2,00	2,00	1,00	0,00	1,00	1,00	0,00	0,00	4,00
2,00	3,00	3,00	3,00	1,00	1,00	0,00	1,00	5,00
2,00	3,00	2,00	1,00	2,00	1,00	0,00	1,00	4,00
3,00	3,00	2,00	1,00	1,00	2,00	1,00	1,00	4,00
1,00	2,00	2,00	1,00	0,00	2,00	1,00	1,00	2,00
3,00	2,00	2,00	1,00	3,00	2,00	1,00	1,00	5,00
2,00	2,00	3,00	3,00	1,00	0,00	0,00	1,00	4,00
0,00	1,00	1,00	0,00	0,00	2,00	1,00	1,00	2,00
2,00	1,00	0,00	0,00	0,00	2,00	1,00	0,00	2,00
0,00	0,00	0,00	1,00	0,00	0,00	1,00	1,00	1,00
2,00	3,00	1,00	0,00	0,00	0,00	0,00	0,00	3,00
1,00	3,00	3,00	2,00	1,00	2,00	1,00	0,00	3,00
0,00	0,00	0,00	0,00	0,00	1,00	0,00	1,00	1,00
1,00	0,00	0,00	0,00	1,00	0,00	1,00	1,00	1,00
1,00	2,00	0,00	0,00	1,00	1,00	0,00	1,00	3,00
2,00	0,00	2,00	3,00	0,00	2,00	1,00	1,00	3,00
0,00	1,00	2,00	0,00	1,00	2,00	1,00	0,00	3,00
1,00	0,00	0,00	0,00	0,00	0,00	1,00	1,00	1,00
2,00	2,00	1,00	1,00	1,00	1,00	0,00	1,00	3,00
2,00	3,00	3,00	1,00	3,00	1,00	0,00	0,00	5,00
2,00	2,00	2,00	2,00	0,00	1,00	0,00	0,00	4,00
0,00	2,00	2,00	3,00	2,00	2,00	1,00	1,00	4,00
0,00	3,00	3,00	3,00	3,00	1,00	0,00	0,00	5,00
3,00	3,00	3,00	1,00	1,00	0,00	1,00	1,00	5,00
1,00	0,00	1,00	0,00	0,00	1,00	0,00	1,00	2,00
1,00	0,00	3,00	2,00	1,00	0,00	0,00	1,00	4,00

0,00	1,00	1,00	0,00	0,00	2,00	1,00	1,00	2,00
0,00	0,00	0,00	0,00	0,00	2,00	1,00	0,00	1,00
0,00	0,00	0,00	1,00	1,00	2,00	1,00	0,00	2,00
0,00	1,00	0,00	0,00	2,00	1,00	0,00	1,00	2,00
2,00	1,00	3,00	2,00	1,00	1,00	0,00	1,00	4,00
1,00	2,00	3,00	0,00	1,00	0,00	1,00	1,00	3,00
1,00	1,00	0,00	0,00	0,00	1,00	0,00	1,00	1,00
1,00	2,00	2,00	1,00	1,00	1,00	0,00	0,00	4,00
1,00	1,00	1,00	0,00	0,00	0,00	1,00	1,00	3,00
2,00	1,00	1,00	0,00	1,00	0,00	0,00	1,00	3,00
2,00	1,00	3,00	1,00	1,00	1,00	0,00	1,00	3,00
2,00	3,00	1,00	0,00	2,00	1,00	0,00	0,00	3,00
0,00	0,00	0,00	0,00	0,00	1,00	0,00	1,00	1,00
3,00	0,00	3,00	0,00	0,00	0,00	1,00	0,00	4,00
1,00	1,00	1,00	1,00	0,00	1,00	0,00	1,00	3,00
2,00	3,00	3,00	1,00	1,00	2,00	1,00	0,00	5,00
3,00	1,00	1,00	0,00	0,00	0,00	0,00	1,00	3,00
2,00	3,00	3,00	3,00	2,00	2,00	1,00	1,00	5,00
1,00	0,00	3,00	0,00	1,00	2,00	1,00	1,00	3,00
2,00	3,00	3,00	2,00	3,00	1,00	0,00	1,00	5,00
2,00	3,00	1,00	0,00	1,00	1,00	0,00	1,00	3,00
1,00	2,00	3,00	2,00	1,00	1,00	0,00	1,00	4,00
2,00	3,00	3,00	1,00	3,00	1,00	0,00	0,00	5,00
1,00	3,00	2,00	0,00	0,00	0,00	0,00	1,00	3,00
2,00	3,00	1,00	2,00	3,00	0,00	0,00	0,00	5,00
3,00	1,00	3,00	1,00	0,00	2,00	1,00	1,00	4,00
2,00	2,00	0,00	0,00	1,00	0,00	1,00	1,00	3,00
3,00	2,00	1,00	1,00	1,00	1,00	0,00	1,00	4,00
3,00	3,00	3,00	0,00	3,00	1,00	0,00	1,00	5,00
1,00	3,00	3,00	0,00	2,00	1,00	0,00	1,00	4,00
1,00	0,00	0,00	0,00	0,00	1,00	0,00	0,00	1,00
3,00	3,00	3,00	0,00	1,00	0,00	0,00	0,00	4,00
1,00	3,00	1,00	1,00	2,00	0,00	1,00	1,00	4,00
0,00	0,00	2,00	1,00	0,00	0,00	0,00	1,00	2,00
0,00	0,00	0,00	0,00	0,00	0,00	1,00	0,00	2,00
0,00	2,00	1,00	1,00	0,00	2,00	1,00	1,00	2,00
2,00	2,00	1,00	0,00	0,00	0,00	1,00	1,00	3,00
2,00	3,00	3,00	3,00	1,00	0,00	0,00	0,00	5,00
2,00	3,00	3,00	1,00	0,00	2,00	1,00	1,00	4,00
2,00	2,00	2,00	0,00	1,00	1,00	0,00	1,00	4,00
1,00	2,00	2,00	1,00	0,00	1,00	0,00	1,00	3,00
2,00	1,00	1,00	1,00	0,00	2,00	1,00	1,00	3,00
3,00	3,00	2,00	3,00	2,00	0,00	1,00	1,00	5,00
3,00	2,00	2,00	0,00	1,00	0,00	0,00	0,00	4,00
3,00	3,00	3,00	0,00	3,00	0,00	1,00	0,00	5,00
3,00	1,00	3,00	1,00	0,00	2,00	1,00	1,00	4,00
3,00	1,00	2,00	2,00	3,00	1,00	0,00	1,00	5,00
1,00	3,00	0,00	1,00	2,00	0,00	0,00	1,00	4,00
0,00	2,00	0,00	1,00	0,00	2,00	1,00	1,00	3,00
1,00	3,00	2,00	2,00	3,00	2,00	1,00	1,00	5,00

3,00	3,00	3,00	3,00	3,00	2,00	1,00	1,00	5,00
2,00	3,00	2,00	1,00	0,00	1,00	0,00	1,00	4,00
0,00	2,00	2,00	1,00	2,00	0,00	0,00	1,00	4,00
1,00	2,00	3,00	1,00	0,00	2,00	1,00	1,00	3,00
3,00	3,00	3,00	2,00	3,00	1,00	0,00	1,00	5,00
2,00	3,00	1,00	0,00	2,00	1,00	0,00	0,00	4,00
2,00	3,00	3,00	1,00	1,00	0,00	0,00	0,00	4,00
2,00	3,00	1,00	3,00	0,00	0,00	1,00	0,00	3,00
2,00	3,00	3,00	2,00	1,00	1,00	0,00	1,00	5,00
1,00	2,00	1,00	0,00	1,00	0,00	1,00	1,00	4,00
2,00	3,00	1,00	1,00	2,00	0,00	0,00	0,00	5,00
0,00	1,00	1,00	1,00	0,00	2,00	1,00	1,00	2,00
3,00	3,00	2,00	1,00	2,00	0,00	1,00	1,00	5,00
1,00	3,00	3,00	2,00	2,00	2,00	1,00	1,00	5,00
2,00	3,00	3,00	3,00	2,00	1,00	0,00	0,00	5,00
3,00	3,00	3,00	0,00	3,00	1,00	0,00	1,00	5,00
1,00	1,00	1,00	0,00	0,00	2,00	1,00	1,00	3,00
2,00	2,00	1,00	1,00	1,00	1,00	0,00	1,00	4,00
3,00	1,00	3,00	1,00	0,00	0,00	1,00	1,00	4,00
3,00	3,00	2,00	1,00	2,00	0,00	1,00	1,00	5,00
2,00	1,00	1,00	1,00	1,00	0,00	1,00	0,00	2,00
1,00	3,00	3,00	1,00	2,00	1,00	0,00	1,00	5,00
3,00	2,00	1,00	0,00	1,00	1,00	0,00	0,00	4,00
1,00	2,00	2,00	1,00	0,00	1,00	0,00	1,00	3,00
2,00	1,00	0,00	1,00	0,00	1,00	0,00	1,00	2,00
3,00	2,00	2,00	1,00	3,00	1,00	0,00	1,00	5,00
2,00	3,00	3,00	0,00	3,00	1,00	0,00	1,00	5,00
3,00	2,00	3,00	1,00	2,00	1,00	0,00	1,00	5,00
3,00	3,00	2,00	1,00	1,00	2,00	1,00	0,00	4,00
1,00	2,00	3,00	0,00	2,00	2,00	1,00	0,00	5,00
3,00	1,00	2,00	1,00	1,00	2,00	1,00	1,00	4,00
0,00	0,00	0,00	0,00	0,00	1,00	0,00	0,00	1,00
1,00	1,00	1,00	0,00	1,00	1,00	0,00	1,00	2,00
1,00	0,00	1,00	2,00	1,00	0,00	0,00	1,00	2,00
3,00	3,00	3,00	3,00	3,00	0,00	1,00	0,00	5,00
0,00	1,00	2,00	0,00	0,00	2,00	1,00	1,00	3,00
1,00	2,00	1,00	2,00	1,00	0,00	1,00	1,00	4,00
1,00	1,00	2,00	1,00	0,00	2,00	1,00	0,00	2,00
1,00	1,00	1,00	2,00	1,00	1,00	0,00	1,00	3,00
1,00	3,00	3,00	1,00	3,00	1,00	0,00	1,00	5,00
2,00	3,00	1,00	0,00	3,00	1,00	0,00	0,00	4,00
2,00	3,00	3,00	0,00	2,00	2,00	1,00	0,00	4,00
1,00	1,00	3,00	1,00	0,00	0,00	0,00	1,00	3,00
0,00	1,00	2,00	0,00	1,00	1,00	0,00	0,00	3,00
3,00	3,00	3,00	1,00	3,00	1,00	0,00	1,00	5,00
3,00	0,00	3,00	2,00	3,00	1,00	0,00	0,00	5,00
3,00	3,00	3,00	2,00	2,00	1,00	0,00	1,00	5,00
1,00	1,00	1,00	1,00	1,00	1,00	0,00	0,00	2,00
2,00	1,00	1,00	1,00	0,00	0,00	1,00	1,00	3,00
3,00	2,00	2,00	2,00	2,00	1,00	0,00	1,00	5,00

1,00	1,00	2,00	0,00	1,00	0,00	0,00	1,00	3,00
3,00	1,00	2,00	0,00	1,00	2,00	1,00	1,00	4,00
2,00	0,00	1,00	1,00	0,00	0,00	0,00	0,00	3,00
0,00	0,00	1,00	0,00	0,00	1,00	0,00	1,00	1,00
3,00	2,00	2,00	3,00	0,00	1,00	0,00	0,00	5,00
3,00	2,00	3,00	2,00	1,00	0,00	0,00	1,00	5,00
1,00	2,00	1,00	0,00	0,00	0,00	1,00	0,00	3,00
1,00	2,00	3,00	1,00	0,00	1,00	0,00	1,00	4,00
0,00	2,00	0,00	0,00	3,00	1,00	0,00	1,00	3,00
2,00	1,00	1,00	1,00	0,00	1,00	0,00	1,00	3,00
3,00	3,00	2,00	2,00	3,00	1,00	0,00	1,00	5,00
1,00	0,00	1,00	0,00	0,00	1,00	0,00	1,00	2,00
2,00	3,00	3,00	0,00	1,00	1,00	0,00	0,00	4,00
3,00	2,00	1,00	2,00	0,00	2,00	1,00	1,00	4,00
2,00	2,00	2,00	2,00	2,00	1,00	0,00	1,00	4,00
0,00	3,00	0,00	0,00	0,00	0,00	0,00	1,00	3,00
1,00	1,00	1,00	0,00	0,00	1,00	0,00	1,00	2,00
2,00	2,00	1,00	3,00	0,00	2,00	1,00	1,00	3,00
1,00	2,00	2,00	2,00	2,00	1,00	0,00	0,00	4,00
1,00	1,00	2,00	1,00	0,00	1,00	0,00	1,00	3,00
0,00	0,00	0,00	0,00	0,00	0,00	1,00	1,00	1,00
2,00	3,00	2,00	2,00	3,00	1,00	0,00	0,00	5,00
2,00	3,00	3,00	2,00	2,00	2,00	1,00	0,00	5,00
1,00	0,00	2,00	0,00	0,00	2,00	1,00	0,00	2,00
0,00	1,00	1,00	1,00	0,00	2,00	1,00	0,00	3,00
0,00	1,00	0,00	0,00	3,00	1,00	0,00	1,00	4,00
3,00	3,00	3,00	0,00	1,00	1,00	0,00	0,00	5,00
2,00	2,00	2,00	1,00	3,00	0,00	1,00	1,00	4,00
2,00	2,00	1,00	1,00	1,00	0,00	1,00	1,00	3,00
2,00	2,00	1,00	1,00	1,00	1,00	0,00	0,00	3,00
2,00	3,00	2,00	1,00	0,00	1,00	0,00	1,00	5,00
1,00	2,00	1,00	1,00	1,00	1,00	0,00	1,00	3,00
3,00	2,00	3,00	0,00	0,00	1,00	0,00	1,00	4,00
1,00	3,00	2,00	1,00	3,00	2,00	1,00	0,00	4,00
3,00	3,00	3,00	2,00	3,00	1,00	0,00	1,00	5,00
0,00	0,00	1,00	0,00	0,00	1,00	0,00	0,00	2,00
3,00	1,00	1,00	0,00	1,00	1,00	0,00	1,00	4,00
3,00	3,00	2,00	1,00	1,00	1,00	0,00	1,00	5,00
1,00	2,00	1,00	2,00	1,00	1,00	0,00	1,00	3,00
3,00	2,00	1,00	1,00	1,00	2,00	1,00	1,00	4,00
1,00	1,00	2,00	1,00	0,00	1,00	0,00	0,00	2,00
3,00	3,00	2,00	0,00	0,00	1,00	0,00	1,00	4,00
2,00	2,00	2,00	3,00	2,00	0,00	1,00	1,00	5,00
3,00	0,00	3,00	2,00	0,00	2,00	1,00	1,00	4,00
0,00	0,00	1,00	1,00	0,00	2,00	1,00	0,00	1,00
3,00	2,00	2,00	2,00	3,00	0,00	0,00	0,00	5,00
2,00	3,00	3,00	0,00	1,00	1,00	0,00	1,00	5,00
1,00	1,00	1,00	1,00	1,00	1,00	0,00	0,00	3,00
2,00	2,00	2,00	1,00	3,00	2,00	1,00	1,00	5,00
2,00	3,00	2,00	2,00	1,00	1,00	0,00	1,00	4,00

1,00	2,00	1,00	0,00	1,00	0,00	1,00	1,00	3,00
3,00	3,00	3,00	1,00	2,00	1,00	0,00	0,00	5,00
3,00	3,00	3,00	3,00	2,00	1,00	0,00	1,00	5,00
2,00	3,00	2,00	1,00	1,00	0,00	1,00	1,00	5,00
1,00	3,00	3,00	1,00	0,00	1,00	0,00	1,00	4,00
1,00	3,00	3,00	1,00	1,00	2,00	1,00	1,00	3,00
2,00	3,00	1,00	1,00	1,00	0,00	0,00	1,00	4,00
2,00	3,00	2,00	0,00	3,00	0,00	1,00	0,00	4,00
2,00	2,00	2,00	1,00	3,00	2,00	1,00	1,00	5,00
3,00	1,00	1,00	1,00	0,00	0,00	1,00	1,00	4,00
0,00	3,00	1,00	0,00	1,00	2,00	1,00	1,00	3,00
1,00	2,00	1,00	2,00	1,00	0,00	0,00	1,00	3,00
1,00	3,00	3,00	1,00	1,00	0,00	0,00	0,00	3,00
1,00	1,00	2,00	1,00	2,00	1,00	0,00	0,00	4,00
2,00	2,00	2,00	1,00	1,00	1,00	0,00	1,00	4,00
3,00	3,00	3,00	1,00	2,00	2,00	1,00	1,00	4,00
2,00	2,00	1,00	0,00	3,00	0,00	1,00	1,00	4,00
1,00	3,00	1,00	0,00	1,00	1,00	0,00	1,00	2,00
1,00	0,00	0,00	0,00	0,00	1,00	0,00	1,00	2,00
2,00	3,00	2,00	1,00	1,00	2,00	1,00	1,00	4,00
2,00	2,00	1,00	0,00	1,00	1,00	0,00	1,00	3,00
3,00	1,00	2,00	2,00	1,00	1,00	0,00	1,00	3,00
3,00	3,00	1,00	0,00	1,00	2,00	1,00	1,00	4,00
3,00	1,00	3,00	1,00	0,00	0,00	1,00	1,00	4,00
3,00	3,00	3,00	1,00	3,00	1,00	0,00	1,00	5,00
3,00	3,00	3,00	0,00	2,00	0,00	0,00	0,00	5,00
3,00	3,00	3,00	2,00	3,00	0,00	0,00	1,00	5,00
0,00	1,00	1,00	0,00	1,00	0,00	1,00	1,00	3,00
0,00	1,00	2,00	1,00	0,00	0,00	1,00	1,00	2,00
3,00	2,00	3,00	2,00	3,00	1,00	0,00	0,00	5,00
2,00	0,00	2,00	1,00	0,00	0,00	0,00	0,00	3,00
1,00	2,00	0,00	0,00	0,00	1,00	0,00	0,00	2,00
3,00	2,00	2,00	1,00	0,00	2,00	1,00	1,00	4,00
3,00	1,00	2,00	1,00	1,00	2,00	1,00	1,00	4,00
3,00	2,00	2,00	2,00	2,00	1,00	0,00	0,00	5,00
1,00	2,00	1,00	0,00	1,00	1,00	0,00	0,00	3,00
1,00	3,00	2,00	2,00	0,00	0,00	1,00	1,00	4,00
1,00	3,00	3,00	3,00	3,00	0,00	0,00	0,00	5,00
3,00	3,00	3,00	1,00	3,00	1,00	0,00	1,00	5,00
1,00	3,00	2,00	1,00	0,00	0,00	1,00	1,00	4,00
3,00	2,00	3,00	1,00	1,00	1,00	0,00	1,00	5,00
2,00	0,00	1,00	1,00	0,00	2,00	1,00	1,00	2,00
1,00	2,00	0,00	0,00	1,00	2,00	1,00	1,00	3,00
1,00	3,00	0,00	1,00	1,00	1,00	0,00	0,00	3,00
2,00	3,00	1,00	1,00	3,00	1,00	0,00	1,00	5,00
2,00	2,00	2,00	1,00	0,00	1,00	0,00	1,00	4,00
2,00	1,00	1,00	1,00	1,00	1,00	0,00	1,00	3,00
1,00	1,00	1,00	1,00	1,00	1,00	0,00	1,00	3,00
1,00	3,00	3,00	1,00	1,00	1,00	0,00	1,00	5,00
1,00	3,00	1,00	0,00	2,00	1,00	0,00	1,00	3,00

0,00	1,00	1,00	0,00	0,00	2,00	1,00	1,00	2,00
2,00	3,00	2,00	1,00	0,00	0,00	1,00	0,00	4,00
3,00	3,00	2,00	0,00	2,00	0,00	1,00	0,00	5,00
2,00	0,00	3,00	0,00	2,00	1,00	0,00	1,00	3,00
1,00	3,00	2,00	1,00	3,00	1,00	0,00	0,00	4,00
1,00	2,00	1,00	0,00	0,00	2,00	1,00	1,00	3,00
3,00	3,00	3,00	0,00	3,00	1,00	0,00	0,00	5,00
1,00	0,00	0,00	0,00	0,00	0,00	0,00	0,00	1,00
2,00	0,00	1,00	1,00	0,00	1,00	0,00	1,00	3,00
3,00	1,00	1,00	0,00	0,00	1,00	0,00	1,00	4,00
3,00	1,00	3,00	1,00	0,00	2,00	1,00	1,00	4,00
2,00	2,00	1,00	1,00	1,00	2,00	1,00	1,00	3,00
2,00	1,00	1,00	0,00	1,00	1,00	0,00	0,00	3,00
3,00	3,00	3,00	1,00	1,00	1,00	0,00	1,00	4,00
2,00	0,00	0,00	0,00	0,00	1,00	0,00	1,00	2,00
2,00	2,00	0,00	0,00	1,00	2,00	1,00	1,00	3,00
2,00	2,00	1,00	1,00	1,00	1,00	0,00	1,00	3,00
2,00	3,00	1,00	1,00	1,00	1,00	0,00	1,00	4,00
1,00	2,00	3,00	1,00	1,00	1,00	0,00	0,00	4,00
1,00	0,00	1,00	0,00	1,00	1,00	0,00	1,00	3,00
2,00	2,00	3,00	2,00	3,00	2,00	1,00	1,00	5,00
3,00	3,00	3,00	3,00	3,00	0,00	1,00	0,00	5,00
2,00	1,00	2,00	1,00	2,00	1,00	0,00	0,00	4,00
1,00	2,00	1,00	0,00	1,00	1,00	0,00	0,00	3,00
1,00	3,00	3,00	0,00	1,00	1,00	0,00	1,00	4,00
1,00	3,00	1,00	2,00	1,00	2,00	1,00	1,00	4,00
3,00	2,00	2,00	0,00	1,00	2,00	1,00	1,00	3,00
1,00	2,00	2,00	1,00	0,00	1,00	0,00	0,00	4,00
1,00	0,00	1,00	1,00	0,00	1,00	0,00	0,00	2,00
0,00	3,00	3,00	2,00	1,00	1,00	0,00	1,00	5,00
2,00	2,00	1,00	0,00	1,00	2,00	1,00	1,00	4,00
3,00	3,00	2,00	3,00	3,00	1,00	0,00	1,00	5,00
0,00	1,00	1,00	0,00	1,00	1,00	0,00	1,00	3,00
0,00	1,00	2,00	1,00	0,00	2,00	1,00	1,00	3,00
3,00	3,00	2,00	0,00	1,00	1,00	0,00	0,00	4,00
2,00	2,00	2,00	1,00	0,00	2,00	1,00	0,00	3,00
0,00	0,00	0,00	0,00	0,00	2,00	1,00	1,00	1,00
0,00	0,00	1,00	0,00	0,00	1,00	0,00	1,00	1,00
1,00	3,00	1,00	0,00	0,00	1,00	0,00	0,00	4,00
1,00	3,00	2,00	0,00	0,00	0,00	0,00	1,00	4,00
3,00	1,00	3,00	2,00	1,00	1,00	0,00	1,00	5,00
2,00	2,00	3,00	3,00	1,00	2,00	1,00	1,00	5,00
3,00	2,00	2,00	2,00	0,00	1,00	0,00	1,00	5,00
1,00	1,00	1,00	0,00	0,00	1,00	0,00	1,00	3,00
0,00	2,00	0,00	0,00	0,00	2,00	1,00	1,00	2,00
3,00	3,00	3,00	1,00	3,00	1,00	0,00	1,00	5,00
0,00	2,00	1,00	1,00	1,00	1,00	0,00	0,00	4,00
2,00	3,00	1,00	0,00	1,00	2,00	1,00	1,00	4,00
3,00	3,00	3,00	1,00	0,00	1,00	0,00	1,00	5,00
1,00	2,00	3,00	0,00	1,00	1,00	0,00	0,00	4,00

3,00	3,00	3,00	3,00	3,00	0,00	1,00	1,00	5,00
2,00	0,00	0,00	0,00	1,00	2,00	1,00	0,00	2,00
0,00	1,00	1,00	0,00	0,00	2,00	1,00	1,00	2,00
1,00	3,00	3,00	3,00	2,00	0,00	0,00	0,00	5,00
2,00	3,00	1,00	0,00	3,00	0,00	0,00	0,00	5,00
3,00	3,00	2,00	2,00	3,00	0,00	0,00	1,00	5,00
1,00	3,00	1,00	0,00	0,00	0,00	0,00	1,00	4,00
2,00	3,00	3,00	3,00	1,00	0,00	1,00	0,00	5,00
0,00	0,00	3,00	3,00	0,00	0,00	1,00	0,00	4,00
0,00	3,00	2,00	0,00	2,00	2,00	1,00	1,00	4,00
3,00	0,00	2,00	0,00	2,00	1,00	0,00	1,00	4,00
0,00	0,00	1,00	0,00	0,00	1,00	0,00	0,00	1,00
2,00	3,00	2,00	0,00	1,00	1,00	0,00	1,00	4,00
3,00	3,00	1,00	1,00	1,00	1,00	0,00	0,00	4,00
0,00	0,00	0,00	1,00	0,00	1,00	0,00	1,00	1,00
1,00	0,00	0,00	0,00	0,00	2,00	1,00	1,00	1,00
3,00	1,00	3,00	2,00	0,00	2,00	1,00	1,00	4,00
2,00	1,00	1,00	0,00	1,00	0,00	1,00	1,00	3,00
3,00	3,00	3,00	1,00	2,00	1,00	0,00	1,00	5,00
1,00	3,00	2,00	0,00	3,00	1,00	0,00	1,00	4,00
3,00	3,00	3,00	0,00	1,00	1,00	0,00	1,00	4,00
0,00	0,00	0,00	0,00	1,00	0,00	1,00	1,00	1,00
1,00	3,00	3,00	1,00	1,00	0,00	0,00	0,00	4,00
2,00	3,00	2,00	2,00	2,00	2,00	1,00	1,00	5,00
1,00	3,00	0,00	0,00	3,00	1,00	0,00	1,00	4,00
2,00	0,00	0,00	0,00	0,00	1,00	0,00	1,00	1,00
3,00	3,00	1,00	0,00	2,00	1,00	0,00	0,00	3,00
0,00	0,00	0,00	0,00	0,00	1,00	0,00	1,00	1,00
2,00	2,00	2,00	1,00	1,00	0,00	1,00	1,00	4,00
1,00	1,00	1,00	0,00	1,00	1,00	0,00	1,00	3,00
0,00	0,00	0,00	0,00	0,00	1,00	0,00	0,00	1,00
3,00	0,00	1,00	0,00	1,00	1,00	0,00	1,00	3,00
0,00	0,00	1,00	0,00	0,00	2,00	1,00	1,00	2,00
2,00	3,00	2,00	0,00	1,00	2,00	1,00	1,00	5,00
0,00	1,00	0,00	0,00	0,00	0,00	0,00	1,00	2,00
3,00	3,00	3,00	1,00	2,00	0,00	0,00	0,00	5,00
2,00	2,00	3,00	0,00	0,00	1,00	0,00	0,00	3,00
3,00	3,00	3,00	3,00	3,00	2,00	1,00	1,00	5,00
3,00	2,00	1,00	2,00	0,00	2,00	1,00	1,00	4,00
0,00	1,00	1,00	0,00	0,00	2,00	1,00	1,00	2,00
2,00	3,00	1,00	2,00	0,00	2,00	1,00	0,00	4,00
1,00	1,00	1,00	0,00	1,00	2,00	1,00	1,00	2,00
2,00	2,00	3,00	1,00	1,00	2,00	1,00	1,00	4,00
1,00	3,00	1,00	0,00	3,00	2,00	1,00	1,00	4,00
2,00	2,00	3,00	3,00	0,00	1,00	0,00	1,00	4,00
3,00	3,00	3,00	3,00	2,00	1,00	0,00	0,00	5,00
3,00	1,00	2,00	2,00	0,00	0,00	0,00	0,00	3,00
0,00	1,00	1,00	0,00	1,00	0,00	0,00	1,00	3,00
1,00	3,00	3,00	2,00	1,00	0,00	0,00	0,00	5,00
3,00	3,00	3,00	3,00	2,00	2,00	1,00	0,00	5,00

1,00	3,00	3,00	1,00	3,00	0,00	1,00	1,00	5,00
3,00	3,00	3,00	3,00	2,00	1,00	0,00	1,00	5,00
1,00	3,00	1,00	2,00	1,00	1,00	0,00	1,00	5,00
1,00	1,00	1,00	0,00	0,00	2,00	1,00	1,00	3,00
3,00	2,00	1,00	1,00	3,00	1,00	0,00	0,00	4,00
2,00	3,00	2,00	2,00	2,00	1,00	0,00	0,00	5,00
2,00	2,00	3,00	2,00	1,00	2,00	1,00	0,00	5,00
1,00	2,00	1,00	0,00	3,00	1,00	0,00	1,00	4,00
1,00	3,00	2,00	1,00	3,00	1,00	0,00	1,00	5,00
0,00	0,00	0,00	0,00	0,00	0,00	1,00	1,00	1,00
0,00	0,00	1,00	2,00	1,00	2,00	1,00	0,00	3,00
0,00	1,00	0,00	0,00	0,00	1,00	0,00	0,00	1,00
1,00	2,00	0,00	1,00	3,00	1,00	0,00	1,00	4,00
2,00	2,00	2,00	0,00	1,00	1,00	0,00	1,00	4,00
3,00	2,00	2,00	0,00	0,00	1,00	0,00	0,00	4,00
2,00	2,00	2,00	0,00	1,00	1,00	0,00	1,00	4,00
3,00	3,00	3,00	2,00	3,00	1,00	0,00	0,00	5,00
2,00	2,00	3,00	0,00	0,00	0,00	1,00	1,00	4,00
3,00	2,00	1,00	3,00	0,00	0,00	1,00	0,00	5,00
3,00	3,00	1,00	3,00	3,00	2,00	1,00	1,00	5,00
1,00	1,00	1,00	0,00	0,00	1,00	0,00	1,00	2,00
2,00	3,00	1,00	1,00	1,00	1,00	0,00	1,00	4,00
1,00	1,00	2,00	0,00	0,00	1,00	0,00	1,00	3,00
3,00	1,00	3,00	0,00	0,00	2,00	1,00	0,00	4,00
1,00	2,00	0,00	1,00	1,00	0,00	1,00	0,00	4,00
1,00	3,00	1,00	1,00	3,00	1,00	0,00	1,00	4,00
2,00	0,00	1,00	1,00	0,00	0,00	1,00	1,00	2,00
2,00	1,00	1,00	1,00	1,00	1,00	0,00	1,00	3,00
3,00	3,00	3,00	0,00	3,00	1,00	0,00	1,00	5,00
1,00	1,00	2,00	0,00	0,00	2,00	1,00	0,00	2,00
3,00	0,00	1,00	1,00	0,00	1,00	0,00	0,00	4,00
3,00	3,00	2,00	1,00	3,00	1,00	0,00	0,00	5,00
3,00	1,00	1,00	1,00	3,00	1,00	0,00	0,00	4,00
3,00	2,00	3,00	2,00	3,00	1,00	0,00	1,00	5,00
3,00	2,00	1,00	3,00	1,00	2,00	1,00	0,00	4,00
1,00	1,00	3,00	1,00	0,00	0,00	1,00	1,00	3,00
3,00	2,00	1,00	1,00	0,00	1,00	0,00	1,00	4,00
3,00	1,00	1,00	1,00	0,00	1,00	0,00	1,00	3,00
3,00	3,00	3,00	2,00	3,00	1,00	0,00	1,00	5,00
3,00	3,00	2,00	1,00	2,00	2,00	1,00	1,00	5,00
2,00	2,00	1,00	1,00	0,00	0,00	1,00	0,00	3,00
3,00	3,00	3,00	2,00	3,00	2,00	1,00	0,00	5,00
3,00	2,00	3,00	2,00	1,00	1,00	0,00	1,00	5,00
2,00	3,00	0,00	0,00	2,00	1,00	0,00	0,00	4,00
3,00	2,00	2,00	2,00	1,00	2,00	1,00	1,00	4,00
3,00	3,00	3,00	0,00	1,00	1,00	0,00	1,00	4,00
3,00	3,00	2,00	1,00	2,00	0,00	1,00	1,00	4,00
3,00	3,00	3,00	2,00	2,00	0,00	0,00	0,00	5,00
1,00	2,00	0,00	0,00	1,00	2,00	1,00	1,00	2,00
1,00	1,00	2,00	0,00	0,00	0,00	1,00	1,00	2,00

3,00	2,00	2,00	0,00	2,00	1,00	0,00	1,00	4,00
1,00	0,00	0,00	0,00	0,00	0,00	1,00	0,00	1,00
1,00	1,00	2,00	0,00	0,00	0,00	1,00	1,00	2,00
1,00	0,00	0,00	0,00	0,00	1,00	0,00	1,00	1,00
2,00	1,00	2,00	1,00	1,00	1,00	0,00	1,00	3,00
3,00	3,00	3,00	2,00	2,00	1,00	0,00	1,00	5,00
0,00	1,00	2,00	0,00	0,00	2,00	1,00	0,00	2,00
1,00	2,00	3,00	1,00	2,00	2,00	1,00	1,00	5,00
3,00	1,00	2,00	0,00	0,00	2,00	1,00	1,00	4,00
2,00	3,00	1,00	1,00	0,00	1,00	0,00	0,00	4,00
2,00	3,00	3,00	0,00	0,00	1,00	0,00	0,00	4,00
0,00	2,00	2,00	0,00	1,00	0,00	1,00	0,00	3,00
1,00	0,00	0,00	0,00	0,00	0,00	1,00	1,00	2,00
3,00	3,00	1,00	0,00	3,00	0,00	1,00	0,00	5,00
1,00	1,00	1,00	1,00	1,00	2,00	1,00	0,00	4,00
1,00	3,00	1,00	0,00	1,00	1,00	0,00	1,00	4,00
2,00	3,00	3,00	3,00	3,00	0,00	1,00	1,00	5,00
3,00	1,00	1,00	1,00	0,00	1,00	0,00	0,00	3,00
0,00	1,00	3,00	0,00	0,00	2,00	1,00	1,00	3,00
3,00	3,00	3,00	2,00	3,00	2,00	1,00	0,00	5,00
3,00	1,00	1,00	0,00	0,00	0,00	1,00	1,00	3,00
3,00	3,00	3,00	1,00	1,00	1,00	0,00	1,00	5,00
1,00	0,00	1,00	2,00	0,00	2,00	1,00	0,00	3,00
2,00	2,00	2,00	1,00	0,00	1,00	0,00	1,00	4,00
1,00	3,00	3,00	0,00	1,00	1,00	0,00	1,00	4,00
1,00	1,00	3,00	0,00	0,00	1,00	0,00	0,00	3,00
2,00	3,00	1,00	1,00	1,00	1,00	0,00	1,00	3,00
1,00	1,00	1,00	0,00	1,00	1,00	0,00	1,00	3,00
2,00	3,00	2,00	1,00	2,00	0,00	1,00	1,00	5,00
2,00	2,00	3,00	3,00	2,00	2,00	1,00	0,00	5,00
2,00	3,00	2,00	2,00	3,00	1,00	0,00	0,00	5,00
2,00	2,00	3,00	3,00	2,00	1,00	0,00	0,00	5,00
2,00	3,00	1,00	1,00	0,00	1,00	0,00	1,00	3,00
3,00	2,00	1,00	0,00	0,00	2,00	1,00	1,00	3,00
0,00	1,00	0,00	2,00	1,00	2,00	1,00	1,00	3,00
3,00	1,00	1,00	1,00	0,00	1,00	0,00	1,00	4,00
1,00	1,00	1,00	0,00	2,00	0,00	1,00	1,00	3,00
2,00	1,00	3,00	0,00	1,00	0,00	1,00	1,00	4,00
2,00	2,00	1,00	0,00	2,00	1,00	0,00	0,00	3,00
0,00	3,00	1,00	0,00	1,00	1,00	0,00	0,00	3,00
1,00	2,00	2,00	2,00	1,00	2,00	1,00	1,00	4,00
3,00	0,00	1,00	2,00	0,00	1,00	0,00	1,00	3,00
3,00	3,00	1,00	0,00	2,00	2,00	1,00	1,00	4,00
1,00	2,00	2,00	1,00	2,00	1,00	0,00	1,00	4,00
3,00	2,00	1,00	1,00	1,00	0,00	0,00	0,00	4,00
1,00	2,00	2,00	0,00	0,00	2,00	1,00	1,00	3,00
0,00	0,00	0,00	0,00	0,00	0,00	0,00	0,00	1,00
2,00	2,00	3,00	0,00	1,00	2,00	1,00	1,00	3,00
1,00	3,00	2,00	0,00	1,00	2,00	1,00	0,00	4,00
2,00	2,00	2,00	1,00	1,00	0,00	0,00	0,00	4,00

1,00	1,00	1,00	1,00	0,00	2,00	1,00	1,00	2,00
1,00	1,00	3,00	1,00	0,00	0,00	0,00	0,00	3,00
0,00	1,00	1,00	0,00	0,00	2,00	1,00	0,00	2,00
0,00	1,00	1,00	0,00	0,00	1,00	0,00	1,00	1,00
1,00	3,00	1,00	0,00	1,00	1,00	0,00	1,00	3,00
3,00	1,00	1,00	1,00	0,00	0,00	1,00	1,00	3,00
1,00	2,00	2,00	1,00	0,00	1,00	0,00	1,00	3,00
2,00	3,00	2,00	1,00	0,00	1,00	0,00	0,00	4,00
2,00	0,00	2,00	3,00	0,00	1,00	0,00	1,00	3,00
3,00	2,00	3,00	0,00	0,00	1,00	0,00	0,00	4,00
0,00	1,00	1,00	1,00	0,00	1,00	0,00	0,00	2,00
0,00	1,00	0,00	1,00	0,00	2,00	1,00	0,00	2,00
0,00	1,00	1,00	0,00	0,00	1,00	0,00	1,00	2,00
1,00	2,00	1,00	2,00	1,00	2,00	1,00	1,00	3,00
1,00	3,00	3,00	2,00	1,00	2,00	1,00	0,00	5,00
1,00	1,00	0,00	0,00	0,00	1,00	0,00	1,00	2,00
3,00	3,00	3,00	2,00	3,00	0,00	0,00	0,00	5,00
0,00	1,00	2,00	0,00	1,00	0,00	0,00	0,00	3,00
2,00	3,00	1,00	0,00	2,00	1,00	0,00	1,00	4,00
1,00	0,00	1,00	1,00	0,00	2,00	1,00	0,00	2,00
3,00	0,00	2,00	1,00	0,00	0,00	1,00	0,00	3,00
2,00	1,00	1,00	1,00	1,00	1,00	0,00	1,00	3,00
0,00	0,00	1,00	0,00	0,00	1,00	0,00	1,00	2,00
3,00	3,00	1,00	1,00	0,00	1,00	0,00	1,00	3,00
0,00	1,00	1,00	0,00	3,00	0,00	1,00	1,00	3,00
3,00	3,00	2,00	3,00	3,00	1,00	0,00	0,00	5,00
3,00	1,00	2,00	3,00	2,00	1,00	0,00	0,00	4,00
1,00	1,00	3,00	1,00	1,00	0,00	0,00	0,00	3,00
2,00	1,00	2,00	1,00	0,00	2,00	1,00	0,00	3,00
1,00	2,00	3,00	1,00	1,00	1,00	0,00	1,00	4,00
3,00	1,00	3,00	0,00	0,00	1,00	0,00	1,00	3,00
3,00	1,00	2,00	0,00	1,00	1,00	0,00	1,00	4,00
2,00	1,00	2,00	1,00	1,00	1,00	0,00	0,00	3,00
2,00	0,00	2,00	1,00	0,00	2,00	1,00	1,00	3,00
2,00	1,00	1,00	1,00	1,00	1,00	0,00	1,00	4,00
1,00	0,00	1,00	1,00	0,00	2,00	1,00	0,00	2,00
1,00	3,00	2,00	1,00	1,00	1,00	0,00	1,00	4,00
1,00	2,00	3,00	2,00	3,00	1,00	0,00	0,00	5,00
3,00	1,00	3,00	3,00	1,00	0,00	0,00	0,00	5,00
3,00	3,00	3,00	3,00	3,00	2,00	1,00	0,00	5,00
1,00	2,00	2,00	2,00	2,00	0,00	1,00	1,00	4,00
1,00	2,00	0,00	1,00	1,00	2,00	1,00	1,00	2,00
1,00	1,00	0,00	1,00	0,00	2,00	1,00	0,00	3,00
1,00	1,00	1,00	2,00	1,00	1,00	0,00	0,00	4,00
3,00	0,00	2,00	2,00	0,00	2,00	1,00	0,00	4,00
3,00	0,00	2,00	1,00	0,00	2,00	1,00	0,00	4,00
1,00	0,00	0,00	0,00	0,00	0,00	0,00	1,00	2,00
2,00	1,00	3,00	2,00	1,00	2,00	1,00	0,00	4,00
3,00	3,00	2,00	1,00	0,00	1,00	0,00	1,00	5,00
3,00	1,00	1,00	0,00	1,00	2,00	1,00	0,00	3,00

1,00	1,00	1,00	1,00	1,00	0,00	0,00	0,00	3,00
2,00	2,00	2,00	3,00	1,00	1,00	0,00	1,00	5,00
3,00	2,00	2,00	1,00	0,00	1,00	0,00	0,00	4,00
3,00	3,00	3,00	2,00	2,00	1,00	0,00	1,00	5,00
1,00	1,00	1,00	0,00	0,00	1,00	0,00	1,00	3,00
1,00	3,00	1,00	0,00	3,00	0,00	1,00	1,00	4,00
1,00	3,00	2,00	2,00	0,00	2,00	1,00	0,00	5,00
3,00	1,00	0,00	0,00	0,00	1,00	0,00	1,00	3,00
1,00	1,00	1,00	0,00	0,00	1,00	0,00	0,00	2,00
3,00	2,00	2,00	2,00	2,00	0,00	0,00	1,00	5,00
1,00	2,00	1,00	0,00	0,00	1,00	0,00	1,00	2,00
0,00	3,00	3,00	2,00	1,00	1,00	0,00	0,00	4,00
1,00	1,00	3,00	1,00	0,00	1,00	0,00	0,00	3,00
0,00	0,00	2,00	3,00	3,00	0,00	0,00	0,00	4,00
3,00	2,00	3,00	2,00	2,00	1,00	0,00	0,00	5,00
1,00	2,00	2,00	1,00	2,00	1,00	0,00	0,00	4,00
2,00	2,00	1,00	1,00	2,00	2,00	1,00	0,00	4,00
1,00	1,00	1,00	1,00	0,00	0,00	1,00	0,00	2,00
3,00	2,00	1,00	0,00	1,00	1,00	0,00	1,00	4,00
1,00	1,00	3,00	3,00	0,00	1,00	0,00	0,00	4,00
3,00	3,00	3,00	3,00	3,00	0,00	0,00	0,00	5,00
1,00	1,00	1,00	1,00	0,00	1,00	0,00	1,00	2,00
1,00	0,00	2,00	0,00	0,00	2,00	1,00	1,00	2,00
1,00	2,00	3,00	3,00	3,00	1,00	0,00	1,00	5,00
1,00	0,00	2,00	1,00	0,00	1,00	0,00	1,00	2,00
2,00	3,00	2,00	1,00	1,00	1,00	0,00	1,00	4,00
2,00	1,00	3,00	1,00	0,00	2,00	1,00	0,00	4,00
2,00	3,00	3,00	1,00	1,00	0,00	0,00	0,00	4,00
0,00	0,00	0,00	1,00	0,00	0,00	1,00	0,00	2,00
3,00	1,00	1,00	1,00	1,00	0,00	0,00	0,00	3,00
2,00	1,00	1,00	2,00	0,00	2,00	1,00	0,00	3,00
0,00	1,00	1,00	1,00	1,00	2,00	1,00	0,00	3,00
0,00	0,00	1,00	0,00	1,00	2,00	1,00	0,00	3,00
3,00	1,00	1,00	0,00	0,00	1,00	0,00	1,00	3,00
3,00	3,00	3,00	3,00	1,00	0,00	0,00	1,00	5,00
0,00	1,00	2,00	0,00	1,00	1,00	0,00	0,00	3,00
3,00	1,00	1,00	1,00	2,00	0,00	0,00	0,00	4,00
2,00	3,00	3,00	1,00	1,00	1,00	0,00	0,00	4,00
2,00	0,00	1,00	1,00	1,00	2,00	1,00	0,00	3,00
2,00	1,00	2,00	2,00	0,00	1,00	0,00	1,00	3,00
0,00	2,00	1,00	1,00	0,00	2,00	1,00	0,00	3,00
3,00	2,00	2,00	1,00	2,00	0,00	0,00	1,00	4,00
3,00	2,00	2,00	0,00	1,00	2,00	1,00	0,00	4,00
0,00	0,00	0,00	0,00	0,00	0,00	1,00	0,00	1,00
1,00	2,00	1,00	1,00	0,00	2,00	1,00	0,00	4,00
3,00	2,00	3,00	2,00	1,00	0,00	1,00	0,00	5,00
2,00	1,00	1,00	1,00	0,00	2,00	1,00	0,00	3,00
3,00	3,00	3,00	2,00	1,00	1,00	0,00	0,00	5,00
2,00	3,00	3,00	3,00	3,00	0,00	0,00	1,00	5,00
0,00	0,00	0,00	0,00	1,00	1,00	0,00	0,00	2,00

2,00	3,00	2,00	2,00	3,00	1,00	0,00	0,00	5,00
1,00	1,00	3,00	1,00	0,00	1,00	0,00	0,00	3,00
2,00	1,00	1,00	0,00	0,00	0,00	0,00	0,00	2,00
3,00	0,00	2,00	0,00	3,00	1,00	0,00	1,00	4,00
0,00	1,00	1,00	2,00	0,00	1,00	0,00	0,00	2,00
1,00	1,00	1,00	0,00	0,00	1,00	0,00	0,00	2,00
1,00	1,00	1,00	0,00	1,00	0,00	1,00	0,00	3,00
0,00	0,00	3,00	3,00	1,00	0,00	0,00	0,00	3,00
1,00	1,00	1,00	0,00	0,00	1,00	0,00	1,00	2,00
1,00	1,00	0,00	1,00	1,00	1,00	0,00	1,00	3,00
3,00	1,00	1,00	0,00	0,00	2,00	1,00	0,00	3,00
3,00	2,00	2,00	1,00	0,00	1,00	0,00	0,00	4,00
0,00	0,00	1,00	1,00	1,00	1,00	0,00	1,00	2,00
1,00	1,00	2,00	0,00	0,00	1,00	0,00	1,00	2,00
0,00	0,00	1,00	1,00	0,00	2,00	1,00	0,00	2,00
2,00	1,00	3,00	2,00	0,00	1,00	0,00	0,00	4,00
2,00	3,00	1,00	1,00	3,00	0,00	1,00	1,00	5,00
0,00	1,00	1,00	0,00	0,00	0,00	1,00	1,00	3,00
0,00	1,00	1,00	1,00	0,00	2,00	1,00	0,00	2,00
2,00	2,00	3,00	1,00	0,00	1,00	0,00	1,00	4,00
1,00	0,00	0,00	0,00	2,00	2,00	1,00	0,00	3,00
2,00	2,00	1,00	3,00	1,00	1,00	0,00	0,00	4,00
3,00	3,00	1,00	2,00	1,00	1,00	0,00	0,00	5,00
0,00	1,00	0,00	0,00	0,00	1,00	0,00	1,00	2,00
2,00	3,00	3,00	1,00	1,00	0,00	1,00	1,00	5,00
1,00	1,00	1,00	0,00	0,00	1,00	0,00	1,00	2,00
1,00	2,00	0,00	1,00	0,00	1,00	0,00	1,00	2,00
0,00	0,00	0,00	0,00	0,00	2,00	1,00	0,00	2,00
2,00	0,00	1,00	0,00	0,00	1,00	0,00	0,00	4,00
2,00	1,00	3,00	1,00	1,00	1,00	0,00	0,00	3,00
3,00	1,00	3,00	2,00	0,00	1,00	0,00	0,00	3,00
3,00	1,00	0,00	2,00	0,00	1,00	0,00	0,00	3,00
1,00	2,00	1,00	2,00	1,00	2,00	1,00	1,00	3,00
2,00	3,00	1,00	1,00	1,00	0,00	0,00	0,00	4,00
1,00	2,00	3,00	0,00	1,00	0,00	0,00	1,00	4,00
3,00	3,00	3,00	0,00	1,00	2,00	1,00	0,00	4,00
2,00	3,00	2,00	2,00	0,00	2,00	1,00	1,00	4,00
0,00	1,00	0,00	2,00	0,00	0,00	0,00	0,00	2,00
3,00	1,00	2,00	2,00	0,00	0,00	0,00	0,00	4,00
1,00	3,00	2,00	0,00	2,00	1,00	0,00	1,00	4,00
1,00	2,00	1,00	1,00	1,00	1,00	0,00	1,00	4,00
1,00	1,00	1,00	0,00	0,00	2,00	1,00	0,00	2,00
2,00	3,00	2,00	1,00	2,00	1,00	0,00	1,00	5,00
3,00	1,00	1,00	0,00	0,00	2,00	1,00	1,00	2,00
0,00	2,00	3,00	2,00	3,00	0,00	0,00	0,00	5,00
2,00	3,00	2,00	1,00	3,00	2,00	1,00	1,00	5,00
0,00	2,00	3,00	3,00	1,00	0,00	0,00	0,00	5,00
1,00	2,00	1,00	0,00	0,00	0,00	1,00	1,00	3,00
0,00	2,00	2,00	1,00	1,00	1,00	0,00	1,00	3,00
3,00	3,00	3,00	2,00	2,00	0,00	0,00	0,00	5,00

0,00	1,00	2,00	1,00	1,00	2,00	1,00	1,00	3,00
0,00	0,00	0,00	0,00	0,00	2,00	1,00	1,00	1,00
3,00	2,00	3,00	1,00	3,00	1,00	0,00	0,00	5,00
0,00	0,00	1,00	0,00	0,00	1,00	0,00	1,00	2,00
3,00	1,00	2,00	0,00	2,00	1,00	0,00	0,00	4,00
1,00	2,00	0,00	1,00	0,00	1,00	0,00	1,00	3,00
3,00	1,00	1,00	2,00	1,00	1,00	0,00	0,00	3,00
3,00	1,00	3,00	2,00	0,00	1,00	0,00	1,00	4,00
0,00	1,00	0,00	0,00	0,00	1,00	0,00	0,00	3,00
3,00	3,00	1,00	2,00	2,00	1,00	0,00	0,00	4,00
3,00	3,00	3,00	2,00	0,00	1,00	0,00	0,00	5,00
3,00	3,00	2,00	0,00	1,00	1,00	0,00	1,00	4,00
3,00	3,00	3,00	3,00	3,00	1,00	0,00	0,00	5,00
0,00	1,00	1,00	1,00	0,00	1,00	0,00	0,00	2,00
1,00	1,00	1,00	2,00	1,00	0,00	1,00	0,00	3,00
1,00	0,00	1,00	0,00	0,00	1,00	0,00	1,00	2,00
1,00	1,00	1,00	2,00	0,00	1,00	0,00	1,00	2,00
3,00	3,00	3,00	0,00	1,00	0,00	0,00	0,00	5,00
1,00	1,00	1,00	0,00	0,00	1,00	0,00	1,00	2,00
1,00	2,00	1,00	1,00	0,00	1,00	0,00	1,00	3,00
1,00	0,00	0,00	0,00	3,00	2,00	1,00	0,00	2,00
3,00	2,00	2,00	2,00	0,00	1,00	0,00	1,00	4,00
3,00	2,00	3,00	2,00	2,00	2,00	1,00	1,00	5,00
1,00	1,00	1,00	2,00	0,00	2,00	1,00	0,00	3,00
2,00	2,00	3,00	3,00	0,00	0,00	0,00	1,00	4,00
0,00	0,00	1,00	1,00	0,00	2,00	1,00	0,00	2,00
0,00	1,00	1,00	1,00	0,00	0,00	1,00	1,00	2,00
2,00	1,00	2,00	1,00	0,00	0,00	0,00	0,00	3,00
3,00	3,00	1,00	1,00	2,00	1,00	0,00	0,00	4,00
2,00	1,00	1,00	1,00	0,00	2,00	1,00	0,00	2,00
1,00	2,00	3,00	1,00	1,00	0,00	0,00	0,00	4,00

pub_b	per_b	self_b	SW_B	PHQ	AGE	SEX	Zpub_b	Zper_b
56,00	24,00	17,00	19,00	3,00	44	2	0,20727	-0,01838
66,00	31,00	44,00	3,00	18,00	52	1	0,80133	0,59160
58,00	21,00	23,00	25,00	10,00	53	1	0,32608	-0,27980
12,00	10,00	20,00	10,00	13,00	35	1	-2,40657	-1,23834
42,00	42,00	39,00	12,00	11,00	29	2	-0,62441	1,55014
58,00	18,00	25,00	24,00	16,00	23	1	0,32608	-0,54122
60,00	32,00	19,00	6,00	8,00	37	1	0,44489	0,67874
60,00	20,00	24,00	14,00	16,00	44	1	0,44489	-0,36694
44,00	11,00	19,00	15,00	17,00	33	1	-0,50560	-1,15120
13,00	10,00	13,00	26,00	5,00	34	1	-2,34717	-1,23834
74,00	17,00	15,00	20,00	4,00	42	2	1,27657	-0,62836
80,00	11,00	14,00	5,00	20,00	27	1	1,63300	-1,15120
55,00	39,00	53,00	2,00	15,00	32	1	0,14787	1,28872
77,00	27,00	45,00	2,00	21,00	29	1	1,45479	0,24304
46,00	23,00	15,00	28,00	9,00	21	1	-0,38678	-0,10552
32,00	17,00	15,00	7,00	10,00	20	1	-1,21846	-0,62836
35,00	50,00	43,00	9,00	10,00	31	1	-1,04024	2,24727
50,00	31,00	32,00	5,00	23,00	18	1	-0,14916	0,59160
60,00	30,00	38,00	19,00	13,00	22	2	0,44489	0,50446
48,00	11,00	10,00	28,00	2,00	21	1	-0,26797	-1,15120
54,00	28,00	19,00	14,00	9,00	26	1	0,08846	0,33018
54,00	16,00	17,00	26,00	7,00	58	1	0,08846	-0,71550
31,00	21,00	29,00	19,00	19,00	71	2	-1,27787	-0,27980
64,00	31,00	49,00	11,00	19,00	53	2	0,68252	0,59160
51,00	24,00	40,00	9,00	12,00	45	1	-0,08976	-0,01838
35,00	14,00	15,00	11,00	13,00	22	1	-1,04024	-0,88978
62,00	23,00	20,00	15,00	6,00	27	2	0,56370	-0,10552
10,00	10,00	10,00	23,00	2,00	33	1	-2,52538	-1,23834
34,00	22,00	22,00	12,00	21,00	63	1	-1,09965	-0,19266
45,00	19,00	22,00	12,00	12,00	22	1	-0,44619	-0,45408
61,00	24,00	37,00	9,00	18,00	53	1	0,50430	-0,01838
54,00	29,00	27,00	16,00	15,00	48	2	0,08846	0,41732
62,00	20,00	31,00	10,00	15,00	42	1	0,56370	-0,36694
64,00	21,00	31,00	2,00	23,00	51	1	0,68252	-0,27980
54,00	27,00	50,00	12,00	20,00	24	1	0,08846	0,24304
49,00	51,00	30,00	17,00	18,00	53	1	-0,20857	2,33441
55,00	21,00	16,00	22,00	7,00	19	1	0,14787	-0,27980
26,00	30,00	32,00	9,00	24,00	49	2	-1,57489	0,50446
67,00	14,00	14,00	15,00	7,00	61	1	0,86073	-0,88978
52,00	28,00	18,00	12,00	12,00	48	2	-0,03035	0,33018
36,00	43,00	33,00	8,00	11,00	50	2	-0,98084	1,63728
27,00	11,00	11,00	25,00	5,00	28	1	-1,51549	-1,15120
13,00	14,00	10,00	27,00	2,00	43	1	-2,34717	-0,88978
62,00	18,00	18,00	21,00	17,00	61	1	0,56370	-0,54122
15,00	10,00	12,00	22,00	8,00	73	1	-2,22836	-1,23834
36,00	13,00	19,00	13,00	5,00	36	1	-0,98084	-0,97692
62,00	28,00	30,00	21,00	5,00	49	1	0,56370	0,33018
63,00	22,00	21,00	9,00	22,00	61	1	0,62311	-0,19266
51,00	47,00	56,00	19,00	18,00	33	1	-0,08976	1,98585

64,00	18,00	28,00	18,00	12,00	30	1	0,68252	-0,54122
90,00	43,00	32,00	20,00	14,00	59	1	2,22706	1,63728
58,00	19,00	17,00	4,00	26,00	18	2	0,32608	-0,45408
37,00	37,00	66,00	9,00	20,00	51	2	-0,92143	1,11444
28,00	10,00	54,00	26,00	2,00	45	1	-1,45608	-1,23834
22,00	20,00	22,00	20,00	9,00	51	1	-1,81252	-0,36694
52,00	26,00	24,00	23,00	17,00	49	1	-0,03035	0,15590
50,00	13,00	19,00	14,00	10,00	47	1	-0,14916	-0,97692
41,00	47,00	22,00	17,00	12,00	54	1	-0,68381	1,98585
22,00	21,00	45,00	7,00	21,00	18	2	-1,81252	-0,27980
42,00	50,00	47,00	4,00	17,00	26	1	-0,62441	2,24727
24,00	11,00	17,00	14,00	17,00	26	2	-1,69371	-1,15120
43,00	18,00	19,00	20,00	9,00	33	1	-0,56500	-0,54122
60,00	18,00	14,00	24,00	15,00	46	1	0,44489	-0,54122
55,00	37,00	52,00	4,00	19,00	21	1	0,14787	1,11444
34,00	15,00	18,00	14,00	10,00	53	1	-1,09965	-0,80264
66,00	32,00	11,00	25,00	2,00	49	2	0,80133	0,67874
49,00	16,00	31,00	9,00	13,00	45	2	-0,20857	-0,71550
73,00	28,00	24,00	5,00	13,00	38	1	1,21717	0,33018
59,00	18,00	15,00	21,00	12,00	41	1	0,38549	-0,54122
24,00	17,00	11,00	14,00	18,00	53	2	-1,69371	-0,62836
74,00	10,00	10,00	20,00	2,00	57	1	1,27657	-1,23834
34,00	10,00	10,00	25,00	7,00	29	1	-1,09965	-1,23834
57,00	22,00	21,00	15,00	14,00	32	1	0,26668	-0,19266
45,00	50,00	47,00	11,00	18,00	25	2	-0,44619	2,24727
50,00	10,00	10,00	23,00	9,00	38	1	-0,14916	-1,23834
79,00	12,00	30,00	2,00	22,00	19	1	1,57360	-1,06406
84,00	18,00	37,00	3,00	25,00	28	1	1,87063	-0,54122
61,00	36,00	11,00	23,00	4,00	23	1	0,50430	1,02730
66,00	39,00	32,00	21,00	11,00	26	1	0,80133	1,28872
47,00	17,00	12,00	9,00	14,00	20	1	-0,32738	-0,62836
32,00	28,00	25,00	9,00	15,00	21	1	-1,21846	0,33018
57,00	36,00	19,00	7,00	22,00	51	1	0,26668	1,02730
61,00	30,00	19,00	13,00	6,00	35	2	0,50430	0,50446
42,00	32,00	26,00	12,00	16,00	19	1	-0,62441	0,67874
62,00	27,00	13,00	29,00	3,00	30	1	0,56370	0,24304
55,00	10,00	10,00	25,00	3,00	32	1	0,14787	-1,23834
73,00	20,00	21,00	8,00	23,00	50	1	1,21717	-0,36694
30,00	10,00	10,00	17,00	13,00	56	2	-1,33727	-1,23834
62,00	10,00	11,00	15,00	10,00	42	1	0,56370	-1,23834
43,00	15,00	23,00	5,00	11,00	18	1	-0,56500	-0,80264
50,00	23,00	16,00	15,00	10,00	30	1	-0,14916	-0,10552
59,00	17,00	21,00	11,00	16,00	32	1	0,38549	-0,62836
73,00	47,00	16,00	11,00	10,00	52	1	1,21717	1,98585
45,00	30,00	42,00	14,00	19,00	40	1	-0,44619	0,50446
52,00	59,00	65,00	12,00	22,00	32	1	-0,03035	3,03153
38,00	27,00	43,00	10,00	22,00	32	1	-0,86203	0,24304
42,00	32,00	26,00	12,00	17,00	40	2	-0,62441	0,67874
71,00	41,00	59,00	2,00	17,00	18	1	1,09835	1,46300
69,00	32,00	40,00	3,00	18,00	40	1	0,97954	0,67874

46,00	17,00	13,00	18,00	10,00	65	1	-0,38678	-0,62836
23,00	16,00	22,00	12,00	18,00	21	1	-1,75311	-0,71550
27,00	18,00	17,00	18,00	22,00	57	1	-1,51549	-0,54122
63,00	35,00	31,00	8,00	17,00	25	1	0,62311	0,94016
36,00	10,00	10,00	25,00	11,00	53	1	-0,98084	-1,23834
56,00	15,00	15,00	15,00	10,00	18	1	0,20727	-0,80264
50,00	16,00	18,00	13,00	18,00	44	1	-0,14916	-0,71550
67,00	24,00	18,00	15,00	13,00	28	1	0,86073	-0,01838
59,00	14,00	24,00	5,00	16,00	19	1	0,38549	-0,88978
45,00	32,00	25,00	23,00	14,00	28	1	-0,44619	0,67874
56,00	45,00	32,00	11,00	22,00	18	1	0,20727	1,81156
67,00	32,00	20,00	12,00	15,00	34	1	0,86073	0,67874
19,00	19,00	16,00	20,00	5,00	67	1	-1,99073	-0,45408
50,00	27,00	20,00	19,00	9,00	24	1	-0,14916	0,24304
56,00	16,00	19,00	3,00	17,00	29	1	0,20727	-0,71550
69,00	27,00	21,00	22,00	19,00	36	1	0,97954	0,24304
45,00	22,00	31,00	17,00	10,00	59	2	-0,44619	-0,19266
64,00	33,00	28,00	19,00	9,00	55	1	0,68252	0,76588
16,00	10,00	10,00	25,00	4,00	33	1	-2,16895	-1,23834
64,00	22,00	20,00	19,00	22,00	39	1	0,68252	-0,19266
37,00	19,00	23,00	16,00	12,00	67	1	-0,92143	-0,45408
17,00	16,00	14,00	18,00	19,00	21	1	-2,10954	-0,71550
64,00	24,00	35,00	9,00	22,00	18	1	0,68252	-0,01838
37,00	14,00	17,00	15,00	22,00	49	1	-0,92143	-0,88978
52,00	16,00	28,00	7,00	16,00	19	2	-0,03035	-0,71550
33,00	14,00	34,00	7,00	23,00	33	1	-1,15906	-0,88978
56,00	28,00	13,00	4,00	19,00	19	1	0,20727	0,33018
61,00	22,00	12,00	19,00	15,00	41	1	0,50430	-0,19266
57,00	31,00	17,00	22,00	9,00	43	2	0,26668	0,59160
75,00	35,00	39,00	1,00	23,00	39	1	1,33598	0,94016
46,00	11,00	10,00	10,00	16,00	36	1	-0,38678	-1,15120
56,00	29,00	19,00	21,00	8,00	38	1	0,20727	0,41732
18,00	10,00	10,00	20,00	6,00	43	1	-2,05014	-1,23834
73,00	28,00	26,00	30,00	2,00	50	1	1,21717	0,33018
33,00	17,00	12,00	13,00	12,00	36	1	-1,15906	-0,62836
72,00	20,00	52,00	3,00	14,00	50	1	1,15776	-0,36694
47,00	15,00	11,00	29,00	1,00	35	1	-0,32738	-0,80264
59,00	23,00	30,00	20,00	4,00	43	2	0,38549	-0,10552
47,00	19,00	15,00	5,00	13,00	21	1	-0,32738	-0,45408
52,00	10,00	22,00	22,00	12,00	45	1	-0,03035	-1,23834
63,00	18,00	30,00	15,00	11,00	57	2	0,62311	-0,54122
17,00	13,00	15,00	26,00	2,00	52	1	-2,10954	-0,97692
66,00	28,00	49,00	13,00	14,00	32	1	0,80133	0,33018
60,00	21,00	40,00	3,00	22,00	19	1	0,44489	-0,27980
35,00	46,00	69,00	11,00	16,00	24	2	-1,04024	1,89871
31,00	10,00	10,00	7,00	18,00	54	1	-1,27787	-1,23834
33,00	16,00	11,00	18,00	23,00	47	2	-1,15906	-0,71550
65,00	48,00	50,00	9,00	21,00	25	1	0,74192	2,07299
58,00	16,00	13,00	19,00	5,00	22	1	0,32608	-0,71550
74,00	25,00	35,00	24,00	15,00	56	1	1,27657	0,06876

58,00	17,00	19,00	25,00	6,00	61	1	0,32608	-0,62836
76,00	10,00	19,00	22,00	4,00	56	1	1,39538	-1,23834
10,00	10,00	12,00	19,00	5,00	60	2	-2,52538	-1,23834
69,00	31,00	48,00	10,00	8,00	24	1	0,97954	0,59160
57,00	21,00	24,00	28,00	17,00	29	1	0,26668	-0,27980
14,00	13,00	12,00	9,00	13,00	21	1	-2,28776	-0,97692
61,00	17,00	11,00	20,00	3,00	33	1	0,50430	-0,62836
57,00	16,00	12,00	10,00	18,00	52	1	0,26668	-0,71550
36,00	12,00	11,00	26,00	10,00	62	2	-0,98084	-1,06406
59,00	28,00	11,00	19,00	10,00	51	1	0,38549	0,33018
61,00	28,00	36,00	13,00	11,00	26	1	0,50430	0,33018
56,00	28,00	53,00	10,00	14,00	20	1	0,20727	0,33018
55,00	24,00	19,00	20,00	4,00	24	2	0,14787	-0,01838
54,00	22,00	20,00	24,00	15,00	35	2	0,08846	-0,19266
47,00	15,00	10,00	18,00	11,00	23	1	-0,32738	-0,80264
70,00	40,00	56,00	4,00	21,00	55	1	1,03895	1,37586
42,00	19,00	18,00	13,00	12,00	36	1	-0,62441	-0,45408
28,00	21,00	25,00	13,00	24,00	55	1	-1,45608	-0,27980
54,00	47,00	27,00	10,00	11,00	52	1	0,08846	1,98585
12,00	10,00	52,00	0,00	22,00	18	1	-2,40657	-1,23834
50,00	12,00	22,00	7,00	14,00	23	1	-0,14916	-1,06406
41,00	24,00	45,00	15,00	15,00	27	1	-0,68381	-0,01838
41,00	17,00	38,00	7,00	22,00	32	1	-0,68381	-0,62836
51,00	11,00	14,00	8,00	11,00	37	1	-0,08976	-1,15120
67,00	32,00	54,00	16,00	20,00	55	2	0,86073	0,67874
38,00	22,00	14,00	26,00	17,00	61	1	-0,86203	-0,19266
69,00	16,00	23,00	10,00	12,00	19	1	0,97954	-0,71550
59,00	33,00	38,00	13,00	19,00	33	1	0,38549	0,76588
70,00	16,00	35,00	7,00	22,00	23	1	1,03895	-0,71550
30,00	49,00	50,00	14,00	19,00	20	1	-1,33727	2,16013
56,00	12,00	13,00	28,00	4,00	48	1	0,20727	-1,06406
50,00	15,00	37,00	9,00	19,00	49	1	-0,14916	-0,80264
53,00	30,00	29,00	7,00	15,00	24	1	0,02905	0,50446
47,00	34,00	25,00	17,00	7,00	65	1	-0,32738	0,85302
70,00	12,00	24,00	21,00	6,00	19	1	1,03895	-1,06406
38,00	39,00	42,00	12,00	7,00	41	2	-0,86203	1,28872
63,00	26,00	28,00	11,00	12,00	29	1	0,62311	0,15590
59,00	18,00	21,00	4,00	20,00	49	1	0,38549	-0,54122
58,00	25,00	27,00	7,00	16,00	20	1	0,32608	0,06876
36,00	41,00	20,00	13,00	16,00	39	1	-0,98084	1,46300
49,00	21,00	23,00	15,00	11,00	28	1	-0,20857	-0,27980
56,00	12,00	15,00	13,00	12,00	50	1	0,20727	-1,06406
22,00	20,00	57,00	8,00	24,00	55	2	-1,81252	-0,36694
25,00	11,00	12,00	4,00	19,00	44	1	-1,63430	-1,15120
90,00	13,00	25,00	3,00	22,00	18	1	2,22706	-0,97692
36,00	20,00	21,00	22,00	17,00	61	1	-0,98084	-0,36694
69,00	45,00	28,00	23,00	21,00	40	2	0,97954	1,81156
37,00	39,00	53,00	7,00	17,00	18	1	-0,92143	1,28872
54,00	25,00	17,00	18,00	12,00	30	1	0,08846	0,06876
65,00	24,00	38,00	2,00	22,00	44	2	0,74192	-0,01838

90,00	62,00	81,00	0,00	27,00	38	1	2,22706	3,29295
66,00	25,00	32,00	6,00	17,00	34	1	0,80133	0,06876
37,00	30,00	23,00	14,00	18,00	39	1	-0,92143	0,50446
58,00	36,00	34,00	13,00	13,00	41	2	0,32608	1,02730
43,00	41,00	36,00	3,00	23,00	23	1	-0,56500	1,46300
20,00	15,00	38,00	8,00	16,00	21	1	-1,93133	-0,80264
83,00	39,00	39,00	8,00	19,00	47	1	1,81122	1,28872
67,00	28,00	29,00	12,00	14,00	34	1	0,86073	0,33018
45,00	40,00	71,00	9,00	21,00	32	1	-0,44619	1,37586
63,00	11,00	14,00	9,00	15,00	44	1	0,62311	-1,15120
68,00	38,00	39,00	5,00	21,00	26	1	0,92014	1,20158
53,00	28,00	29,00	25,00	8,00	59	2	0,02905	0,33018
65,00	26,00	35,00	3,00	22,00	20	1	0,74192	0,15590
58,00	26,00	42,00	8,00	20,00	38	1	0,32608	0,15590
27,00	13,00	14,00	1,00	23,00	20	1	-1,51549	-0,97692
90,00	30,00	39,00	10,00	24,00	33	1	2,22706	0,50446
46,00	11,00	11,00	15,00	10,00	53	1	-0,38678	-1,15120
50,00	10,00	18,00	9,00	15,00	32	1	-0,14916	-1,23834
10,00	10,00	11,00	26,00	15,00	27	1	-2,52538	-1,23834
63,00	40,00	38,00	5,00	20,00	30	1	0,62311	1,37586
42,00	16,00	11,00	24,00	9,00	26	1	-0,62441	-0,71550
41,00	16,00	33,00	5,00	22,00	52	1	-0,68381	-0,71550
53,00	62,00	57,00	14,00	16,00	27	1	0,02905	3,29295
68,00	25,00	14,00	10,00	13,00	25	1	0,92014	0,06876
63,00	27,00	28,00	13,00	7,00	54	1	0,62311	0,24304
26,00	31,00	31,00	13,00	21,00	18	1	-1,57489	0,59160
70,00	47,00	34,00	7,00	22,00	27	2	1,03895	1,98585
81,00	22,00	43,00	9,00	22,00	21	1	1,69241	-0,19266
63,00	21,00	15,00	11,00	19,00	37	1	0,62311	-0,27980
70,00	42,00	43,00	13,00	20,00	38	2	1,03895	1,55014
65,00	26,00	30,00	15,00	17,00	41	1	0,74192	0,15590
55,00	22,00	20,00	21,00	2,00	38	2	0,14787	-0,19266
61,00	14,00	11,00	24,00	9,00	29	1	0,50430	-0,88978
76,00	17,00	40,00	18,00	8,00	53	1	1,39538	-0,62836
14,00	18,00	24,00	2,00	27,00	27	1	-2,28776	-0,54122
44,00	20,00	16,00	12,00	10,00	25	1	-0,50560	-0,36694
61,00	23,00	17,00	15,00	17,00	31	1	0,50430	-0,10552
26,00	16,00	27,00	16,00	9,00	46	2	-1,57489	-0,71550
63,00	37,00	30,00	7,00	14,00	20	1	0,62311	1,11444
75,00	22,00	50,00	9,00	23,00	38	1	1,33598	-0,19266
43,00	38,00	42,00	6,00	19,00	28	1	-0,56500	1,20158
80,00	20,00	12,00	8,00	16,00	30	1	1,63300	-0,36694
27,00	26,00	19,00	18,00	12,00	65	1	-1,51549	0,15590
85,00	52,00	19,00	22,00	12,00	61	1	1,93003	2,42155
52,00	29,00	27,00	12,00	25,00	37	2	-0,03035	0,41732
53,00	21,00	21,00	20,00	22,00	18	1	0,02905	-0,27980
61,00	17,00	26,00	6,00	25,00	20	1	0,50430	-0,62836
26,00	18,00	10,00	14,00	9,00	24	1	-1,57489	-0,54122
27,00	15,00	13,00	11,00	11,00	36	2	-1,51549	-0,80264
34,00	31,00	17,00	9,00	20,00	25	1	-1,09965	0,59160

39,00	27,00	20,00	9,00	10,00	52	1	-0,80262	0,24304
54,00	16,00	22,00	23,00	18,00	39	1	0,08846	-0,71550
80,00	61,00	56,00	17,00	12,00	48	1	1,63300	3,20581
22,00	10,00	10,00	26,00	3,00	33	1	-1,81252	-1,23834
66,00	15,00	11,00	28,00	22,00	52	1	0,80133	-0,80264
55,00	17,00	15,00	18,00	22,00	39	1	0,14787	-0,62836
35,00	10,00	11,00	12,00	11,00	28	1	-1,04024	-1,23834
60,00	38,00	48,00	12,00	17,00	31	1	0,44489	1,20158
57,00	31,00	29,00	3,00	11,00	28	1	0,26668	0,59160
66,00	28,00	25,00	17,00	11,00	37	1	0,80133	0,33018
45,00	12,00	16,00	3,00	23,00	52	1	-0,44619	-1,06406
54,00	13,00	21,00	25,00	5,00	63	1	0,08846	-0,97692
68,00	42,00	45,00	14,00	17,00	52	1	0,92014	1,55014
82,00	20,00	27,00	6,00	18,00	38	1	1,75181	-0,36694
43,00	30,00	52,00	1,00	18,00	28	2	-0,56500	0,50446
57,00	18,00	23,00	8,00	10,00	23	1	0,26668	-0,54122
62,00	34,00	17,00	15,00	6,00	32	1	0,56370	0,85302
26,00	20,00	26,00	13,00	13,00	56	1	-1,57489	-0,36694
57,00	40,00	23,00	12,00	16,00	39	1	0,26668	1,37586
61,00	10,00	14,00	12,00	13,00	50	1	0,50430	-1,23834
38,00	21,00	10,00	15,00	0,00	21	1	-0,86203	-0,27980
61,00	10,00	56,00	2,00	24,00	18	1	0,50430	-1,23834
42,00	30,00	45,00	7,00	21,00	37	2	-0,62441	0,50446
64,00	25,00	10,00	18,00	7,00	45	1	0,68252	0,06876
11,00	11,00	15,00	14,00	10,00	57	1	-2,46598	-1,15120
48,00	18,00	25,00	19,00	15,00	35	1	-0,26797	-0,54122
82,00	28,00	40,00	5,00	21,00	28	2	1,75181	0,33018
53,00	14,00	45,00	8,00	18,00	26	1	0,02905	-0,88978
53,00	29,00	15,00	16,00	14,00	21	1	0,02905	0,41732
18,00	16,00	35,00	12,00	13,00	30	1	-2,05014	-0,71550
43,00	40,00	58,00	9,00	20,00	35	2	-0,56500	1,37586
39,00	31,00	52,00	7,00	13,00	35	1	-0,80262	0,59160
60,00	27,00	53,00	9,00	15,00	21	1	0,44489	0,24304
27,00	17,00	22,00	13,00	17,00	54	1	-1,51549	-0,62836
79,00	51,00	54,00	8,00	26,00	30	1	1,57360	2,33441
56,00	33,00	43,00	13,00	6,00	31	1	0,20727	0,76588
30,00	22,00	25,00	26,00	18,00	53	1	-1,33727	-0,19266
63,00	44,00	42,00	19,00	21,00	22	1	0,62311	1,72442
27,00	19,00	21,00	13,00	13,00	63	2	-1,51549	-0,45408
58,00	21,00	17,00	11,00	16,00	47	1	0,32608	-0,27980
74,00	15,00	34,00	18,00	9,00	30	1	1,27657	-0,80264
33,00	46,00	47,00	7,00	18,00	35	1	-1,15906	1,89871
52,00	20,00	52,00	7,00	21,00	26	1	-0,03035	-0,36694
24,00	15,00	16,00	23,00	18,00	61	1	-1,69371	-0,80264
74,00	16,00	16,00	27,00	4,00	56	1	1,27657	-0,71550
67,00	39,00	33,00	15,00	24,00	40	1	0,86073	1,28872
44,00	16,00	40,00	1,00	21,00	30	1	-0,50560	-0,71550
50,00	20,00	26,00	12,00	12,00	18	1	-0,14916	-0,36694
45,00	46,00	57,00	9,00	20,00	32	1	-0,44619	1,89871
20,00	23,00	31,00	7,00	18,00	27	2	-1,93133	-0,10552

45,00	20,00	17,00	13,00	10,00	39	1	-0,44619	-0,36694
78,00	12,00	16,00	13,00	23,00	58	1	1,51419	-1,06406
79,00	12,00	44,00	6,00	25,00	42	1	1,57360	-1,06406
45,00	30,00	38,00	17,00	21,00	26	1	-0,44619	0,50446
56,00	41,00	30,00	9,00	15,00	24	1	0,20727	1,46300
79,00	25,00	29,00	12,00	11,00	42	1	1,57360	0,06876
55,00	21,00	16,00	9,00	15,00	30	1	0,14787	-0,27980
11,00	10,00	18,00	6,00	16,00	18	1	-2,46598	-1,23834
57,00	16,00	25,00	18,00	20,00	28	1	0,26668	-0,71550
62,00	19,00	33,00	26,00	16,00	46	1	0,56370	-0,45408
61,00	37,00	25,00	11,00	14,00	49	1	0,50430	1,11444
60,00	14,00	12,00	19,00	14,00	54	1	0,44489	-0,88978
41,00	35,00	50,00	5,00	14,00	31	1	-0,68381	0,94016
74,00	42,00	42,00	11,00	16,00	25	1	1,27657	1,55014
44,00	30,00	50,00	6,00	18,00	42	1	-0,50560	0,50446
58,00	10,00	10,00	9,00	19,00	26	1	0,32608	-1,23834
68,00	38,00	49,00	2,00	17,00	20	1	0,92014	1,20158
53,00	22,00	17,00	13,00	8,00	45	1	0,02905	-0,19266
38,00	33,00	47,00	19,00	5,00	38	1	-0,86203	0,76588
61,00	21,00	27,00	12,00	19,00	51	2	0,50430	-0,27980
38,00	15,00	12,00	21,00	14,00	26	1	-0,86203	-0,80264
44,00	30,00	18,00	24,00	13,00	28	2	-0,50560	0,50446
53,00	14,00	15,00	11,00	17,00	58	1	0,02905	-0,88978
18,00	21,00	21,00	17,00	17,00	35	1	-2,05014	-0,27980
41,00	41,00	51,00	5,00	25,00	32	1	-0,68381	1,46300
65,00	21,00	23,00	11,00	22,00	57	1	0,74192	-0,27980
68,00	42,00	44,00	3,00	26,00	56	2	0,92014	1,55014
53,00	10,00	17,00	24,00	13,00	31	1	0,02905	-1,23834
73,00	10,00	12,00	14,00	9,00	36	1	1,21717	-1,23834
78,00	10,00	10,00	1,00	24,00	43	1	1,51419	-1,23834
49,00	13,00	13,00	20,00	12,00	61	1	-0,20857	-0,97692
21,00	56,00	34,00	11,00	9,00	21	1	-1,87192	2,77011
48,00	28,00	19,00	18,00	19,00	53	1	-0,26797	0,33018
68,00	14,00	13,00	14,00	18,00	38	1	0,92014	-0,88978
68,00	26,00	36,00	10,00	22,00	18	1	0,92014	0,15590
64,00	14,00	11,00	12,00	10,00	47	1	0,68252	-0,88978
43,00	27,00	26,00	11,00	18,00	38	2	-0,56500	0,24304
76,00	18,00	24,00	0,00	24,00	50	2	1,39538	-0,54122
70,00	65,00	63,00	3,00	25,00	22	1	1,03895	3,55437
71,00	27,00	41,00	7,00	16,00	24	1	1,09835	0,24304
46,00	15,00	12,00	13,00	20,00	40	1	-0,38678	-0,80264
75,00	14,00	25,00	16,00	8,00	41	1	1,33598	-0,88978
53,00	14,00	24,00	11,00	13,00	34	1	0,02905	-0,88978
60,00	28,00	21,00	13,00	13,00	23	1	0,44489	0,33018
86,00	18,00	19,00	7,00	21,00	41	1	1,98944	-0,54122
57,00	28,00	35,00	13,00	19,00	23	2	0,26668	0,33018
63,00	27,00	39,00	10,00	12,00	34	1	0,62311	0,24304
30,00	48,00	47,00	15,00	11,00	20	1	-1,33727	2,07299
73,00	47,00	43,00	6,00	20,00	34	1	1,21717	1,98585
62,00	23,00	40,00	3,00	14,00	25	2	0,56370	-0,10552

67,00	10,00	14,00	18,00	6,00	42	1	0,86073	-1,23834
67,00	44,00	35,00	7,00	18,00	33	1	0,86073	1,72442
69,00	31,00	12,00	10,00	20,00	55	1	0,97954	0,59160
25,00	23,00	55,00	24,00	13,00	34	2	-1,63430	-0,10552
51,00	34,00	49,00	6,00	17,00	22	1	-0,08976	0,85302
44,00	13,00	14,00	15,00	11,00	49	1	-0,50560	-0,97692
55,00	19,00	33,00	0,00	24,00	20	1	0,14787	-0,45408
17,00	11,00	11,00	30,00	3,00	57	1	-2,10954	-1,15120
62,00	25,00	23,00	21,00	10,00	20	1	0,56370	0,06876
60,00	13,00	11,00	24,00	16,00	31	1	0,44489	-0,97692
50,00	27,00	14,00	12,00	16,00	41	1	-0,14916	0,24304
69,00	14,00	18,00	13,00	14,00	60	1	0,97954	-0,88978
48,00	23,00	22,00	13,00	11,00	35	1	-0,26797	-0,10552
62,00	31,00	18,00	9,00	18,00	20	1	0,56370	0,59160
47,00	22,00	32,00	19,00	7,00	30	1	-0,32738	-0,19266
51,00	18,00	25,00	17,00	14,00	38	1	-0,08976	-0,54122
36,00	15,00	29,00	13,00	12,00	50	2	-0,98084	-0,80264
58,00	10,00	20,00	6,00	17,00	25	1	0,32608	-1,23834
79,00	45,00	40,00	8,00	16,00	23	1	1,57360	1,81156
63,00	32,00	54,00	8,00	11,00	24	1	0,62311	0,67874
58,00	31,00	45,00	6,00	22,00	30	1	0,32608	0,59160
78,00	22,00	46,00	3,00	27,00	18	1	1,51419	-0,19266
33,00	32,00	45,00	13,00	16,00	22	1	-1,15906	0,67874
47,00	20,00	29,00	15,00	13,00	32	2	-0,32738	-0,36694
53,00	27,00	20,00	6,00	18,00	31	1	0,02905	0,24304
55,00	17,00	20,00	9,00	17,00	58	2	0,14787	-0,62836
27,00	17,00	14,00	20,00	14,00	35	1	-1,51549	-0,62836
23,00	22,00	34,00	6,00	17,00	18	1	-1,75311	-0,19266
61,00	28,00	22,00	24,00	8,00	62	1	0,50430	0,33018
78,00	11,00	17,00	4,00	21,00	23	1	1,51419	-1,15120
48,00	28,00	12,00	19,00	15,00	46	1	-0,26797	0,33018
60,00	26,00	59,00	4,00	26,00	27	1	0,44489	0,15590
75,00	10,00	12,00	20,00	13,00	21	1	1,33598	-1,23834
58,00	23,00	14,00	16,00	10,00	55	1	0,32608	-0,10552
41,00	38,00	70,00	2,00	18,00	20	1	-0,68381	1,20158
58,00	15,00	17,00	12,00	11,00	42	2	0,32608	-0,80264
65,00	25,00	17,00	9,00	3,00	49	1	0,74192	0,06876
57,00	16,00	11,00	30,00	4,00	49	1	0,26668	-0,71550
58,00	17,00	19,00	10,00	15,00	51	1	0,32608	-0,62836
70,00	23,00	23,00	8,00	15,00	62	2	1,03895	-0,10552
13,00	16,00	12,00	21,00	22,00	61	1	-2,34717	-0,71550
38,00	13,00	21,00	21,00	22,00	51	2	-0,86203	-0,97692
34,00	25,00	26,00	2,00	20,00	52	1	-1,09965	0,06876
53,00	20,00	13,00	23,00	10,00	27	1	0,02905	-0,36694
53,00	37,00	21,00	20,00	6,00	51	1	0,02905	1,11444
59,00	23,00	43,00	1,00	24,00	32	2	0,38549	-0,10552
26,00	21,00	23,00	10,00	15,00	45	1	-1,57489	-0,27980
67,00	36,00	41,00	13,00	15,00	39	1	0,86073	1,02730
49,00	11,00	32,00	7,00	20,00	35	1	-0,20857	-1,15120
61,00	23,00	15,00	5,00	16,00	26	1	0,50430	-0,10552

43,00	29,00	29,00	3,00	27,00	36	1	-0,56500	0,41732
51,00	51,00	46,00	27,00	9,00	49	1	-0,08976	2,33441
34,00	16,00	24,00	19,00	6,00	40	2	-1,09965	-0,71550
44,00	20,00	37,00	5,00	24,00	45	1	-0,50560	-0,36694
90,00	14,00	29,00	4,00	20,00	21	1	2,22706	-0,88978
63,00	11,00	11,00	6,00	24,00	44	1	0,62311	-1,15120
40,00	38,00	38,00	15,00	15,00	32	1	-0,74322	1,20158
53,00	44,00	42,00	6,00	24,00	20	2	0,02905	1,72442
64,00	12,00	43,00	27,00	18,00	31	2	0,68252	-1,06406
51,00	23,00	28,00	2,00	16,00	42	1	-0,08976	-0,10552
49,00	20,00	22,00	24,00	15,00	21	1	-0,20857	-0,36694
58,00	46,00	10,00	30,00	2,00	24	2	0,32608	1,89871
37,00	29,00	31,00	13,00	17,00	37	1	-0,92143	0,41732
55,00	16,00	41,00	7,00	17,00	50	1	0,14787	-0,71550
67,00	12,00	12,00	21,00	3,00	38	1	0,86073	-1,06406
46,00	24,00	12,00	30,00	4,00	33	1	-0,38678	-0,01838
47,00	30,00	31,00	17,00	19,00	43	1	-0,32738	0,50446
49,00	19,00	35,00	12,00	11,00	26	1	-0,20857	-0,45408
70,00	21,00	25,00	3,00	22,00	19	1	1,03895	-0,27980
14,00	10,00	14,00	20,00	18,00	40	1	-2,28776	-1,23834
52,00	18,00	30,00	4,00	18,00	35	1	-0,03035	-0,54122
30,00	16,00	17,00	20,00	4,00	35	1	-1,33727	-0,71550
57,00	12,00	13,00	13,00	17,00	55	1	0,26668	-1,06406
56,00	22,00	17,00	15,00	21,00	52	1	0,20727	-0,19266
52,00	32,00	50,00	3,00	15,00	18	1	-0,03035	0,67874
50,00	12,00	11,00	25,00	4,00	56	1	-0,14916	-1,06406
82,00	12,00	11,00	10,00	14,00	38	1	1,75181	-1,06406
44,00	22,00	10,00	30,00	0,00	21	1	-0,50560	-0,19266
68,00	19,00	23,00	13,00	16,00	51	1	0,92014	-0,45408
58,00	18,00	14,00	21,00	10,00	46	2	0,32608	-0,54122
54,00	23,00	13,00	25,00	4,00	22	1	0,08846	-0,10552
56,00	16,00	10,00	5,00	11,00	20	1	0,20727	-0,71550
14,00	14,00	10,00	30,00	8,00	46	1	-2,28776	-0,88978
39,00	45,00	57,00	3,00	20,00	52	2	-0,80262	1,81156
44,00	23,00	13,00	12,00	6,00	21	1	-0,50560	-0,10552
62,00	60,00	26,00	1,00	22,00	52	1	0,56370	3,11867
26,00	22,00	18,00	17,00	11,00	54	1	-1,57489	-0,19266
74,00	32,00	50,00	2,00	27,00	36	1	1,27657	0,67874
53,00	26,00	26,00	14,00	16,00	45	1	0,02905	0,15590
45,00	20,00	14,00	20,00	6,00	48	1	-0,44619	-0,36694
28,00	10,00	12,00	11,00	16,00	54	1	-1,45608	-1,23834
75,00	22,00	16,00	14,00	8,00	38	1	1,33598	-0,19266
66,00	27,00	10,00	15,00	15,00	52	1	0,80133	0,24304
43,00	47,00	14,00	14,00	16,00	57	1	-0,56500	1,98585
68,00	17,00	18,00	29,00	19,00	31	1	0,92014	-0,62836
90,00	11,00	12,00	1,00	26,00	43	1	2,22706	-1,15120
64,00	25,00	18,00	23,00	13,00	35	1	0,68252	0,06876
61,00	31,00	42,00	13,00	11,00	46	1	0,50430	0,59160
25,00	19,00	13,00	7,00	21,00	31	1	-1,63430	-0,45408
70,00	31,00	19,00	13,00	24,00	57	1	1,03895	0,59160

75,00	14,00	10,00	3,00	20,00	18	1	1,33598	-0,88978
62,00	29,00	59,00	2,00	26,00	52	1	0,56370	0,41732
75,00	69,00	76,00	2,00	20,00	19	1	1,33598	3,90293
51,00	41,00	21,00	14,00	10,00	53	1	-0,08976	1,46300
62,00	20,00	14,00	20,00	18,00	28	2	0,56370	-0,36694
61,00	21,00	34,00	7,00	22,00	28	2	0,50430	-0,27980
56,00	46,00	46,00	11,00	21,00	28	2	0,20727	1,89871
49,00	18,00	22,00	17,00	15,00	51	1	-0,20857	-0,54122
70,00	31,00	33,00	2,00	22,00	20	1	1,03895	0,59160
79,00	15,00	10,00	17,00	4,00	20	1	1,57360	-0,80264
33,00	19,00	24,00	22,00	12,00	46	1	-1,15906	-0,45408
73,00	80,00	73,00	15,00	4,00	21	2	1,21717	4,86147
69,00	25,00	50,00	5,00	18,00	21	1	0,97954	0,06876
55,00	32,00	40,00	9,00	18,00	23	1	0,14787	0,67874
58,00	15,00	17,00	12,00	19,00	22	2	0,32608	-0,80264
70,00	17,00	10,00	10,00	16,00	25	1	1,03895	-0,62836
76,00	17,00	40,00	7,00	25,00	23	1	1,39538	-0,62836
50,00	32,00	38,00	7,00	16,00	45	2	-0,14916	0,67874
71,00	31,00	42,00	11,00	20,00	38	1	1,09835	0,59160
58,00	19,00	46,00	9,00	22,00	62	1	0,32608	-0,45408
52,00	14,00	19,00	21,00	7,00	56	1	-0,03035	-0,88978
59,00	30,00	42,00	3,00	16,00	34	1	0,38549	0,50446
69,00	41,00	33,00	15,00	11,00	37	1	0,97954	1,46300
57,00	11,00	18,00	12,00	19,00	51	1	0,26668	-1,15120
36,00	17,00	43,00	3,00	15,00	36	1	-0,98084	-0,62836
53,00	23,00	27,00	11,00	17,00	20	1	0,02905	-0,10552
46,00	17,00	17,00	15,00	8,00	28	2	-0,38678	-0,62836
34,00	28,00	28,00	22,00	12,00	26	1	-1,09965	0,33018
74,00	20,00	39,00	1,00	21,00	40	1	1,27657	-0,36694
59,00	15,00	14,00	9,00	7,00	42	1	0,38549	-0,80264
50,00	23,00	25,00	29,00	16,00	51	1	-0,14916	-0,10552
38,00	27,00	37,00	4,00	24,00	23	1	-0,86203	0,24304
61,00	24,00	13,00	14,00	16,00	29	1	0,50430	-0,01838
60,00	19,00	41,00	9,00	24,00	22	1	0,44489	-0,45408
67,00	31,00	35,00	9,00	19,00	54	1	0,86073	0,59160
60,00	10,00	10,00	28,00	14,00	44	1	0,44489	-1,23834
67,00	21,00	12,00	14,00	15,00	54	1	0,86073	-0,27980
65,00	18,00	24,00	20,00	14,00	38	1	0,74192	-0,54122
59,00	49,00	28,00	3,00	26,00	54	1	0,38549	2,16013
68,00	54,00	60,00	2,00	23,00	53	1	0,92014	2,59583
68,00	25,00	22,00	9,00	14,00	20	1	0,92014	0,06876
66,00	28,00	29,00	5,00	26,00	33	1	0,80133	0,33018
47,00	28,00	33,00	15,00	22,00	31	2	-0,32738	0,33018
37,00	10,00	25,00	6,00	18,00	18	1	-0,92143	-1,23834
80,00	35,00	22,00	15,00	19,00	49	1	1,63300	0,94016
62,00	21,00	44,00	6,00	18,00	27	1	0,56370	-0,27980
40,00	19,00	28,00	8,00	17,00	51	1	-0,74322	-0,45408
67,00	63,00	40,00	10,00	23,00	40	1	0,86073	3,38009
45,00	15,00	13,00	18,00	8,00	27	1	-0,44619	-0,80264
49,00	12,00	17,00	15,00	8,00	33	1	-0,20857	-1,06406

68,00	33,00	15,00	13,00	17,00	24	1	0,92014	0,76588
53,00	13,00	12,00	29,00	4,00	48	1	0,02905	-0,97692
69,00	14,00	12,00	20,00	6,00	50	1	0,97954	-0,88978
53,00	18,00	17,00	24,00	3,00	20	1	0,02905	-0,54122
47,00	34,00	37,00	10,00	14,00	39	1	-0,32738	0,85302
42,00	14,00	45,00	4,00	24,00	19	1	-0,62441	-0,88978
33,00	22,00	21,00	13,00	7,00	51	1	-1,15906	-0,19266
49,00	38,00	37,00	8,00	21,00	43	1	-0,20857	1,20158
67,00	22,00	24,00	20,00	15,00	43	2	0,86073	-0,19266
21,00	20,00	18,00	11,00	15,00	18	1	-1,87192	-0,36694
69,00	28,00	29,00	17,00	18,00	46	2	0,97954	0,33018
38,00	24,00	25,00	10,00	10,00	35	1	-0,86203	-0,01838
17,00	15,00	13,00	28,00	5,00	49	2	-2,10954	-0,80264
74,00	14,00	54,00	3,00	21,00	18	1	1,27657	-0,88978
51,00	43,00	49,00	18,00	16,00	62	2	-0,08976	1,63728
56,00	17,00	19,00	9,00	15,00	50	1	0,20727	-0,62836
80,00	18,00	65,00	4,00	25,00	28	1	1,63300	-0,54122
70,00	28,00	52,00	17,00	14,00	23	2	1,03895	0,33018
48,00	16,00	18,00	12,00	11,00	51	1	-0,26797	-0,71550
50,00	30,00	28,00	8,00	26,00	33	1	-0,14916	0,50446
68,00	11,00	17,00	9,00	11,00	28	1	0,92014	-1,15120
29,00	31,00	54,00	1,00	21,00	38	1	-1,39668	0,59160
16,00	12,00	16,00	22,00	10,00	46	2	-2,16895	-1,06406
53,00	33,00	41,00	4,00	18,00	30	1	0,02905	0,76588
69,00	26,00	19,00	4,00	18,00	23	1	0,97954	0,15590
38,00	20,00	21,00	18,00	11,00	44	1	-0,86203	-0,36694
68,00	26,00	19,00	4,00	14,00	43	1	0,92014	0,15590
63,00	19,00	16,00	20,00	10,00	26	1	0,62311	-0,45408
57,00	26,00	30,00	2,00	21,00	22	1	0,26668	0,15590
61,00	21,00	28,00	13,00	23,00	36	1	0,50430	-0,27980
61,00	10,00	13,00	7,00	23,00	62	1	0,50430	-1,23834
62,00	24,00	25,00	19,00	24,00	52	1	0,56370	-0,01838
72,00	39,00	52,00	13,00	12,00	32	1	1,15776	1,28872
18,00	14,00	18,00	21,00	13,00	49	1	-2,05014	-0,88978
49,00	25,00	28,00	14,00	14,00	56	1	-0,20857	0,06876
64,00	19,00	40,00	2,00	16,00	36	1	0,68252	-0,45408
68,00	16,00	11,00	25,00	11,00	50	1	0,92014	-0,71550
33,00	19,00	21,00	18,00	16,00	22	1	-1,15906	-0,45408
33,00	31,00	45,00	10,00	14,00	19	1	-1,15906	0,59160
65,00	15,00	36,00	1,00	14,00	24	1	0,74192	-0,80264
50,00	25,00	26,00	8,00	19,00	40	1	-0,14916	0,06876
45,00	29,00	27,00	17,00	13,00	22	1	-0,44619	0,41732
41,00	19,00	18,00	9,00	18,00	44	1	-0,68381	-0,45408
49,00	10,00	41,00	10,00	17,00	21	1	-0,20857	-1,23834
62,00	10,00	32,00	14,00	18,00	51	1	0,56370	-1,23834
38,00	17,00	14,00	10,00	11,00	26	1	-0,86203	-0,62836
37,00	28,00	20,00	30,00	2,00	59	1	-0,92143	0,33018
64,00	18,00	23,00	22,00	13,00	50	1	0,68252	-0,54122
49,00	32,00	25,00	11,00	18,00	48	2	-0,20857	0,67874
26,00	24,00	34,00	11,00	15,00	56	2	-1,57489	-0,01838

55,00	35,00	11,00	23,00	8,00	49	1	0,14787	0,94016
23,00	13,00	11,00	18,00	12,00	39	1	-1,30497	-0,91773
17,00	13,00	12,00	19,00	6,00	56	2	-1,63380	-0,91773
45,00	13,00	15,00	21,00	4,00	25	2	-0,09926	-0,91773
74,00	22,00	35,00	14,00	12,00	31	1	1,49008	-0,20943
52,00	12,00	23,00	11,00	13,00	27	2	0,28438	-0,99643
12,00	12,00	24,00	12,00	13,00	28	1	-1,90782	-0,99643
53,00	45,00	35,00	11,00	19,00	34	2	0,33918	1,60067
60,00	17,00	11,00	23,00	12,00	20	2	0,72282	-0,60293
10,00	10,00	31,00	13,00	15,00	44	1	-2,01743	-1,15383
45,00	31,00	25,00	13,00	7,00	33	2	-0,09926	0,49887
63,00	17,00	15,00	23,00	5,00	59	2	0,88723	-0,60293
87,00	11,00	13,00	22,00	5,00	24	1	2,20255	-1,07513
72,00	36,00	51,00	14,00	14,00	29	1	1,38047	0,89237
19,00	13,00	20,00	10,00	21,00	38	2	-1,52419	-0,91773
42,00	23,00	11,00	14,00	5,00	23	2	-0,26367	-0,13073
88,00	10,00	41,00	15,00	26,00	44	1	2,25735	-1,15383
47,00	42,00	60,00	13,00	10,00	57	2	0,01035	1,36457
68,00	28,00	34,00	2,00	19,00	21	1	1,16126	0,26277
20,00	15,00	19,00	14,00	8,00	49	1	-1,46938	-0,76033
38,00	13,00	19,00	23,00	13,00	42	1	-0,48289	-0,91773
22,00	18,00	18,00	22,00	10,00	32	1	-1,35977	-0,52423
60,00	12,00	10,00	21,00	5,00	34	1	0,72282	-0,99643
57,00	54,00	46,00	17,00	13,00	19	1	0,55840	2,30897
35,00	20,00	24,00	14,00	13,00	38	1	-0,64731	-0,36683
61,00	16,00	32,00	1,00	25,00	52	2	0,77762	-0,68163
59,00	26,00	31,00	16,00	19,00	21	2	0,66801	0,10537
80,00	41,00	32,00	12,00	14,00	51	2	1,81891	1,28587
40,00	22,00	18,00	21,00	14,00	54	2	-0,37328	-0,20943
54,00	28,00	22,00	9,00	17,00	21	1	0,39399	0,26277
64,00	20,00	21,00	8,00	13,00	25	1	0,94204	-0,36683
20,00	12,00	15,00	22,00	15,00	23	2	-1,46938	-0,99643
58,00	17,00	20,00	19,00	14,00	28	2	0,61321	-0,60293
45,00	61,00	10,00	19,00	11,00	42	1	-0,09926	2,85987
36,00	27,00	20,00	13,00	17,00	21	1	-0,59250	0,18407
46,00	42,00	12,00	20,00	6,00	40	1	-0,04445	1,36457
77,00	27,00	62,00	1,00	19,00	35	1	1,65450	0,18407
29,00	25,00	37,00	9,00	20,00	21	2	-0,97614	0,02667
54,00	12,00	22,00	21,00	23,00	52	1	0,39399	-0,99643
22,00	10,00	45,00	7,00	24,00	55	1	-1,35977	-1,15383
68,00	26,00	36,00	10,00	17,00	24	2	1,16126	0,10537
62,00	30,00	23,00	7,00	9,00	33	2	0,83243	0,42017
22,00	20,00	28,00	10,00	10,00	39	2	-1,35977	-0,36683
64,00	31,00	40,00	11,00	16,00	21	2	0,94204	0,49887
22,00	17,00	34,00	25,00	19,00	39	1	-1,35977	-0,60293
23,00	13,00	47,00	18,00	17,00	59	1	-1,30497	-0,91773
29,00	19,00	23,00	29,00	5,00	26	2	-0,97614	-0,44553
34,00	36,00	47,00	17,00	16,00	33	1	-0,70211	0,89237
60,00	31,00	22,00	7,00	20,00	31	1	0,72282	0,49887
70,00	12,00	23,00	14,00	12,00	59	2	1,27087	-0,99643

33,00	30,00	28,00	13,00	11,00	51	1	-0,75692	0,42017
35,00	35,00	37,00	11,00	21,00	25	1	-0,64731	0,81367
14,00	16,00	19,00	14,00	16,00	52	2	-1,79821	-0,68163
56,00	19,00	24,00	7,00	25,00	49	1	0,50360	-0,44553
61,00	16,00	10,00	27,00	12,00	49	2	0,77762	-0,68163
50,00	10,00	24,00	7,00	15,00	23	1	0,17477	-1,15383
19,00	14,00	49,00	9,00	20,00	48	2	-1,52419	-0,83903
53,00	14,00	15,00	16,00	11,00	26	1	0,33918	-0,83903
59,00	12,00	13,00	18,00	9,00	55	1	0,66801	-0,99643
59,00	33,00	34,00	5,00	22,00	53	2	0,66801	0,65627
71,00	20,00	24,00	13,00	6,00	32	1	1,32567	-0,36683
55,00	46,00	33,00	6,00	19,00	43	1	0,44879	1,67937
52,00	16,00	14,00	13,00	10,00	38	1	0,28438	-0,68163
20,00	13,00	19,00	21,00	19,00	47	2	-1,46938	-0,91773
36,00	38,00	30,00	12,00	22,00	30	1	-0,59250	1,04977
77,00	32,00	22,00	3,00	19,00	26	1	1,65450	0,57757
31,00	33,00	27,00	14,00	16,00	40	1	-0,86653	0,65627
32,00	41,00	38,00	19,00	7,00	55	1	-0,81172	1,28587
43,00	13,00	13,00	12,00	16,00	29	1	-0,20887	-0,91773
84,00	13,00	11,00	13,00	16,00	32	2	2,03813	-0,91773
76,00	58,00	66,00	1,00	27,00	50	1	1,59969	2,62377
59,00	55,00	34,00	21,00	8,00	51	2	0,66801	2,38767
48,00	31,00	19,00	23,00	9,00	56	1	0,06516	0,49887
25,00	18,00	27,00	4,00	22,00	35	2	-1,19536	-0,52423
56,00	35,00	30,00	20,00	9,00	22	2	0,50360	0,81367
49,00	50,00	14,00	5,00	18,00	47	1	0,11996	1,99417
33,00	15,00	17,00	23,00	16,00	42	1	-0,75692	-0,76033
38,00	54,00	54,00	10,00	18,00	61	1	-0,48289	2,30897
30,00	26,00	27,00	17,00	6,00	34	1	-0,92133	0,10537
53,00	12,00	22,00	16,00	13,00	61	1	0,33918	-0,99643
10,00	14,00	10,00	17,00	12,00	62	1	-2,01743	-0,83903
18,00	15,00	22,00	17,00	11,00	40	1	-1,57899	-0,76033
28,00	14,00	14,00	25,00	10,00	66	2	-1,03094	-0,83903
70,00	13,00	33,00	13,00	10,00	40	2	1,27087	-0,91773
38,00	36,00	59,00	5,00	23,00	58	2	-0,48289	0,89237
39,00	31,00	29,00	15,00	11,00	21	2	-0,42809	0,49887
42,00	40,00	46,00	15,00	17,00	52	1	-0,26367	1,20717
33,00	16,00	29,00	13,00	19,00	57	2	-0,75692	-0,68163
50,00	10,00	18,00	25,00	12,00	60	2	0,17477	-1,15383
66,00	36,00	13,00	8,00	13,00	25	1	1,05165	0,89237
46,00	58,00	38,00	15,00	11,00	45	1	-0,04445	2,62377
67,00	26,00	34,00	6,00	19,00	58	1	1,10645	0,10537
70,00	22,00	10,00	9,00	17,00	43	1	1,27087	-0,20943
48,00	21,00	16,00	20,00	4,00	30	2	0,06516	-0,28813
73,00	69,00	55,00	15,00	15,00	54	2	1,43528	3,48947
33,00	28,00	12,00	15,00	23,00	47	1	-0,75692	0,26277
52,00	14,00	17,00	21,00	13,00	48	2	0,28438	-0,83903
67,00	21,00	29,00	7,00	24,00	33	2	1,10645	-0,28813
11,00	20,00	10,00	15,00	23,00	43	1	-1,96262	-0,36683
34,00	37,00	20,00	17,00	7,00	40	2	-0,70211	0,97107

61,00	63,00	55,00	12,00	23,00	49	2	0,77762	3,01727
41,00	17,00	17,00	17,00	14,00	29	2	-0,31848	-0,60293
48,00	21,00	12,00	18,00	9,00	64	1	0,06516	-0,28813
29,00	13,00	29,00	20,00	19,00	38	2	-0,97614	-0,91773
29,00	19,00	18,00	20,00	8,00	43	2	-0,97614	-0,44553
45,00	16,00	10,00	19,00	8,00	49	1	-0,09926	-0,68163
59,00	32,00	38,00	13,00	13,00	29	1	0,66801	0,57757
18,00	34,00	34,00	25,00	12,00	60	2	-1,57899	0,73497
45,00	34,00	24,00	12,00	6,00	33	2	-0,09926	0,73497
66,00	20,00	24,00	11,00	10,00	26	2	1,05165	-0,36683
70,00	18,00	13,00	15,00	10,00	40	1	1,27087	-0,52423
50,00	19,00	30,00	11,00	18,00	38	2	0,17477	-0,44553
35,00	24,00	11,00	26,00	7,00	37	2	-0,64731	-0,05203
40,00	14,00	14,00	13,00	8,00	29	1	-0,37328	-0,83903
37,00	29,00	16,00	23,00	5,00	46	1	-0,53770	0,34147
90,00	13,00	20,00	16,00	17,00	51	1	2,36696	-0,91773
67,00	16,00	42,00	2,00	20,00	24	1	1,10645	-0,68163
61,00	20,00	17,00	14,00	11,00	20	2	0,77762	-0,36683
27,00	27,00	14,00	10,00	5,00	33	1	-1,08575	0,18407
42,00	22,00	18,00	12,00	17,00	21	2	-0,26367	-0,20943
19,00	23,00	13,00	29,00	10,00	69	2	-1,52419	-0,13073
24,00	23,00	20,00	6,00	19,00	25	2	-1,25016	-0,13073
51,00	24,00	36,00	9,00	20,00	26	2	0,22957	-0,05203
39,00	25,00	12,00	23,00	6,00	24	1	-0,42809	0,02667
52,00	24,00	33,00	8,00	22,00	29	1	0,28438	-0,05203
11,00	10,00	19,00	13,00	9,00	41	1	-1,96262	-1,15383
55,00	18,00	19,00	14,00	7,00	20	1	0,44879	-0,52423
20,00	12,00	10,00	22,00	6,00	30	1	-1,46938	-0,99643
61,00	15,00	12,00	16,00	15,00	24	2	0,77762	-0,76033
55,00	25,00	27,00	18,00	13,00	30	1	0,44879	0,02667
19,00	16,00	14,00	15,00	14,00	24	1	-1,52419	-0,68163
68,00	36,00	33,00	18,00	12,00	25	1	1,16126	0,89237
62,00	26,00	30,00	11,00	14,00	35	1	0,83243	0,10537
57,00	53,00	47,00	10,00	15,00	50	2	0,55840	2,23027
63,00	12,00	26,00	6,00	16,00	47	1	0,88723	-0,99643
34,00	39,00	23,00	14,00	17,00	35	1	-0,70211	1,12847
46,00	23,00	20,00	7,00	19,00	54	2	-0,04445	-0,13073
66,00	26,00	16,00	25,00	6,00	57	2	1,05165	0,10537
48,00	43,00	20,00	14,00	15,00	32	2	0,06516	1,44327
51,00	54,00	18,00	9,00	18,00	28	1	0,22957	2,30897
54,00	26,00	21,00	12,00	15,00	62	2	0,39399	0,10537
67,00	31,00	20,00	16,00	8,00	41	2	1,10645	0,49887
45,00	19,00	42,00	4,00	21,00	32	1	-0,09926	-0,44553
26,00	20,00	15,00	8,00	8,00	46	1	-1,14055	-0,36683
42,00	26,00	42,00	5,00	21,00	45	1	-0,26367	0,10537
54,00	44,00	47,00	6,00	21,00	37	1	0,39399	1,52197
72,00	30,00	26,00	4,00	21,00	48	1	1,38047	0,42017
18,00	14,00	15,00	15,00	11,00	42	1	-1,57899	-0,83903
70,00	16,00	17,00	9,00	14,00	35	1	1,27087	-0,68163
55,00	42,00	40,00	13,00	21,00	46	1	0,44879	1,36457

45,00	18,00	24,00	19,00	12,00	50	2	-0,09926	-0,52423
59,00	27,00	13,00	20,00	1,00	49	1	0,66801	0,18407
43,00	23,00	29,00	14,00	23,00	26	1	-0,20887	-0,13073
50,00	10,00	11,00	27,00	6,00	58	1	0,17477	-1,15383
63,00	23,00	14,00	12,00	16,00	29	1	0,88723	-0,13073
61,00	22,00	26,00	16,00	13,00	50	1	0,77762	-0,20943
36,00	24,00	28,00	17,00	14,00	32	2	-0,59250	-0,05203
41,00	13,00	14,00	23,00	16,00	29	1	-0,31848	-0,91773
36,00	20,00	27,00	13,00	10,00	31	1	-0,59250	-0,36683
54,00	20,00	35,00	7,00	17,00	20	2	0,39399	-0,36683
57,00	15,00	25,00	5,00	21,00	24	2	0,55840	-0,76033
46,00	28,00	42,00	8,00	17,00	36	2	-0,04445	0,26277
43,00	10,00	55,00	4,00	27,00	32	1	-0,20887	-1,15383
51,00	15,00	12,00	16,00	7,00	44	2	0,22957	-0,76033
37,00	23,00	38,00	14,00	10,00	26	1	-0,53770	-0,13073
50,00	27,00	48,00	15,00	6,00	24	1	0,17477	0,18407
36,00	24,00	10,00	29,00	5,00	29	2	-0,59250	-0,05203
70,00	23,00	20,00	16,00	22,00	55	1	1,27087	-0,13073
36,00	20,00	17,00	20,00	6,00	27	1	-0,59250	-0,36683
70,00	59,00	50,00	15,00	14,00	21	2	1,27087	2,70247
48,00	39,00	62,00	15,00	7,00	53	1	0,06516	1,12847
53,00	30,00	36,00	14,00	16,00	31	2	0,33918	0,42017
43,00	10,00	39,00	10,00	22,00	35	1	-0,20887	-1,15383
46,00	33,00	28,00	18,00	14,00	46	1	-0,04445	0,65627
70,00	32,00	15,00	11,00	19,00	38	1	1,27087	0,57757
10,00	10,00	18,00	19,00	5,00	56	1	-2,01743	-1,15383
51,00	10,00	11,00	20,00	7,00	30	2	0,22957	-1,15383
23,00	10,00	14,00	15,00	12,00	50	1	-1,30497	-1,15383
66,00	18,00	32,00	8,00	16,00	18	1	1,05165	-0,52423
10,00	10,00	18,00	10,00	9,00	48	1	-2,01743	-1,15383
48,00	47,00	33,00	6,00	18,00	53	1	0,06516	1,75807

Zself_b	ZSW_B	ZPHQ	ZAGE	ZSEX	Sample	treat
-0,71705	0,80032	-1,94656	0,50814	2,27384	0	0
1,17630	-1,37674	0,50859	1,11225	-0,43898	0	1
-0,29631	1,61671	-0,80083	1,18776	-0,43898	0	1
-0,50668	-0,42428	-0,30980	-0,17148	-0,43898	0	0
0,82568	-0,15215	-0,63715	-0,62457	2,27384	0	1
-0,15606	1,48065	0,18123	-1,07765	-0,43898	0	1
-0,57680	-0,96854	-1,12818	-0,02046	-0,43898	0	0
-0,22618	0,11999	0,18123	0,50814	-0,43898	0	0
-0,57680	0,25605	0,34491	-0,32251	-0,43898	0	1
-0,99755	1,75278	-1,61921	-0,24700	-0,43898	0	1
-0,85730	0,93638	-1,78289	0,35711	2,27384	0	1
-0,92743	-1,10461	0,83594	-0,77559	-0,43898	0	0
1,80742	-1,51281	0,01756	-0,39803	-0,43898	0	1
1,24643	-1,51281	0,99962	-0,62457	-0,43898	0	1
-0,85730	2,02491	-0,96450	-1,22868	-0,43898	0	1
-0,85730	-0,83248	-0,80083	-1,30419	-0,43898	0	1
1,10618	-0,56034	-0,80083	-0,47354	-0,43898	0	1
0,33481	-1,10461	1,32697	-1,45522	-0,43898	0	1
0,75556	0,80032	-0,30980	-1,15316	2,27384	0	0
-1,20792	2,02491	-2,11024	-1,22868	-0,43898	0	1
-0,57680	0,11999	-0,96450	-0,85111	-0,43898	0	0
-0,71705	1,75278	-1,29186	1,56533	-0,43898	0	1
0,12444	0,80032	0,67226	2,54701	2,27384	0	1
1,52692	-0,28821	0,67226	1,18776	2,27384	0	1
0,89581	-0,56034	-0,47347	0,58365	-0,43898	0	1
-0,85730	-0,28821	-0,30980	-1,15316	-0,43898	0	1
-0,50668	0,25605	-1,45553	-0,77559	2,27384	0	0
-1,20792	1,34458	-2,11024	-0,32251	-0,43898	0	1
-0,36643	-0,15215	0,99962	1,94290	-0,43898	0	0
-0,36643	-0,15215	-0,47347	-1,15316	-0,43898	0	1
0,68543	-0,56034	0,50859	1,18776	-0,43898	0	1
-0,01581	0,39212	0,01756	0,81019	2,27384	0	1
0,26469	-0,42428	0,01756	0,35711	-0,43898	0	1
0,26469	-1,51281	1,32697	1,03673	-0,43898	0	1
1,59705	-0,15215	0,83594	-1,00214	-0,43898	0	1
0,19456	0,52818	0,50859	1,18776	-0,43898	0	1
-0,78718	1,20851	-1,29186	-1,37970	-0,43898	0	1
0,33481	-0,56034	1,49065	0,88571	2,27384	0	1
-0,92743	0,25605	-1,29186	1,79187	-0,43898	0	1
-0,64693	-0,15215	-0,47347	0,81019	2,27384	0	0
0,40494	-0,69641	-0,63715	0,96122	2,27384	0	0
-1,13780	1,61671	-1,61921	-0,70008	-0,43898	0	1
-1,20792	1,88884	-2,11024	0,43262	-0,43898	0	1
-0,64693	1,07245	0,34491	1,79187	-0,43898	0	1
-1,06767	1,20851	-1,12818	2,69804	-0,43898	0	1
-0,57680	-0,01608	-1,61921	-0,09597	-0,43898	0	1
0,19456	1,07245	-1,61921	0,88571	-0,43898	0	0
-0,43656	-0,56034	1,16330	1,79187	-0,43898	0	1
2,01779	0,80032	0,50859	-0,32251	-0,43898	0	1

0,05431	0,66425	-0,47347	-0,54905	-0,43898	0	1
0,33481	0,93638	-0,14612	1,64084	-0,43898	0	0
-0,71705	-1,24067	1,81800	-1,45522	2,27384	0	1
2,71904	-0,56034	0,83594	1,03673	2,27384	0	1
1,87755	1,75278	-2,11024	0,58365	-0,43898	0	0
-0,36643	0,93638	-0,96450	1,03673	-0,43898	0	1
-0,22618	1,34458	0,34491	0,88571	-0,43898	0	1
-0,57680	0,11999	-0,80083	0,73468	-0,43898	0	1
-0,36643	0,52818	-0,47347	1,26328	-0,43898	0	1
1,24643	-0,83248	0,99962	-1,45522	2,27384	0	0
1,38668	-1,24067	0,34491	-0,85111	-0,43898	0	2
-0,71705	0,11999	0,34491	-0,85111	2,27384	0	1
-0,57680	0,93638	-0,96450	-0,32251	-0,43898	0	1
-0,92743	1,48065	0,01756	0,65917	-0,43898	0	1
1,73730	-1,24067	0,67226	-1,22868	-0,43898	0	0
-0,64693	0,11999	-0,80083	1,18776	-0,43898	0	1
-1,13780	1,61671	-2,11024	0,88571	2,27384	0	0
0,26469	-0,56034	-0,30980	0,58365	2,27384	0	1
-0,22618	-1,10461	-0,30980	0,05506	-0,43898	0	0
-0,85730	1,07245	-0,47347	0,28160	-0,43898	0	1
-1,13780	0,11999	0,50859	1,18776	2,27384	0	1
-1,20792	0,93638	-2,11024	1,48982	-0,43898	0	0
-1,20792	1,61671	-1,29186	-0,62457	-0,43898	0	1
-0,43656	0,25605	-0,14612	-0,39803	-0,43898	0	1
1,38668	-0,28821	0,50859	-0,92662	2,27384	0	1
-1,20792	1,34458	-0,96450	0,05506	-0,43898	0	1
0,19456	-1,51281	1,16330	-1,37970	-0,43898	0	1
0,68543	-1,37674	1,65433	-0,70008	-0,43898	0	1
-1,13780	1,34458	-1,78289	-1,07765	-0,43898	0	1
0,33481	1,07245	-0,63715	-0,85111	-0,43898	0	1
-1,06767	-0,56034	-0,14612	-1,30419	-0,43898	0	1
-0,15606	-0,56034	0,01756	-1,22868	-0,43898	0	1
-0,57680	-0,83248	1,16330	1,03673	-0,43898	0	1
-0,57680	-0,01608	-1,45553	-0,17148	2,27384	0	1
-0,08593	-0,15215	0,18123	-1,37970	-0,43898	0	0
-0,99755	2,16098	-1,94656	-0,54905	-0,43898	0	0
-1,20792	1,61671	-1,94656	-0,39803	-0,43898	0	0
-0,43656	-0,69641	1,32697	0,96122	-0,43898	0	1
-1,20792	0,52818	-0,30980	1,41430	2,27384	0	1
-1,13780	0,25605	-0,80083	0,35711	-0,43898	0	1
-0,29631	-1,10461	-0,63715	-1,45522	-0,43898	0	1
-0,78718	0,25605	-0,80083	-0,54905	-0,43898	0	0
-0,43656	-0,28821	0,18123	-0,39803	-0,43898	0	1
-0,78718	-0,28821	-0,80083	1,11225	-0,43898	0	1
1,03605	0,11999	0,67226	0,20608	-0,43898	0	1
2,64891	-0,15215	1,16330	-0,39803	-0,43898	0	0
1,10618	-0,42428	1,16330	-0,39803	-0,43898	0	1
-0,08593	-0,15215	0,34491	0,20608	2,27384	0	1
2,22817	-1,51281	0,34491	-1,45522	-0,43898	0	1
0,89581	-1,37674	0,50859	0,20608	-0,43898	0	1

-0,99755	0,66425	-0,80083	2,09393	-0,43898	0	1
-0,36643	-0,15215	0,50859	-1,22868	-0,43898	0	1
-0,71705	0,66425	1,16330	1,48982	-0,43898	0	0
0,26469	-0,69641	0,34491	-0,92662	-0,43898	0	1
-1,20792	1,61671	-0,63715	1,18776	-0,43898	0	1
-0,85730	0,25605	-0,80083	-1,45522	-0,43898	0	1
-0,64693	-0,01608	0,50859	0,50814	-0,43898	0	1
-0,64693	0,25605	-0,30980	-0,70008	-0,43898	0	1
-0,22618	-1,10461	0,18123	-1,37970	-0,43898	0	1
-0,15606	1,34458	-0,14612	-0,70008	-0,43898	0	1
0,33481	-0,28821	1,16330	-1,45522	-0,43898	0	0
-0,50668	-0,15215	0,01756	-0,24700	-0,43898	0	1
-0,78718	0,93638	-1,61921	2,24495	-0,43898	0	0
-0,50668	0,80032	-0,96450	-1,00214	-0,43898	0	0
-0,57680	-1,37674	0,34491	-0,62457	-0,43898	0	1
-0,43656	1,20851	0,67226	-0,09597	-0,43898	0	1
0,26469	0,52818	-0,80083	1,64084	2,27384	0	1
0,05431	0,80032	-0,96450	1,33879	-0,43898	0	0
-1,20792	1,61671	-1,78289	-0,32251	-0,43898	0	1
-0,50668	0,80032	1,16330	0,13057	-0,43898	0	1
-0,29631	0,39212	-0,47347	2,24495	-0,43898	0	0
-0,92743	0,66425	0,67226	-1,22868	-0,43898	0	1
0,54518	-0,56034	1,16330	-1,45522	-0,43898	0	1
-0,71705	0,25605	1,16330	0,88571	-0,43898	0	1
0,05431	-0,83248	0,18123	-1,37970	2,27384	0	1
0,47506	-0,83248	1,32697	-0,32251	-0,43898	0	1
-0,99755	-1,24067	0,67226	-1,37970	-0,43898	0	1
-1,06767	0,80032	0,01756	0,28160	-0,43898	0	0
-0,71705	1,20851	-0,96450	0,43262	2,27384	0	0
0,82568	-1,64887	1,32697	0,13057	-0,43898	0	1
-1,20792	-0,42428	0,18123	-0,09597	-0,43898	0	1
-0,57680	1,07245	-1,12818	0,05506	-0,43898	0	1
-1,20792	0,93638	-1,45553	0,43262	-0,43898	0	0
-0,08593	2,29704	-2,11024	0,96122	-0,43898	0	1
-1,06767	-0,01608	-0,47347	-0,09597	-0,43898	0	1
1,73730	-1,37674	-0,14612	0,96122	-0,43898	0	0
-1,13780	2,16098	-2,27392	-0,17148	-0,43898	0	0
0,19456	0,93638	-1,78289	0,43262	2,27384	0	0
-0,85730	-1,10461	-0,30980	-1,22868	-0,43898	0	1
-0,36643	1,20851	-0,47347	0,58365	-0,43898	0	0
0,19456	0,25605	-0,63715	1,48982	2,27384	0	0
-0,85730	1,75278	-2,11024	1,11225	-0,43898	0	0
1,52692	-0,01608	-0,14612	-0,39803	-0,43898	0	0
0,89581	-1,37674	1,16330	-1,37970	-0,43898	0	1
2,92941	-0,28821	0,18123	-1,00214	2,27384	0	0
-1,20792	-0,83248	0,50859	1,26328	-0,43898	0	1
-1,13780	0,66425	1,32697	0,73468	2,27384	0	1
1,59705	-0,56034	0,99962	-0,92662	-0,43898	0	0
-0,99755	0,80032	-1,61921	-1,15316	-0,43898	0	0
0,54518	1,48065	0,01756	1,41430	-0,43898	0	1

-0,57680	1,61671	-1,45553	1,79187	-0,43898	0	1
-0,57680	1,20851	-1,78289	1,41430	-0,43898	0	1
-1,06767	0,80032	-1,61921	1,71636	2,27384	0	0
1,45680	-0,42428	-1,12818	-1,00214	-0,43898	0	0
-0,22618	2,02491	0,34491	-0,62457	-0,43898	0	1
-1,06767	-0,56034	-0,30980	-1,22868	-0,43898	0	1
-1,13780	0,93638	-1,94656	-0,32251	-0,43898	0	0
-1,06767	-0,42428	0,50859	1,11225	-0,43898	0	1
-1,13780	1,75278	-0,80083	1,86739	2,27384	0	0
-1,13780	0,80032	-0,80083	1,03673	-0,43898	0	1
0,61531	-0,01608	-0,63715	-0,85111	-0,43898	0	1
1,80742	-0,42428	-0,14612	-1,30419	-0,43898	0	1
-0,57680	0,93638	-1,78289	-1,00214	2,27384	0	1
-0,50668	1,48065	0,01756	-0,17148	2,27384	0	1
-1,20792	0,66425	-0,63715	-1,07765	-0,43898	0	1
2,01779	-1,24067	0,99962	1,33879	-0,43898	0	1
-0,64693	-0,01608	-0,47347	-0,09597	-0,43898	0	0
-0,15606	-0,01608	1,49065	1,33879	-0,43898	0	1
-0,01581	-0,42428	-0,63715	1,11225	-0,43898	0	1
1,73730	-1,78494	1,16330	-1,45522	-0,43898	0	1
-0,36643	-0,83248	-0,14612	-1,07765	-0,43898	0	0
1,24643	0,25605	0,01756	-0,77559	-0,43898	0	1
0,75556	-0,83248	1,16330	-0,39803	-0,43898	0	1
-0,92743	-0,69641	-0,63715	-0,02046	-0,43898	0	1
1,87755	0,39212	0,83594	1,33879	2,27384	0	1
-0,92743	1,75278	0,34491	1,79187	-0,43898	0	2
-0,29631	-0,42428	-0,47347	-1,37970	-0,43898	0	0
0,75556	-0,01608	0,67226	-0,32251	-0,43898	0	0
0,54518	-0,83248	1,16330	-1,07765	-0,43898	0	1
1,59705	0,11999	0,67226	-1,30419	-0,43898	0	0
-0,99755	2,02491	-1,78289	0,81019	-0,43898	0	0
0,68543	-0,56034	0,67226	0,88571	-0,43898	0	1
0,12444	-0,83248	0,01756	-1,00214	-0,43898	0	1
-0,15606	0,52818	-1,29186	2,09393	-0,43898	0	1
-0,22618	1,07245	-1,45553	-1,37970	-0,43898	0	0
1,03605	-0,15215	-1,29186	0,28160	2,27384	0	1
0,05431	-0,28821	-0,47347	-0,62457	-0,43898	0	1
-0,43656	-1,24067	0,83594	0,88571	-0,43898	0	1
-0,01581	-0,83248	0,18123	-1,30419	-0,43898	0	0
-0,50668	-0,01608	0,18123	0,13057	-0,43898	0	0
-0,29631	0,25605	-0,63715	-0,70008	-0,43898	0	1
-0,85730	-0,01608	-0,47347	0,96122	-0,43898	0	1
2,08792	-0,69641	1,49065	1,33879	2,27384	0	0
-1,06767	-1,24067	0,67226	0,50814	-0,43898	0	1
-0,15606	-1,37674	1,16330	-1,45522	-0,43898	0	2
-0,43656	1,20851	0,34491	1,79187	-0,43898	0	1
0,05431	1,34458	0,99962	0,20608	2,27384	0	1
1,80742	-0,83248	0,34491	-1,45522	-0,43898	0	1
-0,71705	0,66425	-0,47347	-0,54905	-0,43898	0	1
0,75556	-1,51281	1,16330	0,50814	2,27384	0	1

3,77090	-1,78494	1,98168	0,05506	-0,43898	0	1
0,33481	-0,96854	0,34491	-0,24700	-0,43898	0	1
-0,29631	0,11999	0,50859	0,13057	-0,43898	0	1
0,47506	-0,01608	-0,30980	0,28160	2,27384	0	0
0,61531	-1,37674	1,32697	-1,07765	-0,43898	0	1
0,75556	-0,69641	0,18123	-1,22868	-0,43898	0	1
0,82568	-0,69641	0,67226	0,73468	-0,43898	0	1
0,12444	-0,15215	-0,14612	-0,24700	-0,43898	0	1
3,06966	-0,56034	0,99962	-0,39803	-0,43898	0	1
-0,92743	-0,56034	0,01756	0,50814	-0,43898	0	0
0,82568	-1,10461	0,99962	-0,85111	-0,43898	0	1
0,12444	1,61671	-1,12818	1,64084	2,27384	0	1
0,54518	-1,37674	1,16330	-1,30419	-0,43898	0	1
1,03605	-0,69641	0,83594	0,05506	-0,43898	0	1
-0,92743	-1,64887	1,32697	-1,30419	-0,43898	0	2
0,82568	-0,42428	1,49065	-0,32251	-0,43898	0	1
-1,13780	0,25605	-0,80083	1,18776	-0,43898	0	0
-0,64693	-0,56034	0,01756	-0,39803	-0,43898	0	1
-1,13780	1,75278	0,01756	-0,77559	-0,43898	0	1
0,75556	-1,10461	0,83594	-0,54905	-0,43898	0	0
-1,13780	1,48065	-0,96450	-0,85111	-0,43898	0	1
0,40494	-1,10461	1,16330	1,11225	-0,43898	0	1
2,08792	0,11999	0,18123	-0,77559	-0,43898	0	0
-0,92743	-0,42428	-0,30980	-0,92662	-0,43898	0	0
0,05431	-0,01608	-1,29186	1,26328	-0,43898	0	1
0,26469	-0,01608	0,99962	-1,45522	-0,43898	0	1
0,47506	-0,83248	1,16330	-0,77559	2,27384	0	1
1,10618	-0,56034	1,16330	-1,22868	-0,43898	0	1
-0,85730	-0,28821	0,67226	-0,02046	-0,43898	0	1
1,10618	-0,01608	0,83594	0,05506	2,27384	0	1
0,19456	0,25605	0,34491	0,28160	-0,43898	0	1
-0,50668	1,07245	-2,11024	0,05506	2,27384	0	1
-1,13780	1,48065	-0,96450	-0,62457	-0,43898	0	1
0,89581	0,66425	-1,12818	1,18776	-0,43898	0	1
-0,22618	-1,51281	1,98168	-0,77559	-0,43898	0	1
-0,78718	-0,15215	-0,80083	-0,92662	-0,43898	0	1
-0,71705	0,25605	0,34491	-0,47354	-0,43898	0	1
-0,01581	0,39212	-0,96450	0,65917	2,27384	0	0
0,19456	-0,83248	-0,14612	-1,30419	-0,43898	0	1
1,59705	-0,56034	1,32697	0,05506	-0,43898	0	1
1,03605	-0,96854	0,67226	-0,70008	-0,43898	0	0
-1,06767	-0,69641	0,18123	-0,54905	-0,43898	0	1
-0,57680	0,66425	-0,47347	2,09393	-0,43898	0	1
-0,57680	1,20851	-0,47347	1,79187	-0,43898	0	1
-0,01581	-0,15215	1,65433	-0,02046	2,27384	0	1
-0,43656	0,93638	1,16330	-1,45522	-0,43898	0	0
-0,08593	-0,96854	1,65433	-1,30419	-0,43898	0	1
-1,20792	0,11999	-0,96450	-1,00214	-0,43898	0	1
-0,99755	-0,28821	-0,63715	-0,09597	2,27384	0	1
-0,71705	-0,56034	0,83594	-0,92662	-0,43898	0	1

-0,50668	-0,56034	-0,80083	1,11225	-0,43898	0	1
-0,36643	1,34458	0,50859	0,13057	-0,43898	0	1
2,01779	0,52818	-0,47347	0,81019	-0,43898	0	1
-1,20792	1,75278	-1,94656	-0,32251	-0,43898	0	1
-1,13780	2,02491	1,16330	1,11225	-0,43898	0	1
-0,85730	0,66425	1,16330	0,13057	-0,43898	0	2
-1,13780	-0,15215	-0,63715	-0,70008	-0,43898	0	1
1,45680	-0,15215	0,34491	-0,47354	-0,43898	0	2
0,12444	-1,37674	-0,63715	-0,70008	-0,43898	0	2
-0,15606	0,52818	-0,63715	-0,02046	-0,43898	0	1
-0,78718	-1,37674	1,32697	1,11225	-0,43898	0	1
-0,43656	1,61671	-1,61921	1,94290	-0,43898	0	1
1,24643	0,11999	0,34491	1,11225	-0,43898	0	0
-0,01581	-0,96854	0,50859	0,05506	-0,43898	0	1
1,73730	-1,64887	0,50859	-0,70008	2,27384	0	0
-0,29631	-0,69641	-0,80083	-1,07765	-0,43898	0	0
-0,71705	0,25605	-1,45553	-0,39803	-0,43898	0	0
-0,08593	-0,01608	-0,30980	1,41430	-0,43898	0	1
-0,29631	-0,15215	0,18123	0,13057	-0,43898	0	1
-0,92743	-0,15215	-0,30980	0,96122	-0,43898	0	0
-1,20792	0,25605	-2,43759	-1,22868	-0,43898	0	0
2,01779	-1,51281	1,49065	-1,45522	-0,43898	0	1
1,24643	-0,83248	0,99962	-0,02046	2,27384	0	1
-1,20792	0,66425	-1,29186	0,58365	-0,43898	0	0
-0,85730	0,11999	-0,80083	1,48982	-0,43898	0	0
-0,15606	0,80032	0,01756	-0,17148	-0,43898	0	1
0,89581	-1,10461	0,99962	-0,70008	2,27384	0	1
1,24643	-0,69641	0,50859	-0,85111	-0,43898	0	2
-0,85730	0,39212	-0,14612	-1,22868	-0,43898	0	0
0,54518	-0,15215	-0,30980	-0,54905	-0,43898	0	1
2,15804	-0,56034	0,83594	-0,17148	2,27384	0	1
1,73730	-0,83248	-0,30980	-0,17148	-0,43898	0	0
1,80742	-0,56034	0,01756	-1,22868	-0,43898	0	1
-0,36643	-0,01608	0,34491	1,26328	-0,43898	0	0
1,87755	-0,69641	1,81800	-0,54905	-0,43898	0	1
1,10618	-0,01608	-1,45553	-0,47354	-0,43898	0	1
-0,15606	1,75278	0,50859	1,18776	-0,43898	0	0
1,03605	0,80032	0,99962	-1,15316	-0,43898	0	1
-0,43656	-0,01608	-0,30980	1,94290	2,27384	0	1
-0,71705	-0,28821	0,18123	0,73468	-0,43898	0	1
0,47506	0,66425	-0,96450	-0,54905	-0,43898	0	0
1,38668	-0,83248	0,50859	-0,17148	-0,43898	0	1
1,73730	-0,83248	0,99962	-0,85111	-0,43898	0	2
-0,78718	1,34458	0,50859	1,79187	-0,43898	0	1
-0,78718	1,88884	-1,78289	1,41430	-0,43898	0	0
0,40494	0,25605	1,49065	0,20608	-0,43898	0	1
0,89581	-1,64887	0,99962	-0,54905	-0,43898	0	1
-0,08593	-0,15215	-0,47347	-1,45522	-0,43898	0	1
2,08792	-0,56034	0,83594	-0,39803	-0,43898	0	0
0,26469	-0,83248	0,50859	-0,77559	2,27384	0	1

-0,71705	-0,01608	-0,80083	0,13057	-0,43898	0	0
-0,78718	-0,01608	1,32697	1,56533	-0,43898	0	1
1,17630	-0,96854	1,65433	0,35711	-0,43898	0	1
0,75556	0,52818	0,99962	-0,85111	-0,43898	0	1
0,19456	-0,56034	0,01756	-1,00214	-0,43898	0	0
0,12444	-0,15215	-0,63715	0,35711	-0,43898	0	1
-0,78718	-0,56034	0,01756	-0,54905	-0,43898	0	1
-0,64693	-0,96854	0,18123	-1,45522	-0,43898	0	0
-0,15606	0,66425	0,83594	-0,70008	-0,43898	0	0
0,40494	1,75278	0,18123	0,65917	-0,43898	0	1
-0,15606	-0,28821	-0,14612	0,88571	-0,43898	0	1
-1,06767	0,80032	-0,14612	1,26328	-0,43898	0	1
1,59705	-1,10461	-0,14612	-0,47354	-0,43898	0	1
1,03605	-0,28821	0,18123	-0,92662	-0,43898	0	0
1,59705	-0,96854	0,50859	0,35711	-0,43898	0	1
-1,20792	-0,56034	0,67226	-0,85111	-0,43898	0	1
1,52692	-1,51281	0,34491	-1,30419	-0,43898	0	1
-0,71705	-0,01608	-1,12818	0,58365	-0,43898	0	0
1,38668	0,80032	-1,61921	0,05506	-0,43898	0	1
-0,01581	-0,15215	0,67226	1,03673	2,27384	0	1
-1,06767	1,07245	-0,14612	-0,85111	-0,43898	0	0
-0,64693	1,48065	-0,30980	-0,70008	2,27384	0	0
-0,85730	-0,28821	0,34491	1,56533	-0,43898	0	1
-0,43656	0,52818	0,34491	-0,17148	-0,43898	0	1
1,66717	-1,10461	1,65433	-0,39803	-0,43898	0	1
-0,29631	-0,28821	1,16330	1,48982	-0,43898	0	1
1,17630	-1,37674	1,81800	1,41430	2,27384	0	1
-0,71705	1,48065	-0,30980	-0,47354	-0,43898	0	1
-1,06767	0,11999	-0,96450	-0,09597	-0,43898	0	1
-1,20792	-1,64887	1,49065	0,43262	-0,43898	0	1
-0,99755	0,93638	-0,47347	1,79187	-0,43898	0	0
0,47506	-0,28821	-0,96450	-1,22868	-0,43898	0	1
-0,57680	0,66425	0,67226	1,18776	-0,43898	0	1
-0,99755	0,11999	0,50859	0,05506	-0,43898	0	1
0,61531	-0,42428	1,16330	-1,45522	-0,43898	0	0
-1,13780	-0,15215	-0,80083	0,73468	-0,43898	0	2
-0,08593	-0,28821	0,50859	0,05506	2,27384	0	1
-0,22618	-1,78494	1,49065	0,96122	2,27384	0	1
2,50866	-1,37674	1,65433	-1,15316	-0,43898	0	0
0,96593	-0,83248	0,18123	-1,00214	-0,43898	0	1
-1,06767	-0,01608	0,83594	0,20608	-0,43898	0	1
-0,15606	0,39212	-1,12818	0,28160	-0,43898	0	0
-0,22618	-0,28821	-0,30980	-0,24700	-0,43898	0	1
-0,43656	-0,01608	-0,30980	-1,07765	-0,43898	0	0
-0,57680	-0,83248	0,99962	0,28160	-0,43898	0	1
0,54518	-0,01608	0,67226	-1,07765	2,27384	0	1
0,82568	-0,42428	-0,47347	-0,24700	-0,43898	0	1
1,38668	0,25605	-0,63715	-1,30419	-0,43898	0	1
1,10618	-0,96854	0,83594	-0,24700	-0,43898	0	1
0,89581	-1,37674	-0,14612	-0,92662	2,27384	0	1

-0,92743	0,66425	-1,45553	0,35711	-0,43898	0	1
0,54518	-0,83248	0,50859	-0,32251	-0,43898	0	1
-1,06767	-0,42428	0,83594	1,33879	-0,43898	0	2
1,94767	1,48065	-0,30980	-0,24700	2,27384	0	1
1,52692	-0,96854	0,34491	-1,15316	-0,43898	0	1
-0,92743	0,25605	-0,63715	0,88571	-0,43898	0	1
0,40494	-1,78494	1,49065	-1,30419	-0,43898	0	1
-1,13780	2,29704	-1,94656	1,48982	-0,43898	0	0
-0,29631	1,07245	-0,80083	-1,30419	-0,43898	0	1
-1,13780	1,48065	0,18123	-0,47354	-0,43898	0	0
-0,92743	-0,15215	0,18123	0,28160	-0,43898	0	1
-0,64693	-0,01608	-0,14612	1,71636	-0,43898	0	1
-0,36643	-0,01608	-0,63715	-0,17148	-0,43898	0	1
-0,64693	-0,56034	0,50859	-1,30419	-0,43898	0	0
0,33481	0,80032	-1,29186	-0,54905	-0,43898	0	1
-0,15606	0,52818	-0,14612	0,05506	-0,43898	0	0
0,12444	-0,01608	-0,47347	0,96122	2,27384	0	0
-0,50668	-0,96854	0,34491	-0,92662	-0,43898	0	1
0,89581	-0,69641	0,18123	-1,07765	-0,43898	0	1
1,87755	-0,69641	-0,63715	-1,00214	-0,43898	0	0
1,24643	-0,96854	1,16330	-0,54905	-0,43898	0	1
1,31655	-1,37674	1,98168	-1,45522	-0,43898	0	1
1,24643	-0,01608	0,18123	-1,15316	-0,43898	0	1
0,12444	0,25605	-0,30980	-0,39803	2,27384	0	1
-0,50668	-0,96854	0,50859	-0,47354	-0,43898	0	0
-0,50668	-0,56034	0,34491	1,56533	2,27384	0	1
-0,92743	0,93638	-0,14612	-0,17148	-0,43898	0	1
0,47506	-0,96854	0,34491	-1,45522	-0,43898	0	1
-0,36643	1,48065	-1,12818	1,86739	-0,43898	0	1
-0,71705	-1,24067	0,99962	-1,07765	-0,43898	0	0
-1,06767	0,80032	0,01756	0,65917	-0,43898	0	0
2,22817	-1,24067	1,81800	-0,77559	-0,43898	0	1
-1,06767	0,93638	-0,30980	-1,22868	-0,43898	0	1
-0,92743	0,39212	-0,80083	1,33879	-0,43898	0	1
2,99953	-1,51281	0,50859	-1,30419	-0,43898	0	0
-0,71705	-0,15215	-0,63715	0,35711	2,27384	0	1
-0,71705	-0,56034	-1,94656	0,88571	-0,43898	0	1
-1,13780	2,29704	-1,78289	0,88571	-0,43898	0	0
-0,57680	-0,42428	0,01756	1,03673	-0,43898	0	0
-0,29631	-0,69641	0,01756	1,86739	2,27384	0	1
-1,06767	1,07245	1,16330	1,79187	-0,43898	0	0
-0,43656	1,07245	1,16330	1,03673	2,27384	0	1
-0,08593	-1,51281	0,83594	1,11225	-0,43898	0	1
-0,99755	1,34458	-0,80083	-0,77559	-0,43898	0	0
-0,43656	0,93638	-1,45553	1,03673	-0,43898	0	1
1,10618	-1,64887	1,49065	-0,39803	2,27384	0	1
-0,29631	-0,42428	0,01756	0,58365	-0,43898	0	1
0,96593	-0,01608	0,01756	0,13057	-0,43898	0	1
0,33481	-0,83248	0,83594	-0,17148	-0,43898	0	1
-0,85730	-1,10461	0,18123	-0,85111	-0,43898	0	1

0,12444	-1,37674	1,98168	-0,09597	-0,43898	0	1
1,31655	1,88884	-0,96450	0,88571	-0,43898	0	0
-0,22618	0,80032	-1,45553	0,20608	2,27384	0	1
0,68543	-1,10461	1,49065	0,58365	-0,43898	0	2
0,12444	-1,24067	0,83594	-1,22868	-0,43898	0	1
-1,13780	-0,96854	1,49065	0,50814	-0,43898	0	1
0,75556	0,25605	0,01756	-0,39803	-0,43898	0	1
1,03605	-0,96854	1,49065	-1,30419	2,27384	0	1
1,10618	1,88884	0,50859	-0,47354	2,27384	0	1
0,05431	-1,51281	0,18123	0,35711	-0,43898	0	2
-0,36643	1,48065	0,01756	-1,22868	-0,43898	0	1
-1,20792	2,29704	-2,11024	-1,00214	2,27384	0	0
0,26469	-0,01608	0,34491	-0,02046	-0,43898	0	1
0,96593	-0,83248	0,34491	0,96122	-0,43898	0	1
-1,06767	1,07245	-1,94656	0,05506	-0,43898	0	1
-1,06767	2,29704	-1,78289	-0,32251	-0,43898	0	0
0,26469	0,52818	0,67226	0,43262	-0,43898	0	1
0,54518	-0,15215	-0,63715	-0,85111	-0,43898	0	1
-0,15606	-1,37674	1,16330	-1,37970	-0,43898	0	1
-0,92743	0,93638	0,50859	0,20608	-0,43898	0	1
0,19456	-1,24067	0,50859	-0,17148	-0,43898	0	1
-0,71705	0,93638	-1,78289	-0,17148	-0,43898	0	0
-0,99755	-0,01608	0,34491	1,33879	-0,43898	0	1
-0,71705	0,25605	0,99962	1,11225	-0,43898	0	1
1,59705	-1,37674	0,01756	-1,45522	-0,43898	0	1
-1,13780	1,61671	-1,78289	1,41430	-0,43898	0	1
-1,13780	-0,42428	-0,14612	0,05506	-0,43898	0	0
-1,20792	2,29704	-2,43759	-1,22868	-0,43898	0	1
-0,29631	-0,01608	0,18123	1,03673	-0,43898	0	1
-0,92743	1,07245	-0,80083	0,65917	2,27384	0	1
-0,99755	1,61671	-1,78289	-1,15316	-0,43898	0	1
-1,20792	-1,10461	-0,63715	-1,30419	-0,43898	0	1
-1,20792	2,29704	-1,12818	0,65917	-0,43898	0	1
2,08792	-1,37674	0,83594	1,11225	2,27384	0	0
-0,99755	-0,15215	-1,45553	-1,22868	-0,43898	0	1
-0,08593	-1,64887	1,16330	1,11225	-0,43898	0	1
-0,64693	0,52818	-0,63715	1,26328	-0,43898	0	1
1,59705	-1,51281	1,98168	-0,09597	-0,43898	0	1
-0,08593	0,11999	0,18123	0,58365	-0,43898	0	0
-0,92743	0,93638	-1,45553	0,81019	-0,43898	0	2
-1,06767	-0,28821	0,18123	1,26328	-0,43898	0	2
-0,78718	0,11999	-1,12818	0,05506	-0,43898	0	1
-1,20792	0,25605	0,01756	1,11225	-0,43898	0	1
-0,92743	0,11999	0,18123	1,48982	-0,43898	0	1
-0,64693	2,16098	0,67226	-0,47354	-0,43898	0	1
-1,06767	-1,64887	1,81800	0,43262	-0,43898	0	1
-0,64693	1,34458	-0,30980	-0,17148	-0,43898	0	1
1,03605	-0,01608	-0,63715	0,65917	-0,43898	0	1
-0,99755	-0,83248	0,99962	-0,47354	-0,43898	0	1
-0,57680	-0,01608	1,49065	1,48982	-0,43898	0	0

-1,20792	-1,37674	0,83594	-1,45522	-0,43898	0	1
2,22817	-1,51281	1,81800	1,11225	-0,43898	0	1
3,42028	-1,51281	0,83594	-1,37970	-0,43898	0	1
-0,43656	0,11999	-0,80083	1,18776	-0,43898	0	1
-0,92743	0,93638	0,50859	-0,70008	2,27384	0	0
0,47506	-0,83248	1,16330	-0,70008	2,27384	0	1
1,31655	-0,28821	0,99962	-0,70008	2,27384	0	0
-0,36643	0,52818	0,01756	1,03673	-0,43898	0	0
0,40494	-1,51281	1,16330	-1,30419	-0,43898	0	1
-1,20792	0,52818	-1,78289	-1,30419	-0,43898	0	1
-0,22618	1,20851	-0,47347	0,65917	-0,43898	0	0
3,20991	0,25605	-1,78289	-1,22868	2,27384	0	0
1,59705	-1,10461	0,50859	-1,22868	-0,43898	0	1
0,89581	-0,56034	0,50859	-1,07765	-0,43898	0	1
-0,71705	-0,15215	0,67226	-1,15316	2,27384	0	1
-1,20792	-0,42428	0,18123	-0,92662	-0,43898	0	0
0,89581	-0,83248	1,65433	-1,07765	-0,43898	0	0
0,75556	-0,83248	0,18123	0,58365	2,27384	0	1
1,03605	-0,28821	0,83594	0,05506	-0,43898	0	1
1,31655	-0,56034	1,16330	1,86739	-0,43898	0	1
-0,57680	1,07245	-1,29186	1,41430	-0,43898	0	1
1,03605	-1,37674	0,18123	-0,24700	-0,43898	0	1
0,40494	0,25605	-0,63715	-0,02046	-0,43898	0	1
-0,64693	-0,15215	0,67226	1,03673	-0,43898	0	1
1,10618	-1,37674	0,01756	-0,09597	-0,43898	0	2
-0,01581	-0,28821	0,34491	-1,30419	-0,43898	0	1
-0,71705	0,25605	-1,12818	-0,70008	2,27384	0	1
0,05431	1,20851	-0,47347	-0,85111	-0,43898	0	1
0,82568	-1,64887	0,99962	0,20608	-0,43898	0	0
-0,92743	-0,56034	-1,29186	0,35711	-0,43898	0	1
-0,15606	2,16098	0,18123	1,03673	-0,43898	0	1
0,68543	-1,24067	1,49065	-1,07765	-0,43898	0	2
-0,99755	0,11999	0,18123	-0,62457	-0,43898	0	1
0,96593	-0,56034	1,49065	-1,15316	-0,43898	0	1
0,54518	-0,56034	0,67226	1,26328	-0,43898	0	1
-1,20792	2,02491	-0,14612	0,50814	-0,43898	0	1
-1,06767	0,11999	0,01756	1,26328	-0,43898	0	0
-0,22618	0,93638	-0,14612	0,05506	-0,43898	0	1
0,05431	-1,37674	1,81800	1,26328	-0,43898	0	0
2,29829	-1,51281	1,32697	1,18776	-0,43898	0	1
-0,36643	-0,56034	-0,14612	-1,30419	-0,43898	0	1
0,12444	-1,10461	1,81800	-0,32251	-0,43898	0	1
0,40494	0,25605	1,16330	-0,47354	2,27384	0	1
-0,15606	-0,96854	0,50859	-1,45522	-0,43898	0	1
-0,36643	0,25605	0,67226	0,88571	-0,43898	0	1
1,17630	-0,96854	0,50859	-0,77559	-0,43898	0	1
0,05431	-0,69641	0,34491	1,03673	-0,43898	0	1
0,89581	-0,42428	1,32697	0,20608	-0,43898	0	0
-0,99755	0,66425	-1,12818	-0,77559	-0,43898	0	0
-0,71705	0,25605	-1,12818	-0,32251	-0,43898	0	0

-0,85730	-0,01608	0,34491	-1,00214	-0,43898	0	1
-1,06767	2,16098	-1,78289	0,81019	-0,43898	0	0
-1,06767	0,93638	-1,45553	0,96122	-0,43898	0	0
-0,71705	1,48065	-1,94656	-1,30419	-0,43898	0	1
0,68543	-0,42428	-0,14612	0,13057	-0,43898	0	1
1,24643	-1,24067	1,49065	-1,37970	-0,43898	0	1
-0,43656	-0,01608	-1,29186	1,03673	-0,43898	0	1
0,68543	-0,69641	0,99962	0,43262	-0,43898	0	0
-0,22618	0,93638	0,01756	0,43262	2,27384	0	1
-0,64693	-0,28821	0,01756	-1,45522	-0,43898	0	2
0,12444	0,52818	0,50859	0,65917	2,27384	0	1
-0,15606	-0,42428	-0,80083	-0,17148	-0,43898	0	1
-0,99755	2,02491	-1,61921	0,88571	2,27384	0	1
1,87755	-1,37674	0,99962	-1,45522	-0,43898	0	1
1,52692	0,66425	0,18123	1,86739	2,27384	0	0
-0,57680	-0,56034	0,01756	0,96122	-0,43898	0	1
2,64891	-1,24067	1,65433	-0,70008	-0,43898	0	1
1,73730	0,52818	-0,14612	-1,07765	2,27384	0	1
-0,64693	-0,15215	-0,63715	1,03673	-0,43898	0	1
0,05431	-0,69641	1,81800	-0,32251	-0,43898	0	1
-0,71705	-0,56034	-0,63715	-0,70008	-0,43898	0	1
1,87755	-1,64887	0,99962	0,05506	-0,43898	0	1
-0,78718	1,20851	-0,80083	0,65917	2,27384	0	1
0,96593	-1,24067	0,50859	-0,54905	-0,43898	0	1
-0,57680	-1,24067	0,50859	-1,07765	-0,43898	0	1
-0,43656	0,66425	-0,63715	0,50814	-0,43898	0	0
-0,57680	-1,24067	-0,14612	0,43262	-0,43898	0	1
-0,78718	0,93638	-0,80083	-0,85111	-0,43898	0	2
0,19456	-1,51281	0,99962	-1,15316	-0,43898	0	1
0,05431	-0,01608	1,32697	-0,09597	-0,43898	0	1
-0,99755	-0,83248	1,32697	1,86739	-0,43898	0	1
-0,15606	0,80032	1,49065	1,11225	-0,43898	0	1
1,73730	-0,01608	-0,47347	-0,39803	-0,43898	0	1
-0,64693	1,07245	-0,30980	0,88571	-0,43898	0	1
0,05431	0,11999	-0,14612	1,41430	-0,43898	0	1
0,89581	-1,51281	0,18123	-0,09597	-0,43898	0	1
-1,13780	1,61671	-0,63715	0,96122	-0,43898	0	1
-0,43656	0,66425	0,18123	-1,15316	-0,43898	0	1
1,24643	-0,42428	-0,14612	-1,37970	-0,43898	0	0
0,61531	-1,64887	-0,14612	-1,00214	-0,43898	0	1
-0,08593	-0,69641	0,67226	0,20608	-0,43898	0	1
-0,01581	0,52818	-0,30980	-1,15316	-0,43898	0	0
-0,64693	-0,56034	0,50859	0,50814	-0,43898	0	1
0,96593	-0,42428	0,34491	-1,22868	-0,43898	0	1
0,33481	0,11999	0,50859	1,03673	-0,43898	0	1
-0,92743	-0,42428	-0,63715	-0,85111	-0,43898	0	1
-0,50668	2,29704	-2,11024	1,64084	-0,43898	0	0
-0,29631	1,20851	-0,30980	0,96122	-0,43898	0	1
-0,15606	-0,28821	0,50859	0,81019	2,27384	0	1
0,47506	-0,28821	0,01756	1,41430	2,27384	0	1

-1,13780	1,34458	-1,12818	0,88571	-0,43898	0	0
-1,15704	0,61571	-0,33553	0,01631	-0,83321	1	1
-1,07950	0,77750	-1,38284	1,36501	1,19351	1	1
-0,84689	1,10108	-1,73195	-1,09439	1,19351	1	1
0,70387	-0,03146	-0,33553	-0,61837	-0,83321	1	2
-0,22658	-0,51683	-0,16098	-0,93572	1,19351	1	1
-0,14905	-0,35504	-0,16098	-0,85638	-0,83321	1	1
0,70387	-0,51683	0,88634	-0,38037	1,19351	1	1
-1,15704	1,42466	-0,33553	-1,49106	1,19351	1	2
0,39372	-0,19325	0,18813	0,41298	-0,83321	1	2
-0,07151	-0,19325	-1,20829	-0,45970	1,19351	1	1
-0,84689	1,42466	-1,55740	1,60301	1,19351	1	1
-1,00197	1,26287	-1,55740	-1,17372	-0,83321	1	1
1,94449	-0,03146	0,01358	-0,77704	-0,83321	1	2
-0,45920	-0,67863	1,23544	-0,06303	1,19351	1	2
-1,15704	-0,03146	-1,55740	-1,25306	1,19351	1	1
1,16910	0,13033	2,10821	0,41298	-0,83321	1	2
2,64233	-0,19325	-0,68463	1,44434	1,19351	1	1
0,62634	-1,97296	0,88634	-1,41173	-0,83321	1	1
-0,53674	-0,03146	-1,03374	0,80966	-0,83321	1	1
-0,53674	1,42466	-0,16098	0,25431	-0,83321	1	1
-0,61428	1,26287	-0,68463	-0,53904	-0,83321	1	2
-1,23458	1,10108	-1,55740	-0,38037	-0,83321	1	1
1,55680	0,45392	-0,16098	-1,57040	-0,83321	1	2
-0,14905	-0,03146	-0,16098	-0,06303	-0,83321	1	1
0,47126	-2,13475	1,93365	1,04767	1,19351	1	2
0,39372	0,29212	0,88634	-1,41173	1,19351	1	2
0,47126	-0,35504	0,01358	0,96833	1,19351	1	1
-0,61428	1,10108	0,01358	1,20634	1,19351	1	1
-0,30412	-0,84042	0,53723	-1,41173	-0,83321	1	1
-0,38166	-1,00221	-0,16098	-1,09439	-0,83321	1	1
-0,84689	1,26287	0,18813	-1,25306	1,19351	1	1
-0,45920	0,77750	0,01358	-0,85638	1,19351	1	2
-1,23458	0,77750	-0,51008	0,25431	-0,83321	1	2
-0,45920	-0,19325	0,53723	-1,41173	-0,83321	1	1
-1,07950	0,93929	-1,38284	0,09564	-0,83321	1	1
2,79741	-2,13475	0,88634	-0,30103	-0,83321	1	2
0,85895	-0,84042	1,06089	-1,41173	1,19351	1	2
-0,30412	1,10108	1,58455	1,04767	-0,83321	1	2
1,47926	-1,16400	1,75910	1,28567	-0,83321	1	2
0,78141	-0,67863	0,53723	-1,17372	1,19351	1	2
-0,22658	-1,16400	-0,85919	-0,45970	1,19351	1	2
0,16111	-0,67863	-0,68463	0,01631	1,19351	1	2
1,09157	-0,51683	0,36268	-1,41173	1,19351	1	2
0,62634	1,74825	0,88634	0,01631	-0,83321	1	2
1,63433	0,61571	0,53723	1,60301	-0,83321	1	2
-0,22658	2,39541	-1,55740	-1,01505	1,19351	1	1
1,63433	0,45392	0,36268	-0,45970	-0,83321	1	2
-0,30412	-1,16400	1,06089	-0,61837	-0,83321	1	1
-0,22658	-0,03146	-0,33553	1,60301	1,19351	1	1

0,16111	-0,19325	-0,51008	0,96833	-0,83321	1	2
0,85895	-0,51683	1,23544	-1,09439	-0,83321	1	2
-0,53674	-0,03146	0,36268	1,04767	1,19351	1	2
-0,14905	-1,16400	1,93365	0,80966	-0,83321	1	2
-1,23458	2,07183	-0,33553	0,80966	1,19351	1	2
-0,14905	-1,16400	0,18813	-1,25306	-0,83321	1	2
1,78941	-0,84042	1,06089	0,73033	1,19351	1	2
-0,84689	0,29212	-0,51008	-1,01505	-0,83321	1	1
-1,00197	0,61571	-0,85919	1,28567	-0,83321	1	1
0,62634	-1,48758	1,41000	1,12700	1,19351	1	1
-0,14905	-0,19325	-1,38284	-0,53904	-0,83321	1	2
0,54880	-1,32579	0,88634	0,33365	-0,83321	1	1
-0,92443	-0,19325	-0,68463	-0,06303	-0,83321	1	2
-0,53674	1,10108	0,88634	0,65099	1,19351	1	2
0,31618	-0,35504	1,41000	-0,69771	-0,83321	1	2
-0,30412	-1,81117	0,88634	-1,01505	-0,83321	1	2
0,08357	-0,03146	0,36268	0,09564	-0,83321	1	2
0,93649	0,77750	-1,20829	1,28567	-0,83321	1	2
-1,00197	-0,35504	0,36268	-0,77704	-0,83321	1	2
-1,15704	-0,19325	0,36268	-0,53904	1,19351	1	1
3,10756	-2,13475	2,28276	0,88900	-0,83321	1	2
0,62634	1,10108	-1,03374	0,96833	1,19351	1	2
-0,53674	1,42466	-0,85919	1,36501	-0,83321	1	1
0,08357	-1,64937	1,41000	-0,30103	1,19351	1	1
0,31618	0,93929	-0,85919	-1,33239	1,19351	1	1
-0,92443	-1,48758	0,71179	0,65099	-0,83321	1	2
-0,69181	1,42466	0,36268	0,25431	-0,83321	1	2
2,17710	-0,67863	0,71179	1,76168	-0,83321	1	2
0,08357	0,45392	-1,38284	-0,38037	-0,83321	1	1
-0,30412	0,29212	-0,16098	1,76168	-0,83321	1	2
-1,23458	0,45392	-0,33553	1,84102	-0,83321	1	2
-0,30412	0,45392	-0,51008	0,09564	-0,83321	1	1
-0,92443	1,74825	-0,68463	2,15836	1,19351	1	2
0,54880	-0,19325	-0,68463	0,09564	1,19351	1	2
2,56479	-1,48758	1,58455	1,52368	1,19351	1	2
0,23865	0,13033	-0,51008	-1,41173	1,19351	1	2
1,55680	0,13033	0,53723	1,04767	-0,83321	1	2
0,23865	-0,19325	0,88634	1,44434	1,19351	1	2
-0,61428	1,74825	-0,33553	1,68235	1,19351	1	2
-1,00197	-1,00221	-0,16098	-1,09439	-0,83321	1	1
0,93649	0,13033	-0,51008	0,49232	-0,83321	1	1
0,62634	-1,32579	0,88634	1,52368	-0,83321	1	2
-1,23458	-0,84042	0,53723	0,33365	-0,83321	1	1
-0,76935	0,93929	-1,73195	-0,69771	1,19351	1	1
2,25464	0,13033	0,18813	1,20634	1,19351	1	1
-1,07950	0,13033	1,58455	0,65099	-0,83321	1	1
-0,69181	1,10108	-0,16098	0,73033	1,19351	1	2
0,23865	-1,16400	1,75910	-0,45970	1,19351	1	2
-1,23458	0,13033	1,58455	0,33365	-0,83321	1	2
-0,45920	0,45392	-1,20829	0,09564	1,19351	1	1

2,25464	-0,35504	1,58455	0,80966	1,19351	1	2
-0,69181	0,45392	0,01358	-0,77704	1,19351	1	2
-1,07950	0,61571	-0,85919	1,99969	-0,83321	1	1
0,23865	0,93929	0,88634	-0,06303	1,19351	1	1
-0,61428	0,93929	-1,03374	0,33365	1,19351	1	1
-1,23458	0,77750	-1,03374	0,80966	-0,83321	1	1
0,93649	-0,19325	-0,16098	-0,77704	-0,83321	1	1
0,62634	1,74825	-0,33553	1,68235	1,19351	1	2
-0,14905	-0,35504	-1,38284	-0,45970	1,19351	1	1
-0,14905	-0,51683	-0,68463	-1,01505	1,19351	1	2
-1,00197	0,13033	-0,68463	0,09564	-0,83321	1	1
0,31618	-0,51683	0,71179	-0,06303	1,19351	1	2
-1,15704	1,91004	-1,20829	-0,14236	1,19351	1	1
-0,92443	-0,19325	-1,03374	-0,77704	-0,83321	1	1
-0,76935	1,42466	-1,55740	0,57165	-0,83321	1	1
-0,45920	0,29212	0,53723	0,96833	-0,83321	1	2
1,24664	-1,97296	1,06089	-1,17372	-0,83321	1	1
-0,69181	-0,03146	-0,51008	-1,49106	1,19351	1	1
-0,92443	-0,67863	-1,55740	-0,45970	-0,83321	1	1
-0,61428	-0,35504	0,53723	-1,41173	1,19351	1	2
-1,00197	2,39541	-0,68463	2,39637	1,19351	1	1
-0,45920	-1,32579	0,88634	-1,09439	1,19351	1	1
0,78141	-0,84042	1,06089	-1,01505	1,19351	1	2
-1,07950	1,42466	-1,38284	-1,17372	-0,83321	1	1
0,54880	-1,00221	1,41000	-0,77704	-0,83321	1	1
-0,53674	-0,19325	-0,85919	0,17498	-0,83321	1	1
-0,53674	-0,03146	-1,20829	-1,49106	-0,83321	1	1
-1,23458	1,26287	-1,38284	-0,69771	-0,83321	1	1
-1,07950	0,29212	0,18813	-1,17372	1,19351	1	1
0,08357	0,61571	-0,16098	-0,69771	-0,83321	1	1
-0,92443	0,13033	0,01358	-1,17372	-0,83321	1	1
0,54880	0,61571	-0,33553	-1,09439	-0,83321	1	1
0,31618	-0,51683	0,01358	-0,30103	-0,83321	1	2
1,63433	-0,67863	0,18813	0,88900	1,19351	1	2
0,00603	-1,32579	0,36268	0,65099	-0,83321	1	2
-0,22658	-0,03146	0,53723	-0,30103	-0,83321	1	1
-0,45920	-1,16400	0,88634	1,20634	1,19351	1	1
-0,76935	1,74825	-1,38284	1,44434	1,19351	1	2
-0,45920	-0,03146	0,18813	-0,53904	1,19351	1	1
-0,61428	-0,84042	0,71179	-0,85638	-0,83321	1	2
-0,38166	-0,35504	0,18813	1,84102	1,19351	1	1
-0,45920	0,29212	-1,03374	0,17498	1,19351	1	1
1,24664	-1,64937	1,23544	-0,53904	-0,83321	1	2
-0,84689	-1,00221	-1,03374	0,57165	-0,83321	1	1
1,24664	-1,48758	1,23544	0,49232	-0,83321	1	2
1,63433	-1,32579	1,23544	-0,14236	-0,83321	1	2
0,00603	-1,64937	1,23544	0,73033	-0,83321	1	1
-0,84689	0,13033	-0,51008	0,25431	-0,83321	1	1
-0,69181	-0,84042	0,01358	-0,30103	-0,83321	1	2
1,09157	-0,19325	1,23544	0,57165	-0,83321	1	2

-0,14905	0,77750	-0,33553	0,88900	1,19351	1	1
-1,00197	0,93929	-2,25561	0,80966	-0,83321	1	1
0,23865	-0,03146	1,58455	-1,01505	-0,83321	1	1
-1,15704	2,07183	-1,38284	1,52368	-0,83321	1	1
-0,92443	-0,35504	0,36268	-0,77704	-0,83321	1	2
0,00603	0,29212	-0,16098	0,88900	-0,83321	1	2
0,16111	0,45392	0,01358	-0,53904	1,19351	1	2
-0,92443	1,42466	0,36268	-0,77704	-0,83321	1	1
0,08357	-0,19325	-0,68463	-0,61837	-0,83321	1	1
0,70387	-1,16400	0,53723	-1,49106	1,19351	1	2
-0,07151	-1,48758	1,23544	-1,17372	1,19351	1	2
1,24664	-1,00221	0,53723	-0,22170	1,19351	1	2
2,25464	-1,64937	2,28276	-0,53904	-0,83321	1	2
-1,07950	0,29212	-1,20829	0,41298	1,19351	1	2
0,93649	-0,03146	-0,68463	-1,01505	-0,83321	1	1
1,71187	0,13033	-1,38284	-1,17372	-0,83321	1	1
-1,23458	2,39541	-1,55740	-0,77704	1,19351	1	1
-0,45920	0,29212	1,41000	1,28567	-0,83321	1	2
-0,69181	0,93929	-1,38284	-0,93572	-0,83321	1	1
1,86695	0,13033	0,01358	-1,41173	1,19351	1	1
2,79741	0,13033	-1,20829	1,12700	-0,83321	1	2
0,78141	-0,03146	0,36268	-0,61837	1,19351	1	1
1,01403	-0,67863	1,41000	-0,30103	-0,83321	1	1
0,16111	0,61571	0,01358	0,57165	-0,83321	1	2
-0,84689	-0,51683	0,88634	-0,06303	-0,83321	1	2
-0,61428	0,77750	-1,55740	1,36501	-0,83321	1	1
-1,15704	0,93929	-1,20829	-0,69771	1,19351	1	2
-0,92443	0,13033	-0,33553	0,88900	-0,83321	1	2
0,47126	-1,00221	0,36268	-1,64973	-0,83321	1	2
-0,61428	-0,67863	-0,85919	0,73033	-0,83321	1	1
0,54880	-1,32579	0,71179	1,12700	-0,83321	1	2
