# Supplementary material for: Applying Corrigan’s progressive model of self-stigma to people with depression
Source: PLoS One. 2019 Oct 29;14(10):e0224418. doi: 10.1371/journal.pone.0224418 (PMC6818799; doi:10.1371/journal.pone.0224418)
Supplement: S2 File — (PDF) [file pone.0224418.s002.pdf]

Means	Var	Sample
56,00	1,00	1,00
66,00	1,00	1,00
58,00	1,00	1,00
12,00	1,00	1,00
42,00	1,00	1,00
58,00	1,00	1,00
60,00	1,00	1,00
60,00	1,00	1,00
44,00	1,00	1,00
13,00	1,00	1,00
74,00	1,00	1,00
80,00	1,00	1,00
55,00	1,00	1,00
77,00	1,00	1,00
46,00	1,00	1,00
32,00	1,00	1,00
35,00	1,00	1,00
50,00	1,00	1,00
60,00	1,00	1,00
48,00	1,00	1,00
54,00	1,00	1,00
54,00	1,00	1,00
31,00	1,00	1,00
64,00	1,00	1,00
51,00	1,00	1,00
35,00	1,00	1,00
62,00	1,00	1,00
10,00	1,00	1,00
34,00	1,00	1,00
45,00	1,00	1,00
61,00	1,00	1,00
54,00	1,00	1,00
62,00	1,00	1,00
64,00	1,00	1,00
54,00	1,00	1,00
49,00	1,00	1,00
55,00	1,00	1,00
26,00	1,00	1,00
67,00	1,00	1,00
52,00	1,00	1,00
36,00	1,00	1,00
27,00	1,00	1,00
13,00	1,00	1,00
62,00	1,00	1,00
15,00	1,00	1,00
36,00	1,00	1,00
62,00	1,00	1,00
63,00	1,00	1,00
51,00	1,00	1,00

64,00	1,00	1,00
90,00	1,00	1,00
58,00	1,00	1,00
37,00	1,00	1,00
28,00	1,00	1,00
22,00	1,00	1,00
52,00	1,00	1,00
50,00	1,00	1,00
41,00	1,00	1,00
22,00	1,00	1,00
42,00	1,00	1,00
24,00	1,00	1,00
43,00	1,00	1,00
60,00	1,00	1,00
55,00	1,00	1,00
34,00	1,00	1,00
66,00	1,00	1,00
49,00	1,00	1,00
73,00	1,00	1,00
59,00	1,00	1,00
24,00	1,00	1,00
74,00	1,00	1,00
34,00	1,00	1,00
57,00	1,00	1,00
45,00	1,00	1,00
50,00	1,00	1,00
79,00	1,00	1,00
84,00	1,00	1,00
61,00	1,00	1,00
66,00	1,00	1,00
47,00	1,00	1,00
32,00	1,00	1,00
57,00	1,00	1,00
61,00	1,00	1,00
42,00	1,00	1,00
62,00	1,00	1,00
55,00	1,00	1,00
73,00	1,00	1,00
30,00	1,00	1,00
62,00	1,00	1,00
43,00	1,00	1,00
50,00	1,00	1,00
59,00	1,00	1,00
73,00	1,00	1,00
45,00	1,00	1,00
52,00	1,00	1,00
38,00	1,00	1,00
42,00	1,00	1,00
71,00	1,00	1,00
69,00	1,00	1,00

46,00	1,00	1,00
23,00	1,00	1,00
27,00	1,00	1,00
63,00	1,00	1,00
36,00	1,00	1,00
56,00	1,00	1,00
50,00	1,00	1,00
67,00	1,00	1,00
59,00	1,00	1,00
45,00	1,00	1,00
56,00	1,00	1,00
67,00	1,00	1,00
19,00	1,00	1,00
50,00	1,00	1,00
56,00	1,00	1,00
69,00	1,00	1,00
45,00	1,00	1,00
64,00	1,00	1,00
16,00	1,00	1,00
64,00	1,00	1,00
37,00	1,00	1,00
17,00	1,00	1,00
64,00	1,00	1,00
37,00	1,00	1,00
52,00	1,00	1,00
33,00	1,00	1,00
56,00	1,00	1,00
61,00	1,00	1,00
57,00	1,00	1,00
75,00	1,00	1,00
46,00	1,00	1,00
56,00	1,00	1,00
18,00	1,00	1,00
73,00	1,00	1,00
33,00	1,00	1,00
72,00	1,00	1,00
47,00	1,00	1,00
59,00	1,00	1,00
47,00	1,00	1,00
52,00	1,00	1,00
63,00	1,00	1,00
17,00	1,00	1,00
66,00	1,00	1,00
60,00	1,00	1,00
35,00	1,00	1,00
31,00	1,00	1,00
33,00	1,00	1,00
65,00	1,00	1,00
58,00	1,00	1,00
74,00	1,00	1,00

58,00	1,00	1,00
76,00	1,00	1,00
10,00	1,00	1,00
69,00	1,00	1,00
57,00	1,00	1,00
14,00	1,00	1,00
61,00	1,00	1,00
57,00	1,00	1,00
36,00	1,00	1,00
59,00	1,00	1,00
61,00	1,00	1,00
56,00	1,00	1,00
55,00	1,00	1,00
54,00	1,00	1,00
47,00	1,00	1,00
70,00	1,00	1,00
42,00	1,00	1,00
28,00	1,00	1,00
54,00	1,00	1,00
12,00	1,00	1,00
50,00	1,00	1,00
41,00	1,00	1,00
41,00	1,00	1,00
51,00	1,00	1,00
67,00	1,00	1,00
38,00	1,00	1,00
69,00	1,00	1,00
59,00	1,00	1,00
70,00	1,00	1,00
30,00	1,00	1,00
56,00	1,00	1,00
50,00	1,00	1,00
53,00	1,00	1,00
47,00	1,00	1,00
70,00	1,00	1,00
38,00	1,00	1,00
63,00	1,00	1,00
59,00	1,00	1,00
58,00	1,00	1,00
36,00	1,00	1,00
49,00	1,00	1,00
56,00	1,00	1,00
22,00	1,00	1,00
25,00	1,00	1,00
90,00	1,00	1,00
36,00	1,00	1,00
69,00	1,00	1,00
37,00	1,00	1,00
54,00	1,00	1,00
65,00	1,00	1,00

90,00	1,00	1,00
66,00	1,00	1,00
37,00	1,00	1,00
58,00	1,00	1,00
43,00	1,00	1,00
20,00	1,00	1,00
83,00	1,00	1,00
67,00	1,00	1,00
45,00	1,00	1,00
63,00	1,00	1,00
68,00	1,00	1,00
53,00	1,00	1,00
65,00	1,00	1,00
58,00	1,00	1,00
27,00	1,00	1,00
90,00	1,00	1,00
46,00	1,00	1,00
50,00	1,00	1,00
10,00	1,00	1,00
63,00	1,00	1,00
42,00	1,00	1,00
41,00	1,00	1,00
53,00	1,00	1,00
68,00	1,00	1,00
63,00	1,00	1,00
26,00	1,00	1,00
70,00	1,00	1,00
81,00	1,00	1,00
63,00	1,00	1,00
70,00	1,00	1,00
65,00	1,00	1,00
55,00	1,00	1,00
61,00	1,00	1,00
76,00	1,00	1,00
14,00	1,00	1,00
44,00	1,00	1,00
61,00	1,00	1,00
26,00	1,00	1,00
63,00	1,00	1,00
75,00	1,00	1,00
43,00	1,00	1,00
80,00	1,00	1,00
27,00	1,00	1,00
85,00	1,00	1,00
52,00	1,00	1,00
53,00	1,00	1,00
61,00	1,00	1,00
26,00	1,00	1,00
27,00	1,00	1,00
34,00	1,00	1,00

39,00	1,00	1,00
54,00	1,00	1,00
80,00	1,00	1,00
22,00	1,00	1,00
66,00	1,00	1,00
55,00	1,00	1,00
35,00	1,00	1,00
60,00	1,00	1,00
57,00	1,00	1,00
66,00	1,00	1,00
45,00	1,00	1,00
54,00	1,00	1,00
68,00	1,00	1,00
82,00	1,00	1,00
43,00	1,00	1,00
57,00	1,00	1,00
62,00	1,00	1,00
26,00	1,00	1,00
57,00	1,00	1,00
61,00	1,00	1,00
38,00	1,00	1,00
61,00	1,00	1,00
42,00	1,00	1,00
64,00	1,00	1,00
11,00	1,00	1,00
48,00	1,00	1,00
82,00	1,00	1,00
53,00	1,00	1,00
53,00	1,00	1,00
18,00	1,00	1,00
43,00	1,00	1,00
39,00	1,00	1,00
60,00	1,00	1,00
27,00	1,00	1,00
79,00	1,00	1,00
56,00	1,00	1,00
30,00	1,00	1,00
63,00	1,00	1,00
27,00	1,00	1,00
58,00	1,00	1,00
74,00	1,00	1,00
33,00	1,00	1,00
52,00	1,00	1,00
24,00	1,00	1,00
74,00	1,00	1,00
67,00	1,00	1,00
44,00	1,00	1,00
50,00	1,00	1,00
45,00	1,00	1,00
20,00	1,00	1,00

45,00	1,00	1,00
78,00	1,00	1,00
79,00	1,00	1,00
45,00	1,00	1,00
56,00	1,00	1,00
79,00	1,00	1,00
55,00	1,00	1,00
11,00	1,00	1,00
57,00	1,00	1,00
62,00	1,00	1,00
61,00	1,00	1,00
60,00	1,00	1,00
41,00	1,00	1,00
74,00	1,00	1,00
44,00	1,00	1,00
58,00	1,00	1,00
68,00	1,00	1,00
53,00	1,00	1,00
38,00	1,00	1,00
61,00	1,00	1,00
38,00	1,00	1,00
44,00	1,00	1,00
53,00	1,00	1,00
18,00	1,00	1,00
41,00	1,00	1,00
65,00	1,00	1,00
68,00	1,00	1,00
53,00	1,00	1,00
73,00	1,00	1,00
78,00	1,00	1,00
49,00	1,00	1,00
21,00	1,00	1,00
48,00	1,00	1,00
68,00	1,00	1,00
68,00	1,00	1,00
64,00	1,00	1,00
43,00	1,00	1,00
76,00	1,00	1,00
70,00	1,00	1,00
71,00	1,00	1,00
46,00	1,00	1,00
75,00	1,00	1,00
53,00	1,00	1,00
60,00	1,00	1,00
86,00	1,00	1,00
57,00	1,00	1,00
63,00	1,00	1,00
30,00	1,00	1,00
73,00	1,00	1,00
62,00	1,00	1,00

67,00	1,00	1,00
67,00	1,00	1,00
69,00	1,00	1,00
25,00	1,00	1,00
51,00	1,00	1,00
44,00	1,00	1,00
55,00	1,00	1,00
17,00	1,00	1,00
62,00	1,00	1,00
60,00	1,00	1,00
50,00	1,00	1,00
69,00	1,00	1,00
48,00	1,00	1,00
62,00	1,00	1,00
47,00	1,00	1,00
51,00	1,00	1,00
36,00	1,00	1,00
58,00	1,00	1,00
79,00	1,00	1,00
63,00	1,00	1,00
58,00	1,00	1,00
78,00	1,00	1,00
33,00	1,00	1,00
47,00	1,00	1,00
53,00	1,00	1,00
55,00	1,00	1,00
27,00	1,00	1,00
23,00	1,00	1,00
61,00	1,00	1,00
78,00	1,00	1,00
48,00	1,00	1,00
60,00	1,00	1,00
75,00	1,00	1,00
58,00	1,00	1,00
41,00	1,00	1,00
58,00	1,00	1,00
65,00	1,00	1,00
57,00	1,00	1,00
58,00	1,00	1,00
70,00	1,00	1,00
13,00	1,00	1,00
38,00	1,00	1,00
34,00	1,00	1,00
53,00	1,00	1,00
53,00	1,00	1,00
59,00	1,00	1,00
26,00	1,00	1,00
67,00	1,00	1,00
49,00	1,00	1,00
61,00	1,00	1,00

43,00	1,00	1,00
51,00	1,00	1,00
34,00	1,00	1,00
44,00	1,00	1,00
90,00	1,00	1,00
63,00	1,00	1,00
40,00	1,00	1,00
53,00	1,00	1,00
64,00	1,00	1,00
51,00	1,00	1,00
49,00	1,00	1,00
58,00	1,00	1,00
37,00	1,00	1,00
55,00	1,00	1,00
67,00	1,00	1,00
46,00	1,00	1,00
47,00	1,00	1,00
49,00	1,00	1,00
70,00	1,00	1,00
14,00	1,00	1,00
52,00	1,00	1,00
30,00	1,00	1,00
57,00	1,00	1,00
56,00	1,00	1,00
52,00	1,00	1,00
50,00	1,00	1,00
82,00	1,00	1,00
44,00	1,00	1,00
68,00	1,00	1,00
58,00	1,00	1,00
54,00	1,00	1,00
56,00	1,00	1,00
14,00	1,00	1,00
39,00	1,00	1,00
44,00	1,00	1,00
62,00	1,00	1,00
26,00	1,00	1,00
74,00	1,00	1,00
53,00	1,00	1,00
45,00	1,00	1,00
28,00	1,00	1,00
75,00	1,00	1,00
66,00	1,00	1,00
43,00	1,00	1,00
68,00	1,00	1,00
90,00	1,00	1,00
64,00	1,00	1,00
61,00	1,00	1,00
25,00	1,00	1,00
70,00	1,00	1,00

75,00	1,00	1,00
62,00	1,00	1,00
75,00	1,00	1,00
51,00	1,00	1,00
62,00	1,00	1,00
61,00	1,00	1,00
56,00	1,00	1,00
49,00	1,00	1,00
70,00	1,00	1,00
79,00	1,00	1,00
33,00	1,00	1,00
73,00	1,00	1,00
69,00	1,00	1,00
55,00	1,00	1,00
58,00	1,00	1,00
70,00	1,00	1,00
76,00	1,00	1,00
50,00	1,00	1,00
71,00	1,00	1,00
58,00	1,00	1,00
52,00	1,00	1,00
59,00	1,00	1,00
69,00	1,00	1,00
57,00	1,00	1,00
36,00	1,00	1,00
53,00	1,00	1,00
46,00	1,00	1,00
34,00	1,00	1,00
74,00	1,00	1,00
59,00	1,00	1,00
50,00	1,00	1,00
38,00	1,00	1,00
61,00	1,00	1,00
60,00	1,00	1,00
67,00	1,00	1,00
60,00	1,00	1,00
67,00	1,00	1,00
65,00	1,00	1,00
59,00	1,00	1,00
68,00	1,00	1,00
68,00	1,00	1,00
66,00	1,00	1,00
47,00	1,00	1,00
37,00	1,00	1,00
80,00	1,00	1,00
62,00	1,00	1,00
40,00	1,00	1,00
67,00	1,00	1,00
45,00	1,00	1,00
49,00	1,00	1,00

68,00	1,00	1,00
53,00	1,00	1,00
69,00	1,00	1,00
53,00	1,00	1,00
47,00	1,00	1,00
42,00	1,00	1,00
33,00	1,00	1,00
49,00	1,00	1,00
67,00	1,00	1,00
21,00	1,00	1,00
69,00	1,00	1,00
38,00	1,00	1,00
17,00	1,00	1,00
74,00	1,00	1,00
51,00	1,00	1,00
56,00	1,00	1,00
80,00	1,00	1,00
70,00	1,00	1,00
48,00	1,00	1,00
50,00	1,00	1,00
68,00	1,00	1,00
29,00	1,00	1,00
16,00	1,00	1,00
53,00	1,00	1,00
69,00	1,00	1,00
38,00	1,00	1,00
68,00	1,00	1,00
63,00	1,00	1,00
57,00	1,00	1,00
61,00	1,00	1,00
61,00	1,00	1,00
62,00	1,00	1,00
72,00	1,00	1,00
18,00	1,00	1,00
49,00	1,00	1,00
64,00	1,00	1,00
68,00	1,00	1,00
33,00	1,00	1,00
33,00	1,00	1,00
65,00	1,00	1,00
50,00	1,00	1,00
45,00	1,00	1,00
41,00	1,00	1,00
49,00	1,00	1,00
62,00	1,00	1,00
38,00	1,00	1,00
37,00	1,00	1,00
64,00	1,00	1,00
49,00	1,00	1,00
26,00	1,00	1,00

55,00	1,00	1,00
24,00	2,00	1,00
31,00	2,00	1,00
21,00	2,00	1,00
10,00	2,00	1,00
42,00	2,00	1,00
18,00	2,00	1,00
32,00	2,00	1,00
20,00	2,00	1,00
11,00	2,00	1,00
10,00	2,00	1,00
17,00	2,00	1,00
11,00	2,00	1,00
39,00	2,00	1,00
27,00	2,00	1,00
23,00	2,00	1,00
17,00	2,00	1,00
50,00	2,00	1,00
31,00	2,00	1,00
30,00	2,00	1,00
11,00	2,00	1,00
28,00	2,00	1,00
16,00	2,00	1,00
21,00	2,00	1,00
31,00	2,00	1,00
24,00	2,00	1,00
14,00	2,00	1,00
23,00	2,00	1,00
10,00	2,00	1,00
22,00	2,00	1,00
19,00	2,00	1,00
24,00	2,00	1,00
29,00	2,00	1,00
20,00	2,00	1,00
21,00	2,00	1,00
27,00	2,00	1,00
51,00	2,00	1,00
21,00	2,00	1,00
30,00	2,00	1,00
14,00	2,00	1,00
28,00	2,00	1,00
43,00	2,00	1,00
11,00	2,00	1,00
14,00	2,00	1,00
18,00	2,00	1,00
10,00	2,00	1,00
13,00	2,00	1,00
28,00	2,00	1,00
22,00	2,00	1,00
47,00	2,00	1,00

18,00	2,00	1,00
43,00	2,00	1,00
19,00	2,00	1,00
37,00	2,00	1,00
10,00	2,00	1,00
20,00	2,00	1,00
26,00	2,00	1,00
13,00	2,00	1,00
47,00	2,00	1,00
21,00	2,00	1,00
50,00	2,00	1,00
11,00	2,00	1,00
18,00	2,00	1,00
18,00	2,00	1,00
37,00	2,00	1,00
15,00	2,00	1,00
32,00	2,00	1,00
16,00	2,00	1,00
28,00	2,00	1,00
18,00	2,00	1,00
17,00	2,00	1,00
10,00	2,00	1,00
10,00	2,00	1,00
22,00	2,00	1,00
50,00	2,00	1,00
10,00	2,00	1,00
12,00	2,00	1,00
18,00	2,00	1,00
36,00	2,00	1,00
39,00	2,00	1,00
17,00	2,00	1,00
28,00	2,00	1,00
36,00	2,00	1,00
30,00	2,00	1,00
32,00	2,00	1,00
27,00	2,00	1,00
10,00	2,00	1,00
20,00	2,00	1,00
10,00	2,00	1,00
10,00	2,00	1,00
15,00	2,00	1,00
23,00	2,00	1,00
17,00	2,00	1,00
47,00	2,00	1,00
30,00	2,00	1,00
59,00	2,00	1,00
27,00	2,00	1,00
32,00	2,00	1,00
41,00	2,00	1,00
32,00	2,00	1,00

17,00	2,00	1,00
16,00	2,00	1,00
18,00	2,00	1,00
35,00	2,00	1,00
10,00	2,00	1,00
15,00	2,00	1,00
16,00	2,00	1,00
24,00	2,00	1,00
14,00	2,00	1,00
32,00	2,00	1,00
45,00	2,00	1,00
32,00	2,00	1,00
19,00	2,00	1,00
27,00	2,00	1,00
16,00	2,00	1,00
27,00	2,00	1,00
22,00	2,00	1,00
33,00	2,00	1,00
10,00	2,00	1,00
22,00	2,00	1,00
19,00	2,00	1,00
16,00	2,00	1,00
24,00	2,00	1,00
14,00	2,00	1,00
16,00	2,00	1,00
14,00	2,00	1,00
28,00	2,00	1,00
22,00	2,00	1,00
31,00	2,00	1,00
35,00	2,00	1,00
11,00	2,00	1,00
29,00	2,00	1,00
10,00	2,00	1,00
28,00	2,00	1,00
17,00	2,00	1,00
20,00	2,00	1,00
15,00	2,00	1,00
23,00	2,00	1,00
19,00	2,00	1,00
10,00	2,00	1,00
18,00	2,00	1,00
13,00	2,00	1,00
28,00	2,00	1,00
21,00	2,00	1,00
46,00	2,00	1,00
10,00	2,00	1,00
16,00	2,00	1,00
48,00	2,00	1,00
16,00	2,00	1,00
25,00	2,00	1,00

17,00	2,00	1,00
10,00	2,00	1,00
10,00	2,00	1,00
31,00	2,00	1,00
21,00	2,00	1,00
13,00	2,00	1,00
17,00	2,00	1,00
16,00	2,00	1,00
12,00	2,00	1,00
28,00	2,00	1,00
28,00	2,00	1,00
28,00	2,00	1,00
24,00	2,00	1,00
22,00	2,00	1,00
15,00	2,00	1,00
40,00	2,00	1,00
19,00	2,00	1,00
21,00	2,00	1,00
47,00	2,00	1,00
10,00	2,00	1,00
12,00	2,00	1,00
24,00	2,00	1,00
17,00	2,00	1,00
11,00	2,00	1,00
32,00	2,00	1,00
22,00	2,00	1,00
16,00	2,00	1,00
33,00	2,00	1,00
16,00	2,00	1,00
49,00	2,00	1,00
12,00	2,00	1,00
15,00	2,00	1,00
30,00	2,00	1,00
34,00	2,00	1,00
12,00	2,00	1,00
39,00	2,00	1,00
26,00	2,00	1,00
18,00	2,00	1,00
25,00	2,00	1,00
41,00	2,00	1,00
21,00	2,00	1,00
12,00	2,00	1,00
20,00	2,00	1,00
11,00	2,00	1,00
13,00	2,00	1,00
20,00	2,00	1,00
45,00	2,00	1,00
39,00	2,00	1,00
25,00	2,00	1,00
24,00	2,00	1,00

62,00	2,00	1,00
25,00	2,00	1,00
30,00	2,00	1,00
36,00	2,00	1,00
41,00	2,00	1,00
15,00	2,00	1,00
39,00	2,00	1,00
28,00	2,00	1,00
40,00	2,00	1,00
11,00	2,00	1,00
38,00	2,00	1,00
28,00	2,00	1,00
26,00	2,00	1,00
26,00	2,00	1,00
13,00	2,00	1,00
30,00	2,00	1,00
11,00	2,00	1,00
10,00	2,00	1,00
10,00	2,00	1,00
40,00	2,00	1,00
16,00	2,00	1,00
16,00	2,00	1,00
62,00	2,00	1,00
25,00	2,00	1,00
27,00	2,00	1,00
31,00	2,00	1,00
47,00	2,00	1,00
22,00	2,00	1,00
21,00	2,00	1,00
42,00	2,00	1,00
26,00	2,00	1,00
22,00	2,00	1,00
14,00	2,00	1,00
17,00	2,00	1,00
18,00	2,00	1,00
20,00	2,00	1,00
23,00	2,00	1,00
16,00	2,00	1,00
37,00	2,00	1,00
22,00	2,00	1,00
38,00	2,00	1,00
20,00	2,00	1,00
26,00	2,00	1,00
52,00	2,00	1,00
29,00	2,00	1,00
21,00	2,00	1,00
17,00	2,00	1,00
18,00	2,00	1,00
15,00	2,00	1,00
31,00	2,00	1,00

27,00	2,00	1,00
16,00	2,00	1,00
61,00	2,00	1,00
10,00	2,00	1,00
15,00	2,00	1,00
17,00	2,00	1,00
10,00	2,00	1,00
38,00	2,00	1,00
31,00	2,00	1,00
28,00	2,00	1,00
12,00	2,00	1,00
13,00	2,00	1,00
42,00	2,00	1,00
20,00	2,00	1,00
30,00	2,00	1,00
18,00	2,00	1,00
34,00	2,00	1,00
20,00	2,00	1,00
40,00	2,00	1,00
10,00	2,00	1,00
21,00	2,00	1,00
10,00	2,00	1,00
30,00	2,00	1,00
25,00	2,00	1,00
11,00	2,00	1,00
18,00	2,00	1,00
28,00	2,00	1,00
14,00	2,00	1,00
29,00	2,00	1,00
16,00	2,00	1,00
40,00	2,00	1,00
31,00	2,00	1,00
27,00	2,00	1,00
17,00	2,00	1,00
51,00	2,00	1,00
33,00	2,00	1,00
22,00	2,00	1,00
44,00	2,00	1,00
19,00	2,00	1,00
21,00	2,00	1,00
15,00	2,00	1,00
46,00	2,00	1,00
20,00	2,00	1,00
15,00	2,00	1,00
16,00	2,00	1,00
39,00	2,00	1,00
16,00	2,00	1,00
20,00	2,00	1,00
46,00	2,00	1,00
23,00	2,00	1,00

20,00	2,00	1,00
12,00	2,00	1,00
12,00	2,00	1,00
30,00	2,00	1,00
41,00	2,00	1,00
25,00	2,00	1,00
21,00	2,00	1,00
10,00	2,00	1,00
16,00	2,00	1,00
19,00	2,00	1,00
37,00	2,00	1,00
14,00	2,00	1,00
35,00	2,00	1,00
42,00	2,00	1,00
30,00	2,00	1,00
10,00	2,00	1,00
38,00	2,00	1,00
22,00	2,00	1,00
33,00	2,00	1,00
21,00	2,00	1,00
15,00	2,00	1,00
30,00	2,00	1,00
14,00	2,00	1,00
21,00	2,00	1,00
41,00	2,00	1,00
21,00	2,00	1,00
42,00	2,00	1,00
10,00	2,00	1,00
10,00	2,00	1,00
10,00	2,00	1,00
13,00	2,00	1,00
56,00	2,00	1,00
28,00	2,00	1,00
14,00	2,00	1,00
26,00	2,00	1,00
14,00	2,00	1,00
27,00	2,00	1,00
18,00	2,00	1,00
65,00	2,00	1,00
27,00	2,00	1,00
15,00	2,00	1,00
14,00	2,00	1,00
14,00	2,00	1,00
28,00	2,00	1,00
18,00	2,00	1,00
28,00	2,00	1,00
27,00	2,00	1,00
48,00	2,00	1,00
47,00	2,00	1,00
23,00	2,00	1,00

10,00	2,00	1,00
44,00	2,00	1,00
31,00	2,00	1,00
23,00	2,00	1,00
34,00	2,00	1,00
13,00	2,00	1,00
19,00	2,00	1,00
11,00	2,00	1,00
25,00	2,00	1,00
13,00	2,00	1,00
27,00	2,00	1,00
14,00	2,00	1,00
23,00	2,00	1,00
31,00	2,00	1,00
22,00	2,00	1,00
18,00	2,00	1,00
15,00	2,00	1,00
10,00	2,00	1,00
45,00	2,00	1,00
32,00	2,00	1,00
31,00	2,00	1,00
22,00	2,00	1,00
32,00	2,00	1,00
20,00	2,00	1,00
27,00	2,00	1,00
17,00	2,00	1,00
17,00	2,00	1,00
22,00	2,00	1,00
28,00	2,00	1,00
11,00	2,00	1,00
28,00	2,00	1,00
26,00	2,00	1,00
10,00	2,00	1,00
23,00	2,00	1,00
38,00	2,00	1,00
15,00	2,00	1,00
25,00	2,00	1,00
16,00	2,00	1,00
17,00	2,00	1,00
23,00	2,00	1,00
16,00	2,00	1,00
13,00	2,00	1,00
25,00	2,00	1,00
20,00	2,00	1,00
37,00	2,00	1,00
23,00	2,00	1,00
21,00	2,00	1,00
36,00	2,00	1,00
11,00	2,00	1,00
23,00	2,00	1,00

29,00	2,00	1,00
51,00	2,00	1,00
16,00	2,00	1,00
20,00	2,00	1,00
14,00	2,00	1,00
11,00	2,00	1,00
38,00	2,00	1,00
44,00	2,00	1,00
12,00	2,00	1,00
23,00	2,00	1,00
20,00	2,00	1,00
46,00	2,00	1,00
29,00	2,00	1,00
16,00	2,00	1,00
12,00	2,00	1,00
24,00	2,00	1,00
30,00	2,00	1,00
19,00	2,00	1,00
21,00	2,00	1,00
10,00	2,00	1,00
18,00	2,00	1,00
16,00	2,00	1,00
12,00	2,00	1,00
22,00	2,00	1,00
32,00	2,00	1,00
12,00	2,00	1,00
12,00	2,00	1,00
22,00	2,00	1,00
19,00	2,00	1,00
18,00	2,00	1,00
23,00	2,00	1,00
16,00	2,00	1,00
14,00	2,00	1,00
45,00	2,00	1,00
23,00	2,00	1,00
60,00	2,00	1,00
22,00	2,00	1,00
32,00	2,00	1,00
26,00	2,00	1,00
20,00	2,00	1,00
10,00	2,00	1,00
22,00	2,00	1,00
27,00	2,00	1,00
47,00	2,00	1,00
17,00	2,00	1,00
11,00	2,00	1,00
25,00	2,00	1,00
31,00	2,00	1,00
19,00	2,00	1,00
31,00	2,00	1,00

14,00	2,00	1,00
29,00	2,00	1,00
69,00	2,00	1,00
41,00	2,00	1,00
20,00	2,00	1,00
21,00	2,00	1,00
46,00	2,00	1,00
18,00	2,00	1,00
31,00	2,00	1,00
15,00	2,00	1,00
19,00	2,00	1,00
80,00	2,00	1,00
25,00	2,00	1,00
32,00	2,00	1,00
15,00	2,00	1,00
17,00	2,00	1,00
17,00	2,00	1,00
32,00	2,00	1,00
31,00	2,00	1,00
19,00	2,00	1,00
14,00	2,00	1,00
30,00	2,00	1,00
41,00	2,00	1,00
11,00	2,00	1,00
17,00	2,00	1,00
23,00	2,00	1,00
17,00	2,00	1,00
28,00	2,00	1,00
20,00	2,00	1,00
15,00	2,00	1,00
23,00	2,00	1,00
27,00	2,00	1,00
24,00	2,00	1,00
19,00	2,00	1,00
31,00	2,00	1,00
10,00	2,00	1,00
21,00	2,00	1,00
18,00	2,00	1,00
49,00	2,00	1,00
54,00	2,00	1,00
25,00	2,00	1,00
28,00	2,00	1,00
28,00	2,00	1,00
10,00	2,00	1,00
35,00	2,00	1,00
21,00	2,00	1,00
19,00	2,00	1,00
63,00	2,00	1,00
15,00	2,00	1,00
12,00	2,00	1,00

33,00	2,00	1,00
13,00	2,00	1,00
14,00	2,00	1,00
18,00	2,00	1,00
34,00	2,00	1,00
14,00	2,00	1,00
22,00	2,00	1,00
38,00	2,00	1,00
22,00	2,00	1,00
20,00	2,00	1,00
28,00	2,00	1,00
24,00	2,00	1,00
15,00	2,00	1,00
14,00	2,00	1,00
43,00	2,00	1,00
17,00	2,00	1,00
18,00	2,00	1,00
28,00	2,00	1,00
16,00	2,00	1,00
30,00	2,00	1,00
11,00	2,00	1,00
31,00	2,00	1,00
12,00	2,00	1,00
33,00	2,00	1,00
26,00	2,00	1,00
20,00	2,00	1,00
26,00	2,00	1,00
19,00	2,00	1,00
26,00	2,00	1,00
21,00	2,00	1,00
10,00	2,00	1,00
24,00	2,00	1,00
39,00	2,00	1,00
14,00	2,00	1,00
25,00	2,00	1,00
19,00	2,00	1,00
16,00	2,00	1,00
19,00	2,00	1,00
31,00	2,00	1,00
15,00	2,00	1,00
25,00	2,00	1,00
29,00	2,00	1,00
19,00	2,00	1,00
10,00	2,00	1,00
10,00	2,00	1,00
17,00	2,00	1,00
28,00	2,00	1,00
18,00	2,00	1,00
32,00	2,00	1,00
24,00	2,00	1,00

35,00	2,00	1,00
17,00	3,00	1,00
44,00	3,00	1,00
23,00	3,00	1,00
20,00	3,00	1,00
39,00	3,00	1,00
25,00	3,00	1,00
19,00	3,00	1,00
24,00	3,00	1,00
19,00	3,00	1,00
13,00	3,00	1,00
15,00	3,00	1,00
14,00	3,00	1,00
53,00	3,00	1,00
45,00	3,00	1,00
15,00	3,00	1,00
15,00	3,00	1,00
43,00	3,00	1,00
32,00	3,00	1,00
38,00	3,00	1,00
10,00	3,00	1,00
19,00	3,00	1,00
17,00	3,00	1,00
29,00	3,00	1,00
49,00	3,00	1,00
40,00	3,00	1,00
15,00	3,00	1,00
20,00	3,00	1,00
10,00	3,00	1,00
22,00	3,00	1,00
22,00	3,00	1,00
37,00	3,00	1,00
27,00	3,00	1,00
31,00	3,00	1,00
31,00	3,00	1,00
50,00	3,00	1,00
30,00	3,00	1,00
16,00	3,00	1,00
32,00	3,00	1,00
14,00	3,00	1,00
18,00	3,00	1,00
33,00	3,00	1,00
11,00	3,00	1,00
10,00	3,00	1,00
18,00	3,00	1,00
12,00	3,00	1,00
19,00	3,00	1,00
30,00	3,00	1,00
21,00	3,00	1,00
56,00	3,00	1,00

28,00	3,00	1,00
32,00	3,00	1,00
17,00	3,00	1,00
66,00	3,00	1,00
54,00	3,00	1,00
22,00	3,00	1,00
24,00	3,00	1,00
19,00	3,00	1,00
22,00	3,00	1,00
45,00	3,00	1,00
47,00	3,00	1,00
17,00	3,00	1,00
19,00	3,00	1,00
14,00	3,00	1,00
52,00	3,00	1,00
18,00	3,00	1,00
11,00	3,00	1,00
31,00	3,00	1,00
24,00	3,00	1,00
15,00	3,00	1,00
11,00	3,00	1,00
10,00	3,00	1,00
10,00	3,00	1,00
21,00	3,00	1,00
47,00	3,00	1,00
10,00	3,00	1,00
30,00	3,00	1,00
37,00	3,00	1,00
11,00	3,00	1,00
32,00	3,00	1,00
12,00	3,00	1,00
25,00	3,00	1,00
19,00	3,00	1,00
19,00	3,00	1,00
26,00	3,00	1,00
13,00	3,00	1,00
10,00	3,00	1,00
21,00	3,00	1,00
10,00	3,00	1,00
11,00	3,00	1,00
23,00	3,00	1,00
16,00	3,00	1,00
21,00	3,00	1,00
16,00	3,00	1,00
42,00	3,00	1,00
65,00	3,00	1,00
43,00	3,00	1,00
26,00	3,00	1,00
59,00	3,00	1,00
40,00	3,00	1,00

13,00	3,00	1,00
22,00	3,00	1,00
17,00	3,00	1,00
31,00	3,00	1,00
10,00	3,00	1,00
15,00	3,00	1,00
18,00	3,00	1,00
18,00	3,00	1,00
24,00	3,00	1,00
25,00	3,00	1,00
32,00	3,00	1,00
20,00	3,00	1,00
16,00	3,00	1,00
20,00	3,00	1,00
19,00	3,00	1,00
21,00	3,00	1,00
31,00	3,00	1,00
28,00	3,00	1,00
10,00	3,00	1,00
20,00	3,00	1,00
23,00	3,00	1,00
14,00	3,00	1,00
35,00	3,00	1,00
17,00	3,00	1,00
28,00	3,00	1,00
34,00	3,00	1,00
13,00	3,00	1,00
12,00	3,00	1,00
17,00	3,00	1,00
39,00	3,00	1,00
10,00	3,00	1,00
19,00	3,00	1,00
10,00	3,00	1,00
26,00	3,00	1,00
12,00	3,00	1,00
52,00	3,00	1,00
11,00	3,00	1,00
30,00	3,00	1,00
15,00	3,00	1,00
22,00	3,00	1,00
30,00	3,00	1,00
15,00	3,00	1,00
49,00	3,00	1,00
40,00	3,00	1,00
69,00	3,00	1,00
10,00	3,00	1,00
11,00	3,00	1,00
50,00	3,00	1,00
13,00	3,00	1,00
35,00	3,00	1,00

19,00	3,00	1,00
19,00	3,00	1,00
12,00	3,00	1,00
48,00	3,00	1,00
24,00	3,00	1,00
12,00	3,00	1,00
11,00	3,00	1,00
12,00	3,00	1,00
11,00	3,00	1,00
11,00	3,00	1,00
36,00	3,00	1,00
53,00	3,00	1,00
19,00	3,00	1,00
20,00	3,00	1,00
10,00	3,00	1,00
56,00	3,00	1,00
18,00	3,00	1,00
25,00	3,00	1,00
27,00	3,00	1,00
52,00	3,00	1,00
22,00	3,00	1,00
45,00	3,00	1,00
38,00	3,00	1,00
14,00	3,00	1,00
54,00	3,00	1,00
14,00	3,00	1,00
23,00	3,00	1,00
38,00	3,00	1,00
35,00	3,00	1,00
50,00	3,00	1,00
13,00	3,00	1,00
37,00	3,00	1,00
29,00	3,00	1,00
25,00	3,00	1,00
24,00	3,00	1,00
42,00	3,00	1,00
28,00	3,00	1,00
21,00	3,00	1,00
27,00	3,00	1,00
20,00	3,00	1,00
23,00	3,00	1,00
15,00	3,00	1,00
57,00	3,00	1,00
12,00	3,00	1,00
25,00	3,00	1,00
21,00	3,00	1,00
28,00	3,00	1,00
53,00	3,00	1,00
17,00	3,00	1,00
38,00	3,00	1,00

81,00	3,00	1,00
32,00	3,00	1,00
23,00	3,00	1,00
34,00	3,00	1,00
36,00	3,00	1,00
38,00	3,00	1,00
39,00	3,00	1,00
29,00	3,00	1,00
71,00	3,00	1,00
14,00	3,00	1,00
39,00	3,00	1,00
29,00	3,00	1,00
35,00	3,00	1,00
42,00	3,00	1,00
14,00	3,00	1,00
39,00	3,00	1,00
11,00	3,00	1,00
18,00	3,00	1,00
11,00	3,00	1,00
38,00	3,00	1,00
11,00	3,00	1,00
33,00	3,00	1,00
57,00	3,00	1,00
14,00	3,00	1,00
28,00	3,00	1,00
31,00	3,00	1,00
34,00	3,00	1,00
43,00	3,00	1,00
15,00	3,00	1,00
43,00	3,00	1,00
30,00	3,00	1,00
20,00	3,00	1,00
11,00	3,00	1,00
40,00	3,00	1,00
24,00	3,00	1,00
16,00	3,00	1,00
17,00	3,00	1,00
27,00	3,00	1,00
30,00	3,00	1,00
50,00	3,00	1,00
42,00	3,00	1,00
12,00	3,00	1,00
19,00	3,00	1,00
19,00	3,00	1,00
27,00	3,00	1,00
21,00	3,00	1,00
26,00	3,00	1,00
10,00	3,00	1,00
13,00	3,00	1,00
17,00	3,00	1,00

20,00	3,00	1,00
22,00	3,00	1,00
56,00	3,00	1,00
10,00	3,00	1,00
11,00	3,00	1,00
15,00	3,00	1,00
11,00	3,00	1,00
48,00	3,00	1,00
29,00	3,00	1,00
25,00	3,00	1,00
16,00	3,00	1,00
21,00	3,00	1,00
45,00	3,00	1,00
27,00	3,00	1,00
52,00	3,00	1,00
23,00	3,00	1,00
17,00	3,00	1,00
26,00	3,00	1,00
23,00	3,00	1,00
14,00	3,00	1,00
10,00	3,00	1,00
56,00	3,00	1,00
45,00	3,00	1,00
10,00	3,00	1,00
15,00	3,00	1,00
25,00	3,00	1,00
40,00	3,00	1,00
45,00	3,00	1,00
15,00	3,00	1,00
35,00	3,00	1,00
58,00	3,00	1,00
52,00	3,00	1,00
53,00	3,00	1,00
22,00	3,00	1,00
54,00	3,00	1,00
43,00	3,00	1,00
25,00	3,00	1,00
42,00	3,00	1,00
21,00	3,00	1,00
17,00	3,00	1,00
34,00	3,00	1,00
47,00	3,00	1,00
52,00	3,00	1,00
16,00	3,00	1,00
16,00	3,00	1,00
33,00	3,00	1,00
40,00	3,00	1,00
26,00	3,00	1,00
57,00	3,00	1,00
31,00	3,00	1,00

17,00	3,00	1,00
16,00	3,00	1,00
44,00	3,00	1,00
38,00	3,00	1,00
30,00	3,00	1,00
29,00	3,00	1,00
16,00	3,00	1,00
18,00	3,00	1,00
25,00	3,00	1,00
33,00	3,00	1,00
25,00	3,00	1,00
12,00	3,00	1,00
50,00	3,00	1,00
42,00	3,00	1,00
50,00	3,00	1,00
10,00	3,00	1,00
49,00	3,00	1,00
17,00	3,00	1,00
47,00	3,00	1,00
27,00	3,00	1,00
12,00	3,00	1,00
18,00	3,00	1,00
15,00	3,00	1,00
21,00	3,00	1,00
51,00	3,00	1,00
23,00	3,00	1,00
44,00	3,00	1,00
17,00	3,00	1,00
12,00	3,00	1,00
10,00	3,00	1,00
13,00	3,00	1,00
34,00	3,00	1,00
19,00	3,00	1,00
13,00	3,00	1,00
36,00	3,00	1,00
11,00	3,00	1,00
26,00	3,00	1,00
24,00	3,00	1,00
63,00	3,00	1,00
41,00	3,00	1,00
12,00	3,00	1,00
25,00	3,00	1,00
24,00	3,00	1,00
21,00	3,00	1,00
19,00	3,00	1,00
35,00	3,00	1,00
39,00	3,00	1,00
47,00	3,00	1,00
43,00	3,00	1,00
40,00	3,00	1,00

14,00	3,00	1,00
35,00	3,00	1,00
12,00	3,00	1,00
55,00	3,00	1,00
49,00	3,00	1,00
14,00	3,00	1,00
33,00	3,00	1,00
11,00	3,00	1,00
23,00	3,00	1,00
11,00	3,00	1,00
14,00	3,00	1,00
18,00	3,00	1,00
22,00	3,00	1,00
18,00	3,00	1,00
32,00	3,00	1,00
25,00	3,00	1,00
29,00	3,00	1,00
20,00	3,00	1,00
40,00	3,00	1,00
54,00	3,00	1,00
45,00	3,00	1,00
46,00	3,00	1,00
45,00	3,00	1,00
29,00	3,00	1,00
20,00	3,00	1,00
20,00	3,00	1,00
14,00	3,00	1,00
34,00	3,00	1,00
22,00	3,00	1,00
17,00	3,00	1,00
12,00	3,00	1,00
59,00	3,00	1,00
12,00	3,00	1,00
14,00	3,00	1,00
70,00	3,00	1,00
17,00	3,00	1,00
17,00	3,00	1,00
11,00	3,00	1,00
19,00	3,00	1,00
23,00	3,00	1,00
12,00	3,00	1,00
21,00	3,00	1,00
26,00	3,00	1,00
13,00	3,00	1,00
21,00	3,00	1,00
43,00	3,00	1,00
23,00	3,00	1,00
41,00	3,00	1,00
32,00	3,00	1,00
15,00	3,00	1,00

29,00	3,00	1,00
46,00	3,00	1,00
24,00	3,00	1,00
37,00	3,00	1,00
29,00	3,00	1,00
11,00	3,00	1,00
38,00	3,00	1,00
42,00	3,00	1,00
43,00	3,00	1,00
28,00	3,00	1,00
22,00	3,00	1,00
10,00	3,00	1,00
31,00	3,00	1,00
41,00	3,00	1,00
12,00	3,00	1,00
12,00	3,00	1,00
31,00	3,00	1,00
35,00	3,00	1,00
25,00	3,00	1,00
14,00	3,00	1,00
30,00	3,00	1,00
17,00	3,00	1,00
13,00	3,00	1,00
17,00	3,00	1,00
50,00	3,00	1,00
11,00	3,00	1,00
11,00	3,00	1,00
10,00	3,00	1,00
23,00	3,00	1,00
14,00	3,00	1,00
13,00	3,00	1,00
10,00	3,00	1,00
10,00	3,00	1,00
57,00	3,00	1,00
13,00	3,00	1,00
26,00	3,00	1,00
18,00	3,00	1,00
50,00	3,00	1,00
26,00	3,00	1,00
14,00	3,00	1,00
12,00	3,00	1,00
16,00	3,00	1,00
10,00	3,00	1,00
14,00	3,00	1,00
18,00	3,00	1,00
12,00	3,00	1,00
18,00	3,00	1,00
42,00	3,00	1,00
13,00	3,00	1,00
19,00	3,00	1,00

10,00	3,00	1,00
59,00	3,00	1,00
76,00	3,00	1,00
21,00	3,00	1,00
14,00	3,00	1,00
34,00	3,00	1,00
46,00	3,00	1,00
22,00	3,00	1,00
33,00	3,00	1,00
10,00	3,00	1,00
24,00	3,00	1,00
73,00	3,00	1,00
50,00	3,00	1,00
40,00	3,00	1,00
17,00	3,00	1,00
10,00	3,00	1,00
40,00	3,00	1,00
38,00	3,00	1,00
42,00	3,00	1,00
46,00	3,00	1,00
19,00	3,00	1,00
42,00	3,00	1,00
33,00	3,00	1,00
18,00	3,00	1,00
43,00	3,00	1,00
27,00	3,00	1,00
17,00	3,00	1,00
28,00	3,00	1,00
39,00	3,00	1,00
14,00	3,00	1,00
25,00	3,00	1,00
37,00	3,00	1,00
13,00	3,00	1,00
41,00	3,00	1,00
35,00	3,00	1,00
10,00	3,00	1,00
12,00	3,00	1,00
24,00	3,00	1,00
28,00	3,00	1,00
60,00	3,00	1,00
22,00	3,00	1,00
29,00	3,00	1,00
33,00	3,00	1,00
25,00	3,00	1,00
22,00	3,00	1,00
44,00	3,00	1,00
28,00	3,00	1,00
40,00	3,00	1,00
13,00	3,00	1,00
17,00	3,00	1,00

15,00	3,00	1,00
12,00	3,00	1,00
12,00	3,00	1,00
17,00	3,00	1,00
37,00	3,00	1,00
45,00	3,00	1,00
21,00	3,00	1,00
37,00	3,00	1,00
24,00	3,00	1,00
18,00	3,00	1,00
29,00	3,00	1,00
25,00	3,00	1,00
13,00	3,00	1,00
54,00	3,00	1,00
49,00	3,00	1,00
19,00	3,00	1,00
65,00	3,00	1,00
52,00	3,00	1,00
18,00	3,00	1,00
28,00	3,00	1,00
17,00	3,00	1,00
54,00	3,00	1,00
16,00	3,00	1,00
41,00	3,00	1,00
19,00	3,00	1,00
21,00	3,00	1,00
19,00	3,00	1,00
16,00	3,00	1,00
30,00	3,00	1,00
28,00	3,00	1,00
13,00	3,00	1,00
25,00	3,00	1,00
52,00	3,00	1,00
18,00	3,00	1,00
28,00	3,00	1,00
40,00	3,00	1,00
11,00	3,00	1,00
21,00	3,00	1,00
45,00	3,00	1,00
36,00	3,00	1,00
26,00	3,00	1,00
27,00	3,00	1,00
18,00	3,00	1,00
41,00	3,00	1,00
32,00	3,00	1,00
14,00	3,00	1,00
20,00	3,00	1,00
23,00	3,00	1,00
25,00	3,00	1,00
34,00	3,00	1,00

11,00	3,00	1,00
23,00	1,00	2,00
17,00	1,00	2,00
45,00	1,00	2,00
74,00	1,00	2,00
52,00	1,00	2,00
12,00	1,00	2,00
53,00	1,00	2,00
60,00	1,00	2,00
10,00	1,00	2,00
45,00	1,00	2,00
63,00	1,00	2,00
87,00	1,00	2,00
72,00	1,00	2,00
19,00	1,00	2,00
42,00	1,00	2,00
88,00	1,00	2,00
47,00	1,00	2,00
68,00	1,00	2,00
20,00	1,00	2,00
38,00	1,00	2,00
22,00	1,00	2,00
60,00	1,00	2,00
57,00	1,00	2,00
35,00	1,00	2,00
61,00	1,00	2,00
59,00	1,00	2,00
80,00	1,00	2,00
40,00	1,00	2,00
54,00	1,00	2,00
64,00	1,00	2,00
20,00	1,00	2,00
58,00	1,00	2,00
45,00	1,00	2,00
36,00	1,00	2,00
46,00	1,00	2,00
77,00	1,00	2,00
29,00	1,00	2,00
54,00	1,00	2,00
22,00	1,00	2,00
68,00	1,00	2,00
62,00	1,00	2,00
22,00	1,00	2,00
64,00	1,00	2,00
22,00	1,00	2,00
23,00	1,00	2,00
29,00	1,00	2,00
34,00	1,00	2,00
60,00	1,00	2,00
70,00	1,00	2,00

33,00	1,00	2,00
35,00	1,00	2,00
14,00	1,00	2,00
56,00	1,00	2,00
61,00	1,00	2,00
50,00	1,00	2,00
19,00	1,00	2,00
53,00	1,00	2,00
59,00	1,00	2,00
59,00	1,00	2,00
71,00	1,00	2,00
55,00	1,00	2,00
52,00	1,00	2,00
20,00	1,00	2,00
36,00	1,00	2,00
77,00	1,00	2,00
31,00	1,00	2,00
32,00	1,00	2,00
43,00	1,00	2,00
84,00	1,00	2,00
76,00	1,00	2,00
59,00	1,00	2,00
48,00	1,00	2,00
25,00	1,00	2,00
56,00	1,00	2,00
49,00	1,00	2,00
33,00	1,00	2,00
38,00	1,00	2,00
30,00	1,00	2,00
53,00	1,00	2,00
10,00	1,00	2,00
18,00	1,00	2,00
28,00	1,00	2,00
70,00	1,00	2,00
38,00	1,00	2,00
39,00	1,00	2,00
42,00	1,00	2,00
33,00	1,00	2,00
50,00	1,00	2,00
66,00	1,00	2,00
46,00	1,00	2,00
67,00	1,00	2,00
70,00	1,00	2,00
48,00	1,00	2,00
73,00	1,00	2,00
33,00	1,00	2,00
52,00	1,00	2,00
67,00	1,00	2,00
11,00	1,00	2,00
34,00	1,00	2,00

61,00	1,00	2,00
41,00	1,00	2,00
48,00	1,00	2,00
29,00	1,00	2,00
29,00	1,00	2,00
45,00	1,00	2,00
59,00	1,00	2,00
18,00	1,00	2,00
45,00	1,00	2,00
66,00	1,00	2,00
70,00	1,00	2,00
50,00	1,00	2,00
35,00	1,00	2,00
40,00	1,00	2,00
37,00	1,00	2,00
90,00	1,00	2,00
67,00	1,00	2,00
61,00	1,00	2,00
27,00	1,00	2,00
42,00	1,00	2,00
19,00	1,00	2,00
24,00	1,00	2,00
51,00	1,00	2,00
39,00	1,00	2,00
52,00	1,00	2,00
11,00	1,00	2,00
55,00	1,00	2,00
20,00	1,00	2,00
61,00	1,00	2,00
55,00	1,00	2,00
19,00	1,00	2,00
68,00	1,00	2,00
62,00	1,00	2,00
57,00	1,00	2,00
63,00	1,00	2,00
34,00	1,00	2,00
46,00	1,00	2,00
66,00	1,00	2,00
48,00	1,00	2,00
51,00	1,00	2,00
54,00	1,00	2,00
67,00	1,00	2,00
45,00	1,00	2,00
26,00	1,00	2,00
42,00	1,00	2,00
54,00	1,00	2,00
72,00	1,00	2,00
18,00	1,00	2,00
70,00	1,00	2,00
55,00	1,00	2,00

45,00	1,00	2,00
59,00	1,00	2,00
43,00	1,00	2,00
50,00	1,00	2,00
63,00	1,00	2,00
61,00	1,00	2,00
36,00	1,00	2,00
41,00	1,00	2,00
36,00	1,00	2,00
54,00	1,00	2,00
57,00	1,00	2,00
46,00	1,00	2,00
43,00	1,00	2,00
51,00	1,00	2,00
37,00	1,00	2,00
50,00	1,00	2,00
36,00	1,00	2,00
70,00	1,00	2,00
36,00	1,00	2,00
70,00	1,00	2,00
48,00	1,00	2,00
53,00	1,00	2,00
43,00	1,00	2,00
46,00	1,00	2,00
70,00	1,00	2,00
10,00	1,00	2,00
51,00	1,00	2,00
23,00	1,00	2,00
66,00	1,00	2,00
10,00	1,00	2,00
48,00	1,00	2,00
13,00	2,00	2,00
13,00	2,00	2,00
13,00	2,00	2,00
22,00	2,00	2,00
12,00	2,00	2,00
12,00	2,00	2,00
45,00	2,00	2,00
17,00	2,00	2,00
10,00	2,00	2,00
31,00	2,00	2,00
17,00	2,00	2,00
11,00	2,00	2,00
36,00	2,00	2,00
13,00	2,00	2,00
23,00	2,00	2,00
10,00	2,00	2,00
42,00	2,00	2,00
28,00	2,00	2,00
15,00	2,00	2,00

13,00	2,00	2,00
18,00	2,00	2,00
12,00	2,00	2,00
54,00	2,00	2,00
20,00	2,00	2,00
16,00	2,00	2,00
26,00	2,00	2,00
41,00	2,00	2,00
22,00	2,00	2,00
28,00	2,00	2,00
20,00	2,00	2,00
12,00	2,00	2,00
17,00	2,00	2,00
61,00	2,00	2,00
27,00	2,00	2,00
42,00	2,00	2,00
27,00	2,00	2,00
25,00	2,00	2,00
12,00	2,00	2,00
10,00	2,00	2,00
26,00	2,00	2,00
30,00	2,00	2,00
20,00	2,00	2,00
31,00	2,00	2,00
17,00	2,00	2,00
13,00	2,00	2,00
19,00	2,00	2,00
36,00	2,00	2,00
31,00	2,00	2,00
12,00	2,00	2,00
30,00	2,00	2,00
35,00	2,00	2,00
16,00	2,00	2,00
19,00	2,00	2,00
16,00	2,00	2,00
10,00	2,00	2,00
14,00	2,00	2,00
14,00	2,00	2,00
12,00	2,00	2,00
33,00	2,00	2,00
20,00	2,00	2,00
46,00	2,00	2,00
16,00	2,00	2,00
13,00	2,00	2,00
38,00	2,00	2,00
32,00	2,00	2,00
33,00	2,00	2,00
41,00	2,00	2,00
13,00	2,00	2,00
13,00	2,00	2,00

58,00	2,00	2,00
55,00	2,00	2,00
31,00	2,00	2,00
18,00	2,00	2,00
35,00	2,00	2,00
50,00	2,00	2,00
15,00	2,00	2,00
54,00	2,00	2,00
26,00	2,00	2,00
12,00	2,00	2,00
14,00	2,00	2,00
15,00	2,00	2,00
14,00	2,00	2,00
13,00	2,00	2,00
36,00	2,00	2,00
31,00	2,00	2,00
40,00	2,00	2,00
16,00	2,00	2,00
10,00	2,00	2,00
36,00	2,00	2,00
58,00	2,00	2,00
26,00	2,00	2,00
22,00	2,00	2,00
21,00	2,00	2,00
69,00	2,00	2,00
28,00	2,00	2,00
14,00	2,00	2,00
21,00	2,00	2,00
20,00	2,00	2,00
37,00	2,00	2,00
63,00	2,00	2,00
17,00	2,00	2,00
21,00	2,00	2,00
13,00	2,00	2,00
19,00	2,00	2,00
16,00	2,00	2,00
32,00	2,00	2,00
34,00	2,00	2,00
34,00	2,00	2,00
20,00	2,00	2,00
18,00	2,00	2,00
19,00	2,00	2,00
24,00	2,00	2,00
14,00	2,00	2,00
29,00	2,00	2,00
13,00	2,00	2,00
16,00	2,00	2,00
20,00	2,00	2,00
27,00	2,00	2,00
22,00	2,00	2,00

23,00	2,00	2,00
23,00	2,00	2,00
24,00	2,00	2,00
25,00	2,00	2,00
24,00	2,00	2,00
10,00	2,00	2,00
18,00	2,00	2,00
12,00	2,00	2,00
15,00	2,00	2,00
25,00	2,00	2,00
16,00	2,00	2,00
36,00	2,00	2,00
26,00	2,00	2,00
53,00	2,00	2,00
12,00	2,00	2,00
39,00	2,00	2,00
23,00	2,00	2,00
26,00	2,00	2,00
43,00	2,00	2,00
54,00	2,00	2,00
26,00	2,00	2,00
31,00	2,00	2,00
19,00	2,00	2,00
20,00	2,00	2,00
26,00	2,00	2,00
44,00	2,00	2,00
30,00	2,00	2,00
14,00	2,00	2,00
16,00	2,00	2,00
42,00	2,00	2,00
18,00	2,00	2,00
27,00	2,00	2,00
23,00	2,00	2,00
10,00	2,00	2,00
23,00	2,00	2,00
22,00	2,00	2,00
24,00	2,00	2,00
13,00	2,00	2,00
20,00	2,00	2,00
20,00	2,00	2,00
15,00	2,00	2,00
28,00	2,00	2,00
10,00	2,00	2,00
15,00	2,00	2,00
23,00	2,00	2,00
27,00	2,00	2,00
24,00	2,00	2,00
23,00	2,00	2,00
20,00	2,00	2,00
59,00	2,00	2,00

39,00	2,00	2,00
30,00	2,00	2,00
10,00	2,00	2,00
33,00	2,00	2,00
32,00	2,00	2,00
10,00	2,00	2,00
10,00	2,00	2,00
10,00	2,00	2,00
18,00	2,00	2,00
10,00	2,00	2,00
47,00	2,00	2,00
11,00	3,00	2,00
12,00	3,00	2,00
15,00	3,00	2,00
35,00	3,00	2,00
23,00	3,00	2,00
24,00	3,00	2,00
35,00	3,00	2,00
11,00	3,00	2,00
31,00	3,00	2,00
25,00	3,00	2,00
15,00	3,00	2,00
13,00	3,00	2,00
51,00	3,00	2,00
20,00	3,00	2,00
11,00	3,00	2,00
41,00	3,00	2,00
60,00	3,00	2,00
34,00	3,00	2,00
19,00	3,00	2,00
19,00	3,00	2,00
18,00	3,00	2,00
10,00	3,00	2,00
46,00	3,00	2,00
24,00	3,00	2,00
32,00	3,00	2,00
31,00	3,00	2,00
32,00	3,00	2,00
18,00	3,00	2,00
22,00	3,00	2,00
21,00	3,00	2,00
15,00	3,00	2,00
20,00	3,00	2,00
10,00	3,00	2,00
20,00	3,00	2,00
12,00	3,00	2,00
62,00	3,00	2,00
37,00	3,00	2,00
22,00	3,00	2,00
45,00	3,00	2,00

36,00	3,00	2,00
23,00	3,00	2,00
28,00	3,00	2,00
40,00	3,00	2,00
34,00	3,00	2,00
47,00	3,00	2,00
23,00	3,00	2,00
47,00	3,00	2,00
22,00	3,00	2,00
23,00	3,00	2,00
28,00	3,00	2,00
37,00	3,00	2,00
19,00	3,00	2,00
24,00	3,00	2,00
10,00	3,00	2,00
24,00	3,00	2,00
49,00	3,00	2,00
15,00	3,00	2,00
13,00	3,00	2,00
34,00	3,00	2,00
24,00	3,00	2,00
33,00	3,00	2,00
14,00	3,00	2,00
19,00	3,00	2,00
30,00	3,00	2,00
22,00	3,00	2,00
27,00	3,00	2,00
38,00	3,00	2,00
13,00	3,00	2,00
11,00	3,00	2,00
66,00	3,00	2,00
34,00	3,00	2,00
19,00	3,00	2,00
27,00	3,00	2,00
30,00	3,00	2,00
14,00	3,00	2,00
17,00	3,00	2,00
54,00	3,00	2,00
27,00	3,00	2,00
22,00	3,00	2,00
10,00	3,00	2,00
22,00	3,00	2,00
14,00	3,00	2,00
33,00	3,00	2,00
59,00	3,00	2,00
29,00	3,00	2,00
46,00	3,00	2,00
29,00	3,00	2,00
18,00	3,00	2,00
13,00	3,00	2,00

38,00	3,00	2,00
34,00	3,00	2,00
10,00	3,00	2,00
16,00	3,00	2,00
55,00	3,00	2,00
12,00	3,00	2,00
17,00	3,00	2,00
29,00	3,00	2,00
10,00	3,00	2,00
20,00	3,00	2,00
55,00	3,00	2,00
17,00	3,00	2,00
12,00	3,00	2,00
29,00	3,00	2,00
18,00	3,00	2,00
10,00	3,00	2,00
38,00	3,00	2,00
34,00	3,00	2,00
24,00	3,00	2,00
24,00	3,00	2,00
13,00	3,00	2,00
30,00	3,00	2,00
11,00	3,00	2,00
14,00	3,00	2,00
16,00	3,00	2,00
20,00	3,00	2,00
42,00	3,00	2,00
17,00	3,00	2,00
14,00	3,00	2,00
18,00	3,00	2,00
13,00	3,00	2,00
20,00	3,00	2,00
36,00	3,00	2,00
12,00	3,00	2,00
33,00	3,00	2,00
19,00	3,00	2,00
19,00	3,00	2,00
10,00	3,00	2,00
12,00	3,00	2,00
27,00	3,00	2,00
14,00	3,00	2,00
33,00	3,00	2,00
30,00	3,00	2,00
47,00	3,00	2,00
26,00	3,00	2,00
23,00	3,00	2,00
20,00	3,00	2,00
16,00	3,00	2,00
20,00	3,00	2,00
18,00	3,00	2,00

21,00	3,00	2,00
20,00	3,00	2,00
42,00	3,00	2,00
15,00	3,00	2,00
42,00	3,00	2,00
47,00	3,00	2,00
26,00	3,00	2,00
15,00	3,00	2,00
17,00	3,00	2,00
40,00	3,00	2,00
24,00	3,00	2,00
13,00	3,00	2,00
29,00	3,00	2,00
11,00	3,00	2,00
14,00	3,00	2,00
26,00	3,00	2,00
28,00	3,00	2,00
14,00	3,00	2,00
27,00	3,00	2,00
35,00	3,00	2,00
25,00	3,00	2,00
42,00	3,00	2,00
55,00	3,00	2,00
12,00	3,00	2,00
38,00	3,00	2,00
48,00	3,00	2,00
10,00	3,00	2,00
20,00	3,00	2,00
17,00	3,00	2,00
50,00	3,00	2,00
62,00	3,00	2,00
36,00	3,00	2,00
39,00	3,00	2,00
28,00	3,00	2,00
15,00	3,00	2,00
18,00	3,00	2,00
11,00	3,00	2,00
14,00	3,00	2,00
32,00	3,00	2,00
18,00	3,00	2,00
33,00	3,00	2,00
