# Supplementary material for: Applying Corrigan’s progressive model of self-stigma to people with depression
Source: PLoS One. 2019 Oct 29;14(10):e0224418. doi: 10.1371/journal.pone.0224418 (PMC6818799; doi:10.1371/journal.pone.0224418)
Supplement: S3 File — (PDF) [file pone.0224418.s003.pdf]

```
* Encoding: UTF-8.

/* PROCESS version 3.0 */.
/* Written by Andrew F. Hayes */.
/* www.afhayes.com */.
/* www.processmacro.org */.
/* Copyright 2018 by Andrew F. Hayes */.
/* Documented in http://www.guilford.com/p/hayes3 */.
/* PROCESS workshop schedule at http://www.processmacro.org/workshops.html */.

/* Distribution of this code in any form, except through processmacro.org, is prohibited */.
/* without the permission of the copyright holder */.

/* THIS SOFTWARE IS PROVIDED "AS IS", WITHOUT WARRANTY OF ANY KIND */.
/* EXPRESS OR IMPLIED, INCLUDING BUT NOT LIMITED TO THE WARRANTIES OF */.
/* MERCHANTABILITY, FITNESS FOR A PARTICULAR PURPOSE AND NONINFRINGEMENT */.
/* IN NO EVENT SHALL THE COPYRIGHT HOLDERS BE LIABLE FOR ANY CLAIM, */.
/* DAMAGES OR OTHER LIABILITY, WHETHER IN AN ACTION OF CONTRACT, TORT */.
/* OR OTHERWISE, ARISING FROM, OUT OF OR IN CONNECTION WITH THE */.
/* SOFTWARE OR THE USE OR OTHER DEALINGS IN THE SOFTWARE */.
/* USE OF THIS SOFTWARE IMPLIES AGREEMENT WITH THESE TERMS */.

set printback=off.

define bcboot3 (databcbt = !charend ('/')/estmte = !charend ('/') !default(9999)).
compute temp = !databcbt.
compute temp(GRADE(!databcbt)) = !databcbt.
compute badlo = 0.
```

```
compute badhi = 0.
do if (!estmte <> 9999).
  compute pv=csum(temp < !estmte)/nrow(temp).
  compute ppv = pv.
  do if (pv > .5).
    compute ppv = 1-pv.
  end if.
  compute y5=sqrt(-2*ln(ppv)).
  compute xp=y5+((((y5*p4+p3)*y5+p2)*y5+p1)*y5+p0)/((((y5*q4+q3)*y5+q2)*y5+q1)*y5+q0).
  do if (pv <= .5).
    compute xp = -xp.
  end if.
  compute cilow=rnd(nrow(temp)*(cdfnorm(2*xp+xp2))).
  compute cihigh=trunc(nrow(temp)*(cdfnorm(2*xp+(-xp2))))+1.
  do if (cilow < 1).
    compute cilow = 1.
     compute booterr=1.
     compute badlo = 1.
  end if.
  do if (cihigh > nrow(temp)).
    compute cihigh = boot.
    compute booterr=1.
     compute badhi = 1.
  end if.
  compute llcit=temp(cilow,1).
  compute ulcit=temp(cihigh,1).
end if.
do if (!estmte = 9999).
```

```
   compute llcit=temp(cilow,1).
   compute ulcit=temp(cihigh,1).
end if.
compute bootse=t(sqrt((cssq(temp)-((csum(temp)&**2)/nrow(temp)))/(nrow(temp)-1))).
!enddefine.

DEFINE describe (descdatf=!charend('/')/type=!charend('/') !default(0)).
compute desctmp=make((8-(4*!type)),ncol(!descdatf),-999).
/* mean, sd, min, max, 16th, 50th, 84th, dich toggle */.
loop jd=1 to ncol(!descdatf).
  compute descdat=!descdatf(:,jd).
  /* get the mean, sd, minimum, and maximum */.
  compute desctmp(1,jd) = csum(descdat)/nrow(descdat).
  compute desctmp(2,jd) = (nrow(descdat)*sscp(descdat))-(t(csum(descdat))*(csum(descdat))).
  compute desctmp(2,jd) = sqrt(desctmp(2,jd)/(nrow(descdat)*(nrow(descdat)-1))).
  compute desctmp(3,jd)=cmin(descdat).
  compute desctmp(4,jd)=cmax(descdat).
  do if (!type=0).
     compute minwarn=0.
     compute maxwarn=0.
    /* check to see if the variable is a constant */.
    do if ((desctmp(3,jd)=desctmp(4,jd)) and novar=0).
      compute errcode(errs,1)=15.
      compute errs=errs+1.
      compute criterr=1.
      compute novar=1.
    end if.
    /* check and set the dichotomous toggle */.
```

```
compute tmp=((descdat(:,1)=desctmp(3,jd))+(descdat(:,1)=desctmp(4,jd))).

compute desctmp(8,jd)=(csum(tmp)=nrow(tmp)).

/* here we calculate the percentiles of the distribution */.

compute tmp = descdat.

compute tmp(GRADE(descdat),:) = descdat.

compute descdat = tmp.

release tmp.

compute decval={.16;.5;.84}.

loop kd=1 to 3.

  compute low=trunc(decval(kd,1)*(nrow(descdat)+1)).

  compute lowdec=decval(kd,1)*(nrow(descdat)+1)-low.

  compute value=descdat(low,1)+(descdat((low+1),1)-descdat(low,1))*lowdec.

  compute desctmp((4+kd),jd)=value.

end loop.

compute mnotev=1.

compute modvals=desctmp(5:7,:).

/* change to mean and plus/minus one SD if needed */.

do if (quantile <> 1).

  compute desctmp(5,jd)=desctmp(1,jd)-desctmp(2,jd).

  compute desctmp(6,jd)=desctmp(1,jd).

  compute desctmp(7,jd)=desctmp(1,jd)+desctmp(2,jd).

  compute modvals=desctmp(5:7,:).

  compute mnotev=2.

  do if (modvals(1,1) < desctmp(3,1)).

    compute modvals(1,1)=desctmp(3,1).

    compute minwarn=1.

  end if.

  do if (modvals(3,1) > desctmp(4,1)).
```

```
      compute modvals(3,1)=desctmp(4,1).
      compute maxwarn=1.
    end if.
  end if.
  do if (desctmp(8,1)=1).
    compute modvals={desctmp(3,1);desctmp(4,1)}.
    compute mnotev=0.
    compute minwarn=0.
    compute maxwarn=0.
  end if.
 end if.
end loop.
!ENDDEFINE.


DEFINE ftest3 (lm=!charend('/')/bcoef=!charend('/')/cv=!charend('/') !default(0)/chr=!charend('/')
!default(0)/
 brsq=!charend('/') !default(0)/skip=!charend('/') !default(0)).
compute lmat2=!lm.
do if (!skip=0).
 compute lmat2 = mdiag(!lm).
 compute lmat3=make(nrow(lmat2),1,0).
 loop flp=1 to ncol(lmat2).
  do if (csum(lmat2(:,flp))=1).
    compute lmat3={lmat3,lmat2(:,flp)}.
  end if.
 end loop.
 compute lmat2=lmat3(:,2:ncol(lmat3)).
end if.
```

```
compute fratio = (t(t(lmat2)*!bcoef)*inv(t(lmat2)*!cv*lmat2)*((t(lmat2)*!bcoef)))/ncol(lmat2).
compute pfr = 1-fcdf(fratio,ncol(lmat2),(n-nrow(!bcoef))).
compute fresult={fratio,ncol(lmat2),(n-nrow(!bcoef)),pfr}.
do if (!chr=1).
  compute lmat3=1-rsum(lmat2).
  compute xfm=make(n,csum(lmat3),0).
  compute flpc=1.
  loop flp=1 to nrow(lmat3).
    do if (lmat3(flp,1)=1).
      compute xfm(:,flpc)=x(:,flp).
      compute flpc=flpc+1.
    end if.
  end loop.
  compute bfm=inv(t(xfm)*xfm)*t(xfm)*y.
  compute resid=y-(xfm*bfm).
  compute sstotal=(y-(csum(y)/n)).
  compute sstotal=csum(sstotal&*sstotal).
  compute ssresid=csum(resid&*resid).
  compute rsqch=!brsq-((sstotal-ssresid)/sstotal).
  compute fresult={rsqch,fresult}.
  release xfm,flpc, resid, ssresid, bfm.
end if.
!ENDDEFINE.

DEFINE hcest3 (x=!charend('/')/resid=!charend('/')/hc=!charend('/')/mse=!charend('/')).
  compute n1=nrow(!x).
  compute invXtX = inv(t(!x)*!x).
  compute varb = !mse*invXtX.
```

```
compute k3 = ncol(!x).
compute xhc=0.
do if (!hc <> 5).
  compute xhc=!x.
  compute hat = xhc(:,1).
  loop i3=1 to nrow(xhc).
    compute hat(i3,1)= xhc(i3,:)*invXtX*t(xhc(i3,:)).
  end loop.
  do if (!hc = 0 or !hc =1).
    loop i3 = 1 to k3.
      compute xhc(:,i3)=xhc(:,i3)&*!resid.
    end  loop.
  end if.
  do if (!hc =3 or !hc=2).
    loop i3=1 to k3.
      compute xhc(:,i3) = (!resid&/(1-hat)&**(1/(4-!hc)))&*xhc(:,i3).
    end loop.
  end if.
  do if (!hc = 4).
    compute hcmn=make(n,2,4).
    compute hcmn(:,2)=(n1*hat)/k3.
    loop i3= 1 to k3.
      compute xhc(:,i3) = (!resid&/(1-hat)&**(rmin(hcmn)/2))&*xhc(:,i3).
    end loop.
  end if.
  compute varb=(invXtX*t(xhc)*xhc*invXtX).
  do if (!hc=1).
    compute varb=(n1/(n1-ncol(!x)))&*varb.
```

```
    end if.
  end if.
  compute hclab={"se(HC0)","se(HC1)","se(HC2)","se(HC3)","se(HC4)","se"}.
  compute hclab=hclab(1,(!hc+1)).
  compute hcflab={"F(HC0)","F(HC1)","F(HC2)","F(HC3)","F(HC4)","F"}.
  compute hcflab=hcflab(1,(!hc+1)).
  release xhc.
!ENDDEFINE.

DEFINE longchk (variab=!charend('/')).
/* check for any long variable names */.
  !let !toomany=0.
  !do !i !in (!variab).
    !do !j = 1 !to !length(!i).
      !if ((!j > 8) !and (!toomany = 0)) !then.
        !let !toomany = 1.
        compute toomany=1.
      !ifend.
    !doend.
  !doend.
!ENDDEFINE.

DEFINE drindef (decpnt=!charend('/') !default(F10.4)).
do if (criterr = 0 and nms > 0).
  compute paths=paths(:,2:ncol(paths)).
  compute pathsw=pathsw(:,2:ncol(pathsw)).
  compute pathsz=pathsz(:,2:ncol(pathsz)).
  compute pathswz=pathswz(:,2:ncol(pathswz)).
```

```
  compute pathsmod=pathsw+pathsz+pathswz.

  compute pathsdv=pathsdv(:,2:ncol(pathsdv)).

  compute pathsfoc=pathsfoc(:,2:ncol(pathsfoc)).

  compute pathtype=pathtype(:,2:ncol(pathtype)).

  compute anymod=(rsum(pathsmod) > 0).

  compute obscoeff=obscoeff(1,2:ncol(obscoeff)).

  do if (dototal = 0).

    print/title = "****************** DIRECT AND INDIRECT EFFECTS OF X ON Y
*****************".

  end if.

  do if (dototal = 1).

    print/title = "************** TOTAL, DIRECT, AND INDIRECT EFFECTS OF X ON Y
**************".

    compute toteffl b(1,1)="Effect".

    do if (counterf <>  1).

      do if (effsize=1).

        compute toteffsz=toteff(:,1)/ysd.

        compute totefflb={totefflb,"c_ps"}.

        do if (xdich = 0 and mcx = 0).

          compute toteffsz={toteffsz, (toteffsz*xsd)}.

          compute totefflb={totefflb,"c_cs"}.

        end if.

        compute toteff={toteff,toteffsz}.

      end if.

      do if (nxvls > 1).

        print toteff/title="Relative total effects of X on
Y:"/rnames=toteffl2/cnames=totefflb/format=!decpnt.

        compute clabtmp={"R2-chng", hcflab, "df1","df2","p"}.

        print totomni/title="Omnibus test of total effect of X on Y:"/cnames=clabtmp/format=!decpnt.
```

```
      print/title= "----------"/space=0.
    else.
      print toteff/title="Total effect of X on Y"/cnames=totefflb/format=!decpnt.
    end if.
  end if.
end if.
compute moddir=wcmat(nrow(bcmat),1)+zcmat(nrow(bcmat),1).
do if (bcmat(nrow(bcmat),1)=1 and counterf <> 1).
  do if (moddir=0).
    compute direfflb(1,1)="Effect".
  end if.
  do if (effsize=1 and moddir=0 and anymod = 0).
    compute direffsz=direff(:,1)/ysd.
    compute direfflb={direfflb,"c'_ps"}.
    do if (xdich = 0 and mcx = 0).
      compute direffsz={direffsz, (direffsz*xsd)}.
    compute direfflb={direfflb,"c'_cs"}.
    end if.
    compute direff={direff,direffsz}.
  end if.
  do if (moddir = 0 and nxvls=1).
    print direff/title="Direct effect of X on Y"/cnames=direfflb/format=!decpnt.
  end if.
  do if (moddir = 0 and nxvls>1).
    print direff/title="Relative direct effects of X on
Y"/rnames=direffl2/cnames=direfflb/format=!decpnt.
    compute clabtmp={"R2-chng", hcflab, "df1","df2","p"}.
    print diromni/title="Omnibus test of direct effect of X on Y:"/cnames=clabtmp/format=!decpnt.
```

```
      print/title= "----------"/space=0.
    end if.
    do if (moddir > 0 and nxvls=1).
      print direff/title="Conditional direct effect(s) of X on Y:"/cnames=diresfflb/format=!decpnt.
    end if.
    do if (moddir > 0 and nxvls>1).
      compute direffl2={" "}.
      loop i = 1 to nxvls.
        loop j = 1 to (nrow(direff)/nxvls).
          compute direffl2={direffl2;xcatlab(i,1)}.
        end loop.
      end loop.
      compute direffl2=direffl2(2:nrow(direffl2),1).
      print direff/title="Relative conditional direct effect(s) of X on
Y:"/cnames=direfflb/rnames=direffl2/format=!decpnt.
    end if.
  end if.
  do if (bcmat(nrow(bcmat),1)=0 and counterf <> 1).
      print/title="The direct effect of X on Y is fixed to zero.".
  end if.
  /* print/title= "----------"/space=0 */.


  /* Here is the start of the indirect effects */.


  do if (nms = 1).
    compute indmark={2}.
    compute indsets={1,2}.
  end if.
```

```
 do if (nms = 2).
 compute indmark={2,2,3}.
 compute indsets={1,4,2,5,1,3,5}.
   compute thetam=1.
 end if.
 do if (nms = 3).
   compute indmark={2,2,2,3,3,3,4}.
   compute indsets={1,7,2,8,4,9,1,3,8,1,5,9,2,6,9,1,3,6,9}.
   compute thetam={1,2,3}.
 end if.
 do if (nms = 4).
   compute indmark={2,2,2,2,3,3,3,3,3,3,4,4,4,4,5}.
   compute
indsets={1,11,2,12,4,13,7,14,1,3,12,1,5,13,1,8,14,2,6,13,2,9,14,4,10,14,1,3,6,13,1,3,9,14,1,5,10,14,2,
6,10,14,1,3,6,10,14}.
   compute thetam={1,2,4,3,5,6}.
 end if.
 do if (nms = 5).
   compute indmark={2,2,2,2,2,3,3,3,3,3,3,3,3,3,3,4,4,4,4,4,4,4,4,4,4,5,5,5,5,5,6}.
   compute
indsets={1,16,2,17,4,18,7,19,11,20,1,3,17,1,5,18,1,8,19,1,12,20,2,6,18,2,9,19,2,13,20,4,10,19,4,14,20
,7,15,20,1,3,6,18,

1,3,9,19,1,3,13,20,1,5,10,19,1,5,14,20,1,8,15,20,2,6,10,19,2,6,14,20,2,9,15,20,4,10,15,20,1,3,6,10,19,
                                     1,3,6,14,20,1,3,9,15,20,1,5,10,15,20,2,6,10,15,20,1,3,6,10,15,20}.
   compute thetam={1,2,5,3,6,8,4,7,9,10}.
 end if.
 do if (nms = 6).
   compute
indmark={2,2,2,2,2,2,3,3,3,3,3,3,3,3,3,3,3,3,3,3,3,4,4,4,4,4,4,4,4,4,4,4,4,4,4,4,4,4,4,4,4,5,5,5,5,5,5,5,5,
5,5,5,5,5,5,5,6,6,6,6,6,6,7}.
```

```
   compute
indsets={1,22,2,23,4,24,7,25,11,26,16,27,1,3,23,1,5,24,1,8,25,1,12,26,1,17,27,2,6,24,2,9,25,2,13,26,2
,18,27,4,10,25,4,14,26,

4,19,27,7,15,26,7,20,27,11,21,27,1,3,6,24,1,3,9,25,1,3,13,26,1,3,18,27,1,5,10,25,1,5,14,26,1,5,19,27,
1,8,15,26,1,8,20,27,

1,12,21,27,2,6,10,25,2,6,14,26,2,6,19,27,2,9,15,26,2,9,20,27,2,13,21,27,4,10,15,26,4,10,20,27,4,14,2
1,27,7,15,21,27,

1,3,6,10,25,1,3,6,14,26,1,3,6,19,27,1,3,9,15,26,1,3,9,20,27,1,3,13,21,27,1,5,10,15,26,1,5,10,20,27,1,5
,14,21,27,

1,8,15,21,27,2,6,10,15,26,2,6,10,20,27,2,6,14,21,27,2,9,15,21,27,4,10,15,21,27,1,3,6,10,15,26,1,3,6,1
0,20,27,

1,3,6,14,21,27,1,3,9,15,21,27,1,5,10,15,21,27,2,6,10,15,21,27,1,3,6,10,15,21,27}.
   compute thetam={1,2,6,3,7,10,4,8,11,13,5,9,12,14,15}.
  end if.
  do if (nms = 7).
   compute indmark={2,2,2,2,2,2,2}.
   compute indsets={1,29,2,30,4,31,7,32,11,33,16,34,22,35}.
  end if.
  do if (nms = 8).
   compute indmark={2,2,2,2,2,2,2,2}.
   compute indsets={1,37,2,38,4,39,7,40,11,41,16,42,22,43,29,44}.
  end if.
  do if (nms = 9).
   compute indmark={2,2,2,2,2,2,2,2,2}.
   compute indsets={1,46,2,47,4,48,7,49,11,50,16,51,22,52,29,53,37,54}.
  end if.
  do if (nms=10).
```

```
    compute indmark={2,2,2,2,2,2,2,2,2,2}.
    compute indsets={1,56,2,57,4,58,7,59,11,60,16,61,22,62,29,63,37,64,46,65}.
  end if.
  compute indlbl = {"Ind1"; "Ind2"; "Ind3"; "Ind4"; "Ind5"; "Ind6"; "Ind7"; "Ind8"; "Ind9"; "Ind10";
"Ind11"; "Ind12"; "Ind13"; "Ind14"; "Ind15"}.
  compute indlbl = {indlbl;
"Ind16";"Ind17";"Ind18";"Ind19";"Ind20";"Ind21";"Ind22";"Ind23";"Ind24";"Ind25";"Ind26";"Ind27";"
Ind28";"Ind29";"Ind30"}.
  compute indlbl = {indlbl;
"Ind31";"Ind32";"Ind33";"Ind34";"Ind35";"Ind36";"Ind37";"Ind38";"Ind39";"Ind40";"Ind41";"Ind42";"
Ind43";"Ind44";"Ind45"}.
  compute indlbl = {indlbl;
"Ind46";"Ind47";"Ind48";"Ind49";"Ind50";"Ind51";"Ind52";"Ind53";"Ind54";"Ind55";"Ind56";"Ind57";"
Ind58";"Ind59";"Ind60"}.
  compute indlbl = {indlbl; "Ind61";"Ind62";"Ind63"}.
  compute
cntname={"(C1)";"(C2)";"(C3)";"(C4)";"(C5)";"(C6)";"(C7)";"(C8)";"(C9)";"(C10)";"(C11)";"(C12)";"(C13)
";"(C14)";"(C15)";"(C16)";"(C17)"}.
  compute
cntname={cntname;"(C18)";"(C19)";"(C20)";"(C21)";"(C22)";"(C23)";"(C24)";"(C25)";"(C26)";"(C27)";"(
C28)";"(C29)";"(C30)";"(C31)"}.
  compute
cntname={cntname;"(C32)";"(C33)";"(C34)";"(C35)";"(C36)";"(C37)";"(C38)";"(C39)";"(C40)";"(C41)";"(
C42)";"(C43)";"(C44)";"(C45)"}.
  compute
cntname={cntname;"(C46)";"(C47)";"(C48)";"(C49)";"(C50)";"(C51)";"(C52)";"(C53)";"(C54)";"(C55)";"(
C56)";"(C57)";"(C58)";"(C59)"}.
  compute
cntname={cntname;"(C60)";"(C61)";"(C62)";"(C63)";"(C64)";"(C65)";"(C66)";"(C67)";"(C68)";"(C69)";"(
C70)";"(C71)";"(C72)";"(C73)"}.
  compute
cntname={cntname;"(C74)";"(C75)";"(C76)";"(C77)";"(C78)";"(C79)";"(C80)";"(C81)";"(C82)";"(C83)";"(
C84)";"(C85)";"(C86)";"(C87)"}.
  compute
cntname={cntname;"(C88)";"(C89)";"(C90)";"(C91)";"(C92)";"(C93)";"(C94)";"(C95)";"(C96)";"(C97)";"(
C98)";"(C99)";"(C100)";"(C101)"}.
```

```
compute cntname={cntname;"(C102)";"(C103)";"(C104)";"(C105)"}.
compute indmake=make(ncol(indmark),(nms+2),0).
compute indmod=make(ncol(indmark),1,999).
compute indmmm=make(ncol(indmark),1,0).
compute indmmmt=make(ncol(indmark),1,0).
compute start=1.
compute end=0.
compute nindfx=0.
compute indlocs=make(nrow(thetaxmb),ncol(paths),999).
compute indkey=make(ncol(indmark),1+((rmax(indmark)*2)+1),"     ").
compute c1=1.
compute c2=1.
compute c3=1.
loop i = 1 to ncol(paths).
  do if (pathtype(1,i)=1).
    compute indlocs(:,i)=thetaxmb(:,c1).
    compute c1=c1+1.
  end if.
  do if (pathtype(1,i)=3).
    compute indlocs(:,i)=thetamyb(:,c2).
    compute c2=c2+1.
  end if.
  do if (pathtype(1,i)=2 and nms < 7 and serial=1).
    compute indlocs(:,i)=thetammb(:,thetam(1,c3)).
    compute c3=c3+1.
  end if.
end loop.
/* print indlocs */.
```

```
loop i = 1 to ncol(indlocs).
  compute c1=2.
  loop j = 2 to nrow(indlocs).
    do if (indlocs(j,i) <> 0).
      compute indlocs(c1,i)=indlocs(j,i).
      compute c1=c1+1.
    end if.
  end loop.
  compute indlocs(1,i)=c1-2.
end loop.
compute indlocs=indlocs(1:rmax((indlocs(1,:))+1),:).
loop i = 1 to ncol(indmark).
  compute numget=indmark(1,i).
  compute end=end+numget.
  compute gotcha=indsets(1,start:end).
  compute start=end+1.
  compute ok=1.
  compute temp=0.
  compute repoman=make(4,1,0).
    loop j = 1 to ncol(gotcha).
      do if paths(1,gotcha(1,j))=0.
        compute ok=0.
      end if.
      do if (pathsmod(1,gotcha(1,j)) > 0).
        compute temp=1.
        compute temp2={pathsw(1,gotcha(1,j));pathsz(1,gotcha(1,j));pathswz(1,gotcha(1,j));0}.
        do if (temp2(1,1)=1 and temp2(2,1)=1 and temp2(3,1)=0).
          compute temp2(4,1)=1.
```

```
    end if.
    compute repoman=repoman+temp2.
  end if.
end loop.
compute temp=0.
compute tempmmm=0.
compute typemmm=0.
do if ((repoman(1,1) > 0) and (repoman(2,1) = 0)).
  compute temp=1.
  do if (repoman(1,1)=1).
    compute tempmmm=1.
  end if.
  do if (repoman(1,1) > 1 and (wdich=1 or mcw > 0)).
    compute tempmmm=12.
    compute typemmm=mcw.
    do if (wdich=1).
      compute typemmm=1.
    end if.
  end if.
  do if (repoman(1,1) > 1 and (wdich=0 and mcw = 0)).
    compute tempmmm=101.
  end if.
end if.
do if ((repoman(1,1) = 0) and (repoman(2,1) > 0)).
  compute temp=2.
  do if (repoman(2,1)=1).
    compute tempmmm=2.
  end if.
```

```
  do if (repoman(2,1) > 1 and (zdich = 1 or mcz > 0)).
    compute tempmmm=22.
    compute typemmm=mcz.
    do if (zdich=1).
      compute typemmm=1.
    end if.
  end if.
  do if (repoman(2,1) > 1 and (zdich = 0 and mcw = 0)).
    compute tempmmm=102.
  end if.
end if.
do if (repoman(1,1)>0 and repoman(2,1)>0).
  compute temp=3.
  do if (repoman(1,1)=1 and repoman(2,1)=1).
    do if (repoman(4,1)=1).
      compute tempmmm=31.
    end if.
    do if (repoman(3,1)=1).
      compute tempmmm=41.
    end if.
  end if.
end if.
do if (repoman(1,1)=1 and repoman(2,1)=1 and repoman(3,1)=0 and repoman(4,1)=0).
  compute tempmmm=51.
end if.
do if (ok = 1).
  compute nindfx=nindfx+1.
  compute indmake(nindfx,1)=numget.
```

```
      compute indmod(nindfx,1)=temp.
      compute indmmm(nindfx,1)=tempmmm.
      compute indmmmt(nindfx,1)=typemmm.
      compute indmake(nindfx,2:(1+numget))=gotcha.
      compute indkey(nindfx,1)=xnames.
       loop j = 1 to numget.
         compute indkey(nindfx,(j*2+1))=pathsdv(1,gotcha(1,j)).
         compute indkey(nindfx,(j*2))="   ->   ".
       end loop.
    end if.
end loop.
compute indkey=indkey(1:nindfx,1:((cmax(indmake(:,1))*2)+1)).
compute indmake=indmake(1:nindfx,1:(cmax(indmake(:,1))+1)).
compute indmod=indmod(1:nrow(indmake),1).
compute indmmm=indmmm(1:nrow(indmake),1).
compute indmmmt=indmmmt(1:nrow(indmake),1).
compute ncpairs = (((nindfx)*(nindfx-1))/2).
do if ((contrast = 1 or contrast = 2) and (ncpairs > 105)).
  compute contrast=0.
  compute notecode(notes,1) = 13.
  compute notes = notes + 1.
end if.
do if (contrast = 3).
  do if (ncol(contvec) <> nindfx).
    compute contrast=0.
    compute notecode(notes,1) = 14.
    compute notes = notes + 1.
  end if.
```

```
end if.

/* This is for models with no moderator */.
do if (anymod=0).
  do if (nms = 1 and contrast > 0).
    compute contrast=0.
  end if.
  compute efloop=(((1-(effsize=0))*2)+1)-(((mcx>0 or xdich=1))*(1-(effsize=0))).
  loop kk=1 to efloop.
    do if (boot = 0).
      compute bootres=obscoeff.
      compute indtab=999.
      compute inddiff=999.
      compute bootysd=ysd.
      compute bootxsd=xsd.
    end if.
    do if (boot > 0).
      compute bootres={obscoeff;bootres}.
      compute indtab=make(1,4,999).
      compute inddiff=make(nrow(bootres),1,999).
    end if.
    compute indtotal=make(nrow(bootres),1,0).
    loop i = 1 to nrow(indmake).
      loop j = 1 to nxvls.
        compute indtemp=make(nrow(bootres),1,1).
        loop k = 1 to indmake(i,1).
          compute jtemp=1.
          do if (j > 1 and k=1).
```

```
      compute jtemp=j.
    end if.
    compute indtemp=indtemp&*bootres(:,pathsfoc(jtemp,indmake(i,(k+1)))).
  end loop.
  do if (kk = 2).
    compute indtemp=indtemp/bootysd.
  end if.
  do if (kk = 3).
    compute indtemp=(bootxsd&*indtemp)/bootysd.
  end if.
  do if (contrast <> 0).
    compute inddiff={inddiff,indtemp}.
  end if.
  do if (nxvls=1).
    compute indtotal=indtotal+indtemp.
  end if.
  compute indeff=indtemp(1,1).
  do if (boot > 0).
    bcboot3 databcbt = indtemp(2:nrow(indtemp),1).
    compute indeff={indeff,bootse,llcit,ulcit}.
  end if.
  compute indtab={indtab;indeff}.
 end loop.
end loop.
compute indtab=indtab(2:nrow(indtab),:).
compute rowlbs=indlbl(1:nrow(indtab),1).
do if (mc > 0).
 compute inddiff=make(mc,1,-999).
```

```
compute indtab2=make(nrow(indtab),4,-999).
compute indtab2(:,1)=indtab.
compute indtab=indtab2.
release indtab2.
compute mcct=0.
compute indtotal=make(mc,1,0).
do if (kk = 1).
  compute x1 = sqrt(-
2*ln(uniform(mc,nrow(mcsopath))))&*cos((2*3.14159265358979)*uniform(mc,nrow(mcsopath))).
  compute x1=x1*chol(indcov).
  loop ii=1 to nrow(x1).
    compute x1(ii,:)=x1(ii,:)+t(mcsopath).
  end loop.
end if.
loop ii=1 to nms.
  compute tmpb=x1(:,((nms*nxvls)+ii)).
  compute tmpb2=tmpb.
  do if (nxvls > 1).
    loop jj=1 to (nxvls-1).
      compute tmpb2={tmpb2,tmpb}.
    end loop.
  end if.
  compute indtemp=x1(:,(((ii-1)*nxvls)+1):(ii*nxvls))&*tmpb2.
  loop jj=1 to ncol(indtemp).
    do if (kk = 2).
      compute indtemp(:,jj)=indtemp(:,jj)/ysd.
    end if.
    do if (kk = 3).
```

```
      compute indtemp(:,jj)=(xsd*indtemp(:,jj))/ysd.
    end if.
    bcboot3 databcbt = indtemp(:,jj).
    compute mcct=mcct+1.
    compute indtab(mcct,2:4)={bootse,llcit,ulcit}.
  end loop.
  do if (nxvls=1).
    compute indtotal=indtotal+indtemp.
    do if (contrast <> 0).
      compute inddiff={inddiff,indtemp}.
    end if.
  end if.
 end loop.
 release indtemp,tmpb.
end if.
do if (normal = 1 and sobelok=1).
 compute sobelmat=indtab(:,1).
 compute sobelmat={sobelmat,(sobelmat/2),sobelmat,sobelmat}.
 loop ii=1 to nms.
  compute se2b=indcov(((nms*nxvls)+ii),((nms*nxvls)+ii)).
  compute bpath2=mcsopath(((nms*nxvls)+ii),1)&**2.
  compute se2a=diag(indcov((((ii-1)*nxvls)+1):(ii*nxvls),(((ii-1)*nxvls)+1):(ii*nxvls))).
  compute apath2=mcsopath((((ii-1)*nxvls)+1):(ii*nxvls) ,1)&**2.
  compute sesobel=sqrt(apath2*se2b+bpath2*se2a+se2a*se2b).
  compute sobelmat((((ii-1)*nxvls)+1):(ii*nxvls),2)=sesobel.
 end loop.
 release se2b,bpath2,se2a,apath2,sesobel,ii.
 compute sobelmat(:,3)=sobelmat(:,1)&/sobelmat(:,2).
```

```
  compute sobelmat(:,4) = 2*(1-cdfnorm(abs(sobelmat(:,3)))).
end if.
do if (serial = 0).
  compute rowlbs=t(mnames).
end if.
do if (nxvls=1 and nms > 1).
  compute rowlbs={"TOTAL";rowlbs}.
  compute indtemp=indtotal(1,1).
  do if (boot > 0 and nxvls=1).
    bcboot3 databcbt = indtotal(2:nrow(indtotal),1).
    compute indtemp={indtemp, bootse,llcit,ulcit}.
  end if.
  do if (mc > 0).
    compute obtmc=indtab(:,1).
    compute indtemp=csum(obtmc).
    bcboot3 databcbt = indtotal(:,1).
    compute indtemp={indtemp, bootse,llcit,ulcit}.
  end if.
  compute indtab={indtemp;indtab}.
end if.
compute bootlbs={"Effect", "BootSE","BootLLCI","BootULCI"}.
do if (mc > 0).
  compute bootlbs={"Effect", "MC SE","MC LLCI","MC ULCI"}.
end if.
do if (nxvls = 1).
  do if (contrast <> 0).
    compute inddiff=inddiff(:,2:ncol(inddiff)).
    do if (mc > 0).
```

```
  compute inddiff={t(obtmc);inddiff}.
end if.
do if (contrast = 3).
  compute inddifft=inddiff*t(contvec).
  compute indtemp=inddifft(1,1).
  do if (boot > 0 or mc > 0).
    bcboot3 databcbt = inddifft(2:nrow(inddifft),1).
    compute indtemp={indtemp, bootse,llcit,ulcit}.
  end if.
  compute indtab={indtab;indtemp}.
end if.
do if (contrast = 1 or contrast = 2).
  compute conkey=make(1,4," ").
  loop i = 1 to ncol(inddiff)-1.
    loop j = (i+1) to ncol(inddiff).
      compute inddifft=inddiff(:,i)-inddiff(:,j).
      do if (contrast=2).
        compute inddifft=abs(inddiff(:,i))-abs(inddiff(:,j)).
      end if.
      compute indtemp=inddifft(1,1).
      compute conkeyt={" ", rowlbs((i+1),1)," minus  ",rowlbs((j+1),1)}.
      compute conkey={conkey;conkeyt}.
      do if (boot > 0 or mc > 0).
        bcboot3 databcbt = inddifft(2:nrow(inddifft),1).
        compute indtemp={indtemp, bootse,llcit,ulcit}.
      end if.
      compute indtab={indtab;indtemp}.
    end loop.
```

```
    end loop.
   end if.
   release inddiff.
   compute contlbs=cntname(1:(((nindfx)*(nindfx-1))/2),1).
   compute rowlbs={rowlbs;contlbs}.
  end if.
  do if (kk=1).
   print indtab/title = "Indirect effect(s) of X on Y:"/rnames=rowlbs/cnames=bootlbs/format=!decpnt.
  end if.
  do if (kk = 2).
   print indtab/title = "Partially standardized indirect effect(s) of X on Y:"/rnames=rowlbs/cnames=bootlbs/format=!decpnt.
  end if.
  do if (kk = 3).
   print indtab/title = "Completely standardized indirect effect(s) of X on Y:"/rnames=rowlbs/cnames=bootlbs/format=!decpnt.
  end if.
  do if (normal=1 and sobelok=1 and kk=1).
   compute sobellab={"Effect",hclab,"Z","p"}.
   compute sobelrlb=rowlbs.
   do if (nms > 1).
    compute sobelrlb=rowlbs(2:(1+nms),1).
   end if.
   print sobelmat/title="   Normal theory test for indirect effect(s):"/cnames=sobellab/rnames=sobelrlb/format=!decpnt.
  end if.
  do if (contrast <> 0).
   do if ((contrast=1 or contrast = 2) and kk=efloop ).
    compute conkey=conkey(2:nrow(conkey),:).
```

```
print conkey/title = "Specific indirect effect contrast
definition(s):"/rnames=contlbs/format=A8.
end if.
do if (contrast = 3 and kk=efloop).
compute crowlbs=rowlbs(2:(nindfx+1),1).
print contvec/title = "Specific indirect effect contrast
weights:"/cnames=crowlbs/rlabels="(C1)"/format=!decpnt.
end if.
do if (contrast = 2 and kk=efloop).
print/title = "Contrasts are differences between absolute values of indirect effects".
end if.
end if.
do if (serial = 1 and kk=efloop).
compute rowlbst=rowlbs(2:nrow(rowlbs),1).
print indkey/title = "Indirect effect key:"/rnames=rowlbst/format = A8.
end if.
else.
do if (kk = 1).
print/title = "Relative indirect effects of X on Y".
end if.
do if (kk = 2).
print/title = "Partially standardized relative indirect effect(s) of X on Y:".
end if.
do if (kk = 3).
print/title = "Completely standardized relative indirect effect(s) of X on Y:".
end if.
loop i = 1 to nrow(indmake).
compute indtabsm=indtab((((i-1)*nxvls)+1):(nxvls*i),:).
compute indkeyt=indkey(i,:).
```

```
        print indkeyt/title=" "/space=0/format=A8.
        print indtabsm/title = " "/cnames=bootlbs/rnames=direffl2/format=!decpnt/space=0.
        do if (normal=1 and sobelok=1 and kk=1).
          compute sobelsm=sobelmat((((i-1)*nxvls)+1):(nxvls*i),:).
          compute sobellab={"Effect",hclab,"Z","p"}.
          print sobelsm/title="      Normal theory test for relative indirect
effects:"/cnames=sobellab/rnames=direffl2/format=!decpnt.
        end if.
      end loop.
    end if.
    do if (effsize = 1 and boot > 0).
      compute bootres=bootres(2:nrow(bootres),:).
    end if.
  end loop.
 end if.
 /* this is the end of the no moderators loop */.


/* HERE IS WHERE WE WILL BE WORKING */.


 do if (anymod > 0).
  do if (boot = 0).
    compute bootres=obscoeff.
    compute indtab=999.
    /* compute inddiff=999 */.
  end if.
  do if (boot > 0).
    compute bootres={obscoeff;bootres}.
    compute indtab=make(1,4,999).
```

```
  /* compute inddiff=make(nrow(bootres),1,999) */.
end if.
do if (csum((indmod > 0))=nrow(indmod)).
  do if (nxvls > 1).
    print/title= "Relative conditional indirect effects of X on Y:".
  end if.
  do if (nxvls = 1).
    print/title= "Conditional indirect effects of X on Y:".
  end if.
end if.
do if (csum((indmod > 0)) < nrow(indmod)).
  do if (nxvls > 1).
    print/title= "Relative conditional and unconditional indirect effects of X on Y:".
  end if.
  do if (nxvls = 1).
    print/title= "Conditional and unconditional indirect effects of X on Y:".
  end if.
end if.

loop i = 1 to nrow(indmake).
  /* I added this but not sure */.
  compute indtab=indtab(1,:)*0.
  print indkey(i,:)/title = "INDIRECT EFFECT:"/format=A8.
  do if (indmod(i,1)=0).
    loop j = 1 to nxvls.
      compute indtemp=make(nrow(bootres),1,1).
      loop k = 1 to indmake(i,1).
        compute jtemp=1.
```

```
          do if (j > 1 and k=1).
            compute jtemp=j.
          end if.
          compute indtemp=indtemp&*bootres(:,pathsfoc(jtemp,indmake(i,(k+1)))).
        end loop.
        compute indeff=indtemp(1,1).
        do if (boot > 0).
          bcboot3 databcbt = indtemp(2:nrow(indtemp),1).
          compute indeff={indeff,bootse,llcit,ulcit}.
        end if.
        compute indtab={indtab;indeff}.
      end loop.
      do if (nxvls > 1).
        compute indefflb=xcatlab(1:nxvls,1).
        print indtab(2:nrow(indtab),:)/title = " "/clabels="Effect", "BootSE",  "BootLLCI",
"BootULCI"/rnames=indefflb/format=!decpnt/space=0.
      end if.
      do if (nxvls = 1).
        print indtab(2:nrow(indtab),:)/title = " "/clabels="Effect", "BootSE",  "BootLLCI",
"BootULCI"/format=!decpnt/space=0.
      end if.
    end if.
    /* end of unmoderated */.
    /* start of moderated */.
    do if (indmod(i,1)>0).
      do if (indmod(i,1)=1).
        compute indmodva=wmodvals.
        compute indprova=wprobval.
        compute condlbs={wnames}.
```

```
  compute printw=1.
 else if (indmod(i,1)=2).
  compute indmodva=zmodvals.
  compute indprova=zprobval.
  compute condlbs={znames}.
  compute printz=1.
 else if (indmod(i,1)=3).
  compute cntmp=1.
  compute printz=1.
  compute printw=1.
  compute indmodva=make((nrow(wmodvals)*nrow(zmodvals)),2,999).
  loop k7 = 1 to nrow(wmodvals).
   loop k8 = 1 to nrow(zmodvals).
    compute indmodva(cntmp,:)={wmodvals(k7,1),zmodvals(k8,1)}.
    compute cntmp=cntmp+1.
   end loop.
  end loop.
  compute condlbs={wnames,znames}.
 end if.
 compute condres=make(nrow(indmodva),1,999).
 do if (boot > 0).
  compute condres=make(nrow(indmodva),4,999).
 end if.
 compute condres={indmodva,condres}.
 /* Here is where the computations start */.
loop k4 = 1 to nxvls.
 compute imm3=make(nrow(bootres),1,1).
 compute imm4=make(nrow(bootres),1,1).
```

```
compute indcontr=0.
do if (indmod(i,1)=3).
  compute tihsw=wprobval.
  compute tihsz=zprobval.
end if.
loop k1=1 to nrow(indmodva).
  compute tucker2=make(nrow(bootres),1,1).
  compute imm2=make(nrow(bootres),1,1).
  compute wfirst=0.
  compute zfirst=0.
  compute immset=0.
  loop k2=1 to indmake(i,1).
    compute colnumb=indmake(i,(k2+1)).
    do if (k2=1).
       compute wbb=make(nrow(bootres),(nwvls*nxvls),0).
       compute zbb=make(nrow(bootres),(nzvls*nxvls),0).
       compute wzbb=make(nrow(bootres),(nwvls*nzvls*nxvls),0).
    end if.
    do if (k2<>1).
       compute wbb=make(nrow(bootres),nwvls,0).
       compute zbb=make(nrow(bootres),nzvls,0).
       compute wzbb=make(nrow(bootres),(nwvls*nzvls),0).
    end if.
    compute cnt=1.
    compute tihs=indlocs(2:((indlocs(1,colnumb))+1),colnumb).
    do if (k2 = 1).
      compute focbb=tihs(1:nxvls,1).
      compute focbb=bootres(:,focbb).
```

```
do if (indmmm(i,1)>0).
  compute imm=focbb(:,k4).
  compute condbb=imm.
end if.
compute focaddon=make(1,nxvls,0).
compute focaddon(1,k4)=1.
compute cnt=cnt+nxvls.
compute placeh=nxvls.
do if (indmod(i,1)=1).
  compute tihsz=make(nrow(wprobval),(nzvls*nxvls),0).
  compute tihswz=make(nrow(wprobval),(nwvls*nzvls*nxvls),0).
  do if (pathsw(1,colnumb)=1).
    compute temp=make(nrow(wprobval),(nxvls*nwvls),0).
    loop k5 = 1 to nrow(wprobval).
      loop k6=1 to nwvls.
        compute temp(k5, (((k4-1)*nwvls)+k6))=wprobval(k5,k6).
      end loop.
    end loop.
    compute indprova={temp,tihsz,tihswz}.
  else.
    compute indprova={wprobval,tihsz,tihswz}.
  end if.
end if.
do if (indmod(i,1)=2).
  compute tihsw=make(nrow(zprobval),(nwvls*nxvls),0).
  compute tihswz=make(nrow(zprobval),(nwvls*nzvls*nxvls),0).
  do if (pathsz(1,colnumb)=1).
    compute temp=make(nrow(zprobval),(nxvls*nzvls),0).
```

```
      loop k5 = 1 to nrow(zprobval).
        loop k6 =1 to nzvls.
          compute temp(k5,(((k4-1)*nzvls)+k6))=zprobval(k5,k6).
        end loop.
      end loop.
      compute indprova={tihsw,temp,tihswz}.
    else.
      compute indprova={tihsw,zprobval,tihswz}.
    end if.
  end if.
  do if (indmod(i,1)=3).
    compute
indprova=make((nrow(wprobval)*nrow(zprobval)),((ncol(wprobval)*nxvls)+(ncol(zprobval)*nxvls)+(n
wvls*nzvls*nxvls)),0).
    compute cntemp=1.
    loop k7=1 to nrow(wprobval).
      loop k8 =1 to nrow(zprobval).
        compute temp=wprobval(k7,:)*focaddon(1,k4).
        compute indprova(cntemp,(((k4-1)*nwvls)+1):(k4*(nwvls)))=temp.
        compute temp=zprobval(k8,:)*focaddon(1,k4).
        compute indprova(cntemp,  ((((k4-1)*nzvls)+1)+(nxvls*nwvls)) : ((((k4-
1)*nzvls)+1)+(nxvls*nwvls)+(nzvls-1)))=temp.
        compute cntemp=cntemp+1.
      end loop.
    end loop.
    do if (pathsz(1,colnumb)=0).
      compute temp=make(nrow(indprova),(ncol(zprobval)*nxvls),0).
      compute
indprova(:,((ncol(wprobval)*nxvls)+1):((ncol(wprobval)+ncol(zprobval))*nxvls))=temp.
    end if.
```

```
        do if (pathsw(1,colnumb)=0).
          compute temp=make(nrow(indprova),(ncol(wprobval)*nxvls),0).
          compute indprova(:,1:(ncol(wprobval)*nxvls))=temp.
        end if.
        do if (pathswz(1,colnumb)=1).
           compute cntemp=(ncol(wprobval)*nxvls)+(ncol(zprobval)*nxvls)+((k4-
1)*ncol(wprobval)*ncol(zprobval))+1.
           loop k7=1 to ncol(wprobval).
             loop k8=1 to ncol(zprobval).
               compute indprova(:,cntemp)=indprova(:,((ncol(wprobval)*(k4-
1))+k7))&*indprova(:,((((k4-1)*ncol(zprobval))+k8)+(nxvls*ncol(wprobval)))).
               compute cntemp=cntemp+1.
             end loop.
           end loop.
        end if.
      end if.
    end if.
    do if (k2 > 1).
      compute focbb=tihs(1,1).
      compute focbb=bootres(:,focbb).
      do if (indmmm(i,1)>0).
        compute imm=focbb(:,1).
        compute condbb=imm.
      end if.
      compute focaddon=1.
      compute cnt=cnt+1.
      compute placeh=1.
      do if (indmod(i,1)=1).
        compute tihsz=make(nrow(wprobval),nzvls,0).
```

```
  compute tihswz=make(nrow(wprobval),(nwvls*nzvls),0).
  compute indprova={wprobval,tihsz,tihswz}.
end if.
do if (indmod(i,1)=2).
  compute tihsw=make(nrow(zprobval),nwvls,0).
  compute tihswz=make(nrow(zprobval),(nwvls*nzvls),0).
  compute indprova={tihsw,zprobval,tihswz}.
end if.
do if (indmod(i,1)=3).
  compute
indprova=make((nrow(wprobval)*nrow(zprobval)),((ncol(wprobval)+ncol(zprobval))+(nwvls*nzvls)),0
).
  compute cntemp=1.
  loop k7=1 to nrow(wprobval).
    loop k8 =1 to nrow(zprobval).
      compute
indprova(cntemp,1:(ncol(wprobval)+ncol(zprobval)))={wprobval(k7,:),zprobval(k8,:)}.
      compute cntemp=cntemp+1.
    end loop.
  end loop.
  do if (pathsz(1,colnumb)=0).
    compute temp=make(nrow(indprova),ncol(zprobval),0).
    compute indprova(:,(ncol(wprobval)+1):(ncol(wprobval)+ncol(zprobval)))=temp.
  end if.
  do if (pathsw(1,colnumb)=0).
    compute temp=make(nrow(indprova),ncol(wprobval),0).
    compute indprova(:,1:ncol(wprobval))=temp.
  end if.
  do if (pathswz(1,colnumb)=1).
```

```
      compute cntemp=ncol(wprobval)+ncol(zprobval)+1.
      loop k7=1 to ncol(wprobval).
        loop k8=1 to ncol(zprobval).
          compute indprova(:,cntemp)=indprova(:,k7)&*indprova(:,(ncol(wprobval)+k8)).
          compute cntemp=cntemp+1.
        end loop.
      end loop.
    end if.
  end if.
end if.
do if (pathsw(1,colnumb)) = 1.
  compute wbb=tihs(cnt:(cnt+(placeh*nwvls)-1),1).
  compute wbb=bootres(:,wbb).
  compute immlbs2=wcatlab(1:nwvls,1).
  do if (zfirst=0).
    compute wfirst=1.
  end if.
  do if (indmmm(i,1)=1 or indmmm(i,1)=31 or indmmm(i,1)=51).
    compute imm=wbb(:,1).
    loop k7 = 1 to nwvls.
      compute imm={imm,wbb(:,(((k4-1)*nwvls*(k2=1))+k7))}.
    end loop.
    compute imm=imm(:,2:ncol(imm)).
  end if.
  do if (indmmm(i,1)=41 or indmmm(i,1)=51).
    compute condbb=make(nrow(bootres),1,0).
    loop k7 = 1 to nwvls.
      compute condbb={condbb,wbb(:,(((k4-1)*nwvls*(k2=1))+k7))}.
```

```
    end loop.
    compute condbb=condbb(:,2:ncol(condbb)).
  end if.
  compute cnt=cnt+(placeh*nwvls).
end if.
do if (pathsz(1,colnumb)) = 1.
  compute zbb=tihs(cnt:(cnt+(placeh*nzvls)-1),1).
  compute zbb=bootres(:,zbb).
  do if (wfirst=0).
    compute zfirst=1.
  end if.
  do if (indmmm(i,1) <> 31).
    compute immlbs2=zcatlab(1:nzvls,1).
  end if.
  do if (indmmm(i,1)=2 or indmmm(i,1)=31 or indmmm(i,1)=51).
    do if (indmmm(i,1)=2).
      compute imm=zbb(:,1).
    end if.
    loop k7 = 1 to nzvls.
      compute imm={imm,zbb(:,(((k4-1)*nzvls*(k2=1))+k7))}.
    end loop.
    do if (indmmm(i,1)=2 or indmmm(i,1)=51).
      compute imm=imm(:,2:ncol(imm)).
      do if (indmmm(i,1)=51).
        compute condbb={condbb,imm}.
      end if.
    end if.
  end if.
```

```
  compute cnt=cnt+(placeh*nzvls).
end if.
do if (pathswz(1,colnumb)) = 1.
  compute wzbb=tihs(cnt:(cnt+(placeh*nwvls*nzvls)-1),1).
  compute wzbb=bootres(:,wzbb).
  do if (indmmm(i,1)=41).
    compute imm=wzbb(:,1).


   /* the next end if was here */.
    loop k7=1 to nwvls*nzvls.
      compute imm={imm,wzbb(:,(((k4-1)*nzvls*nwvls*(k2=1))+k7))}.
    end loop.
  end if.
  do if (indmmm(i,1)=41).
    compute imm=imm(:,2:ncol(imm)).
    compute condbb={condbb,imm(:,(ncol(imm)-(nwvls*nzvls)+1):ncol(imm))}.
  end if.
  compute cnt=cnt+(placeh*nzvls*nwvls).
end if.
compute indprobe={focaddon,indprova(k1,:)}.
compute tucker={focbb,wbb,zbb,wzbb}.
loop k3=1 to ncol(indprobe).
  compute tucker(:,k3)=tucker(:,k3)*indprobe(1,k3).
end loop.
compute tucker2=tucker2&*rsum(tucker).
do if (indmmm(i,1) = 1 or indmmm(i,1)=2 or indmmm(i,1)=31 or indmmm(i,1)=41 or
indmmm(i,1)=51).
  do if (immset=1).
```

```
do if (ncol(imm2)=1 and ncol(imm) = 1).
 compute imm2=imm2&*imm.
end if.
do if (indmmm(i,1)=41 or indmmm(i,1)=51).
 do if ((ncol(condbb2) > 1) and (ncol(condbb)>1)).
  compute condbb2t=make(nrow(condbb2),(ncol(condbb2)*ncol(condbb)),-999999).
  compute k9=1.
  do if (wfirst=1).
   loop k7=1 to ncol(condbb2).
    loop k8 = 1 to ncol(condbb).
     compute condbb2t(:,k9)=condbb2(:,k7)&*condbb(:,k8).
     compute k9=k9+1.
    end loop.
   end loop.
  end if.
  do if (zfirst=1).
   loop k7=1 to ncol(condbb).
    loop k8 = 1 to ncol(condbb2).
     compute imm2t(:,k9)=condbb(:,k7)&*condbb2(:,k8).
     compute k9=k9+1.
    end loop.
   end loop.
  end if.
  compute condbb2=condbb2t.
  release condbb2t.
 end if.
 do if ((ncol(condbb2) > 1) and (ncol(condbb)=1)).
  loop k7 = 1 to ncol(condbb2).
```

```
    compute condbb2(:,k7)=condbb2(:,k7)&*condbb.
   end loop.
  end if.
  do if ((ncol(condbb2) = 1) and (ncol(condbb)>1)).
   loop k7 = 1 to ncol(condbb).
    compute condbb(:,k7)=condbb2&*condbb(:,k7).
   end loop.
   compute condbb2=condbb.
  end if.
end if.
do if (ncol(imm2) <> 1 and ncol(imm) <> 1).
  compute imm2t=make(nrow(imm2),(ncol(imm2)*ncol(imm)),-999999).
  compute k9=1.
  do if (wfirst=1).
   loop k7=1 to ncol(imm2).
    loop k8 = 1 to ncol(imm).
      compute imm2t(:,k9)=imm2(:,k7)&*imm(:,k8).
      compute k9=k9+1.
    end loop.
   end loop.
  end if.
  do if (zfirst=1).
   loop k7=1 to ncol(imm).
    loop k8 = 1 to ncol(imm2).
      compute imm2t(:,k9)=imm(:,k7)&*imm2(:,k8).
      compute k9=k9+1.
    end loop.
   end loop.
```

```
        end if.
        compute imm2=imm2t.
        release imm2t.
      end if.
      do if ((ncol(imm2) > 1) and (ncol(imm)=1)).
        loop k7=1 to ncol(imm2).
          compute imm2(:,k7)=imm2(:,k7)&*imm.
        end loop.
      end if.
      do if ((ncol(imm2) = 1) and (ncol(imm) > 1)).
        loop k7=1 to ncol(imm).
          compute imm(:,k7)=imm2&*imm(:,k7).
        end loop.
        compute imm2=imm.
      end if.
    end if.
    do if (immset=0).
      compute imm2=imm.
      do if (indmmm(i,1)=41 or indmmm(i,1)=51).
        compute condbb2=condbb.
      end if.
      compute immset=1.
    end if.
  end if.
  /* that is it for mmm loop */.
end loop.
/* end of looping through paths: k2 */.
compute indtemp=tucker2(1,1).
```

```
do if (indmmm(i,1)=12 or indmmm(i,1)=22).
  compute imm3={imm3,tucker2}.
  do if (k1=nrow(indmodva)).
    compute imm3=imm3(:,2:ncol(imm3)).
    compute immstop=ncol(imm3).
    loop k8=2 to immstop.
      do if (indmmmt(i,1)=1).
        compute imm3={imm3,(imm3(:,k8)-imm3(:,1))}.
      end if.
      do if (indmmmt(i,1)=2).
        compute imm3={imm3,(imm3(:,k8)-imm3(:,(k8-1)))}.
      end if.
      do if (indmmmt(i,1)=3).
        compute imm3={imm3,((rsum(imm3(:,(k8:immstop)))/(immstop-k8+1))-imm3(:,(k8-1)))}.
      end if.
      do if (indmmmt(i,1)=4).
        compute imm3={imm3,(imm3(:,k8)-(rsum(imm3(:,1:immstop))/immstop))}.
      end if.
    end loop.
    do if (indmmmt(i,1)<5).
      compute imm2=imm3(:,(immstop+1):ncol(imm3)).
      release imm3.
    end if.
  end if.
end if.
/* -1 used to be 0 */.
do if (indmmm(i,1)>-1 and (contrast = 1 or contrast = 2)).
  compute imm4={imm4,tucker2}.
```

```
do if (k1=nrow(indmodva) and k1 > 1).
 compute imm4=imm4(:,2:ncol(imm4)).
 compute immstop=ncol(imm4).
 compute condcont=make((immstop*(immstop-1)/2),6,-999).
 loop k8 = 1 to (immstop-1).
  loop k9 = (k8+1) to immstop.
   do if (contrast=1).
    compute imm4={imm4,(imm4(:,k9)-imm4(:,k8))}.
   end if.
   do if (contrast=2).
    compute imm4={imm4,(abs(imm4(:,k9))-abs(imm4(:,k8)))}.
   end if.
   compute condcont((ncol(imm4)-immstop),1)=imm4(1,k9).
   compute condcont((ncol(imm4)-immstop),2)=imm4(1,k8).
  end loop.
 end loop.
 compute imm4=imm4(:,(immstop+1):ncol(imm4)).
 loop k8=1 to ncol(imm4).
  compute condcont(k8,3)=imm4(1,k8).
  do if (boot > 0).
   bcboot3 databcbt = imm4(2:nrow(imm4),k8).
   compute condcont(k8,4:6)={bootse,llcit,ulcit}.
  end if.
 end loop.
 do if (boot=0).
  compute condcont=condcont(:,1:3).
 end if.
 compute indcontr=1.
```

```
   end if.
  end if.
  do if (boot > 0).
    bcboot3 databcbt = tucker2(2:nrow(tucker2),1).
    compute indtemp={indtemp, bootse,llcit,ulcit}.
  end if.
  compute condres(k1,(ncol(indmodva)+1):ncol(condres))=indtemp.
end loop.



/* end of looping through indirect effects: k1 */.
 /* Here is where the computations end */.
 compute condlbs={condlbs,"Effect"}.
 do if (boot > 0).
   compute condlbs={condlbs,"BootSE",  "BootLLCI",  "BootULCI"}.
 end if.
 do if (nxvls=1).
   print condres/title=" "/cnames=condlbs/format=!decpnt/space=0.
 else.
   compute condrlb=make(nrow(condres),1,xcatlab(k4,1)).
   print condres/title=" "/cnames=condlbs/rnames=condrlb/format=!decpnt/space=0.
 end if.
 compute dichadj=0.
 compute immcat=0.
 do if (indmmm(i,1)>0).
   do if (indmmm(i,1)=1 or indmmm(i,1)=12 or indmmm(i,1)=31).
     do if (wdich=1 and mcw=0).
       do if (indmmm(i,1) <> 12).
```

```
      compute imm2(:,1)=imm2(:,1)*(wmax-wmin).
    end if.
    do if (indmmm(i,1) <> 31).
      compute dichadj=1.
    end if.
  end if.
  do if ((mcw = 1 or mcw = 2) and indmmm(i,1) <> 31)).
    compute immcat=1.
  end if.
end if.
do if (indmmm(i,1)=2 or indmmm(i,1)=22 or indmmm(i,1)=31).
  do if (zdich=1 and mcz=0).
    do if (indmmm(i,1) = 31).
      compute imm2(:,(nwvls+1):ncol(imm2))=imm2(:,(nwvls+1):ncol(imm2))*(zmax-zmin).
    end if.
    do if (indmmm(i,1) = 2).
      compute imm2(:,1)=imm2(:,1)*(zmax-zmin).
    end if.
    do if (indmmm(i,1) <> 31).
      compute dichadj=1.
    end if.
  end if.
  do if ((mcz = 1 or mcz = 2) and indmmm(i,1) <> 31)).
      compute immcat=1.
  end if.
end if.
compute immtemp2=t(imm2(1,:)).
compute immtemp=immtemp2.
```

```
compute immlbs={"Index"}.
do if (boot > 0).
  compute immtemp=make(1,3,0).
  loop k7=1 to ncol(imm2).
    bcboot3 databcbt = imm2(2:nrow(imm2),k7).
    compute temp={bootse,llcit,ulcit}.
    compute immtemp={immtemp;temp}.
  end loop.
  compute immtemp=immtemp(2:nrow(immtemp),:).
  compute immtemp={immtemp2,immtemp}.
  compute immlbs={immlbs,"BootSE",  "BootLLCI",  "BootULCI"}.
end if.
do if (dichadj=0 and immcat=0 and indmmmt(i,1)<>5 and indmmm(i,1) < 100 ).
  do if (indmmm(i,1) < 30).
    print immtemp/title="      Index of moderated 
mediation:"/cnames=immlbs/rnames=immlbs2/format=!decpnt.
  end if.
  do if (indmmm(i,1) = 31).
    compute immlbs2={immlbs2;zcatlab(1:nzvls,1)}.
    print immtemp/title="      Indices of partial moderated 
mediation:"/cnames=immlbs/rnames=immlbs2/format=!decpnt.
  end if.
  do if (indmmm(i,1) = 41 or indmmm(i,1)=51).
    loop k7=1 to nwvls.
      compute immlbs2=zcatlab(1:nzvls,1).
      compute immtemp2=immtemp((((k7-1)*nzvls)+1):(((k7-1)*nzvls)+nzvls),:).
      do if (nwvls > 1).
        compute primodv={"        ", wcatlab(k7,1)}.
        print primodv/title="      Primary moderator:"/format=A8.
```

```
end if.
do if (nzvls=1).
  print immtemp2/title="      Index of moderated moderated 
mediation"/cnames=immlbs/format=!decpnt.
else.
  print immtemp2/title="      Indices of moderated moderated 
mediation"/cnames=immlbs/rnames=immlbs2/format=!decpnt.
end if.
compute cmmtemp=make(nrow(zprobval),4,0).
loop k8=1 to nrow(zprobval).
  compute condbb3=condbb2(:,((nwvls+1)+((k7-1)*nzvls)):((nwvls+1)+((k7-1)*nzvls)+(nzvls-
1))).
  do if (ncol(zprobval) > 1).
    compute condbb3=condbb3*mdiag(zprobval(k8,:)).
  else.
    compute condbb3=condbb3&*zprobval(k8,:).
  end if.
  compute condbb3={condbb2(:,k7),condbb3}.
  compute icmm=rsum(condbb3).
  compute cmmtemp(k8,1)=icmm(1,1).
  do if (boot > 0).
    bcboot3 databcbt = icmm(2:nrow(icmm),1).
    compute cmmtemp(k8,2:4)={bootse,llcit,ulcit}.
  end if.
end loop.
compute cmmtemp={zmodvals,cmmtemp}.
do if (boot=0).
  compute cmmtemp=cmmtemp(:,1:2).
end if.
```

```
            compute cmmlbs={znames,immlbs}.
            print cmmtemp/title="      Indices of conditional moderated mediation by W"/cnames=cmmlbs/format=!decpnt.
          end loop.
        end if.
      end if.
      do if (dichadj=1 or immcat=1 and indmmm(i,1) < 30).
        print immtemp/title="Index of moderated mediation (difference between conditional indirect effects):"/cnames=immlbs/rnames=immlbs2/format=!decpnt.
      end if.
    end if.
    do if (indcontr=1).
         compute condctlb={"Effect1","Effect2","Contrast","BootSE", "BootLLCI","BootULCI"}.
         print condcont/title=" Pairwise contrasts between conditional indirect effects (Effect1 minus Effect2)"/cnames=condctlb/format=!decpnt.
    end if.
    print/title= "---"/space=0.
   end loop.
  end if.
  /* end of moderated */.
 end loop.
end if.
/* This is the end of the moderated loop */.
end if.
!ENDDEFINE.


DEFINE modelest (y=!charend('/')/x=!charend('/')/type=!charend('/')/full=!charend('/')).
 do if (!type=1).
  compute b = inv(t(!x)*!x)*t(!x)*!y.
```

```
      compute modres=b.
      do if (!full=1).
        compute n1=nrow(!x).
        compute dfres=n1-(ncol(!x)).
        compute sstotal = t(!y-(csum(!y)/n1))*(!y-(csum(!y)/n1)).
        compute resid=!y-!x*b.
        compute ssresid = csum((resid)&**2).
        compute r2 = (sstotal-ssresid)/sstotal.
        compute adjr2 = 1-((1-r2)*(n1-1)/(dfres)).
        compute mse=ssresid/(n1-ncol(!x)).
        hcest3 x=x/resid=resid/hc=hc/mse=mse.
        compute seb=sqrt(diag(varb)).
        compute trat = b&/seb.
        compute p = 2*(1-tcdf(abs(trat), (dfres))).
        compute tval =  sqrt(dfres* (exp((dfres-(5/6))*((xp2/(dfres-(2/3)+(.11/dfres)))*(xp2/(dfres-
(2/3)+(.11/dfres)))))-1)).
        compute modres={modres,seb,trat,p}.
        compute modres={modres,(b-tval&*seb),(b+tval&*seb)}.
        compute modresl={"coeff",hclab,"t","p","LLCI","ULCI"}.
        compute lmat = ident(ncol(!x)).
        compute lmat = lmat(:,2:ncol(lmat)).
        compute fratio = (t(t(lmat)*b)*inv(t(lmat)*varb*lmat)*((t(lmat)*b)))/(ncol(!x)-1).
        compute pfr = 1-fcdf(fratio,(ncol(!x)-1),dfres).
        compute modsum={sqrt(r2),r2,mse,fratio,(ncol(!x)-1),dfres,pfr}.
        compute modsuml={"R","R-sq","MSE",hcflab,"df1","df2", "p"}.
      end if.
    end if.
!ENDDEFINE.
```

```
DEFINE makdummy (dd=!charend('/')/method=!charend('/')/custcodv=!charend('/') !default
(0)/custcode=!charend('/') !default("-999")).
 compute dd=!dd.
 compute temp = dd.
 compute temp(GRADE(dd(:,2)),:) = dd.
 compute dd = temp.
 compute dummy = design(dd(:,2)).
 compute nvls = ncol(dummy).
 compute nnvls = csum(dummy).
 compute mnvls = cmin(t(nnvls)).
 compute conmat1=1.
 do if (mnvls < 2).
   compute errcode(errs,1) = 5.
   compute errs = errs + 1.
   compute criterr = 1.
 end if.
 do if (nvls > 9).
   compute errcode(errs,1) = 4.
   compute errs = errs+1.
   compute criterr = 1.
 end if.
 do if (criterr = 0).
   compute dumok = 1.
   compute nnvls=make(nvls,1,0).
   compute nnvls(1,1)=dd(1,2).
   compute temp = 2.
   loop i = 2 to n.
```

```
  do if (dd(i,2) <> nnvls((temp-1),1)).
     compute nnvls(temp,1)=dd(i,2).
     compute temp = temp+1.
  end if.
end loop.
do if (!method > 0).
  compute x = dummy(:,2:ncol(dummy)).
  compute nx = ncol(x).
  compute minus1 = make(1,ncol(x),-1).
  compute xdes=make((nx+1),3,0).
  compute xdes(1,1)=dd(1,2).
  compute xdes(1,2)=1.
  compute temp = 2.
  loop k = 2 to n.
    do if (dd(k,2) <> dd((k-1),2)).
      compute xdes(temp,2) = k.
      compute xdes(temp,1) = dd(k,2).
      compute xdes((temp-1),3) = k-1.
      compute temp=temp+1.
    end if.
  end loop.
  compute xdes((temp-1),3)=n.
  compute xdes = {xdes, (xdes(:,3)-xdes(:,2)+1)}.
  do if (!method = 4).
    loop k = 1 to n.
      do if (rsum(x(k,:)) = 0).
        compute x(k,:) = minus1.
      end if.
```

```
  end loop.
end if.
do if (!method = 2 or !method = 3 or !method=5).
   loop k = 1 to n.
     do if (rsum(x(k,:)) > 0).
         loop i = 1 to ncol(x).
             do if (x(k,i) = 0).
                 compute x(k,i) = 1.
             else.
                  break.
             end if.
          end loop.
     end if.
   end loop.
   do if (!method = 3).
     compute conmat1={-8,1,1,1,1,1,1,1,1;
                      0,-7,1,1,1,1,1,1,1;
                      0,0,-6,1,1,1,1,1,1;
                      0,0,0,-5,1,1,1,1,1;
                      0,0,0,0,-4,1,1,1,1;
                      0,0,0,0,0,-3,1,1,1;
                      0,0,0,0,0,0,-2,1,1;
                      0,0,0,0,0,0,0,-1,1}.
     loop i = 1 to 8.
       compute conmat1(i,:)=conmat1(i,:)/(10-i).
     end loop.
     compute conmat1=t(conmat1((10-nvls):8,(10-nvls):9)).
     loop k=1 to n.
```

```
        compute x(k,:)=conmat1((rsum(x(k,:))+1),:).
      end loop.
    end if.
end if.
do if (!method = 5).
  compute custcode={!custcode}.
  do if (ncol(custcode) <> (nvls*(nvls-1))).
    compute errcode(errs,1) = (37+!custcodv).
    compute errs = errs + 1.
    compute criterr = 1.
  end if.
  do if (ncol(custcode) = (nvls*(nvls-1))).
    compute conmat1=make(nvls,(nvls-1),0).
    compute cnt=1.
    loop i = 1 to nvls.
      loop k = 1 to (nvls-1).
        compute conmat1(i,k)=custcode(1,cnt).
        compute cnt=cnt+1.
      end loop.
    end loop.
    /* compute conmat2=conmat1*inv(t(conmat1)*conmat1) */.
    /* compute conmat1=conmat2 */.
    loop k=1 to n.
      compute x(k,:)=conmat1((rsum(x(k,:))+1),:).
    end loop.
  end if.
end if.
compute xskip = 1.
```

```
      compute dummat = make((nx+1),nx,0).
      compute dummat((2:nrow(dummat)),:)=ident(nx).
      do if (!method = 4).
         compute dummat(1,:) = minus1.
      end if.
      do if (!method = 2).
         loop i = 2 to nrow(dummat).
            loop j = 1 to (i-1).
               compute dummat(i,j) = 1.
            end loop.
          end loop.
      end if.
      do if (!method = 3).
        compute dummat=conmat1.
      end if.
      do if (!method = 5 and criterr=0).
        compute dummat=conmat1.
      end if.
      compute dummat={nnvls, dummat}.
      compute x={dd(:,1),x}.
      compute temp = x.
      compute temp(GRADE(x(:,1)),:) = x.
      compute x = temp.
      release conmat1,temp,dd,xskip,xdes,dummy.
    end if.
  end if.
!ENDDEFINE.
```

```
DEFINE probe (decpnt=!charend('/') !default(F10.4)).
do if (criterr=0).
 compute threeway=0.
 compute didprint=0.
 compute didsome=0.
 compute sigintct=0.
 /* couldn't this loop be changed to <= i? */.
 /* start R */.
 loop jmed =1 to (nms+1).
  compute hasw=0.
  compute hasz=0.
  compute jnok=0.
  compute nm1vls=0.
  compute nm2vls=0.
  compute panelgrp=0.
  compute graphixs={"WITH", outnames(1,i), "BY"}.
  compute focpred4={" "}.
  compute intprint=0.
  compute modcat=0.
  /* start A */.
  do if (jmed <= i).
   do if ((jmed = 1) and ((i+1) = nrow(bcmat))).
    compute pathscnt=pathscnt+1.
   else.
    compute paths={paths,bcmat((i+1),jmed)}.
    compute pathsw={pathsw,wcmat((i+1),jmed)}.
    compute pathsz={pathsz,zcmat((i+1),jmed)}.
```

```
  compute pathswz={pathswz,wzcmat((i+1),jmed)}.
  compute temp=fochigh(:,pathscnt)&*bootloc(:,i).
  compute pathsfoc={pathsfoc,pathsfoc(:,1)}.
  do if (jmed=1).
    compute pathtype={pathtype,1}.
  end if.
  do if ((i+1)=nrow(bcmat)).
    compute pathtype={pathtype,3}.
  end if.
  do if (jmed > 1) and ((i+1) < nrow(bcmat)).
    compute pathtype={pathtype,2}.
  end if.
  do if (jmed=1 and nxvls > 1 and (bcmat((i+1),jmed)=1)).
    compute pathsfoc(:,(pathscn2+1))=temp(2:(nxvls+1),1).
  end if.
  do if ((jmed > 1) or (jmed=1 and nxvls=1)).
    compute temp=cmax(temp).
    compute pathsfoc(1,(pathscn2+1))=temp.
  end if.
  compute pathscnt=pathscnt+1.
  compute pathscn2=pathscn2+1.
  do if (i <= nms).
    compute pathsdv={pathsdv,mnames(1,i)}.
  end if.
  do if (i > nms).
    compute pathsdv={pathsdv,ynames}.
  end if.
end if.
```

```
compute coeffcol=coeffcol+1.
compute probettt=coeffs(1:nrow(b),coeffcol).
do if (jmed=1 and (bcmat((i+1),jmed)=1)).
  compute omni=make(nrow(probettt),nxvls,0).
  compute omnitmp=ident(nxvls).
  compute omni(2:(1+nxvls),:)=omnitmp.
end if.
do if (csum(probettt)>0).
  compute probvarb=make(csum(probettt),csum(probettt),999).
  compute probcoef=make(csum(probettt),1,999).
  compute coefflp2=1.
  loop coefflp=1 to nrow(probettt).
    do if (probettt(coefflp,1)=1).
      compute probcoef(coefflp2,1)=b(coefflp,1).
      compute coefflp2=coefflp2+1.
    end if.
  end loop.
  compute coefflp=0.
  compute coefflp2=0.
  loop iclp=1 to nrow(probettt).
    do if probettt(iclp,1)=1.
      compute coefflp=coefflp+1.
      compute coefflp2=coefflp.
      compute probvarb(coefflp,coefflp) = varb(iclp,iclp).
      do if (iclp < nrow(probettt)).
        loop jclp=(iclp+1) to nrow(probettt).
          do if (probettt(jclp,1)=1).
            compute coefflp2=coefflp2+1.
```

```
        compute probvarb(coefflp,coefflp2)=varb(iclp, jclp).
        compute probvarb(coefflp2,coefflp)=varb(iclp, jclp).
       end if.
      end loop.
     end if.
    end if.
   end loop.
   /* release coefflp,coefflp2,probettt */.
  end if.
end if.
/* end A */.

compute xprobval=xmodvals.
do if (nxvls > 1).
  compute xprobval=dummatx(:,2:ncol(dummatx)).
end if.
/* start B */.
do if ((wcmat((i+1),jmed)=1) and (zcmat((i+1),jmed)=0)).
  compute numplps=1.
  compute modvals=wmodvals.
  compute probeval=wmodvals.
  compute wheremv1=wherexw.
  compute nm1vls=nwvls.
  compute lpstsp={1,1}.
  compute modcat=0.
  compute jnmod=wtmp.
  compute jnmodlab=wnames.
  compute jnok=1.
```

```
compute jnmin=wmin.
compute jnmax=wmax.
compute wherejn1=2.
do if (jmed=1).
  compute wherejn3=wherexw(1,i).
  do if (nxvls > 1).
    compute jnok=0.
  end if.
end if.
do if (jmed > 1).
  compute wherejn1=wherem((jmed-1),i).
  compute wherejn3=wheremw(((2*jmed)-3),i).
end if.
do if (nwvls > 1).
  /* compute probeval=dummatw(:,2:ncol(dummatw)) */.
  compute probeval=wprobval.
  compute lpstsp(1,2)=ncol(probeval).
  compute modcat=1.
  compute jnok=0.
end if.
do if (wdich = 1).
  compute modcat=1.
  compute jnok=0.
end if.
compute problabs=wnames.
compute focpred3={wnames,"(W)"}.
compute hasw=1.
compute modgrph=wnames.
```

```
  compute intprint=1.
  compute sigintct=sigintct+1.
  compute printpbe=intpb(sigintct,1).
end if.
/* end B */.
/* start C */.
do if ((wcmat((i+1),jmed)=0) and (zcmat((i+1),jmed)=1)).
  compute numplps=1.
  compute modvals=zmodvals.
  compute probeval=zmodvals.
  compute wheremv1=wherexz.
  compute nm1vls=nzvls.
  compute lpstsp={1,1}.
  compute jnok=1.
  compute jnmod=ztmp.
  compute jnmin=zmin.
  compute jnmax=zmax.
  compute jnmodlab=znames.
  compute wherejn1=2.
  do if (jmed=1).
    compute wherejn3=wherexz(1,i).
    do if (nxvls > 1).
      compute jnok=0.
    end if.
  end if.
  do if (jmed > 1).
    compute wherejn1=wherem((jmed-1),i).
    compute wherejn3=wheremz(((2*jmed)-3),i).
```

```
  end if.
  do if (nzvls > 1).
    /* compute probeval=dummatz(:,2:ncol(dummatz)) */.
    compute probeval=zprobval.
    compute lpstsp(1,2)=ncol(probeval).
    compute modcat=1.
    compute jnok=0.
  end if.
  do if (zdich = 1).
    compute modcat=1.
    compute jnok=0.
  end if.
  compute problabs=znames.
  compute focpred3={znames,"(Z)"}.
  compute modgrph=znames.
  compute hasz=1.
  compute intprint=1.
  compute sigintct=sigintct+1.
  compute printpbe=intpb(sigintct,1).
end if.
/* end C */.
/* start D */.
do if ((wzcmat((i+1),jmed)=1) or ((wcmat((i+1),jmed)=1) and (zcmat((i+1),jmed)=1))).
  compute numplps=2.
  compute probecnt=1.
  compute intprint=1.
  do if (wzcmat((i+1),jmed)=1).
    compute sigintct=sigintct+1.
```

```
  compute printpbe=intpb(sigintct,1).
else.
  compute sigintct=sigintct+2.
  compute printpbe=cmin(intpb((sigintct-1):sigintct,1)).
end if.
compute panelgrp=1.
compute hasw=1.
compute hasz=1.
compute panelcde={"/PANEL","ROWVAR=",znames,"."}.
compute modgrph=wnames.
compute lpstsp={1,1;1,1}.
compute wheremv1=wherexw.
compute nm1vls=nwvls.
compute wheremv2=wherexz.
compute nm2vls=nzvls.
/* compute wprobval=wmodvals */.
compute jnok=0.
do if (wzcmat((i+1),jmed)=1).
  compute jnok=1.
end if.
do if (jmed > 1).
  compute mprobval=mmodvals.
end if.
do if (jmed=1).
  do if (nxvls > 1).
    compute jnok=0.
  end if.
end if.
```

```
do if (nwvls > 1).
  /* compute wprobval=dummatw(:,2:ncol(dummatw)) */.
  compute lpstsp(1,2)=ncol(wprobval).
  compute modcat=1.
  compute jnok=0.
end if.
do if (zdich=1).
  compute modcat=1.
  compute jnok=0.
end if.
/* compute zprobval=zmodvals */.
compute lpstsp(2,1)=lpstsp(1,2)+1.
compute lpstsp(2,2)=lpstsp(1,2)+1.
do if (nzvls > 1).
  /* compute zprobval=dummatz(:,2:ncol(dummatz)) */.
  compute lpstsp(2,1)=lpstsp(1,2)+1.
  compute lpstsp(2,2)=lpstsp(1,2)+ncol(zprobval).
  compute jnok=0.
end if.
do if (zdich=1).
  compute jnok=0.
end if.
compute omni3=make(nrow(b),(nxvls*nwvls),0).
do if (jmed > 1).
  compute omni3=make(nrow(b),nwvls,0).
end if.
compute focpred3={wnames,"(W)"}.
compute focpred4={"    ", "Mod var:", znames, "(Z)"}.
```

```
compute modvals=make((nrow(wmodvals)*nrow(zmodvals)),2,0).
compute probeval=make((nrow(wmodvals)*nrow(zmodvals)),(ncol(wprobval)+ncol(zprobval)),0).
loop probei= 1 to nrow(wmodvals).
  loop probej =1 to nrow(zmodvals).
    compute modvals(probecnt,1)=wmodvals(probei,1).
    compute probeval(probecnt,1:nwvls)=wprobval(probei,:).
    compute modvals(probecnt,2)=zmodvals(probej,1).
    compute probeval(probecnt,(nwvls+1):(nwvls+nzvls))=zprobval(probej,:).
    compute probecnt=probecnt+1.
  end loop.
end loop.
do if (wzcmat((i+1),jmed)=1).
  compute numplps=numplps+1.
  compute probprod=make(1,(ncol(wprobval)*ncol(zprobval)),0).
  compute lpstsp2={1,1}.
  compute lpstsp={lpstsp;lpstsp2}.
  compute lpstsp(3,1)=lpstsp(2,2)+1.
  compute lpstsp(3,2)=lpstsp(2,2)+ncol(probprod).
  compute jnmod=ztmp.
  compute jnmin=zmin.
  compute jnmax=zmax.
  compute jnmodlab=znames.
  do if (jmed = 1).
    compute wherejn1=wherexw(1,i).
    compute wherejn3=wherexwz(1,i).
  end if.
  do if (jmed > 1).
    /* compute wherejn1=wheremw((jmed-1),i) */.
```

```
      compute wherejn1=wheremw(((2*jmed)-3),i).
      compute wherejn3=wheremwz(((2*jmed)-3),i).
    end if.
    loop probei = 1 to nrow(wmodvals).
      loop probej = 1 to nrow(zmodvals).
        compute probtemp=1.
        loop probek = 1 to ncol(wprobval).
          compute probtemp={probtemp,(wprobval(probei,probek)&*zprobval(probej,:))}.
        end loop.
        compute probprod={probprod;probtemp(1,2:ncol(probtemp))}.
      end loop.
    end loop.
    compute probprod=probprod(2:nrow(probprod),:).
    compute probeval={probeval,probprod}.
  end if.
  compute problabs={wnames,znames}.
  release probecnt, probei, probej.
end if.
/* end D */.
/* Start E */.
do if (intprint=1).
  compute focpred={"   Focal", "predict:"}.
  do if (jmed=1).
    compute focpred={focpred,xnames,"(X)"}.
    compute focplotv=xmodvals.
  end if.
  do if (jmed >1).
    do if (nms > 1).
```

```
    compute focpred={focpred,mnames(1,(jmed-1)), medlb2(1,(jmed-1))}.
  end if.
  do if (nms = 1).
    compute focpred={focpred,mnames(1,(jmed-1)), "(M)"}.
  end if.
  compute focplotv=mmodvals(:,(jmed-1)).
end if.
compute focpred2={"    ", "Mod var:",focpred3}.
compute focpred={focpred;focpred2}.
do if (ncol(focpred4) > 1).
  compute focpred={focpred;focpred4}.
  compute focpred4={" "}.
end if.
release focpred2,focpred3.
do if ((plot = 1 or plot = 2) or (printpbe <= intprobe)).
  print focpred/title="----------"/format=A8/space=0.
end if.
compute foctmp=make(nrow(modvals),1,1).
compute probexpl=1.
compute probeva2={foctmp,probeval}.
do if (jmed=1 and nxs > 0 and mcx > 0).
  compute probexpl=nxvls.
end if.
compute foctmp=make(nrow(modvals),1,1).
compute modvals3=make(1,(6+ncol(problabs)),0).
compute probrown=make(nrow(probeval),1,0).
compute jtmp=1.
loop probei = 1 to nrow(probeval).
```

```
  compute probrown(probei,1)=jtmp.
  compute jtmp=jtmp+nxvls.
end loop.
release jtmp.
compute probrow=999.
compute modvarl=problabs.
/* start F */.
do if (plot = 1 or plot = 2 or nxvls > 1).
  compute plotvals=make((nrow(modvals)*nrow(focplotv)),(ncol(modvals)+1),999).
  loop ploti=1 to nrow(modvals).
    loop plotj=1 to nrow(focplotv).
      compute plotvals((((ploti-1)*nrow(focplotv))+plotj),2:ncol(plotvals))=modvals(ploti,:).
      compute plotvals((((ploti-1)*nrow(focplotv))+plotj),1)=focplotv(plotj,1).
    end loop.
  end loop.
  compute focpredn=3.
  do if (jmed=1).
    do if (nxvls > 1).
      compute focpredn=(nxvls+1).
    end if.
    do if (nxvls=1 and xdich=1).
      compute focpredn=2.
    end if.
  end if.
  compute meanmat=mdiag(means).
  compute onesmat=make(nrow(meanmat),(nrow(probeval)*focpredn),1).
  compute probeplt=t(mdiag(means)*onesmat).
  /* start G */.
```

```
do if (jmed=1).
 do if ((wcmat((i+1),1)=1) or (zcmat((i+1),1)=1)).
  compute plotcnt=1.
  compute iloops=nwpval*nzpval.
  compute plotmx=nxpval*nzpval.
  do if ((wcmat((i+1),1)=1) and (zcmat((i+1),1)=0)).
   compute iloops=nwpval.
   compute plotmx=nxpval.
  end if.
  do if ((wcmat((i+1),1)=0) and (zcmat((i+1),1)=1)).
   compute iloops=nzpval.
   compute plotmx=nxpval.
  end if.
  compute xestvals=make((nxpval*iloops),ncol(xprobval),-999).
  do if (wcmat((i+1),1)=1).
   compute westvals=make(nrow(xestvals),ncol(wprobval),-999).
  end if.
  do if (zcmat((i+1),1)=1).
   compute zestvals=make(nrow(xestvals),ncol(zprobval),-999).
  end if.
  loop ploti=1 to iloops.
   loop plotj=1 to nxpval.
    compute xestvals(plotcnt,:)=xprobval(plotj,:).
    compute plotcnt=plotcnt+1.
   end loop.
  end loop.
  compute plotcnt=1.
  compute plotcnt1=1.
```

```
compute plotcnt2=1.
compute plotcntz=1.
loop ploti = 1 to (iloops*nxpval).
  do if (wcmat((i+1),1)=1).
    compute westvals(ploti,:)=wprobval(plotcnt1,:).
  end if.
  do if ((wcmat((i+1),1)=0) and (zcmat((i+1),1)=1)).
    compute zestvals(ploti,:)=zprobval(plotcnt1,:).
  end if.
  do if ((wcmat((i+1),1)=1) and (zcmat((i+1),1)=1)).
    compute zestvals(ploti,:)=zprobval(plotcnt2,:).
    compute plotcntz=plotcntz+1.
  end if.
  compute plotcnt=plotcnt+1.
  do if (plotcnt > plotmx).
    compute plotcnt=1.
    compute plotcnt1=plotcnt1+1.
  end if.
  do if (plotcntz > nxpval).
    compute plotcnt2=plotcnt2+1.
    compute plotcntz=1.
    do if (plotcnt2 > nzpval).
      compute plotcnt2=1.
    end if.
  end if.
end loop.
compute probeplt(:,2:(1+(ncol(xestvals))))=xestvals.
do if (wcmat((i+1),1)=1).
```

```
    compute probeplt(:,(wherew(1,i)):(wherew(2,i)))=westvals.
   end if.
   do if (zcmat((i+1),1)=1).
    compute probeplt(:,(wherez(1,i)):(wherez(2,i)))=zestvals.
   end if.
  end if.
end if.
/* end G */.
/* start H */.
do if (jmed > 1).
  do if ((wcmat((i+1),jmed)=1) or (zcmat((i+1),jmed)=1)).
   compute plotcnt=1.
   compute iloops=nwpval*nzpval.
   compute plotmx=3*nzpval.
   do if ((wcmat((i+1),jmed)=1) and (zcmat((i+1),jmed)=0)).
    compute iloops=nwpval.
    compute plotmx=3.
    end if.
    do if ((wcmat((i+1),jmed)=0) and (zcmat((i+1),jmed)=1)).
     compute iloops=nzpval.
     compute plotmx=3.
    end if.
    compute mestvals=make((3*iloops),1,-999).
    do if (wcmat((i+1),jmed)=1).
     compute westvals=make(nrow(mestvals),ncol(wprobval),-999).
    end if.
    do if (zcmat((i+1),jmed)=1).
     compute zestvals=make(nrow(mestvals),ncol(zprobval),-999).
```

```
end if.
loop ploti=1 to iloops.
 loop plotj=1 to 3.
  compute mestvals(plotcnt,:)=mprobval(plotj,(jmed-1)).
  compute plotcnt=plotcnt+1.
 end loop.
end loop.
compute plotcnt=1.
compute plotcnt1=1.
compute plotcnt2=1.
compute plotcntz=1.
loop ploti = 1 to (iloops*3).
 do if (wcmat((i+1),jmed)=1).
  compute westvals(ploti,:)=wprobval(plotcnt1,:).
 end if.
 do if ((wcmat((i+1),jmed)=0) and (zcmat((i+1),jmed)=1)).
  compute zestvals(ploti,:)=zprobval(plotcnt1,:).
 end if.
 do if ((wcmat((i+1),jmed)=1) and (zcmat((i+1),jmed)=1)).
  compute zestvals(ploti,:)=zprobval(plotcnt2,:).
  compute plotcntz=plotcntz+1.
 end if.
 compute plotcnt=plotcnt+1.
 do if (plotcnt > plotmx).
  compute plotcnt=1.
  compute plotcnt1=plotcnt1+1.
 end if.
 do if (plotcntz > 3).
```

```
        compute plotcnt2=plotcnt2+1.
        compute plotcntz=1.
        do if (plotcnt2 > nzpval).
          compute plotcnt2=1.
        end if.
      end if.
    end loop.
    compute probeplt(:,wherem((jmed-1),i))=mestvals.
    do if (wcmat((i+1),jmed)=1).
      compute probeplt(:,(wherew(1,i)):(wherew(2,i)))=westvals.
    end if.
    do if (zcmat((i+1),jmed)=1).
      compute probeplt(:,(wherez(1,i)):(wherez(2,i)))=zestvals.
    end if.
  end if.
end if.
/* end H */.
/* Here I am doing the multiplications to produce data for the plot */.
compute prodloop = 1.
do if (jmed=1).
  compute prodloop=ncol(xestvals).
end if.
do if (wcmat((i+1),jmed))=1.
  compute plotcnt=0.
  loop ploti = 1 to prodloop.
    loop plotj = 1 to ncol(westvals).
      do if (jmed=1).
        compute probeplt(:,(wherexw(1,i)+plotcnt))=xestvals(:,ploti)&*westvals(:,plotj).
```

```
          end if.
          do if (jmed > 1).
            compute probeplt(:,(wheremw(((jmed*2)-3)
,i)+plotcnt))=mestvals(:,ploti)&*westvals(:,plotj).
          end if.
          compute plotcnt=plotcnt+1.
        end loop.
      end loop.
    end if.
    do if (zcmat((i+1),jmed))=1.
      compute plotcnt=0.
      loop ploti = 1 to prodloop.
        loop plotj = 1 to ncol(zestvals).
          do if (jmed = 1).
            compute probeplt(:,(wherexz(1,i)+plotcnt))=xestvals(:,ploti)&*zestvals(:,plotj).
          end if.
          do if (jmed > 1).
            compute probeplt(:,(wheremz(((jmed*2)-3),i)+plotcnt))=mestvals(:,ploti)&*zestvals(:,plotj).
          end if.
          compute plotcnt=plotcnt+1.
        end loop.
      end loop.
    end if.
    do if (wzcmat((i+1),jmed))=1.
      compute plotcnt=0.
      compute threeway=1.
      loop ploti = 1 to ncol(westvals).
        loop plotj = 1 to ncol(zestvals).
```

```
compute probeplt(:,(wherewz(1,i)+plotcnt))=westvals(:,ploti)&*zestvals(:,plotj).
compute plotcnt=plotcnt+1.
end loop.
end loop.
compute plotcnt=0.
loop plotk = 1 to prodloop.
loop ploti = 1 to ncol(westvals).
loop plotj = 1 to ncol(zestvals).
do if (jmed = 1).
compute
probeplt(:,(wherexwz(1,i)+plotcnt))=xestvals(:,plotk)&*westvals(:,ploti)&*zestvals(:,plotj).
end if.
do if (jmed > 1).
compute probeplt(:,(wheremwz(((jmed*2)-
3),i)+plotcnt))=mestvals(:,plotk)&*westvals(:,ploti)&*zestvals(:,plotj).
end if.
compute plotcnt=plotcnt+1.
end loop.
end loop.
end loop.
end if.
/* here is where we add holding constant products as needed */.
/* start I */.
loop newplp=1 to i.
do if (newplp <> jmed).
do if (wcmat((i+1),newplp))=1.
compute prodloop=1.
do if (newplp=1).
compute prodloop=nxvls.
```

```
            end if.
            compute plotcnt=0.
            loop ploti = 1 to prodloop.
              loop plotj = 1 to nwvls.
                do if (newplp = 1).
                  compute
probeplt(:,(wherexw(1,i)+plotcnt))=probeplt(:,(1+ploti))&*probeplt(:,(wherew(1,i)+plotj-1)).
                end if.
                do if (newplp > 1).
                  compute probeplt(:,(wheremw(((newplp*2)-3) ,i)+plotcnt))=probeplt(:,wherem((newplp-
1),i))&*probeplt(:,(wherew(1,i)+plotj-1)).
                end if.
                compute plotcnt=plotcnt+1.
              end loop.
            end loop.
          end if.
          do if (zcmat((i+1),newplp))=1.
            compute prodloop=1.
            do if (newplp=1).
              compute prodloop=nxvls.
            end if.
            compute plotcnt=0.
            loop ploti = 1 to prodloop.
              loop plotj = 1 to nzvls.
                do if (newplp = 1).
                  compute
probeplt(:,(wherexz(1,i)+plotcnt))=probeplt(:,(1+ploti))&*probeplt(:,(wherez(1,i)+plotj-1)).
                end if.
                do if (newplp > 1).
```

```
        compute probeplt(:,(wheremz(((newplp*2)-3),i)+plotcnt))=probeplt(:,wherem((newplp-1),i))&*probeplt(:,(wherez(1,i)+plotj-1)).
      end if.
      compute plotcnt=plotcnt+1.
    end loop.
  end loop.
end if.
do if (wzcmat((i+1),newplp))=1.
  compute plotcnt=0.
  do if (threeway=0).
    loop ploti = 1 to nwvls.
      loop plotj = 1 to nzvls.
        compute probeplt(:,(wherewz(1,i)+plotcnt))=probeplt(:,(wherew(1,i)+ploti-1))&*probeplt(:,(wherez(1,i)+plotj-1)).
        compute plotcnt=plotcnt+1.
      end loop.
    end loop.
  end if.
  compute prodloop=1.
  do if (newplp=1).
    compute prodloop=nxvls.
  end if.
  compute plotcnt=0.
  loop plotk = 1 to prodloop.
    loop ploti = 1 to nwvls.
      loop plotj = 1 to nzvls.
        do if (newplp = 1).
          compute probeplt(:,(wherexwz(1,i)+plotcnt))=probeplt(:,(1+plotk))&*probeplt(:,(wherew(1,i)+ploti-1))&*probeplt(:,(wherez(1,i)+plotj-1)).
```

```
            end if.
            do if (newplp > 1).
             compute probeplt(:,(wheremwz(((newplp*2)-
3),i)+plotcnt))=probeplt(:,wherem((newplp-1),i))&*probeplt(:,(wherew(1,i)+ploti-
1))&*probeplt(:,(wherez(1,i)+plotj-1)).
            end if.
            compute plotcnt=plotcnt+1.
           end loop.
          end loop.
         end loop.
        end if.
       end if.
      end loop.
      /* END I */.
      compute predvals=probeplt*b.
      compute sepred=make(nrow(plotvals),3,999).
      loop sei=1 to nrow(plotvals).
       compute ask=probeplt(sei,:).
       compute sepred(sei,1)=sqrt(ask*varb*t(ask)).
       compute sepred(sei,2)=predvals(sei,1)-tval*sepred(sei,1).
       compute sepred(sei,3)=predvals(sei,1)+tval*sepred(sei,1).
      end loop.
      compute prevloc=ncol(plotvals)+1.
      compute probeplt={plotvals,predvals}.
      do if (plot = 2).
       compute probeplt={probeplt,sepred}.
      end if.
      compute didsome=0.
     end if.
```

```
/* END F */.

/* here is the loop that is printing the conditional effects */.

/* this does conditional two way interactions */.

/* start J */.

do if ((wzcmat((i+1),jmed)=1) and (printpbe <= intprobe)).

 do if (jmed=1).

  compute omnilp2=nxvls*nwvls.

  compute omnitmp=ident(omnilp2).

  compute omni3(wherexw(1,i):wherexw(2,i),:)=omnitmp.

 end if.

 do if (jmed>1).

  compute omnilp2=nwvls.

  compute omnitmp=ident(omnilp2).

  compute omni3(wheremw(((jmed*2)-3),i):wheremw(((jmed*2)-2),i),:)=omnitmp.

 end if.

 compute omnif=make(1,4,0).

 compute condeff3=0.

 /* we had a problem here once  I think we can cut this */.

 /* mod1val=probeval(probek,1:nm1vls) */.

 loop omnilp1=1 to nrow(zprobval).

  loop omnilp=1 to (omnilp2).

   do if (jmed=1).

    compute omni3((wherexwz(1,i)+((omnilp-1)*nzvls)):(wherexwz(1,i)+((omnilp-
1)*nzvls)+(nzvls-1)),omnilp)=t(zprobval(omnilp1,:)).

   end if.

   do if (jmed > 1).

    compute omni3((wheremwz(((jmed*2)-3),i)+((omnilp-1)*nzvls)):(wheremwz(((jmed*2)-
3),i)+((omnilp-1)*nzvls)+(nzvls-1)),omnilp)=t(zprobval(omnilp1,:)).

   end if.
```

```
end loop.
compute condeff=t(omni3)*b.
compute condeff3={condeff3;condeff}.
ftest3 lm=omni3/bcoef=b/cv=varb/skip=1.
compute omnif={omnif;fresult}.
end loop.
compute omnif=omnif(2:nrow(omnif),:).
compute clabtmp=znames.
compute condeff3=condeff3(2:nrow(condeff3),:).
do if ((nxvls*nwvls)=1).
compute omnif={condeff3,omnif}.
compute clabtmp={clabtmp,"Effect"}.
end if.
compute omnif={zmodvals,omnif}.
compute clabtmp={clabtmp,hcflab,"df1","df2","p"}.
do if (jmed=1).
print omnif/title="Test of conditional X*W interaction at value(s) of
Z:"/cnames=clabtmp/format=!decpnt.
end if.
do if (jmed>1).
print omnif/title="Test of conditional M*W interaction at value(s) of
Z:"/cnames=clabtmp/format=!decpnt.
end if.
release omni3.
end if.
/* end J */.
/* start O */.
loop probei = 1 to probexpl.
do if (probexpl > 1).
```

```
  compute foctmp=make(nrow(modvals),probexpl,0).
  compute foctmp(:,probei)=foctmp(:,probei)+1.
  compute probtemp=make(nrow(modvals),1,0).
  loop probem = 1 to numplps.
    loop probek = 1 to nxvls.
      loop probej=lpstsp(probem,1) to lpstsp(probem,2).
        compute probtemp={probtemp,foctmp(:,probek)&*probeval(:,probej)}.
      end loop.
    end loop.
  end loop.
  compute probeva2=probtemp(:,2:ncol(probtemp)).
  compute probeva2={foctmp,probeva2}.
end if.
compute probres=probeva2*probcoef.
compute probrese=sqrt(diag(probeva2*probvarb*t(probeva2))).
compute tratio = probres&/probrese.
compute p = 2*(1-tcdf(abs(tratio), dfres)).
compute modvals2={modvals,probres,probrese,tratio, p}.
compute modvals2={modvals2,(probres-tval&*probrese),(probres+tval&*probrese)}.
compute problabs={problabs,"Effect",hclab,"t", "p", "LLCI", "ULCI"}.
/* start L */.
do if (probexpl > 1 and (printpbe <= intprobe)).
  do if (hasz = 1).
    compute printz=1.
  end if.
  do if (hasw=1).
    compute printw=1.
  end if.
```

```
compute probrlab=make(nrow(modvals),1,xcatlab(probei,1)).
compute modvals3={modvals3; modvals2}.
compute probrow={probrow;probrown}.
compute probrown=probrown+1.
do if (probei=probexpl).
  compute xproblab=xcatlab(1:nxvls,1).
  compute probrow=probrow(2:nrow(probrow),1).
  compute modvals3=modvals3(2:nrow(modvals3),:).
  compute temp=modvals3.
  compute temp(GRADE(probrow(:,1)),:)=modvals3.
  compute modvals3=temp.
  compute start2=1.
  compute problabs=problabs(1,(1+(ncol(modvarl))):ncol(problabs)).
  compute pstart=1.
  /* start K */.
  loop probek= 1 to nrow(probeval).
    compute endstart=start2+(nxvls-1).
    compute temp=modvals3(start2:endstart,(1+ncol(modvarl)):ncol(modvals3)).
    compute temp2=t(modvals3(start2:start2,1:ncol(modvarl))).
    compute trnames=t(modvarl).
    do if (probek > 1).
      print/title="----------"/space=0.
    else.
      print/title = "Conditional effects of the focal predictor at values of the moderator(s):".
      do if ((jmed=1) and (i = (nms+nys)) and (nms > 0)).
        do if (nxvls = 1).
          print/title = "(These are also the conditional direct effects of X on Y)"/space=0.
        else.
```

```
        print/title = "(These are also the relative conditional direct effects of X on Y)"/space=0.
      end if.
    end if.
    print.
  end if.
  print temp2/title = "Moderator value(s):"/rnames=trnames/format=!decpnt/space=0.
  print temp/title = " "/cnames=problabs/rnames=xproblab/format=!decpnt/space=0.
  compute start2=start2+nxvls.
  compute didsome=1.
  do if (jmed=1).
    compute mod1val=probeval(probek,1:nm1vls).
    loop omnilp=1 to nxvls.
      compute omni((wheremv1(1,i)+((omnilp-1)*nm1vls)):(wheremv1(1,i)+((omnilp-
1)*nm1vls)+(nm1vls-1)),omnilp)=t(mod1val).
      do if (nm1vls < ncol(probeval)).
        compute mod2val=probeval(probek,(nm1vls+1):(nm1vls+nm2vls)).
        compute omni((wheremv2(1,i)+((omnilp-1)*nm2vls)):(wheremv2(1,i)+((omnilp-
1)*nm2vls)+(nm2vls-1)),omnilp)=t(mod2val).
        do if ((nm1vls+nm2vls) < ncol(probeval)).
          compute intlen=nm1vls*nm2vls.
          compute modintvl=probeval(probek,(nm1vls+nm2vls+1):ncol(probeval)).
          compute omni((wherexwz(1,i)+((omnilp-1)*intlen)):(wherexwz(1,i)+((omnilp-
1)*intlen)+(intlen-1)),omnilp)=t(modintvl).
        end if.
      end if.
    end loop.
    /* compute condeff=t(omni)*b */.
    ftest3 lm=omni/bcoef=b/cv=varb/skip=1.
    compute clabtmp={hcflab,"df1","df2","p"}.
```

```
        print fresult/title="Test of equality of conditional means"/cnames=clabtmp/format=!decpnt.
        compute probetmp=probeplt(pstart:(pstart+nxvls),1).
        compute probetmp={probetmp,probeplt(pstart:(pstart+nxvls),prevloc:ncol(probeplt))}.
        compute pstart=pstart+(nxvls+1).
        compute clabtmp={xnames, outnames(1,i), hclab, "LLCI", "ULCI"}.
        print probetmp/title = "Estimated conditional means being
compared:"/cnames=clabtmp/format=!decpnt.
      end if.
    end loop.
    /* end K */.
    release probrow, start2, endstart, temp, temp2.
  end if.
end if.
/* end L */.
/* start N */.
do if (probexpl = 1 and (printpbe <= intprobe)).
  print/title = "Conditional effects of the focal predictor at values of the moderator(s):".
  print modvals2/cnames=problabs/title = " "/space=0/format=!decpnt.
  compute didsome=1.
  do if (hasz = 1).
    compute printz=1.
  end if.
  do if (hasw=1).
    compute printw=1.
  end if.
  /* start M */.
  do if (jn = 1 and jnok=1).
    do if (criterr = 0).
```

```
compute dfres=n-nrow(b).
compute jncrit =(dfres* (exp((dfres-(5/6))*((xp2/(dfres-(2/3)+(.11/dfres)))*(xp2/(dfres-(2/3)+(.11/dfres)))))-1)).
compute jnb1=b(wherejn1,1).
compute jnb3=b(wherejn3,1).
compute jnsb1=varb(wherejn1,wherejn1).
compute jnsb3=varb(wherejn3,wherejn3).
compute jnsb1b3=varb(wherejn1,wherejn3).
compute ajn =(jncrit*jnsb3)-(jnb3*jnb3).
compute bjn = 2*((jncrit*jnsb1b3)-(jnb1*jnb3)).
compute cjn = (jncrit*jnsb1)-(jnb1*jnb1).
compute radarg = (bjn*bjn)-(4*ajn*cjn).
compute den = 2*ajn.
compute nrts = 0.
do if (radarg >= 0 and den <> 0).
  compute x21 = (-bjn+sqrt(radarg))/den.
  compute x22 = (-bjn-sqrt(radarg))/den.
  compute roots = 0.
  do if (x21 >= jnmin and x21 <= jnmax).
    compute nrts = 1.
    compute roots = {roots; x21}.
  end if.
  do if (x22 >= jnmin and x22 <= jnmax).
    compute nrts = nrts + 1.
    compute roots = {roots; x22}.
  end if.
  compute roots={roots,make(nrow(roots),2,0)}.
end if.
```

```
do if (nrts > 0).
  compute roots = roots(2:nrow(roots),1:3).
  compute roots(1,2)=(csum(jnmod < roots(1,1))/n)*100.
  compute roots(1,3)=(csum(jnmod > roots(1,1))/n)*100.
  do if (nrow(roots)=2).
    compute roots(2,2)=(csum(jnmod < roots(2,1))/n)*100.
    compute roots(2,3)=(csum(jnmod > roots(2,1))/n)*100.
  end if.
  print roots/title = "Moderator value(s) defining Johnson-Neyman significance
region(s):"/clabels = "Value", "% below", "% above"/format !decpnt.
end if.
do if (nrts = 0).
  print/title = "There are no statistical significance transition points within the observed".
  print/title = "range of the moderator found using the Johnson-Neyman method."/space=0.
end if.
compute jnvals=make((21+nrts),7,0).
loop jni= 0 to 20.
  compute jnvals((jni+1),1)=jnmin+(jni*((jnmax-jnmin)/20)).
end loop.
do if (nrts > 0).
  loop jni = 1 to nrts.
    loop jnj = 2 to nrow(jnvals).
      do if ((roots(jni,1) > jnvals((jnj-1),1)) and (roots(jni,1) < jnvals(jnj,1))).
        compute jnvals((jnj+1):(21+jni),1)=jnvals(jnj:(20+jni),1).
        compute jnvals(jnj,1)=roots(jni,1).
      end if.
    end loop.
  end loop.
```

```
end if.
loop jni = 1 to nrow(jnvals).
  compute jnvals(jni,2)=jnb1+jnb3*jnvals(jni,1).
  compute jnvals(jni,3)=sqrt(jnsb1+2*jnvals(jni,1)*jnsb1b3+(jnvals(jni,1)*jnvals(jni,1))*jnsb3).
  compute jnvals(jni,4)=jnvals(jni,2)/jnvals(jni,3).
  compute jnvals(jni,5)=2*(1-tcdf(abs(jnvals(jni,4)), dfres)).
  compute jnvals(jni,6)=jnvals(jni,2)-sqrt(jncrit)*jnvals(jni,3).
  compute jnvals(jni,7)=jnvals(jni,2)+sqrt(jncrit)*jnvals(jni,3).
end loop.
compute jnclbs={jnmodlab,"Effect",hclab,"t", "p", "LLCI", "ULCI"}.
do if (((wcmat((i+1),jmed)=1) or (zcmat((i+1),jmed)=1)) and (wzcmat((i+1),jmed)=0)).
  print jnvals/title = "Conditional effect of focal predictor at values of the
moderator:"/cnames =jnclbs/format = !decpnt.
end if.
do if ((jmed = 1) and (wzcmat((i+1),jmed)=1)).
  print jnvals/title = "Conditional X*W interaction at values of the moderator Z:"/cnames
=jnclbs/format = !decpnt.
end if.
do if ((jmed > 1) and (wzcmat((i+1),jmed)=1)).
  print jnvals/title = "Conditional M*W interaction at values of the moderator Z:"/cnames
=jnclbs/format = !decpnt.
end if.
 end if.
end if.
/* END M */.
end if.
/* END N */.
do if ((i = (nms+nys)) and (jmed=1) and (bcmat(nrow(bcmat),1)=1)).
 do if (probei=1).
```

```
    compute direfflb=problabs.
    compute direff=modvals2.
  end if.
  do if (probei>1).
    compute direff={direff;modvals2}.
    /* compute direfflb=problabs */.
  end if.
end if.
compute intprint=0.
/* This does the contrast for conditional effects */.
do if ((jmed=1) and (i=1) and nms=0) and modcok=1).
  compute contvec2=make(2,1,1).
  compute contvec2={contvec2,wcontval,zcontval}.
  do if (wzcmat((i+1),jmed)=1).
    loop conti= 1 to ncol(wcontval).
      loop contj = 1 to ncol(zcontval).
        compute contvec2={contvec2,wcontval(:,conti)&*zcontval(:,contj)}.
      end loop.
    end loop.
  end if.
  compute conteff=contvec2*probcoef.
  compute contdiff=contvec2(1,:)-contvec2(2,:).
  compute contse=sqrt(contdiff*probvarb*t(contdiff)).
  compute conteffd=conteff(1,1)-conteff(2,1).
  compute p=2*(1-tcdf(abs(conteffd/contse), dfres)).
  compute contvec={contvec,conteff}.
  print/title="Contrast between conditional effects of X:".
```

```
print contvec/title=" "/rlabels="Effect1:","Effect2:"/cnames=problabs/format =
!decpnt/space=0.
compute contvec={conteffd,contse,conteffd/contse, p}.
compute contvec={contvec,(conteffd-(tval*contse))}.
compute contvec={contvec,(conteffd+(tval*contse))}.
compute contlabs={"Contrast", hclab, "t", "p", "LLCI", "ULCI"}.
print contvec/title="Test of Effect1 minus Effect2"/format=!decpnt/cnames=contlabs.
end if.
end loop.
/* end O */.
do if (plot = 1 or plot = 2).
compute datalabs={t(focpred(:,3)),outnames(1,i)}.
do if (plot = 1).
compute datalabs={datalabs,"."}.
end if.
do if (plot = 2).
compute datalabs={datalabs,"se", "LLCI", "ULCI."}.
end if.
print/title = "Data for visualizing the conditional effect of the focal predictor:".
print/title = "Paste text below into a SPSS syntax window and execute to produce plot."/space=0.
compute dumb = {" ", " ", " ", " ", " ", " ", " "}.
print datalabs/title = "DATA LIST FREE/"/format=A10.
print probeplt/title = "BEGIN DATA."/format.=!decpnt/space=0.
print/title = "END DATA."/space=0.
compute focgrph=datalabs(1,1).
compute graphix={focgrph,graphixs,modgrph}.
do if (((xdich=1) or (nxvls > 1)) and ((modcat=0) and (focgrph = xnames))).
compute graphix={modgrph,graphixs,focgrph}.
```

```
        end if.
        do if (panelgrp = 0).
          compute graphix={graphix,"."}.
        else.
          compute graphix={graphix,panelcde}.
        end if.
        print graphix/title = "GRAPH/SCATTERPLOT="/format=A8/space=0.
      end if.
    end if.
    /* end E */.
  end loop.
  /* end R */.
  release jmed, intprint,didprint.
end if.
!ENDDEFINE.
```

```
DEFINE PROCESS (vars=!charend('/') !default(xxxxx)/y=!charend('/') !default(xxxxx)/x=!charend('/') !default(xxxxx)
   /m=!charend('/') !default(xxxxx)/cov=!charend('/') !default(xxxxx)/z=!charend('/') !default(xxxxx)/w=!charend('/') !default(xxxxx)
   /v=!charend('/') !default(xxxxx)/q=!charend('/') !default(xxxxx)/total=!charend('/') !default(0)/varorder=!charend('/') !default(0)
   /mcw=!charend('/') !default(0)/mcx=!charend('/') !default(0)/mcz=!charend('/') !default(0)/normal=!charend('/') !default(0)
   /quantile=!charend('/') !default(999)/hc=!charend('/') !default(5)/hc3=!charend('/') !default(0)/moments=!charend('/') !default(0)
   /vmodval=!charend('/') !default(999)/qmodval=!charend('/') !default(999)/percent=!charend('/') !default(1)
   /cluster=!charend('/') !default(xxxxx)/conf=!charend('/') !default(95)/boot=!charend('/') !default(5000)
```

```
  /seed=!charend('/') !default(random)/bmatrix=!charend('/') !default("-999")/jn=!charend('/') !default(0)
  /wmatrix=!charend('/') !default("-999")/zmatrix=!charend('/') !default("-999")/wzmatrix=!charend('/') !default("-999")
  /cmatrix=!charend('/') !default("-999")/model = !charend('/') !default(999)/decimals=!charend('/') !default(F10.4)
  /matrices=!charend('/') !default (0)/covmy=!charend('/') !default(0)/center=!charend('/') !default(0)/ws=!charend('/') !default(0)
  /contrast=!charend('/') !default("999")/effsize=!charend('/') !default(0)/mc=!charend('/') !default(0)/mdichok=!charend('/') !default(0)
  /save=!charend('/') !default(0)/maxboot=!charend('/') !default(0)/covcoeff=!charend('/') !default(0)/ydichok=!charend('/') !default(0)
  /wmodval=!charend('/') !default("999")/intprobe=!charend('/') !default(.1)/zmodval=!charend('/') !default("999")/plot=!charend('/') !default(0)/
   modelbt = !charend('/') !default(0)/xcatcode=!charend('/')!default("-999")/wcatcode=!charend('/')!default("-999")/zcatcode=!charend('/')!default("-999")).
set printback = off.
set seed = !seed.
set mxloop = 100000000.
MATRIX.
compute errcode=make(100,1,0).
compute notecode=make(100,1,0).
compute model = trunc(!model).
compute maxwwarn=0.
compute minwwarn=0.
compute maxzwarn=0.
compute minzwarn=0.
compute toomany=0.
compute wdich=0.
compute zdich=0.
compute wnotev=0.
```

```
compute znotev=0.
compute nxpval=1.
compute nwpval=1.
compute nzpval=1.
compute errs=1.
compute notes=1.
compute criterr=0.
compute novar=0.
compute adjust=0.
compute ncs=0.
compute serial=0.
compute sobelok=0.
compute hasw=0.
compute hasz=0.
compute printw=0.
compute printz=0.
compute counterf=0.
compute wmodcust=0.
compute zmodcust=0.
compute cov = !quote(!cov).
compute varorder=(!varorder <> 0).
compute nws=0.
compute w=!quote(!w).
compute nzs=0.
compute z = !quote(!z).
compute nms=0.
compute m = !quote(!m).
compute nys=0.
```

```
compute y = !quote(!y).
compute nxs=0.
compute x = !quote(!x).
compute v = !quote(!v).
compute q = !quote(!q).
compute mcxok=0.
compute mcwok=0.
compute mczok=0.
compute xprod=0.
compute zprod=0.
compute wprod=0.
compute modcok=0.
compute hc3=trunc(!hc3).
compute jn=(!jn = 1).
compute effsize=(!effsize=1).
compute normal=(!normal=1).
compute sobelok=0.
compute normal=(!normal=1).
compute mdichok=(!mdichok=1).
compute ydichok=(!ydichok=1).
compute contrast={!contrast}.
compute ncontr=ncol(contrast).
compute ncontrow=nrow(contrast).
do if (contrast(1,1) = 999).
  compute ncontr=1.
  compute contrast=0.
end if.
do if (ncontr = 1).
```

```
  compute contrast=trunc(contrast).
  do if (contrast > 2 or contrast < 0)).
    compute ncontr=1.
    compute contrast = 0.
  end if.
end if.
do if (ncontr > 1).
    compute contvec=contrast.
    compute contrast=3.
    do if (ncontrow > 1).
      compute contrast=0.
      compute modcok=1.
      compute wcontval=contvec(:,1).
      compute zcontval=contvec(:,2).
      do if ((ncontr <> 2) or (ncontrow <> 2)).
        compute notecode(notes,1) = 19.
        compute notes = notes + 1.
        compute modcok=0.
      end if.
    end if.
end if.
do if (varorder = 1).
  compute notecode(notes,1) = 21.
  compute notes = notes + 1.
end if.
do if (!vmodval <> 999 or !qmodval <> 999).
  compute notecode(notes,1) = 22.
  compute notes = notes + 1.
```

```
end if.
compute modelbt=(!modelbt=1).
compute cluster=!quote(!cluster).
compute matrices=(!matrices=1).
compute covcoeff=(!covcoeff=1).
compute covmy=trunc(!covmy).
do if (covmy < 0 or covmy > 2).
   compute covmy = 0.
end if.
compute boot = abs(trunc(!boot)).
compute mc=abs(trunc(!mc)).
compute hc=trunc(!hc).
compute intprobe = !intprobe.
do if (intprobe < 0 or intprobe > 1).
  compute intprobe = .10.
end if.
compute plot=trunc(!plot).
do if (plot < 0 or plot > 2).
  compute plot=0.
end if.
compute total=(!total=1).
compute dototal=0.
compute saveboot = (!save = 1).
compute saveest=(!save = 2).
do if (hc >= 0 and hc < 5).
  compute notecode(notes,1) = 4.
  compute notes = notes + 1.
end if.
```

```
do if (hc > 5 or hc < 0).
  compute hc=5.
end if.
compute mcw=trunc(!mcw).
compute mcz=trunc(!mcz).
compute mcx=trunc(!mcx).
do if (mcx > 0 and model = 74).
  compute mcw=mcx.
end if.
compute nxvls=1.
compute nmvls=1.
compute nwvls=1.
compute nzvls=1.
compute paths=999.
compute pathsw=999.
compute pathsz=999.
compute pathswz=999.
compute pathsmod=999.
compute pathtype=999.
compute obscoeff=999.
compute pathsdv={" "}.
compute quantile=1.
do if (!quantile<>999).
  compute notecode(notes,1) = 23.
  compute notes = notes + 1.
end if.
compute moments=(!moments=1).
do if (moments=1).
```

```
  compute quantile=0.

end if.

compute center=(!center=1).

compute bmatrix={!bmatrix}.

compute wmatrix={!wmatrix}.

compute zmatrix={!zmatrix}.

compute wzmatrix={!wzmatrix}.

compute cmatrix={!cmatrix}.

compute xcatcode={!xcatcode}.

compute wcatcode={!wcatcode}.

compute zcatcode={!zcatcode}.

compute needed=0.

compute conf=!conf.

do if (trunc(!conf) >= 100 or (trunc(!conf) <= 50)).

  compute conf = 95.

  compute notecode(notes,1)=2.

  compute notes=notes+1.

end if.

do if (model > 0 and model < 4 and modelbt=0).

  compute boot=0.

  compute mc=0.

end if.

do if (boot > 0 and mc > 0).

  compute boot=0.

end if.

compute p0=-.322232431088.

compute p1 = -1.

compute p2 = -.342242088547.
```

```
compute p3 = -.0204231210245.

compute p4 = -.0000453642210148.

compute q0 = .0993484626060.

compute q1 = .588581570495.

compute q2 = .531103462366.

compute q3 = .103537752850.

compute q4 = .0038560700634.

compute alpha2 = (1-(conf/100))/2.

compute cilm=alpha2*2.

compute y5=sqrt(-2*ln(alpha2)).

compute xp2=(y5+((((y5*p4+p3)*y5+p2)*y5+p1)*y5+p0)/((((y5*q4+q3)*y5+q2)*y5+q1)*y5+q0)).

compute medlb={"   M1  :";"   M2  :";"   M3  :";"   M4  :";"   M5  :";"   M6  :";"   M7  :";"   M8  :";"   M9
:";"   M10 :"}.

compute medlb2={"(M1)","(M2)","(M3)","(M4)","(M5)","(M6)","(M7)","(M8)","(M9)","(M10)"}.

compute xlb={"   X1  :";"   X2  :";"   X3  :";"   X4  :";"   X5  :";"   X6  :";"   X7  :";"   X8  :";"   X9  :"}.

compute highlbw={"M1*W"; "M2*W"; "M3*W"; "M4*W"; "M5*W"; "M6*W"; "M7*W"; "M8*W";
"M9*W"; "M10*W"}.

compute highlbz={"M1*Z"; "M2*Z"; "M3*Z"; "M4*Z"; "M5*Z"; "M6*Z"; "M7*Z"; "M8*Z";
"M9*Z";"M10*Z"}.

compute highlbwz={"M1*W*Z"; "M2*W*Z"; "M3*W*Z"; "M4*W*Z"; "M5*W*Z"; "M6*W*Z";
"M7*W*Z"; "M8*W*Z"; "M9*W*Z";"M10*W*Z"}.


/* check to see if model number specified is acceptable for this version */.

compute skipwz=0.

compute validm={1,1,1,1,1,1,1,1,1,1,1,1,1,1,1,1,1,1,1,1,1,1,0,0,0,0,0,1,1,0,0,0,0,


0,0,0,0,0,0,0,0,0,0,0,0,0,0,0,0,0,0,0,0,0,0,0,0,1,1,1,1,1,1,1,1,1,1,1,1,1,1,1,1,0,1,1,0,0,0,1,1,1,1,1,1,1,1,
1,1,1,1,1}.

do if ((!ws=1)=1).

   compute errcode(errs,1)=42.
```

```
    compute errs=errs+1.
    compute criterr=1.
end if.
do if (model > 0 and model < 93).
  do if (validm(1,model)=0).
    do if (model <> 74).
      compute errcode(errs,1)=6.
      compute errs=errs+1.
      compute criterr=1.
    end if.
    do if (model = 74).
      compute errcode(errs,1)=46.
      compute errs=errs+1.
      compute criterr=1.
    end if.
  end if.
  release validm.
end if.
do if ((model > 92 or model < 0) and model <> 999)).
  compute errcode(errs,1)=7.
  compute errs=errs+1.
  compute criterr=1.
end if.
do if (model = 999 and bmatrix(1,1)=-999).
  compute errcode(errs,1)=24.
  compute errs=errs+1.
  compute criterr=1.
end if.
```

```
do if (model <> 999 and bmatrix(1,1) <> -999).

  compute errcode(errs,1)=25.

  compute errs=errs+1.

  compute criterr=1.

end if.

do if ((model = 74 or (model > 0 and model < 4)) and ((wmatrix(1,1) <> -999) or (zmatrix(1,1)<>-999)
or (wzmatrix(1,1)<>-999)))).

  compute errcode(errs,1)=41.

  compute errs=errs+1.

  compute criterr=1.

end if.

/* Tell user that HC3 is discontinued */.

do if (hc3 <> 0).

  compute notecode(notes,1) = 5.

  compute notes = notes + 1.

  do if (hc3 = 1).

    compute hc=3.

  end if.

end if.

/* Do not allow V or Q */.

do if ((v <> "xxxxx") or (q <> "xxxxx")).

  compute errcode(errs,1)=14.

  compute errs=errs+1.

  compute criterr=1.

end if.

/* Cluster option is disabled */.

do if (cluster <> "xxxxx").

  compute errcode(errs,1)=27.
```

```
  compute errs=errs+1.
  compute criterr=1.
end if.
/* Check to make sure an X and a Y are specified */.
do if ((y = "xxxxx") or (x = "xxxxx")).
  compute errcode(errs,1)=1.
  compute errs=errs+1.
  compute criterr=1.
end if.
/* Check to make sure an M is specified if needed */.
do if ((m = "xxxxx") and model > 3).
  compute errcode(errs,1)=8.
  compute errs=errs+1.
  compute criterr=1.
end if.

/* start A */.
do if (criterr=0).
  /* extract data and variable names */.
  get ytmp/variables = !y/names = ynames/MISSING = 99999.
  compute nys=ncol(ytmp).
  compute needed=nys.
  compute n=nrow(ytmp).
  compute varnames={ynames}.
  compute dat=ytmp.
  longchk variab=!y.
  compute modelvar={!quote(!model);t(ynames)}.
  do if (!model=999).
```

```
  compute modelvar(1,1)="CUSTOM".
end if.
get xtmp/variables = !x/names = xnames/MISSING = 99999.
compute nxs=ncol(xtmp).
compute n=nrow(xtmp).
compute needed=needed+nxs.
compute varnames={varnames,xnames}.
compute xcatlab=t(xnames).
compute dat={dat,xtmp}.
longchk variab=!x.
compute modelvar={modelvar;t(xnames)}.
do if (nxs = 1).
  compute modelvlb={"Model  :";"    Y  :";"    X  :"}.
else.
  compute modelvlb={"Model  :";"    Y  :";xlb(1:nxs,1)}.
end if.

do if (m <> "xxxxx").
  get mtmp/variables = !m/names = mnames/MISSING = 99999.
  compute nms=ncol(mtmp).
  compute mprod=make(1,nms,0).
  compute n=nrow(mtmp).
  compute needed=needed+nms.
  compute varnames={varnames,mnames}.
  compute dat={dat,mtmp}.
  compute modelvar={modelvar;t(mnames)}.
  compute x2m=make(99,nms,0).
  compute m2y=make(99,nms,0).
```

```
  compute onem=make(nms,1,1).
  longchk variab=!m.
  do if (nms > 1 and nms < 11).
    compute modelvlb={modelvlb;medlb(1:nms,1)}.
  else.
    compute modelvlb={modelvlb;"    M  :"}.
  end if.
  do if (nms > 0 and model < 4).
    compute errcode(errs,1)=9.
    compute errs=errs+1.
    compute criterr=1.
  end if.
end if.
compute wlocatet=0.
compute wlocate=0.
do if (w <> "xxxxx").
  get wtmp/variables = !w/names = wnames/MISSING = 99999.
  compute nws=ncol(wtmp).
  compute n=nrow(wtmp).
  longchk variab=!w.
  compute varnames={varnames,wnames}.
  compute wlocate=ncol(varnames).
  do if (model=74).
    compute wlocatet=1.
    do if (xnames <> wnames).
      compute errcode(errs,1)=45.
      compute errs=errs+1.
      compute criterr=1.
```

```
    end if.
  end if.
  compute wcatlab=t(wnames).
  compute dat={dat,wtmp}.
  compute modelvar={modelvar;t(wnames)}.
  compute modelvlb={modelvlb;"    W  :"}.
end if.
do if (z <> "xxxxx").
  get ztmp/variables = !z/names = znames/MISSING = 99999.
  compute nzs=ncol(ztmp).
  compute n=nrow(ztmp).
  longchk variab=!z.
  compute varnames={varnames,znames}.
  compute zcatlab=t(znames).
  compute dat={dat,ztmp}.
  compute modelvar={modelvar;t(znames)}.
  compute modelvlb={modelvlb;"    Z  :"}.
end if.
do if (cov <> "xxxxx").
  get ctmp/variables = !cov/names = covnames/MISSING = 99999.
  compute ncs=ncol(ctmp).
  compute n=nrow(ctmp).
  longchk variab=!cov.
  compute varnames={varnames,covnames}.
  compute dat={dat,ctmp}.
end if.
/* check for too many variables specified */.
do if (nws > 1 or nzs > 1 or nys > 1 or nxs > 1).
```

```
  compute errcode(errs,1)=3.
  compute errs=errs+1.
  compute criterr=1.
end if.
/* check for proper number of mediators in some models */.
do if ((model = 80 or model = 81) and (nms < 3 or nms > 6)).
  compute errcode(errs,1)=32.
  compute errs=errs+1.
  compute criterr=1.
end if.
do if (model = 82 and nms <> 4).
  compute errcode(errs,1)=33.
  compute errs=errs+1.
  compute criterr=1.
end if.
do if (nms > 10).
  compute errcode(errs,1)=37.
  compute errs=errs+1.
  compute criterr=1.
end if.
do if ((model = 6 or (model > 82 and model < 999)) and (nms < 2 or nms > 6)).
  compute errcode(errs,1)=34.
  compute errs=errs+1.
  compute criterr=1.
end if.
/* check for redundant variable names */.
compute match=0.
loop i = 1 to (ncol(varnames)-1).
```

```
  loop j = (i+1) to ncol(varnames).
    do if (varnames(i)=varnames(j)).
      compute match=1.
      do if (wlocatet=1 and i=2 and j=wlocate).
        compute match=0.
      end if.
    end if.
  end loop.
end loop.
do if (match=1).
  compute errcode(errs,1)=2.
  compute errs=errs+1.
  compute criterr=1.
end if.
/* listwise deletion */.
compute ninit=nrow(dat).
compute rownum=make(ninit,1,0).
loop i = 1 to ninit.
  compute rownum(i,1)=i.
end loop.
compute dat={rownum,dat}.
compute j=1.
compute missrow=0.
loop i = 1 to n.
  do if (rsum(dat(i,2:ncol(dat))=99999)=0).
    compute dat(j,:)=dat(i,:).
    compute j=j+1.
  else.
```

```
    compute missrow={missrow;dat(i,1)}.
  end if.
end loop.
compute rownum=dat(1:(j-1),1).
do if (nrow(missrow) > 1).
  compute missrow=t(missrow(2:nrow(missrow),1)).
end if.
compute dat=dat(1:(j-1),2:ncol(dat)).
compute n=nrow(dat).
compute nmiss=ninit-n.
/* reextract the data columns after deletion */.
compute ytmp=dat(:,1:nys).
describe descdatf=ytmp.
compute ysd=desctmp(2,:).
do if ((desctmp(8,1)=1) and (ydichok <> 1)).
  compute errcode(errs,1)=44.
  compute errs=errs+1.
  compute criterr=1.
end if.
compute xtmp=dat(:,(nys+1):(nys+nxs)).
describe descdatf=xtmp.
compute xsd=desctmp(2,:).
compute xmodvals=modvals.
compute nxpval=nrow(xmodvals).
compute xprobval=xmodvals.
compute xdich=desctmp(8,1).
do if (model = 74 and xdich=1).
  compute counterf=1.
```

```
end if.
do if (nms > 0).
  compute mtmp=dat(:,(nys+nxs+1):(nys+nxs+nms)).
  describe descdatf=mtmp.
  do if ((rsum(desctmp(8,:))>0) and (mdichok <> 1)).
    compute errcode(errs,1)=43.
    compute errs=errs+1.
    compute criterr=1.
  end if.
  compute mmodvals=modvals.
  compute mprobval=mmodvals.
end if.
do if (nws > 0).
  compute wtmp=dat(:,(nys+nxs+nms+1):(nys+nxs+nms+nws)).
  describe descdatf=wtmp.
  compute wmodvals=modvals.
  compute wdich=desctmp(8,1).
  compute wmin=desctmp(3,1).
  compute wmax=desctmp(4,1).
  compute minwwarn=minwarn.
  compute maxwwarn=maxwarn.
  compute wnotev=mnotev.
  compute wmodval={!wmodval}.
  compute nwcontr=ncol(wmodval).
  do if (wmodval(1,1) <> 999).
    compute wmodvals=wmodval(1,1).
    compute wmodcust=1.
    do if (nwcontr > 1).
```

```
      compute wmodvals=t(wmodval).
    end if.
    compute minwwarn=0.
    compute maxwwarn=0.
    compute wnotev=0.
  end if.
  compute wprobval=wmodvals.
  compute nwpval=nrow(wmodvals).
end if.
do if (nzs > 0).
  compute ztmp=dat(:,(nys+nxs+nms+nws+1):(nys+nxs+nms+nws+nzs)).
  describe descdatf=ztmp.
  compute zmodvals=modvals.
  compute zdich=desctmp(8,1).
  compute zmin=desctmp(3,1).
  compute zmax=desctmp(4,1).
  compute minzwarn=minwarn.
  compute maxzwarn=maxwarn.
  compute znotev=mnotev.
  compute zmodval={!zmodval}.
  compute nzcontr=ncol(zmodval).
  do if (zmodval(1,1) <> 999).
    compute zmodvals=zmodval(1,1).
    compute zmodcust=1.
    do if (nzcontr > 1).
      compute zmodvals=t(zmodval).
    end if.
    compute minzwarn=0.
```

```
    compute maxzwarn=0.
    compute znotev=0.
  end if.
  compute zprobval=zmodvals.
  compute nzpval=nrow(zmodvals).
end if.
do if (ncs > 0).
  compute ctmp=dat(:,(nys+nxs+nms+nws+nzs+1):(nys+nxs+nms+nws+nzs+ncs)).
  describe descdatf=ctmp.
end if.
compute n=nrow(ytmp).
compute ones=make(n,1,1).
release dat.
/* create codes for categorical variables */.
do if (nws > 0 and mcw > 0).
  compute tmp={rownum,wtmp(:,1)}.
  makdummy dd=tmp/method=mcw/custcodv=2/custcode=!wcatcode.
  compute wmodvals=nnvls.
  compute nwpval=nrow(wmodvals).
  do if (criterr=0).
    compute minwwarn=0.
    compute maxwwarn=0.
    compute wnotev=0.
    compute wtmp=x(:,2:ncol(x)).
    compute wcatlab={"W1";"W2";"W3";"W4";"W5";"W6";"W7";"W8";"W9"}.
    compute nwvls=nvls-1.
    compute mcwok=1.
    compute dummatw=dummat.
```

```
    compute wprobval=dummatw(:,2:ncol(dummatw)).
    do if (modcok=1).
      compute wcontval=make(2,ncol(wprobval),-999).
      compute temp=0.
      loop i = 1 to 2.
        loop j = 1 to nrow(dummatw).
          do if (contvec(i,1)=dummatw(j,1)).
            compute wcontval(i,:)=wprobval(j,:).
            compute temp=temp+1.
          end if.
        end loop.
      end loop.
      do if (temp < 2).
        compute notecode(notes,1) = 20.
        compute notes = notes + 1.
        compute modcok=0.
      end if.
    end if.
    do if (wmodval(1,1) <> 999).
      compute notecode(notes,1) = 9.
      compute notes = notes + 1.
    end if.
    release tmp, dummat.
  end if.
end if.
do if (nzs > 0 and mcz > 0).
  compute tmp={rownum,ztmp(:,1)}.
  makdummy dd=tmp/method=mcz/custcodv=3/custcode=!zcatcode.
```

```
compute zmodvals=nnvls.
compute nzpval=nrow(zmodvals).
do if (criterr=0).
 compute minzwarn=0.
 compute maxzwarn=0.
 compute znotev=0.
 compute ztmp=x(:,2:ncol(x)).
 compute zcatlab={"Z1";"Z2";"Z3";"Z4";"Z5";"Z6";"Z7";"Z8";"Z9"}.
 compute nzvls=nvls-1.
 compute mczok=1.
 compute dummatz=dummat.
 compute zprobval=dummatz(:,2:ncol(dummatz)).
 do if (modcok=1).
  compute zcontval=make(2,ncol(zprobval),-999).
  compute temp=0.
  loop i = 1 to 2.
   loop j = 1 to nrow(dummatz).
    do if (contvec(i,2)=dummatz(j,1)).
     compute zcontval(i,:)=zprobval(j,:).
     compute temp=temp+1.
    end if.
   end loop.
  end loop.
  do if (temp < 2).
   compute notecode(notes,1) = 20.
   compute notes = notes + 1.
   compute modcok=0.
  end if.
```

```
    end if.
    do if (zmodval(1,1) <> 999).
      compute notecode(notes,1) = 10.
      compute notes = notes + 1.
    end if.
    release tmp, dummat.
  end if.
end if.
do if (nxs > 0 and mcx > 0).
  compute tmp={rownum,xtmp(:,1)}.
  makdummy dd=tmp/method=mcx/custcodv=1/custcode=!xcatcode.
  do if (criterr=0).
    compute xtmp=x(:,2:ncol(x)).
    compute xcatlab={"X1";"X2";"X3";"X4";"X5";"X6";"X7";"X8";"X9"}.
    compute nxvls=nvls-1.
    compute xdich=(nvls=2).
    compute mcxok=1.
    compute dummatx=dummat.
    compute xmodvals=dummatx(:,1).
    compute nxpval=nrow(xmodvals).
    release tmp, dummat.
  end if.
end if.
compute intlab=make(100,1," ").
!do !i = 1 !to 100.
  !let !v2=!concat(Int_,!unquote(!quote(!i))).
  compute intlab(!i,1)=!quote(!v2).
!doend.
```

```
  compute bcmat=make(needed,needed,0).
  compute wcmat=make(needed,needed,0).
  compute zcmat=make(needed,needed,0).
  compute wzcmat=make(needed,needed,0).
  compute wsum=0.
  compute zsum=0.
  compute wzsum=0.
end if.
/* end A */.



/* DEFINE MODEL MATRICES FOR CANNED MODELS */.
do if (criterr = 0 and model <> 999).
  /* X->MW, X->MZ, X->MWZ, M->YW, M->YZ, M->YWZ, X->YW, X->YZ, X->YWZ */.
  compute modelmat=
  {1,0,0,0,0,0,0,1,0,0;2,0,0,0,0,0,0,1,1,0;3,0,0,0,0,0,0,1,1,1;4,0,0,0,0,0,0,0,0,0;
  5,0,0,0,0,0,0,1,0,0;6,0,0,0,0,0,0,0,0,0;7,1,0,0,0,0,0,0,0,0;8,1,0,0,0,0,0,1,0,0;
  9,1,1,0,0,0,0,0,0,0;10,1,1,0,0,0,0,1,1,0;11,1,1,1,0,0,0,0,0,0;12,1,1,1,0,0,0,1,1,1;
  13,1,1,1,0,0,0,1,0,0;14,0,0,0,1,0,0,0,0,0;15,0,0,0,1,0,0,1,0,0;16,0,0,0,1,1,0,0,0,0;
  17,0,0,0,1,1,0,1,1,0;18,0,0,0,1,1,1,0,0,0;19,0,0,0,1,1,1,1,1,1;20,0,0,0,1,1,1,1,0,0;
  21,1,0,0,0,1,0,0,0,0;22,1,0,0,0,1,0,1,0,0;23,0,0,0,0,0,0,0,0,0;24,0,0,0,0,0,0,0,0,0;
  25,0,0,0,0,0,0,0,0,0;26,0,0,0,0,0,0,0,0,0;27,0,0,0,0,0,0,0,0,0;28,1,0,0,0,1,0,0,1,0;
  29,1,0,0,0,1,0,1,1,0;30,0,0,0,0,0,0,0,0,0;31,0,0,0,0,0,0,0,0,0;32,0,0,0,0,0,0,0,0,0;
  33,0,0,0,0,0,0,0,0,0;34,0,0,0,0,0,0,0,0,0;35,0,0,0,0,0,0,0,0,0;36,0,0,0,0,0,0,0,0,0;
  37,0,0,0,0,0,0,0,0,0;38,0,0,0,0,0,0,0,0,0;39,0,0,0,0,0,0,0,0,0;40,0,0,0,0,0,0,0,0,0;
  41,0,0,0,0,0,0,0,0,0;42,0,0,0,0,0,0,0,0,0;43,0,0,0,0,0,0,0,0,0;44,0,0,0,0,0,0,0,0,0;
  45,0,0,0,0,0,0,0,0,0;46,0,0,0,0,0,0,0,0,0;47,0,0,0,0,0,0,0,0,0;48,0,0,0,0,0,0,0,0,0;
```

```
49,0,0,0,0,0,0,0,0,0;50,0,0,0,0,0,0,0,0,0,0;51,0,0,0,0,0,0,0,0,0,0;52,0,0,0,0,0,0,0,0,0,0;
53,0,0,0,0,0,0,0,0,0;54,0,0,0,0,0,0,0,0,0,0;55,0,0,0,0,0,0,0,0,0,0;56,0,0,0,0,0,0,0,0,0,0;
57,0,0,0,0,0,0,0,0,0;58,1,0,0,1,0,0,0,0,0;59,1,0,0,1,0,0,1,0,0;60,1,1,0,1,0,0,0,0,0;
61,1,1,0,1,0,0,1,0,0;62,1,1,0,1,0,0,0,1,0;63,1,1,0,1,0,0,1,1,0;64,1,0,0,1,1,0,0,0,0;
65,1,0,0,1,1,0,1,0,0;66,1,0,0,1,1,0,0,1,0;67,1,0,0,1,1,0,1,1,0;68,1,1,1,1,0,0,0,0,0;
69,1,1,1,1,0,0,1,1,1;70,1,0,0,1,1,1,0,0,0;71,1,0,0,1,1,1,1,1,1;72,1,1,1,1,1,1,0,0,0;
73,1,1,1,1,1,1,1,1,1;74,0,0,0,1,0,0,0,0,0;75,1,1,0,1,1,0,0,0,0;76,1,1,0,1,1,0,1,1,0;
77,0,0,0,0,0,0,0,0,0;78,0,0,0,0,0,0,0,0,0,0;79,0,0,0,0,0,0,0,0,0,0;80,0,0,0,0,0,0,0,0,0,0;
81,0,0,0,0,0,0,0,0,0;82,0,0,0,0,0,0,0,0,0,0;83,1,0,0,0,0,0,0,0,0,0;84,1,0,0,0,0,0,0,0,0,0;
85,1,0,0,0,0,0,1,0,0;86,1,0,0,0,0,0,1,0,0;87,0,0,0,1,0,0,0,0,0;88,0,0,0,1,0,0,0,0,0;
89,0,0,0,1,0,0,1,0,0;90,0,0,0,1,0,0,1,0,0;91,0,0,0,0,0,0,0,0,0,0;92,1,0,0,1,0,0,1,0,0}.
compute tmp=modelmat(model,2:ncol(modelmat)).
do if (model < 4).
  compute bcmat((nxs+1),1)=1.
end if.
do if ((model > 3) and (model <> 6)).
  compute bcmat((nxs+1):(nxs+nms),1)=onem.
  compute bcmat(nrow(bcmat),(nxs+1):(nxs+nms))=t(onem).
  compute bcmat(nrow(bcmat),1)=1.
end if.
/* models 6, 80, 81, and 82 are serial models */.
do if ((model = 6) or (model > 82 and model < 93)).
  loop j = 2 to nrow(bcmat).
    loop i = 1 to (j-1).
      compute bcmat(j,i)=1.
    end loop.
  end loop.
end if.
```

```
do if (model = 80).
  loop i = 1 to nms.
    compute bcmat((nrow(bcmat)-1),i)=1.
  end loop.
end if.
do if (model = 81).
  loop j = 3 to nrow(bcmat).
    compute bcmat(j,2)=1.
  end loop.
end if.
do if (model = 82).
  compute bcmat(3,2)=1.
  compute bcmat(5,4)=1.
end if.
/* set up moderator matrices */.
do if (tmp(1,1)=1).
  compute wcmat((nxs+1):(nxs+nms),1)=onem.
  compute wprod=1.
  compute xprod=1.
  do if (model = 83 or model = 86).
    compute onemsx=onem.
    loop i = 1 to (nms-1).
      compute onemsx(i+1,1)=0.
    end loop.
    compute wcmat((nxs+1):(nxs+nms),1)=onemsx.
  end if.
end if.
do if (tmp(1,4)=1).
```

```
  compute wcmat(nrow(wcmat),(nxs+1):(nxs+nms))=t(onem).
  compute wprod=1.
  do if (model = 87 or model = 90).
    compute onemsx=onem.
    loop i = 1 to (nms-1).
      compute onemsx(i,1)=0.
    end loop.
    compute wcmat(nrow(wcmat),(nxs+1):(nxs+nms))=t(onemsx).
  end if.
end if.
do if (tmp(1,7)=1).
  compute wcmat(nrow(wcmat),1)=1.
  compute wprod=1.
  compute xprod=1.
end if.
do if (tmp(1,2)=1).
  compute zcmat((nxs+1):(nxs+nms),1)=onem.
  compute zprod=1.
  compute xprod=1.
end if.
do if (tmp(1,5)=1).
  compute zcmat(nrow(zcmat),(nxs+1):(nxs+nms))=t(onem).
  compute zprod=1.
end if.
do if (tmp(1,8)=1).
  compute zcmat(nrow(zcmat),1)=1.
  compute zprod=1.
  compute xprod=1.
```

```
end if.
do if (tmp(1,3)=1).
  compute wzcmat((nxs+1):(nxs+nms),1)=onem.
  compute xprod=1.
  compute wprod=1.
  compute zprod=1.
end if.
do if (tmp(1,6)=1).
  compute wzcmat(nrow(wzcmat),(nxs+1):(nxs+nms))=t(onem).
  compute zprod=1.
  compute wprod=1.
end if.
do if (tmp(1,9)=1).
  compute wzcmat(nrow(wzcmat),1)=1.
  compute xprod=1.
  compute wprod=1.
  compute zprod=1.
end if.
do if (model = 91 or model = 92).
  loop j = 1 to (nms-1).
    loop i = 1 to j.
      compute wcmat((nxs+1+j),(nxs+i))=1.
    end loop.
  end loop.
end if.
do if (nms < 0).
loop i = 1 to nms.
```

```
    /* compute
tmp=wcmat(nrow(wcmat),(1+i))+zcmat(nrow(zcmat),(1+i))+wzcmat(nrow(wzcmat),(1+i)) */.
    compute tmp=csum(wcmat(:,(1+i)))+csum(zcmat(:,(1+i)))+csum(wzcmat(:,(1+i))).
    compute mprod(1,i)=(tmp>0).
  end loop.
  end if.
end if.

/* DEFINE AND CHECK COVARIATES MATRIX */.
do if (ncs > 0).
  compute ccmat=make((nms+nys),ncs,1).
  do if (covmy=1).
    compute ccmat(nrow(ccmat),:)=make(1,ncs,0).
  end if.
  do if (covmy=2).
    compute ccmat(1:nms,:)=make(nms,ncs,0).
  end if.
  do if (cmatrix(1,1) <> -999).
    do if (ncol(cmatrix) <> ((nms+nys)*ncs)).
      compute errcode(errs,1)=29.
      compute errs=errs+1.
      compute criterr=1.
    end if.
    do if (criterr = 0).
      compute tmp=1.
      loop i = 1 to (nms+nys).
        loop j = 1 to ncs.
          compute ccmat(i,j)=1-(cmatrix(1,tmp) = 0).
```

```
        compute tmp=tmp+1.
      end loop.
    end loop.
    do if (rsum((csum(ccmat)=0)) <> 0).
      compute errcode(errs,1)=30.
      compute errs=errs+1.
      compute criterr=1.
    end if.
  end if.
  do if (covmy <> 0).
    compute notecode(notes,1)=1.
    compute notes=notes+1.
  end if.
 end if.
end if.

/* DEFINE MATRICES FOR CUSTOM MODELS AND DO SOME ERROR CHECKING */.
do if (criterr=0).
 compute needed=needed*(needed-1)/2.
 compute nopath=0.
 do if (bmatrix(1,1) <> -999).
  compute tmp=1.
  do if ((ncol(bmatrix) <> needed) or (csum(rsum(bmatrix))=0)).
    compute errcode(errs,1)=16.
    compute errs=errs+1.
    compute criterr=1.
  else.
    loop i = 2 to nrow(bcmat).
```

```
    loop j = 1 to (i-1).
      compute bcmat(i,j)=1-(bmatrix(1,tmp) = 0).
      compute tmp=tmp+1.
    end loop.
  end loop.
end if.
/* check to make sure X affects something */.
do if ((csum(bcmat(:,1))=0) and criterr=0).
  compute errcode(errs,1)=22.
  compute errs=errs+1.
  compute criterr=1.
end if.
/* check to make sure Y is affected by something */.
do if ((rsum(bcmat(nrow(bcmat),:))=0) and criterr=0).
  compute errcode(errs,1)=23.
  compute errs=errs+1.
  compute criterr=1.
end if.
/* check for dangling mediators */.
compute dm=0.
do if (nms > 0).
  loop i = 1 to nms.
    do if (((rsum(bcmat((nxs+i),:)) = 0) or (csum(bcmat(:,(nxs+i))) = 0)) and (dm=0) and (criterr=0)).
      compute errcode(errs,1)=26.
      compute errs=errs+1.
      compute criterr=1.
      compute dm=1.
    end if.
```

```
      end loop.
    end if.
    release dm.
  end if.
end if.

/* start b */.
do if (criterr=0).
  do if (wmatrix(1,1) <> -999).
    compute tmp=1.
    do if (ncol(wmatrix) <> needed).
      compute errcode(errs,1)=17.
      compute errs=errs+1.
      compute criterr=1.
    else.
      compute modelvar(1,1)="CUSTOM".
      loop i = 2 to nrow(wcmat).
        loop j = 1 to (i-1).
          compute wcmat(i,j)=1-(wmatrix(1,tmp) = 0).
          /* dont allow to specify moderation of a path that doesnt exist */.
          do if ((wcmat(i,j)=1) and (bcmat(i,j)=0) and (nopath=0)).
            compute errcode(errs,1)=20.
            compute errs=errs+1.
            compute criterr=1.
            compute nopath=1.
          end if.
          compute tmp=tmp+1.
        end loop.
```

```
    end loop.
  end if.
end if.
do if (zmatrix(1,1) <> -999).
  compute tmp=1.
  do if (ncol(zmatrix) <> needed).
    compute errcode(errs,1)=18.
    compute errs=errs+1.
    compute criterr=1.
  else.
    compute modelvar(1,1)="CUSTOM".
    /* dont allow for a ZMODEL without W being used somewhere in the model */.
    do if (csum(rsum(wcmat))=0 and model=999).
      compute errcode(errs,1)=21.
      compute errs=errs+1.
      compute criterr=1.
    end if.
    loop i = 2 to nrow(zcmat).
      loop j = 1 to (i-1).
        compute zcmat(i,j)=1-(zmatrix(1,tmp) = 0).
        /* dont allow to specify moderation of a path that doesnt exist */.
        do if ((zcmat(i,j)=1) and (bcmat(i,j)=0) and (nopath=0)).
          compute errcode(errs,1)=20.
          compute errs=errs+1.
          compute criterr=1.
          compute nopath=1.
        end if.
        compute tmp=tmp+1.
```

```
      end loop.
    end loop.
  end if.
end if.
compute tmp=1.

do if (wzmatrix(1,1) <> -999).
  do if (ncol(wzmatrix) <> needed).
    compute errcode(errs,1)=19.
    compute errs=errs+1.
    compute criterr=1.
  end if.
  compute modelvar(1,1)="CUSTOM".
end if.
do if (criterr=0).
  loop i = 2 to nrow(wzcmat).
    loop j = 1 to (i-1).
      /* set corresponding elements in W and Z for three way interaction */.
      do if (wzmatrix(1,1) <> -999).
        compute wzcmat(i,j)=1-(wzmatrix(1,tmp) = 0).
      end if.
      do if (wzcmat(i,j)=1).
        compute wcmat(i,j)=1.
        compute zcmat(i,j)=1.
      end if.
      /* dont allow to specify moderation of a path that doesnt exist */.
      do if ((wzcmat(i,j)=1) and (bcmat(i,j)=0) and (nopath=0)).
        compute errcode(errs,1)=20.
```

```
          compute errs=errs+1.
          compute criterr=1.
          compute nopath=1.
        end if.
        compute tmp=tmp+1.
      end loop.
    end loop.
  end if.
end if.
/* end B */.

do if (criterr=0).
  compute xprod=csum(wcmat(:,1))+csum(zcmat(:,1))+csum(wzcmat(:,1)).
  compute xprod=(xprod > 0).
  compute wsum=csum(rsum(wcmat)).
  compute wprod=(wsum > 0).
  do if (nms > 0).
    loop i = 1 to nms.
      /* compute
tmp=wcmat(nrow(wcmat),(1+i))+zcmat(nrow(zcmat),(1+i))+wzcmat(nrow(wzcmat),(1+i)) */.
      compute tmp=csum(wcmat(:,(1+i)))+csum(zcmat(:,(1+i)))+csum(wzcmat(:,(1+i))).
      compute mprod(1,i)=(tmp>0).
    end loop.
  end if.
  do if ((wsum > 0) and (w = "xxxxx")).
    compute errcode(errs,1)=11.
    compute errs=errs+1.
    compute criterr=1.
```

```
  end if.
  do if ((wsum = 0) and (w <> "xxxxx")).
    compute errcode(errs,1)=10.
    compute errs=errs+1.
    compute criterr=1.
  end if.
  compute zsum=csum(rsum(zcmat)).
  compute zprod=(zsum > 0).
  do if ((zsum > 0) and (z = "xxxxx")).
    compute errcode(errs,1)=13.
    compute errs=errs+1.
    compute criterr=1.
  end if.
  do if ((zsum = 0) and (z <> "xxxxx")).
    compute errcode(errs,1)=12.
    compute errs=errs+1.
    compute criterr=1.
  end if.
  do if ((zsum > 0) and (wsum = 0)).
    compute errcode(errs,1)=35.
    compute errs=errs+1.
    compute criterr=1.
  end if.
end if.

do if (criterr=0 and nms > 1).
  compute serchk=bcmat(2:(nrow(bcmat)-1),2:ncol(bcmat)).
  do if (csum(rsum(serchk))) > 0.
```

```
    compute serial=1.
    do if (nms > 6).
      compute errcode(errs,1)=36.
      compute errs=errs+1.
      compute criterr=1.
    end if.
  end if.
end if.

/* MEAN CENTER IF NEEDED */.
do if (center = 1 and criterr=0).
  compute centvar={" "}.
  do if (criterr=0).
    do if (wprod=1 and mcwok=0 and nwpval > 0).
      loop i = 1 to nws.
        compute wtmp(:,i)=wtmp(:,i)-(csum(wtmp(:,i))/n).
        compute centvar={centvar,wnames(1,i)}.
      end loop.
      describe descdatf=wtmp/type=wmodcust.
      compute wmin=desctmp(3,1).
      compute wmax=desctmp(4,1).
      do if (wmodcust=0).
        compute wmodvals=modvals.
        compute wprobval=wmodvals.
      end if.
    end if.
    do if (zprod=1 and mczok=0 and nzpval > 0).
      loop i = 1 to nzs.
```

```
    compute ztmp(:,i)=ztmp(:,i)-(csum(ztmp(:,i))/n).
    compute centvar={centvar,znames(1,i)}.
  end loop.
  describe descdatf=ztmp/type=zmodcust.
  compute zmin=desctmp(3,1).
  compute zmax=desctmp(4,1).
  do if (zmodcust=0).
    compute zmodvals=modvals.
    compute zprobval=zmodvals.
  end if.
end if.
do if (xprod=1 and mcxok=0).
  loop i = 1 to nxs.
    compute xtmp(:,i)=xtmp(:,i)-(csum(xtmp(:,i))/n).
    compute centvar={centvar,xnames(1,i)}.
  end loop.
  describe descdatf=xtmp.
  compute xmodvals=modvals.
  compute xprobval=xmodvals.
end if.
do if (nms > 0).
  loop i = 1 to nms.
    do if (mprod(1,i)=1).
      compute mtmp(:,i)=mtmp(:,i)-(csum(mtmp(:,i))/n).
      compute centvar={centvar,mnames(1,i)}.
    end if.
  end loop.
describe descdatf=mtmp.
```

```
    compute mmodvals=modvals.
    compute mprobval=mmodvals.
    end if.
  end if.
  do if (ncol(centvar) > 1).
    /* compute centvar=centvar(1,2:ncol(centvar)) */.
    compute notecode(notes,1)=3.
    compute notes=notes+1.
  end if.
end if.

/* start D */.
/* CONSTRUCT THE DATA MATRICES FOR EACH OF THE MODELS */.
do if (criterr=0).
  /* The i loop is the dependent variable in the model matrices */.
  /* The j loop is the predictor variables in the model matrices */.
  compute wsum=rsum(csum(wcmat)).
  compute zsum=rsum(csum(zcmat)).
  compute wzsum=rsum(csum(wzcmat)).
  compute nump=make(1,(nys+nms),-999).
  compue numint=make(1,(nys+nms),0).
  /* DV */.
  compute datcount=1.
  compute xtmpuse=0.
  compute wtmpuse=0.
  compute ztmpuse=0.
  compute xwtmpus=0.
  compute xztmpus=0.
```

```
compute wztmpus=0.
compute xwztmpu=0.
compute xtmploc=-999.
compute wtmploc=-999.
compute xwtmplo=-999.
compute ztmploc=-999.
compute xztmplo=-999.
compute wztmplo=-999.
compute xwztmplo=-999.
compute vlabs={" "}.
do if (ncs > 0).
  compute ctmpuse=make(1,ncs,0).
end if.
do if (nms > 0).
  compute mtmpuse=make(1,nms,0).
  compute mwtmpus=make(1,nms,0).
  compute mztmpus=make(1,nms,0).
  compute mwztmpu=make(1,nms,0).
  compute mtmploc=make(1,nms,0).
  compute mwtmplo=make(nwvls,nms,-999).
  compute mztmplo=make(nzvls,nms,-999).
  compute mwztmplo=make((nwvls*nzvls),nms,-999).
end if.
do if (ncs > 0).
  compute ctmploc=make(1,ncs,0).
end if.
compute fulldat=make(n,1,1).
compute datindx=make(1000,(nms+nys),-999).
```

```
compute wherew=make(2,(nms+nys),-999).
compute wherex=make(2,(nms+nys),-999).
compute wherez=make(2,(nms+nys),-999).
compute wherexw=make(2,(nms+nys),-999).
compute wherexz=make(2,(nms+nys),-999).
compute wherewz=make(2,(nms+nys),-999).
compute wherexwz=make(2,(nms+nys),-999).
do if (nms > 0).
  compute wherem=make(nms,(nms+nys),-999).
  compute wheremw = make(nms*2,(nms+nys),-999).
  compute wheremz = make(nms*2,(nms+nys),-999).
  compute wheremwz = make(nms*2,(nms+nys),-999).
end if.
compute wzhigh=make(1000,(((nms+1)*(nms+2))/2),0).
compute whigh=make(1000,(((nms+1)*(nms+2))/2),0).
compute zhigh=make(1000,(((nms+1)*(nms+2))/2),0).
compute fochigh=make(1000,(((nms+1)*(nms+2))/2),0).
compute xcoefloc={1;2;3;4;5;6;7;8;9}.
compute intkey = {" ", " ", " ", " ", " ", " ", " "}.
compute wzhighct=0.
compute whighct=0.
compute zhighct=0.
compute foccnt=0.
loop i = 2 to nrow(bcmat).
  compute wdid=0.
  compute zdid=0.
  compute wzdid=0.
  compute cntmp=1.
```

```
compute start=1.
do if (i < nrow(bcmat)).
  compute outv=mtmp(:,(i-1)).
  compute modlabel={mnames(1,(i-1));"constant"}.
end if.
do if (i = nrow(bcmat)).
  compute outv=ytmp.
  compute modlabel={ynames;"constant"}.
end if.
/* The j loop is the mediator */.
/* MED */.
/* do if (nms > 0) */.
loop j = 1 to (i-1).
  compute foccnt=foccnt+1.
  do if (j = 1 and bcmat(i,j)=1).
    compute outv={outv,xtmp}.
    compute modlabel={modlabel;xcatlab(1:nxvls,1)}.
    do if (xtmpuse=0).
      compute fulldat={fulldat,xtmp}.
      compute xtmpuse=1.
      loop k4=datcount to (datcount+(nxvls-1)).
        compute xtmploc={xtmploc;k4}.
      end loop.
      compute xtmploc=xtmploc(2:nrow(xtmploc),1).
      compute datcount=datcount+nxvls.
    end if.
    compute datindx(start:(start+nrow(xtmploc)-1),(i-1))=xtmploc.
    compute wherex(1,(i-1))=start+1.
```

```
    compute wherex(2,(i-1))=start+nrow(xtmploc)-1+1.
    compute onebl=make(nrow(xtmploc),1,1).
    compute fochigh((start+1):(start+nrow(xtmploc)),foccnt)=onebl.
    compute start=start+nrow(xtmploc).
  end if.
  do if (j > 1 and bcmat(i,j)=1).
    compute outv={outv,mtmp(:,(j-1))}.
    compute modlabel={modlabel;mnames(1,(j-1))}.
    do if (mtmpuse(1,(j-1))=0).
      compute fulldat={fulldat,mtmp(:,(j-1))}.
      compute mtmpuse(1,(j-1))=1.
      compute mtmploc(1,(j-1))=datcount.
      compute datcount=datcount+1.
    end if.
    compute datindx(start:(start+nrow(mtmploc)-1),(i-1))=mtmploc(1,(j-1)).
    compute wherem((j-1),(i-1))=start+1.
    compute onebl=make(nrow(mtmploc(1,j-1)),1,1).
    compute ttt=nrow(mtmploc(1,(j-1)))+start-1.
    compute fochigh((start+1):(start+nrow(mtmploc(1,(j-1)))),foccnt)=onebl.
    compute start=start+nrow(mtmploc(1,(j-1))).
  end if.
end loop.
/* end if */.
/* END MED */.
/* This starts the W loop */.
/* W */.
do if (wsum > 0).
  loop j = 1 to (i-1).
```

```
compute whighct=whighct+1.
 do if (j = 1 and wcmat(i,j)=1).
  do if (wdid=0).
   compute outv={outv,wtmp}.
   compute modlabel={modlabel;wcatlab(1:nwvls,1)}.
   compute wdid=1.
   do if (wtmpuse=0).
    compute fulldat={fulldat,wtmp}.
    compute wtmpuse=1.
    loop k4=datcount to (datcount+(nwvls-1)).
     compute wtmploc={wtmploc;k4}.
    end loop.
    compute wtmploc=wtmploc(2:nrow(wtmploc),1).
    compute datcount=datcount+nwvls.
   end if.
  end if.
  compute datindx(start:(start+nrow(wtmploc)-1),(i-1))=wtmploc.
  compute wherew(1,(i-1))=start+1.
  compute wherew(2,(i-1))=start+nrow(wtmploc)-1+1.
  compute start=start+nrow(wtmploc).
  loop k1=1 to nxvls.
   loop k2 = 1 to nwvls.
    compute outv={outv,(xtmp(:,k1)&*wtmp(:,k2))}.
    compute modlabel={modlabel;intlab(cntmp,1)}.
    compute intkey={intkey;intlab(cntmp,1),":",xcatlab(k1,1),"x",wcatlab(k2,1)," "," "}.
    compute cntmp=cntmp+1.
   end loop.
  end loop.
```

```
  do if (xwtmpus=0).
    compute fulldat={fulldat,outv(:,(ncol(outv)-(nxvls*nwvls)+1):ncol(outv))}.
    compute xwtmpus=1.
    loop k4=datcount to (datcount+((nwvls*nxvls)-1)).
      compute xwtmplo={xwtmplo;k4}.
    end loop.
    compute xwtmplo=xwtmplo(2:nrow(xwtmplo),1).
    compute datcount=datcount+(nxvls*nwvls).
  end if.
  compute datindx(start:(start+nrow(xwtmplo)-1),(i-1))=xwtmplo.
  compute wherexw(1,(i-1))=start+1.
  compute wherexw(2,(i-1))=start+nrow(xwtmplo)-1+1.
  compute onebl=make(nrow(xwtmplo),1,1).
  compute whigh((start+1):(start+nrow(xwtmplo)),whighct)=onebl.
  compute start=start+nrow(xwtmplo).
end if.
do if (j > 1 and wcmat(i,j)=1).
  do if (wdid=0 and model <> 74).
    compute outv={outv,wtmp}.
    compute modlabel={modlabel;wcatlab(1:nwvls,1)}.
    compute wdid=1.
    do if (wtmpuse=0).
      compute fulldat={fulldat,wtmp}.
      compute wtmpuse=1.
      loop k4=datcount to (datcount+(nwvls-1)).
        compute wtmploc={wtmploc;k4}.
      end loop.
      compute wtmploc=wtmploc(2:nrow(wtmploc),1).
```

```
    compute datcount=datcount+nwvls.
  end if.
  compute datindx(start:(start+nrow(wtmploc)-1),(i-1))=wtmploc.
  compute wherew(1,(i-1))=start+1.
  compute wherew(2,(i-1))=start+nrow(wtmploc)-1+1.
  compute start=start+nrow(wtmploc).
end if.
loop k2 = 1 to nwvls.
  compute outv={outv,(mtmp(:,(j-1))&*wtmp(:,k2))}.
  compute modlabel={modlabel;intlab(cntmp,1)}.
  compute intkey={intkey;intlab(cntmp,1),":", mnames(1,(j-1)),"x",wcatlab(k2,1)," "," "}.
  compute cntmp=cntmp+1.
end loop.
do if (mwtmpus(1,(j-1))=0).
  compute fulldat={fulldat,outv(:,(ncol(outv)-nwvls+1):ncol(outv))}.
  compute mwtmpus(1,(j-1))=1.
  compute mw22=-999.
  loop k4=datcount to (datcount+(nwvls-1)).
    compute mw22={mw22;k4}.
  end loop.
  compute mwtmplo(:,(j-1))=mw22(2:nrow(mw22),1).
  compute datcount=datcount+nwvls.
end if.
compute datindx(start:(start+nrow(mwtmplo)-1),(i-1))=mwtmplo(:,(j-1)).
compute wheremw(((2*j)-3),(i-1))=start+1.
compute wheremw(((2*j)-2),(i-1))=start+nrow(mwtmplo)-1+1.
compute onebl=make(nrow(mwtmplo),1,1).
compute whigh((start+1):(start+nrow(mwtmplo)),whighct)=onebl.
```

```
      compute start=start+nrow(mwtmplo).
    end if.
  end loop.
end if.
/* END W */.
/* This starts the Z loop */.
/* Z */.
do if (zsum > 0).
  loop j = 1 to (i-1).
    compute zhighct=zhighct+1.
    do if (j = 1 and zcmat(i,j)=1).
      do if (zdid=0).
        compute outv={outv,ztmp}.
        compute modlabel={modlabel;zcatlab(1:nzvls,1)}.
        compute zdid=1.
        do if (ztmpuse=0).
          compute fulldat={fulldat,ztmp}.
          compute ztmpuse=1.
          loop k4=datcount to (datcount+(nzvls-1)).
            compute ztmploc={ztmploc;k4}.
          end loop.
          compute ztmploc=ztmploc(2:nrow(ztmploc),1).
          compute datcount=datcount+nzvls.
        end if.
      end if.
      compute datindx(start:(start+nrow(ztmploc)-1),(i-1))=ztmploc.
      compute wherez(1,(i-1))=start+1.
      compute wherez(2,(i-1))=start+nrow(ztmploc)-1+1.
```

```
  compute start=start+nrow(ztmploc).
  loop k1=1 to nxvls.
    loop k2 = 1 to nzvls.
      compute outv={outv,(xtmp(:,k1)&*ztmp(:,k2))}.
      compute modlabel={modlabel;intlab(cntmp,1)}.
      compute intkey={intkey;intlab(cntmp,1),":",xcatlab(k1,1),"x",zcatlab(k2,1)," "," "}.
      compute cntmp=cntmp+1.
    end loop.
  end loop.
  do if (xztmpus=0).
    compute fulldat={fulldat,outv(:,(ncol(outv)-(nxvls*nzvls)+1):ncol(outv))}.
    compute xztmpus=1.
    loop k4=datcount to (datcount+((nzvls*nxvls)-1)).
      compute xztmplo={xztmplo;k4}.
    end loop.
    compute xztmplo=xztmplo(2:nrow(xztmplo),1).
    compute datcount=datcount+(nxvls*nzvls).
  end if.
  compute datindx(start:(start+nrow(xztmplo)-1),(i-1))=xztmplo.
  compute wherexz(1,(i-1))=start+1.
  compute wherexz(2,(i-1))=start+nrow(xztmplo)-1+1.
  compute onebl=make(nrow(xztmplo),1,1).
  compute zhigh((start+1):(start+nrow(xztmplo)),zhighct)=onebl.
  compute start=start+nrow(xztmplo).
end if.
do if (j > 1 and zcmat(i,j)=1).
  do if (zdid=0).
    compute outv={outv,ztmp}.
```

```
  compute modlabel={modlabel;zcatlab(1:nzvls,1)}.
  compute zdid=1.
  do if (ztmpuse=0).
    compute fulldat={fulldat,ztmp}.
    compute ztmpuse=1.
    loop k4=datcount to (datcount+(nzvls-1)).
      compute ztmploc={ztmploc;k4}.
    end loop.
    compute ztmploc=ztmploc(2:nrow(ztmploc),1).
    compute datcount=datcount+nzvls.
  end if.
  compute datindx(start:(start+nrow(ztmploc)-1),(i-1))=ztmploc.
  compute wherez(1,(i-1))=start+1.
  compute wherez(2,(i-1))=start+nrow(ztmploc)-1+1.
  compute start=start+nrow(ztmploc).
end if.
loop k2 = 1 to nzvls.
  compute outv={outv,(mtmp(:,(j-1))&*ztmp(:,k2))}.
  compute modlabel={modlabel;intlab(cntmp,1)}.
  compute intkey={intkey;intlab(cntmp,1),":", mnames(1,(j-1)),"x",zcatlab(k2,1)," "," "}.
  compute cntmp=cntmp+1.
end loop.
do if (mztmpus(1,(j-1))=0).
  compute fulldat={fulldat,outv(:,(ncol(outv)-nzvls+1):ncol(outv))}.
  compute mztmpus(1,(j-1))=1.
  compute mz22=-999.
  loop k4=datcount to (datcount+(nzvls-1)).
    compute mz22={mz22;k4}.
```

```
        end loop.
        compute mztmplo(:,(j-1))=mz22(2:nrow(mz22),1).
        compute datcount=datcount+nzvls.
      end if.
      compute datindx(start:(start+nrow(mztmplo)-1),(i-1))=mztmplo(:,(j-1)).
      compute wheremz(((2*j)-3),(i-1))=start+1.
      compute wheremz(((2*j)-2),(i-1))=start+nrow(mztmplo)-1+1.
      compute onebl=make(nrow(mztmplo),1,1).
      compute zhigh((start+1):(start+nrow(mztmplo)),zhighct)=onebl.
      compute start=start+nrow(mztmplo).
    end if.
  end loop.
end if.
/* END Z */.
/* This starts the WZ loop */.
/* WZ */.
do if (wzsum > 0).
  loop j = 1 to (i-1).
    compute wzhighct=wzhighct+1.
    do if (j = 1 and wzcmat(i,j)=1).
      do if (wzdid=0).
        loop k1=1 to nwvls.
          loop k2 = 1 to nzvls.
            compute outv={outv,(wtmp(:,k1)&*ztmp(:,k2))}.
            compute modlabel={modlabel;intlab(cntmp,1)}.
            compute intkey={intkey;intlab(cntmp,1),":",wcatlab(k1,1),"x",zcatlab(k2,1)," "," "}.
            compute cntmp=cntmp+1.
          end loop.
```

```
  end loop.
  do if (wztmpus=0).
    compute fulldat={fulldat,outv(:,(ncol(outv)-(nwvls*nzvls)+1):ncol(outv))}.
    compute wztmpus=1.
    loop k4=datcount to (datcount+((nwvls*nzvls)-1)).
      compute wztmplo={wztmplo;k4}.
    end loop.
    compute wztmplo=wztmplo(2:nrow(wztmplo),1).
    compute datcount=datcount+(nzvls*nwvls).
  end if.
  compute wzdid=1.
end if.
compute datindx(start:(start+nrow(wztmplo)-1),(i-1))=wztmplo.
compute wherewz(1,(i-1))=start+1.
compute wherewz(2,(i-1))=start+nrow(wztmplo)-1+1.
compute start=start+nrow(wztmplo).
loop k1=1 to nxvls.
  loop k2=1 to nwvls.
    loop k3=1 to nzvls.
      compute outv={outv,(xtmp(:,k1)&*wtmp(:,k2)&*ztmp(:,k3))}.
      compute modlabel={modlabel;intlab(cntmp,1)}.
      compute
intkey={intkey;intlab(cntmp,1),":",xcatlab(k1,1),"x",wcatlab(k2,1),"x",zcatlab(k3,1)}.
      compute cntmp=cntmp+1.
    end loop.
  end loop.
end loop.
do if (xwztmpu=0).
```

```
    compute fulldat={fulldat,outv(:,(ncol(outv)-(nxvls*nwvls*nzvls)+1):ncol(outv))}.
    compute xwztmpu=1.
    loop k4=datcount to (datcount+((nzvls*nxvls*nwvls)-1)).
      compute xwztmplo={xwztmplo;k4}.
    end loop.
    compute xwztmplo=xwztmplo(2:nrow(xwztmplo),1).
    compute datcount=datcount+(nxvls*nzvls*nwvls).
  end if.
  compute datindx(start:(start+nrow(xwztmplo)-1),(i-1))=xwztmplo.
  compute wherexwz(1,(i-1))=start+1.
  compute wherexwz(2,(i-1))=start+nrow(xwztmplo)-1+1.
  compute onebl=make(nrow(xwztmplo),1,1).
  compute wzhigh((start+1):(start+nrow(xwztmplo)),wzhighct)=onebl.
  compute start=start+nrow(xwztmplo).
end if.
do if (j > 1 and wzcmat(i,j)=1).
  do if (wzdid=0).
    loop k1=1 to nwvls.
      loop k2 = 1 to nzvls.
        compute outv={outv,(wtmp(:,k1)&*ztmp(:,k2))}.
        compute modlabel={modlabel;intlab(cntmp,1)}.
        compute intkey={intkey;intlab(cntmp,1),":",wcatlab(k1,1),"x",zcatlab(k2,1)," "," "}.
        compute cntmp=cntmp+1.
      end loop.
    end loop.
    do if (wztmpus=0).
      compute fulldat={fulldat,outv(:,(ncol(outv)-(nwvls*nzvls)+1):ncol(outv))}.
      compute wztmpus=1.
```

```
    loop k4=datcount to (datcount+((nwvls*nzvls)-1)).
      compute wztmplo={wztmplo;k4}.
    end loop.
    compute wztmplo=wztmplo(2:nrow(wztmplo),1).
    compute datcount=datcount+(nzvls*nwvls).
  end if.
  compute wzdid=1.
  compute datindx(start:(start+nrow(wztmplo)-1),(i-1))=wztmplo.
  compute wherewz(1,(i-1))=start+1.
  compute wherewz(2,(i-1))=start+nrow(wztmplo)-1+1.
  compute start=start+nrow(wztmplo).
end if.
loop k1 = 1 to nwvls.
  loop k2 = 1 to nzvls.
    compute outv={outv,(mtmp(:,(j-1))&*wtmp(:,k1)&*ztmp(:,k2))}.
    compute modlabel={modlabel;intlab(cntmp,1)}.
    compute intkey={intkey;intlab(cntmp,1),":",mnames(1,(j-
1)),"x",wcatlab(k1,1),"x",zcatlab(k2,1)}.
    compute cntmp=cntmp+1.
  end loop.
end loop.
do if (mwztmpu(1,(j-1))=0).
  compute fulldat={fulldat,outv(:,(ncol(outv)-(nwvls*nzvls)+1):ncol(outv))}.
  compute mwztmpu(1,(j-1))=1.
  compute mz22=-999.
  loop k4=datcount to (datcount+(nwvls*nzvls)-1).
    compute mz22={mz22;k4}.
  end loop.
```

```
      compute mwztmplo(:,(j-1))=mz22(2:nrow(mz22),1).
      compute datcount=datcount+(nwvls*nzvls).
    end if.
    compute datindx(start:(start+nrow(mwztmplo)-1),(i-1))=mwztmplo(:,(j-1)).
    compute wheremwz(((2*j)-3),(i-1))=start+1.
    compute wheremwz(((2*j)-2),(i-1))=start+nrow(mwztmplo)-1+1.
    compute onebl=make(nrow(mwztmplo),1,1).
    compute wzhigh((start+1):(start+nrow(mwztmplo)),wzhighct)=onebl.
    compute start=start+nrow(mwztmplo).
  end if.
 end loop.
end if.
/* END WZ */.
/* This starts the COV loop */.
/* COV */.
do if (ncs > 0).
 loop j = 1 to ncs.
  do if (ccmat((i-1),j))=1.
     compute outv={outv,ctmp(:,j)}.
     compute modlabel={modlabel;covnames(1,j)}.
     do if (ctmpuse(1,j)=0).
       compute fulldat={fulldat,ctmp(:,j)}.
       compute ctmpuse(1,j)=1.
       compute ctmploc(1,j)=datcount.
       compute datcount=datcount+1.
     end if.
     compute datindx(start:(start+nrow(ctmploc)-1),(i-1))=ctmploc(1,j).
     compute start=start+nrow(ctmploc(1,j)).
```

```
      end if.
    end loop.
  end if.
  /* END COV */.
  compute wdid=0.
  compute zdid=0.
  compute wzdid=0.
  compute vlabs={vlabs;modlabel(2:nrow(modlabel),1)}.
  compute numint(1,(i-1))=cntmp-1.
  compute nump(1,(i-1))=nrow(modlabel)-1.
end loop.
/* END DV*/.
release datcount, xtmpuse, wtmpuse, ztmpuse, xwtmpus, xztmpus, wztmpus, xwztmpu.
release xtmploc, wtmploc, xwtmplo, ztmploc, xztmplo, wztmplo, xwztmplo, foccnt.
do if (modcok=1 and ((nms > 0) or (zcmat(2,1) <> 1) or (mcx <> 0))).
  compute notecode(notes,1) = 19.
  compute notes = notes + 1.
  compute modcok=0.
end if.
do if ((serial = 1 or (rsum(numint)>0) or nms=0) and mc > 0).
  compute notecode(notes,1) = 15.
  compute notes = notes + 1.
  compute boot=mc.
  compute mc=0.
end if.
do if (boot <> 0 or mc <> 0).
  compute bootsz=boot.
  do if (mc > 0).
```

```
    compute bootsz=mc.
    compute saveboot=0.
  end if.
  loop.
    compute cilow = rnd(bootsz*(1-(conf/100))/2).
    compute cihigh = trunc((bootsz*(conf/100)+(bootsz*(1-(conf/100))/2)))+1.
    do if (cilow < 1 or cihigh > bootsz).
      compute bootsz=trunc((bootsz+1000)/1000)*1000.
      compute adjust = 1.
    end if.
  end loop if (cilow gt 0 and cihigh le bootsz).
  do if (boot > 0).
    compute boot=bootsz.
  end if.
  do if (mc > 0).
    compute mc=bootsz.
  end if.
  do if (adjust = 1 and boot > 0).
    compute notecode(notes,1) = 8.
    compute notes = notes + 1.
  end if.
  do if (adjust = 1 and mc > 0).
    compute notecode(notes,1) = 16.
    compute notes = notes + 1.
  end if.
end if.
compute maxboot = trunc(2*boot).
do if (!maxboot > maxboot).
```

```
  compute maxboot=trunc(!maxboot).
end if.
do if (nms > 0).
  release mtmpuse, mwtmpus, mwztmpu, mtmploc, mwtmplo, mztmplo, mwztmplo.
end if.
release wdid, zdid, wzdid, start,modlabel.
compute vlabs=vlabs(2:nrow(vlabs),1).
do if (rsum(numint) > 0).
  compute intkey=intkey(2:nrow(intkey),:).
end if.
compute fulldat=fulldat(:,2:ncol(fulldat)).
compute fochigh=fochigh(1:rmax(nump),:).
compute whigh=whigh(1:rmax(nump),:).
compute zhigh=zhigh(1:rmax(nump),:).
compute wzhigh=wzhigh(1:rmax(nump),:).
compute coeffs=fochigh+whigh+zhigh+wzhigh.
compute bootloc=make(rmax(nump),ncol(nump),0).
/* here i am deriving the locations in boot file needed for indirect effects */.
do if (nms > 0).
  compute cntmp=1.
  loop i = 1 to ncol(nump).
    loop j = 1 to nump(1,i).
      compute bootloc(j,i)=cntmp.
      compute cntmp=cntmp+1.
    end loop.
  end loop.
  compute fochighb=make(nrow(fochigh),ncol(fochigh),0).
  compute whighb=fochighb.
```

```
compute zhighb=fochighb.
compute wzhighb=fochighb.
compute thetaxmb=make(nrow(fochighb),nms,0).
compute thetaxyb=make(nrow(fochighb),1,0).
compute pathsfoc=make(nxvls,1,0).
compute cntmp=1.
loop i = 1 to (nms+nys).
  loop j = 1 to i.
    compute fochighb(:,cntmp)=fochigh(:,cntmp)&*bootloc(:,i).
    compute whighb(:,cntmp)=whigh(:,cntmp)&*bootloc(:,i).
    compute zhighb(:,cntmp)=zhigh(:,cntmp)&*bootloc(:,i).
    compute wzhighb(:,cntmp)=wzhigh(:,cntmp)&*bootloc(:,i).
    compute coeffsb=fochighb+whighb+zhighb+wzhighb.
    do if ((i < (nms+nys)) and (j = 1)).
      compute thetaxmb(:,i)=coeffsb(:,cntmp).
    end if.
    do if ((i = (nms+nys)) and (j = 1)).
      compute thetaxyb(:,1)=coeffsb(:,cntmp).
    end if.
    compute cntmp=cntmp+1.
  end loop.
end loop.
compute thetamyb=coeffsb(:,(ncol(coeffsb)-nms+1):ncol(coeffsb)).
do if (serial = 1).
  compute thetammb=make(nrow(coeffsb),((nms*(nms-1))/2),0).
end if.
compute cntmp=1.
do if (nms > 1 and serial = 1).
```

```
      loop i = 1 to (nms-1).
        compute start=((i+2)*(i+1))/2.
        loop j = 2 to (nms-i+1).
          compute thetammb(:,cntmp)=coeffsb(:,start).
          compute start=start+j+i-1.
          compute cntmp=cntmp+1.
        end loop.
      end loop.
    end if.
  end if.
  do if ((total = 1) and rsum(numint)=0).
    compute dototal=1.
    do if ((csum(bcmat(:,1)) <> (nms+nys)) or (rsum(bcmat(nrow(bcmat),:)) <> (nms+nys))).
      compute dototal=0.
      compute notecode(notes,1) = 12.
      compute notes = notes + 1.
    end if.
    do if (ncs > 0).
      do if ((csum(rsum(ccmat))) < (nrow(ccmat)*ncol(ccmat))).
        compute dototal=0.
        compute notecode(notes,1) = 11.
        compute notes = notes + 1.
      end if.
    end if.
  end if.
end if.
/* END D */.
```

```
print/title = "**************** PROCESS Procedure for SPSS Version 3.00 *****************".

print/title = "          Written by Andrew F. Hayes, Ph.D.       www.afhayes.com".

print/title = "    Documentation available in Hayes (2018). www.guilford.com/p/hayes3"/space=0.

do if (criterr=0).

  compute funny=1.

  print modelvar/title =
"****************************************************************************"/format
= A8/rnames=modelvlb.

  do if (ncs > 0).

    print covnames/title="Covariates:"/format=A8.

  end if.

  print n/title="Sample"/rlabel="Size:".

  do if ((!quote(!seed) <> "random")).

    compute seedt=!quote(!seed).

    print seedt/title="Custom"/format=A12/rlabel = "Seed:".

  end if.

  do if (mcxok=1).

    compute labtmp={xnames,t(xcatlab(1:nxvls,1))}.

    print dummatx/title = "Coding of categorical X variable for analysis:"/cnames = labtmp/format =
F6.3.

  end if.

  do if (mcwok=1).

    compute labtmp={wnames,t(wcatlab(1:nwvls,1))}.

    print dummatw/title = "Coding of categorical W variable for analysis:"/cnames = labtmp/format =
F6.3.

  end if.

  do if (mczok=1).

    compute labtmp={znames,t(zcatlab(1:nzvls,1))}.

    print dummatz/title = "Coding of categorical Z variable for analysis:"/cnames = labtmp/format =
F6.3.
```

```
  end if.
end if.
/* cycle through the models */.
do if (criterr = 0).
  compute outnames=ynames.
  compute outvars=ytmp.
  do if (nms > 0).
    compute outnames={mnames,ynames}.
    compute outvars={mtmp,ytmp}.
    compute indcov=make(((nms*2)+(nms*(nxvls-1))),((nms*2)+(nms*(nxvls-1))),0).
    compute mcsopath=make(((nms*2)+(nms*(nxvls-1))),1,0).
  end if.
  compute labstart=1.
  compute intstart=1.
  compute start=1.
  compute coeffmat=make(1,6,0).
  compute conseq={"        "}.
  compute dfmat=0.
  compute coeffcol=0.
  compute pathscnt=1.
  compute pathscn2=1.
  /* START G LOOP */.
  loop i = 1 to (nms+nys).
    print/title =
"*************************************************************************".
    compute highf=make(1,5,0).
    compute flabel={" "}.
    compute y=outvars(:,i).
```

```
compute xindx=datindx(1:(nump(1,i)-1),i).
compute x = fulldat(:,xindx).
compute x={ones,x}.
compute xsq=t(x)*x.
compute exsq=eval(xsq).
release xsq.
compute zeroeig=csum(exsq <= 0.000000000002).
print outnames(1,i)/title = "OUTCOME VARIABLE:"/format = A8/space=0.
do if (zeroeig > 0).
  print / title = "SINGULAR OR NEAR SINGULAR DATA MATRIX.".
  compute criterr=1.
  compute errcode(errs,1)=31.
  compute errs=errs+1.
end if.
compute means=csum(x)/n.
compute vlabsm=vlabs(labstart:(labstart+(nump(1,i)-1)),1).
/* type: 1 = ols */.
/* full:  0 = just coefficients, 1 = everything */.
/* START E */.
do if (criterr=0).
  modelest y=y/x=x/type=1/full=1.
  compute obscoeff={obscoeff,t(b)}.
  print modsum/title = "Model Summary"/cnames = modsuml/format=!decimals.
  print modres/title="Model"/rnames=vlabsm/cnames=modresl/format=!decimals.
  compute coeffmat={coeffmat;modres}.
  compute conseqt=make(nrow(modres),1,outnames(1,i)).
  compute conseq={conseq;conseqt}.
  compute dfmatt=make(nrow(modres),1,modsum(1,6)).
```

```
compute dfmat={dfmat;dfmatt}.
compute labstart=labstart+nump(1,i).
do if (nms > 0 and serial = 0 and (rsum(numint) = 0) and (normal=1 or mc > 0)).
  do if (i < (nms+nys)).
    compute indcov((((i-1)*nxvls)+1):(i*nxvls),(((i-
1)*nxvls)+1):(i*nxvls))=varb(2:(1+nxvls),2:(1+nxvls)).
    compute mcsopath((((i-1)*nxvls)+1):(i*nxvls) ,1)=modres(2:(1+nxvls),1).
  end if.
  do if (i = (nms+nys)).
    compute atm=ncol(wherem).
    compute
indcov(((nms*nxvls)+1):nrow(mcsopath),((nms*nxvls)+1):nrow(mcsopath))=varb(wherem(1,atm):(wh
erem(1,atm)+nms-1),wherem(1,atm):(wherem(1,atm)+nms-1)).
    compute
mcsopath(((nms*nxvls)+1):nrow(mcsopath),1)=modres(wherem(1,atm):(wherem(1,atm)+nms-1),1).
    compute sobelok=1.
  end if.
end if.
do if ((i = (nms+nys)) and (bcmat(nrow(bcmat),1)=1)).
  compute direff=modres(2:(1+nxvls),:).
  compute direfflb=modresl.
  compute direffl2=vlabsm(2:(1+nxvls),:).
  compute lmat=make(nrow(b),1,0).
  compute lmat2=make(nxvls,1,1).
  compute lmat(2:(1+nxvls),1)=lmat2.
  ftest3 lm=lmat/bcoef=b/cv=varb/chr=1/brsq=r2.
  compute diromni=fresult.
end if.
do if (numint(1,i) > 0).
  compute intkeym=intkey(intstart:(intstart+numint(1,i)-1),:).
```

```
  print intkeym/title="Product terms key:"/format = A8.
end if.
do if (covcoeff=1).
  print varb/title="Covariance matrix of regression parameter estimates:"/rnames=vlabsm
  /cnames=vlabsm/format=!decimals.
end if.
/* here is where we do F tests */.
/* START F */.
do if (criterr = 0).
  compute jj=0.
  loop j = start to ((start+i)-1).
    compute lmat=whigh(1:nump(1,i),j).
    compute lmat2=wzhigh(1:nump(1,i),j).
    do if ((csum(lmat) > 0) and (csum(lmat2) = 0)).
      ftest3 lm=lmat/bcoef=b/cv=varb/chr=1/brsq=r2.
      compute highf={highf;fresult}.
      do if (j = start).
        compute flabel={flabel;"X*W"}.
      end if.
      do if (j > start).
        do if (nms > 1).
          compute flabel={flabel;highlbw(jj,1)}.
        else if (nms = 1).
          compute flabel={flabel;"M*W"}.
        end if.
      end if.
    end if.
    compute lmat=zhigh(1:nump(1,i),j).
```

```
compute lmat2=wzhigh(1:nump(1,i),j).
do if ((csum(lmat) > 0) and (csum(lmat2) = 0)).
  ftest3 lm=lmat/bcoef=b/cv=varb/chr=1/brsq=r2.
  compute highf={highf;fresult}.
  do if (j = start).
    compute flabel={flabel;"X*Z"}.
  end if.
  do if (j > start).
    do if (nms > 1).
      compute flabel={flabel;highlbz(jj,1)}.
    else if (nms = 1).
      compute flabel={flabel;"M*Z"}.
    end if.
  end if.
end if.
compute lmat2=wzhigh(1:nump(1,i),j).
do if (csum(lmat2) > 0).
  ftest3 lm=lmat2/bcoef=b/cv=varb/chr=1/brsq=r2.
  compute highf={highf;fresult}.
  do if (j = start).
    compute flabel={flabel;"X*W*Z"}.
  end if.
  do if (j > start).
    do if (nms > 1).
      compute flabel={flabel;highlbwz(jj,1)}.
    else if (nms = 1).
      compute flabel={flabel;"M*W*Z"}.
    end if.
```

```
        end if.
       end if.
       compute jj=jj+1.
      end loop.
      release jj.
      compute start=start+i.
     end if.
     /* END F*/.
     do if (nrow(highf) > 1).
      compute highf=highf(2:nrow(highf),:).
      compute flabel=flabel(2:nrow(flabel),1).
      compute clabtmp={"R2-chng", hcflab,"df1","df2","p"}.
      print highf/format = !decimals/rnames=flabel/cnames=clabtmp/
       title = "Test(s) of highest order unconditional interaction(s):".
      compute intpb=highf(:,5).
     end if.
     compute intstart=intstart+numint(1,i).
    end if.
    /* END E*/.
    PROBE decpnt=!decimals.
   end loop if criterr=1.
   /* end G loop */.
   do if (criterr=0 and dototal = 1).
    print/title = "************************** TOTAL EFFECT MODEL
****************************".
    print outnames(1,ncol(outnames))/title = "OUTCOME VARIABLE:"/format = A8/space=0.
    compute x=xtmp.
    compute vlabsm={"constant";xcatlab(1:nxvls,1)}.
```

```
    do if (ncs > 0).
      compute x = {x,ctmp}.
      compute vlabsm={vlabsm;t(covnames)}.
    end if.
    compute x = {ones,x}.
    modelest y=y/x=x/type=1/full=1.
    print modsum/title = "Model Summary"/cnames = modsuml/format=!decimals.
    print modres/title="Model"/rnames=vlabsm/cnames=modresl/format=!decimals.
    compute toteff=modres(2:(1+nxvls),:).
    compute totefflb=modresl.
    compute toteffl2=vlabsm(2:(1+nxvls),:).
    compute lmat=make(nrow(b),1,0).
    compute lmat2=make(nxvls,1,1).
    compute lmat(2:(1+nxvls),1)=lmat2.
    ftest3 lm=lmat/bcoef=b/cv=varb/chr=1/brsq=r2.
    compute totomni=fresult.
    do if (covcoeff=1).
      print varb/title="Covariance matrix of regression parameter estimates:"/rnames=vlabsm
        /cnames=vlabsm/format=!decimals.
    end if.
  end if.
end if.
/* end cycle through the models */.

/* DO BOOTSTRAPPING */.
do if (criterr=0 and boot > 0).
  compute bootres=make(1,rsum(nump),-999).
  do if (effsize=1).
```

```
  compute bootysd=make(1,1,-999).
  compute bootxsd=make(1,1,-999).
end if.
compute badboot=0.
compute goodboot=0.
compute smallest=1.
loop j = 1 to maxboot.
  compute nobootx=1.
  compute modres2=999.
  compute v=trunc(uniform(n,1)*n)+1.
  compute bad=0.
  loop i = 1 to (nms+nys).
    compute y=outvars(v,i).
    compute xindx=datindx(1:(nump(1,i)-1),i).
    compute hello=0.
    compute x = fulldat(v,xindx).
    compute x={ones,x}.
    compute xsq=t(x)*x.
    compute exsq=eval(xsq).
    release xsq.
    compute holymoly=cmin(exsq).
                                        /*     .000000000002344350286  */.
    compute zeroeig=csum(exsq <= 0.000000000002).
    compute bad=bad + (zeroeig > 0).
    describe descdatf=y/type=1.
    compute bad=bad+ (desctmp(2,1) <= 0.00000000001).
    do if (bad = 0).
      do if (holymoly < smallest).
```

```
      compute smallest=holymoly.
     end if.
     /* print smallest/format=F16.15 */.
     modelest y=y/x=x/type=1/full=0.
     compute modres2={modres2,t(modres)}.
     do if ( (bcmat((i+1),1) = 1) and (nobootx=1) and (effsize=1)).
      compute nobootx=0.
      compute xsdtemp= (nrow(x)*sscp(x(:,2)))-(t(csum(x(:,2)))*(csum(x(:,2)))).
      compute xsdtemp= sqrt(xsdtemp/(nrow(x)*(nrow(x)-1))).
      compute bootxsd={bootxsd;xsdtemp}.
     end if.
    end if.
  end loop.
  do if (bad = 0).
    compute bootres={bootres;modres2(:,2:ncol(modres2))}.
    do if (effsize=1).
     compute ysdtemp= (nrow(y)*sscp(y))-(t(csum(y))*(csum(y))).
     compute ysdtemp= sqrt(ysdtemp/(nrow(y)*(nrow(y)-1))).
     compute bootysd={bootysd;ysdtemp}.
    end if.
    compute goodboot=goodboot+1.
  end if.
  do if (bad <> 0).
    compute badboot=badboot+1.
  end if.
end loop if (goodboot = boot).
/* print smallest/format=F16.15 */.
compute bootres=bootres(2:nrow(bootres),:).
```

```
do if (effsize=1).
  compute bootysd=bootysd(2:nrow(bootysd),:).
  do if (nrow(bootxsd) > 1).
    compute bootxsd=bootxsd(2:nrow(bootxsd),:).
  end if.
end if.
do if (goodboot < (boot)).
  compute boot=0.
  compute modelbt=0.
  compute notecode(notes,1) = 7.
  compute notes = notes + 1.
end if.
do if (boot > 0).
   do if (effsize=1).
    compute bootysd={ysd;bootysd}.
    compute bootxsd={xsd;bootxsd}.
  end if.
  do if (saveboot = 1).
    save bootres/outfile = *.
  end if.
 compute bootcim=make(ncol(bootres),5,-99999).
 compute bootcim(:,2) = t(csum(bootres)/nrow(bootres)).
 compute bootcim(:,1) = coeffmat(2:nrow(coeffmat),1).
  loop i = 1 to ncol(bootres).
    bcboot3 databcbt = bootres(:,i).
    compute bootcim(i,4:5)={llcit,ulcit}.
    compute bootcim(i,3)=bootse.
  end loop.
```

```
  end if.
  do if (badboot > 0).
    compute notecode(notes,1) = 6.
    compute notes = notes + 1.
  end if.
end if.

/* print direct and indirect effects */.
do if (criterr=0).
  release fulldat,x,y.
end if.
DRINDEF decpnt=!decimals.

do if (criterr=0 and (saveest = 1 or saveboot=1)).
  compute coeffsav=coeffmat(2:nrow(coeffmat),:).
  compute conseq=conseq(2:nrow(conseq),1).
  compute dfmat=dfmat(2:nrow(dfmat),1).
  compute dfmat=dfmat.
  compute saven={"conseqnt","antecdnt","coeff","se","t","p","LLCI","ULCI","df"}.
  do if (saveest=1).
    save {conseq,vlabs,coeffsav,dfmat}/outfile = */names=saven/strings=conseqnt antecdnt.
  end if.
  do if (saveboot=1 and boot > 0 ).
    print/title =
"*********************************************************************************".
    print/title="Bootstrap estimates were saved to a file"/space=0.
    compute colslab=make(300,1," ").
    !do !i = 1 !to 300.
```

```
      !let !v2=!concat("COL",!unquote(!quote(!i))).
      compute colslab(!i,1)=!quote(!v2).
    !doend.
    compute colslab=colslab(1:ncol(bootres),1).
    compute colslab={colslab,conseq,vlabs}.
    print colslab/title="Map of column names to model coefficients:"/clabels="
","Conseqnt","Antecdnt"/format=a8.
  end if.
end if.


/* PRINT BOOTSTRAP RESULTS FOR MODEL PARAMETERS */.
do if (criterr=0 and boot > 0 and modelbt=1).
  compute labstart=1.
  print/title = "*********** BOOTSTRAP RESULTS FOR REGRESSION MODEL PARAMETERS
************".
  loop iboot = 1 to (nms+nys).
    print outnames(1,iboot)/title = "OUTCOME VARIABLE:"/format = A8.
    compute vlabsm=vlabs(labstart:(labstart+(nump(1,iboot)-1)),1).
    print bootcim(labstart:(labstart+(nump(1,iboot)-1)),:)/title=" "/rnames=vlabsm/
    clabels="Coeff" "BootMean" "BootSE" "BootLLCI" "BootULCI"/format=!decimals/space=0.
    compute labstart=labstart+nump(1,iboot).
    do if (iboot < (nms+nys)).
      print/title= "----------".
    end if.
  end loop.
end if.

/* PRINT MODEL MATRICES */.
```

```
do if (criterr = 0 and matrices=1).
  print/title = "************************ MODEL DEFINITION MATRICES
************************".
  print/title = "FROM variables are columns, TO variables are rows.".
  compute temp2=make(nrow(bcmat),ncol(bcmat),"0").
  loop i = 2 to nrow(bcmat).
    loop j = 1 to (ncol(bcmat)-1).
      do if (bcmat(i,j)=1).
        compute temp2(i,j)="1".
      end if.
      do if (j >= i).
        compute temp2(i,j)=" ".
      end if.
    end loop.
  end loop.
  compute temp2=temp2(2:nrow(bcmat),(1:(ncol(bcmat)-1))).
  do if (nms > 0).
    compute cmatlabs={xnames,mnames}.
    compute rmatlabs={mnames,ynames}.
  end if.
  do if (nms = 0).
    compute cmatlabs={xnames}.
    compute rmatlabs={ynames}.
  end if.
  print temp2/title="BMATRIX: Paths freely estimated (1) and fixed to zero
(0):"/cnames=cmatlabs/rnames=rmatlabs/format A3.
  compute z=0.
  do if (rsum(csum(wcmat))<>0).
    compute temp2=make(nrow(wcmat),ncol(wcmat),"0").
```

```
    loop i = 2 to nrow(wcmat).
      loop j = 1 to (ncol(wcmat)-1).
        do if (wcmat(i,j)=1).
          compute temp2(i,j)="1".
        end if.
        do if (j >= i).
          compute temp2(i,j)=" ".
        end if.
      end loop.
    end loop.
    compute temp2=temp2(2:nrow(wcmat),(1:(ncol(wcmat)-1))).
    print temp2/title="WMATRIX: Paths moderated (1) and not moderated (0) by
W:"/cnames=cmatlabs/rnames=rmatlabs/format A3.
  end if.
  do if (rsum(csum(zcmat))<>0).
    compute temp2=make(nrow(zcmat),ncol(zcmat),"0").
    loop i = 2 to nrow(zcmat).
      loop j = 1 to (ncol(zcmat)-1).
        do if (zcmat(i,j)=1).
          compute temp2(i,j)="1".
        end if.
        do if (j >= i).
          compute temp2(i,j)=" ".
        end if.
      end loop.
    end loop.
    compute temp2=temp2(2:nrow(zcmat),(1:(ncol(zcmat)-1))).
    print temp2/title="ZMATRIX: Paths moderated (1) and not moderated (0) by
Z:"/cnames=cmatlabs/rnames=rmatlabs/format a3.
```

```
  end if.
  do if (rsum(csum(wzcmat))<>0).
    compute temp2=make(nrow(wzcmat),ncol(wzcmat),"0").
    loop i = 2 to nrow(wzcmat).
      loop j = 1 to (ncol(wzcmat)-1).
        do if (wzcmat(i,j)=1).
          compute temp2(i,j)="1".
        end if.
        do if (j >= i).
          compute temp2(i,j)=" ".
        end if.
      end loop.
    end loop.
    compute temp2=temp2(2:nrow(wzcmat),(1:(ncol(wzcmat)-1))).
    print temp2/title="WZMATRIX: W moderated paths moderated (1) and not moderated (0) by
Z:"/cnames=cmatlabs/rnames=rmatlabs/format a3.
  end if.
  do if (ncs > 0).
    print ccmat/title="CMATRIX: Covariates (columns) in (1) and not in (0) the models of M and Y
(rows):"/rnames=rmatlabs/cnames=covnames.
  end if.
end if.

/* PRINT NOTES */.
print/title = "*********************** ANALYSIS NOTES AND ERRORS
************************".
do if (criterr=0).
  print conf/title = "Level of confidence for all confidence intervals in output:"/format = F8.4.
  do if (boot > 0).
```

```
    do if (goodboot = boot).
      print boot/title="Number of bootstrap samples for percentile bootstrap confidence intervals:".
    end if.
  end if.
  do if (mc > 0).
    print mc/title="Number of samples for Monte Carlo confidence intervals:".
  end if.
  do if (wnotev > 0 and printw=1).
    do if (wnotev=1).
      print/title = "W values in conditional tables are the 16th, 50th, and 84th percentiles.".
    else if (wnotev=2).
      do if (minwwarn=0 and maxwwarn=0).
        print/title = "W values in conditional tables are the mean and +/- SD from the mean.".
      end if.
      do if (minwwarn=1).
        print/title = "W values in conditional tables are the minimum, the mean, and 1 SD above the
mean.".
      end if.
      do if (maxwwarn=1).
        print/title = "W values in conditional tables are 1 SD below the mean, the mean, and the
maximum.".
      end if.
    end if.
  end if.
  do if (znotev > 0 and printz=1).
    do if (znotev=1).
      print/title = "Z values in conditional tables are the 16th, 50th, and 84th percentiles.".
    else if (znotev=2).
      do if (minzwarn=0 and maxzwarn=0).
```

```
      print/title = "Z values in conditional tables are the mean and +/- SD from the mean.".
    end if.
    do if (minzwarn=1).
      print/title = "Z values in conditional tables are the minimum, the mean, and 1 SD above the mean.".
    end if.
    do if (maxzwarn=1).
      print/title = "Z values in conditional tables are 1 SD below the mean, the mean, and the maximum.".
    end if.
  end if.
end if.
do if (minwwarn > 0).
  print/title = "NOTE: One SD below the mean is below the minimum observed in the data for W,".
  print/title = "      so the minimum measurement on W is used for conditioning instead."/space=0.
end if.
do if (maxwwarn > 0).
  print/title = "NOTE: One SD above the mean is above the maximum observed in the data for W,".
  print/title = "      so the maximum measurement for W is used for conditioning instead."/space=0.
end if.
do if (minzwarn > 0).
  print/title = "NOTE: One SD below the mean is below the minimum observed in the data for Z,".
  print/title = "      so the minimum measurement for Z is used for conditioning instead."/space=0.
end if.
do if (maxzwarn > 0).
  print/title = "NOTE: One SD above the mean is above the maximum observed in the data for Z,".
  print/title = "      so the maximum measurement for Z is used for conditioning instead."/space=0.
end if.
loop i = 1 to 100.
```

```
   do if (notecode(i,1)=1).
     print/title = "NOTE: COVMY is ignored when using CMATRIX option.".
   end if.
   do if (notecode(i,1)=2).
     print/title = "NOTE: Confidence level restricted to between 50 and 99.9999%.  95% confidence is
provided in output".
   end if.
   do if (notecode(i,1)=3).
     print centvar/title = "NOTE: The following variables were mean centered prior to
analysis:"/format = a8.
   end if.
   do if (notecode(i,1) = 4).
     print/title = "NOTE: A heteroscedasticity consistent standard error and covariance matrix
estimator was used.".
   end if.
   do if (notecode(i,1) = 5).
     print/title = "NOTE: The HC3 option has been replaced with HC.  See the documentation.".
   end if.
   do if (notecode(i,1) = 6).
     print/title = "NOTE: Due to estimation problems, some bootstrap samples had to be replaced.".
     print badboot/title="      The number of times this happened was:"/space=0/format=F8.0.
   end if.
   do if (notecode(i,1) = 7).
     print/title = "NOTE: The bootstrapping was not completed due to problematic bootstrap
samples.".
     print/title = "      Bootstrap confidence intervals are therefore suppressed."/space=0.
   end if.
   do if (notecode(i,1) = 8).
     print/title = "NOTE: The number of bootstrap samples was adjusted upward given your desired
confidence.".
```

```
  end if.
  do if (notecode(i,1) = 9).
    print/title = "NOTE: WMODVAL is ignored when W is specified as multicategorical.".
  end if.
  do if (notecode(i,1) = 10).
    print/title = "NOTE: ZMODVAL is ignored when Z is specified as multicategorical.".
  end if.
  do if (notecode(i,1) = 11).
    print/title = "NOTE: Total effect model and estimate generated only when all covariates are
specified in all".
    print/title = "      models of M and Y."/space=0.
  end if.
  do if (notecode(i,1) = 12).
    print/title = "NOTE: Total effect model and estimate generated only when X is freely estimated to
affect each M".
    print/title = "      and both X and M are freely estimated to affect Y"/space=0.
  end if.
  do if (notecode(i,1) = 13).
    print/title = "NOTE: There are too many pairwise contrasts to conduct with this model.".
  end if.
  do if (notecode(i,1) = 14).
    print/title = "NOTE: The number of contrast weights must equal the number of indirect effects.".
  end if.
  do if (notecode(i,1) = 15).
    print/title = "NOTE: Monte Carlo confidence intervals not available for this model.".
    print/title = "      Bootstrapping is used instead."/space=0.
  end if.
  do if (notecode(i,1) = 16).
```

```
      print/title = "NOTE: The number of Monte Carlo samples was adjusted upward given your desired confidence.".
    end if.
    do if (notecode(i,1) = 19).
      print/title = "NOTE: Your contrast matrix is invalid or not applicable to this model.".
    end if.
    do if (notecode(i,1) = 20).
      print/title = "NOTE: One of the groups specified by your contrast matrix does not exist in the data.".
    end if.
    do if (notecode(i,1) = 21).
      print/title = "NOTE: The VARORDER option is not available in this release.".
    end if.
    do if (notecode(i,1) = 22).
      print/title = "NOTE: The VMODVAL and QMODVAL options are not available in this release.".
    end if.
    do if (notecode(i,1) = 23).
      print/title = "NOTE: The QUANTILE option is not available in this release.".
    end if.
  end loop.
  do if (toomany=1).
    print/title="NOTE: Variables names longer than eight characters can produce incorrect output.".
    print/title="      Shorter variable names are recommended."/space=0.
  end if.
end if.


/* PRINT ERRORS */.
loop i = 1 to 100.
```

```
  do if (errcode(i,1)=1).

    print/title = "ERROR: You must specify a Y and an X variable.".

  end if.

  do if (errcode(i,1)=2).

    print/title = "ERROR: A variable can appear only once in a PROCESS command.".

    do if (toomany = 1).

      print/title = "       This might be caused by the use of variables names longer"/space=0.

      print varnames/title = "       than eight characters. Here are the variables I
see:"/space=0/format=A8.

    end if.

  end if.

  do if (errcode(i,1)=3).

    print/title = "ERROR: You have specified more than one variable for W, Y, M, or Z".

  end if.

  do if (errcode(i,1)=4).

    print/title = "ERROR: A variable specified as multicategorical has more than nine categories.".

  end if.

  do if (errcode(i,1)=5).

    print/title = "ERROR: One of the categories contains only a single case.".

  end if.

  do if (errcode(i,1)=6).

    print/title = "ERROR: Invalid model number in this version of PROCESS.".

  end if.

  do if (errcode(i,1)=7).

    print/title = "ERROR: Invalid model number.".

  end if.

  do if (errcode(i,1)=8).

    print/title = "ERROR: You must specify an M variable for this model.".
```

```
end if.
do if (errcode(i,1)=9).
  print/title = "ERROR: You have specified an M variable in a model that does not use it.".
  print/title = "In this release of PROCESS, moderators are W and Z in models 1, 2, and 3."/space=0.
end if.
do if (errcode(i,1)=10).
  print/title = "ERROR: You have specified a W variable in a model that does not use it.".
end if.
do if (errcode(i,1)=11).
  print/title = "ERROR: You have not specified a W variable in a model that requires it.".
end if.
do if (errcode(i,1)=12).
  print/title = "ERROR: You have specified a Z variable in a model that does not use it.".
end if.
do if (errcode(i,1)=13).
  print/title = "ERROR: You have not specified a Z variable in a model that requires it.".
end if.
do if (errcode(i,1)=14).
  print/title = "ERROR: V and Q are not proper specifications in this release of PROCESS.".
  print/title = "       Moderators must be specified as W and/or Z."/space=0.
end if.
do if (errcode(i,1)=15).
  print/title = "ERROR: One of your model variables exhibits no variation (it is a constant).".
end if.
do if (errcode(i,1)=16).
  print/title = "ERROR: BMATRIX is not the correct length or is otherwise invalid.".
end if.
do if (errcode(i,1)=17).
```

```
  print/title = "ERROR: WMATRIX is not the correct length or is otherwise invalid.".
end if.
do if (errcode(i,1)=18).
  print/title = "ERROR: ZMATRIX is not the correct length or is otherwise invalid.".
end if.
do if (errcode(i,1)=19).
  print/title = "ERROR: WZMATRIX is not the correct length or is otherwise invalid.".
end if.
do if (errcode(i,1)=20).
  print/title = "ERROR: A path fixed at zero cannot be moderated.".
end if.
do if (errcode(i,1)=21).
  print/title = "ERROR: If only one moderator is specified, it must be specified as W.".
end if.
do if (errcode(i,1)=22).
  print/title = "ERROR: In BMATRIX, X must be specified to affect at least one variable.".
end if.
do if (errcode(i,1)=23).
  print/title = "ERROR: In BMATRIX, at least one variable must be specified to affect Y.".
end if.
do if (errcode(i,1)=24).
  print/title = "ERROR: You must specify a model number or a custom BMATRIX specification.".
end if.
do if (errcode(i,1)=25).
  print/title = "ERROR: BMATRIX cannot be used in conjunction with a model number.".
end if.
do if (errcode(i,1)=26).
  print/title = "ERROR: Your model has a dangling mediator (all Ms must affect and be affected).".
```

```
end if.
do if (errcode(i,1)=27).
  print/title = "ERROR: CLUSTER is not available on this release of PROCESS.".
end if.
do if (errcode(i,1)=29).
  print/title = "ERROR: CMATRIX is not the correct length or is otherwise invalid.".
end if.
do if (errcode(i,1)=30).
  print/title = "ERROR: In CMATRIX, all covariates must be assigned to an M or a Y.".
end if.
do if (errcode(i,1)=31).
  print/title = "ERROR: A linear or near linear dependency (singularity) exists in the data.".
end if.
do if (errcode(i,1)=32).
  print/title = "ERROR: Models 80 and 81 require between 3 and 6 mediators.".
end if.
do if (errcode(i,1)=33).
  print/title = "ERROR: Model 82 requires 4 mediators.".
end if.
do if (errcode(i,1)=34).
  print/title = "ERROR: This model number requires between 2 and 6 mediators.".
end if.
do if (errcode(i,1)=35).
  print/title = "ERROR: In a model with only one moderator, that moderator must be W.".
end if.
do if (errcode(i,1)=36).
  print/title = "ERROR: A serial mediation model cannot have more than 6 mediators.".
end if.
```

```
  do if (errcode(i,1)=37).
    print/title = "ERROR: No more than 10 mediators are allowed in a PROCESS command.".
  end if.
  do if (errcode(i,1)=38).
    print/title = "ERROR: XCATCODE is not provided, not the correct length, or is otherwise invalid.".
  end if.
  do if (errcode(i,1)=39).
    print/title = "ERROR: WCATCODE is not provided, not the correct length, or is otherwise invalid.".
  end if.
  do if (errcode(i,1)=40).
    print/title = "ERROR: ZCATCODE is not provided, not the correct length, or is otherwise invalid.".
  end if.
  do if (errcode(i,1)=41).
    print/title = "ERROR: Models 1, 2, 3, and 74 cannot be customized.".
  end if.
  do if (errcode(i,1)=42).
    print/title = "ERROR: WS option available only in PROCESS v2. Or use the MEMORE macro
instead.".
    print/title = "       MEMORE can be downloaded from www.akmontoya.com."/space=0.
  end if.
  do if (errcode(i,1)=43).
    print/title = "ERROR: PROCESS does not allow dichotomous mediators.".
  end if.
  do if (errcode(i,1)=44).
    print/title = "ERROR: This release of PROCESS does not allow a dichotomous Y.".
  end if.
  do if (errcode(i,1)=45).
    print/title = "ERROR: In model 74, X and W must be the same variable.".
```

```
  end if.
  do if (errcode(i,1)=46).
    print/title = "ERROR: Model 74 is temporarily disabled in this release of PROCESS.".
  end if.
end loop.
END MATRIX.
set printback=on.
!ENDDEFINE.




***.
***.
***.
***.

**********************************************************************************.
***Stichprobenbeschreibung**************************************************.
**********************************************************************************.

**********************************************************************************.
***Sample A*******************************************************************.
**********************************************************************************.

USE ALL.
COMPUTE filter_$=(Sample = 0).
VARIABLE LABELS filter_$ 'Sample = 0 (FILTER)'.
```

```
VALUE LABELS filter_$ 0 'Not Selected' 1 'Selected'.

FORMATS filter_$ (f1.0).

FILTER BY filter_$.

EXECUTE.


***Häufigkeiten Geschlecht***.

FREQUENCIES VARIABLES=sex

  /BARCHART FREQ

  /ORDER=ANALYSIS.


***Alter***.

DESCRIPTIVES VARIABLES=age

  /STATISTICS=MEAN STDDEV MIN MAX.


***Häufigkeiten Schulbildung***.

FREQUENCIES VARIABLES=schul

  /BARCHART FREQ

  /ORDER=ANALYSIS.


***Häufigkeiten Ledig***.

FREQUENCIES VARIABLES=fam

  /BARCHART FREQ

  /ORDER=ANALYSIS.


***Häufigkeiten aktuelle Behandlungsart***.

FREQUENCIES VARIABLES=treat

  /ORDER=ANALYSIS.
```

```
***PHQ***.
DESCRIPTIVES VARIABLES=PHQ
  /STATISTICS=MEAN STDDEV MIN MAX.


***pub***.
DESCRIPTIVES VARIABLES=pub_b
  /STATISTICS=MEAN STDDEV MIN MAX.


***per***.
DESCRIPTIVES VARIABLES=per_b
  /STATISTICS=MEAN STDDEV MIN MAX.


***self***.
DESCRIPTIVES VARIABLES=self_b
  /STATISTICS=MEAN STDDEV MIN MAX.


***SW***.
DESCRIPTIVES VARIABLES=SW_B
  /STATISTICS=MEAN STDDEV MIN MAX.



***********************************************************************************.
***Reliabilities****************************************.
***********************************************************************************.
***PHQ-9***.
RELIABILITY
  /VARIABLES=PH01 PH02 PH03 PH04 PH05 PH06 PH07 PH08 PH09
  /SCALE('ALL VARIABLES') ALL
```

```
  /MODEL=ALPHA
  /STATISTICS=DESCRIPTIVE SCALE CORR
  /SUMMARY=MEANS VARIANCE.

***SSMIS: stereotype awareness***.
RELIABILITY
  /VARIABLES=PUB01_B PUB02_B PUB03_B PUB04_B PUB05_B PUB06_B  PUB07_B
    PUB08_B PUB09_B PUB10_B
  /SCALE('ALL VARIABLES') ALL
  /MODEL=ALPHA
  /STATISTICS=DESCRIPTIVE SCALE CORR
  /SUMMARY=MEANS VARIANCE.

***SSMIS: stereotype agreement***.
RELIABILITY
  /VARIABLES=PER01_B PER02_B PER03_B PER04_B PER05_B PER06_B  PER07_B
    PER08_B PER09_B PER10_B
  /SCALE('ALL VARIABLES') ALL
  /MODEL=ALPHA
  /STATISTICS=DESCRIPTIVE SCALE CORR
  /SUMMARY=MEANS VARIANCE.

***SSMIS: self concurrence***.
RELIABILITY
  /VARIABLES=SELB01_B SELB02_B SELB03_B SELB04_B SELB05_B SELB06_B SELB07_B
    SELB08_B SELB09_B SELB10_B
  /SCALE('ALL VARIABLES') ALL
  /MODEL=ALPHA
```

```
  /STATISTICS=DESCRIPTIVE SCALE CORR
  /SUMMARY=MEANS VARIANCE.

***self esteem***.
RELIABILITY
  /VARIABLES=SW02_B SW05_B SW06_B SW08_B SW09_B SW01_B SW03_B SW04_B SW07_B
SW10_B
  /SCALE('ALL VARIABLES') ALL
  /MODEL=ALPHA
  /STATISTICS=DESCRIPTIVE SCALE CORR
  /SUMMARY=MEANS VARIANCE.

**Outliers***.
EXAMINE VARIABLES=pub_b per_b self_b SW_B AGE SEX PHQ
  /PLOT BOXPLOT STEMLEAF
  /COMPARE GROUPS
  /STATISTICS DESCRIPTIVES
  /CINTERVAL 95
  /MISSING LISTWISE
  /NOTOTAL.

***normal distribution***.
EXAMINE VARIABLES=pub_b per_b self_b SW_B AGE SEX PHQ
  /PLOT BOXPLOT HISTOGRAM NPPLOT
  /COMPARE GROUPS
  /STATISTICS DESCRIPTIVES
  /CINTERVAL 95
  /MISSING LISTWISE
```

```
  /NOTOTAL.

***Spearman Correlations***.
NONPAR CORR
  /VARIABLES=sex age PHQ pub_b per_b self_b SW_B
  /PRINT=SPEARMAN TWOTAIL NOSIG
  /MISSING=PAIRWISE.



**********************************************************************************.
***Sample B********************************************************************.
**********************************************************************************.

USE ALL.
COMPUTE filter_$=(Sample = 1).
VARIABLE LABELS filter_$ 'Sample = 1 (FILTER)'.
VALUE LABELS filter_$ 0 'Not Selected' 1 'Selected'.
FORMATS filter_$ (f1.0).
FILTER BY filter_$.
EXECUTE.

***Häufigkeiten Geschlecht***.
FREQUENCIES VARIABLES=sex
  /BARCHART FREQ
  /ORDER=ANALYSIS.

***Alter***.
DESCRIPTIVES VARIABLES=age
```

```
  /STATISTICS=MEAN STDDEV MIN MAX.

***Häufigkeiten Schulbildung***.
FREQUENCIES VARIABLES=schul
  /BARCHART FREQ
  /ORDER=ANALYSIS.

***Häufigkeiten Ledig***.
FREQUENCIES VARIABLES=fam
  /BARCHART FREQ
  /ORDER=ANALYSIS.

***Häufigkeiten aktuelle Behandlungsart***.
FREQUENCIES VARIABLES=treat
  /ORDER=ANALYSIS.

***PHQ***.
DESCRIPTIVES VARIABLES=PHQ
  /STATISTICS=MEAN STDDEV MIN MAX.

***pub***.
DESCRIPTIVES VARIABLES=pub_b
  /STATISTICS=MEAN STDDEV MIN MAX.

***per***.
DESCRIPTIVES VARIABLES=per_b
  /STATISTICS=MEAN STDDEV MIN MAX.
```

```
***self***.
DESCRIPTIVES VARIABLES=self_b
  /STATISTICS=MEAN STDDEV MIN MAX.

***SW***.
DESCRIPTIVES VARIABLES=SW_B
  /STATISTICS=MEAN STDDEV MIN MAX.



***********************************************************************************.
***Reliabilities****************************************.
***********************************************************************************.

***PHQ-9***.
RELIABILITY
  /VARIABLES=PH01 PH02 PH03 PH04 PH05 PH06 PH07 PH08 PH09
  /SCALE('ALL VARIABLES') ALL
  /MODEL=ALPHA
  /STATISTICS=DESCRIPTIVE SCALE CORR
  /SUMMARY=MEANS VARIANCE.

***SSMIS: stereotype awareness***.
RELIABILITY
  /VARIABLES=PUB01_B PUB02_B PUB03_B PUB04_B PUB05_B PUB06_B  PUB07_B
    PUB08_B PUB09_B PUB10_B
  /SCALE('ALL VARIABLES') ALL
  /MODEL=ALPHA
  /STATISTICS=DESCRIPTIVE SCALE CORR
```

```
  /SUMMARY=MEANS VARIANCE.

***SSMIS: stereotype agreement***.
RELIABILITY
  /VARIABLES=PER01_B PER02_B PER03_B PER04_B PER05_B PER06_B  PER07_B
    PER08_B PER09_B PER10_B
  /SCALE('ALL VARIABLES') ALL
  /MODEL=ALPHA
  /STATISTICS=DESCRIPTIVE SCALE CORR
  /SUMMARY=MEANS VARIANCE.

***SSMIS: self concurrence***.
RELIABILITY
  /VARIABLES=SELB01_B SELB02_B SELB03_B SELB04_B SELB05_B SELB06_B SELB07_B
    SELB08_B SELB09_B SELB10_B
  /SCALE('ALL VARIABLES') ALL
  /MODEL=ALPHA
  /STATISTICS=DESCRIPTIVE SCALE CORR
  /SUMMARY=MEANS VARIANCE.

***self esteem***.
RELIABILITY
  /VARIABLES=SW02_B SW05_B SW06_B SW08_B SW09_B SW01_B SW03_B SW04_B SW07_B
SW10_B
  /SCALE('ALL VARIABLES') ALL
  /MODEL=ALPHA
  /STATISTICS=DESCRIPTIVE SCALE CORR
  /SUMMARY=MEANS VARIANCE.
```

```
**Outliers***.

EXAMINE VARIABLES=pub_b per_b self_b SW_B AGE SEX PHQ

  /PLOT BOXPLOT STEMLEAF

  /COMPARE GROUPS

  /STATISTICS DESCRIPTIVES

  /CINTERVAL 95

  /MISSING LISTWISE

  /NOTOTAL.


***normal distribution***.

EXAMINE VARIABLES=pub_b per_b self_b SW_B AGE SEX PHQ

  /PLOT BOXPLOT HISTOGRAM NPPLOT

  /COMPARE GROUPS

  /STATISTICS DESCRIPTIVES

  /CINTERVAL 95

  /MISSING LISTWISE

  /NOTOTAL.


***Spearman Correlations***.

NONPAR CORR

  /VARIABLES=sex age PHQ pub_b per_b self_b SW_B

  /PRINT=SPEARMAN TWOTAIL NOSIG

  /MISSING=PAIRWISE.



*********************************************************************************.

***Total Sample*******************************************************************.
```

```
******************************************************************************************.

USE ALL.

***Häufigkeiten Geschlecht***.
FREQUENCIES VARIABLES=sex
  /BARCHART FREQ
  /ORDER=ANALYSIS.

***Alter***.
DESCRIPTIVES VARIABLES=age
  /STATISTICS=MEAN STDDEV MIN MAX.

***Häufigkeiten Schulbildung***.
FREQUENCIES VARIABLES=schul
  /BARCHART FREQ
  /ORDER=ANALYSIS.

***Häufigkeiten Ledig***.
FREQUENCIES VARIABLES=fam
  /BARCHART FREQ
  /ORDER=ANALYSIS.

***Häufigkeiten aktuelle Behandlungsart***.
FREQUENCIES VARIABLES=treat
  /ORDER=ANALYSIS.

***PHQ***.
```

```
DESCRIPTIVES VARIABLES=PHQ
  /STATISTICS=MEAN STDDEV MIN MAX.


***pub***.
DESCRIPTIVES VARIABLES=pub_b
  /STATISTICS=MEAN STDDEV MIN MAX.


***per***.
DESCRIPTIVES VARIABLES=per_b
  /STATISTICS=MEAN STDDEV MIN MAX.


***self***.
DESCRIPTIVES VARIABLES=self_b
  /STATISTICS=MEAN STDDEV MIN MAX.


***SW***.
DESCRIPTIVES VARIABLES=SW_B
  /STATISTICS=MEAN STDDEV MIN MAX.


**********************************************************************************.
***Reliabilities***************************************.
**********************************************************************************.


***PHQ-9***.
RELIABILITY
  /VARIABLES=PH01 PH02 PH03 PH04 PH05 PH06 PH07 PH08 PH09
  /SCALE('ALL VARIABLES') ALL
  /MODEL=ALPHA
```

```
  /STATISTICS=DESCRIPTIVE SCALE CORR
  /SUMMARY=MEANS VARIANCE.

***SSMIS: stereotype awareness***.
RELIABILITY
  /VARIABLES=PUB01_B PUB02_B PUB03_B PUB04_B PUB05_B PUB06_B  PUB07_B
    PUB08_B PUB09_B PUB10_B
  /SCALE('ALL VARIABLES') ALL
  /MODEL=ALPHA
  /STATISTICS=DESCRIPTIVE SCALE CORR
  /SUMMARY=MEANS VARIANCE.

***SSMIS: stereotype agreement***.
RELIABILITY
  /VARIABLES=PER01_B PER02_B PER03_B PER04_B PER05_B PER06_B  PER07_B
    PER08_B PER09_B PER10_B
  /SCALE('ALL VARIABLES') ALL
  /MODEL=ALPHA
  /STATISTICS=DESCRIPTIVE SCALE CORR
  /SUMMARY=MEANS VARIANCE.

***SSMIS: self concurrence***.
RELIABILITY
  /VARIABLES=SELB01_B SELB02_B SELB03_B SELB04_B SELB05_B SELB06_B SELB07_B
    SELB08_B SELB09_B SELB10_B
  /SCALE('ALL VARIABLES') ALL
  /MODEL=ALPHA
  /STATISTICS=DESCRIPTIVE SCALE CORR
```

```
  /SUMMARY=MEANS VARIANCE.

***self esteem***.
RELIABILITY
  /VARIABLES=SW02_B SW05_B SW06_B SW08_B SW09_B SW01_B SW03_B SW04_B SW07_B
SW10_B
  /SCALE('ALL VARIABLES') ALL
  /MODEL=ALPHA
  /STATISTICS=DESCRIPTIVE SCALE CORR
  /SUMMARY=MEANS VARIANCE.

**Outliers***.
EXAMINE VARIABLES=pub_b per_b self_b SW_B AGE SEX PHQ
  /PLOT BOXPLOT STEMLEAF
  /COMPARE GROUPS
  /STATISTICS DESCRIPTIVES
  /CINTERVAL 95
  /MISSING LISTWISE
  /NOTOTAL.

***normal distribution***.
EXAMINE VARIABLES=pub_b per_b self_b SW_B AGE SEX PHQ
  /PLOT BOXPLOT HISTOGRAM NPPLOT
  /COMPARE GROUPS
  /STATISTICS DESCRIPTIVES
  /CINTERVAL 95
  /MISSING LISTWISE
  /NOTOTAL.
```

Spearman Correlations.

```
NONPAR CORR
  /VARIABLES=sex age PHQ pub_b per_b self_b SW_B
  /PRINT=SPEARMAN TWOTAIL NOSIG
  /MISSING=PAIRWISE.
```

Testing assumptions for t-tests of sample characteristics.

1. normal distribution.

```
USE ALL.
COMPUTE filter_$=(Sample = 0).
VARIABLE LABELS filter_$ 'Sample = 0 (FILTER)'.
VALUE LABELS filter_$ 0 'Not Selected' 1 'Selected'.
FORMATS filter_$ (f1.0).
FILTER BY filter_$.
EXECUTE.

EXAMINE VARIABLES=AGE PHQ_kat
  /PLOT BOXPLOT HISTOGRAM NPPLOT
  /COMPARE GROUPS
  /STATISTICS DESCRIPTIVES
  /CINTERVAL 95
  /MISSING LISTWISE
  /NOTOTAL.
```

```
USE ALL.

COMPUTE filter_$=(Sample = 1).

VARIABLE LABELS filter_$ 'Sample = 1 (FILTER)'.

VALUE LABELS filter_$ 0 'Not Selected' 1 'Selected'.

FORMATS filter_$ (f1.0).

FILTER BY filter_$.

EXECUTE.


EXAMINE VARIABLES=AGE PHQ_kat

  /PLOT BOXPLOT HISTOGRAM NPPLOT

  /COMPARE GROUPS

  /STATISTICS DESCRIPTIVES

  /CINTERVAL 95

  /MISSING LISTWISE

  /NOTOTAL.


***t-Tests cannot be conducted.***

***Instead: Mann-Whitney-U-Tests for interval scaled variables of independent samples***.


USE ALL.


NPAR TESTS

 /M-W=AGE BY SAMPLE (0 1)

 /STATISTICS=DESCRIPTIVES.


NPAR TESTS

 /M-W=PHQ_kat BY SAMPLE (0 1)

 /STATISTICS=DESCRIPTIVES.
```

```
CROSSTABS
  /TABLES=SEX schul part fam treat BY Sample
  /FORMAT=AVALUE TABLES
  /STATISTICS=CHISQ PHI
  /CELLS=COUNT EXPECTED ROW COLUMN TOTAL
  /COUNT ROUND CELL
  /HIDESMALLCOUNTS COUNT=5.

NPAR TESTS
 /M-W=PHQ BY SAMPLE (0 1)
 /STATISTICS=DESCRIPTIVES.

NPAR TESTS
 /M-W=pub_b BY SAMPLE (0 1)
 /STATISTICS=DESCRIPTIVES.

NPAR TESTS
 /M-W=per_b BY SAMPLE (0 1)
 /STATISTICS=DESCRIPTIVES.

NPAR TESTS
 /M-W=self_b BY SAMPLE (0 1)
 /STATISTICS=DESCRIPTIVES.

NPAR TESTS
 /M-W=SW_b BY SAMPLE (0 1)
 /STATISTICS=DESCRIPTIVES.
```

```
***Testing assumptions of OLS***.

USE ALL.

COMPUTE filter_$=(Sample = 0).

VARIABLE LABELS filter_$ 'Sample = 0 (FILTER)'.

VALUE LABELS filter_$ 0 'Not Selected' 1 'Selected'.

FORMATS filter_$ (f1.0).

FILTER BY filter_$.

EXECUTE.


REGRESSION

  /MISSING LISTWISE

  /STATISTICS COEFF OUTS R ANOVA COLLIN TOL

  /CRITERIA=PIN(.05) POUT(.10)

  /NOORIGIN

  /DEPENDENT ZSW_B

  /METHOD=ENTER Zpub_b ZPHQ ZAGE ZSEX Zper_b Zself_b

  /SCATTERPLOT=(*SDRESID ,*ZPRED)

  /RESIDUALS DURBIN HISTOGRAM(ZRESID).


USE ALL.

COMPUTE filter_$=(Sample = 1).

VARIABLE LABELS filter_$ 'Sample = 1 (FILTER)'.

VALUE LABELS filter_$ 0 'Not Selected' 1 'Selected'.

FORMATS filter_$ (f1.0).

FILTER BY filter_$.

EXECUTE.
```

```
REGRESSION
  /MISSING LISTWISE
  /STATISTICS COEFF OUTS R ANOVA COLLIN TOL
  /CRITERIA=PIN(.05) POUT(.10)
  /NOORIGIN
  /DEPENDENT ZSW_B
  /METHOD=ENTER Zpub_b ZPHQ ZAGE ZSEX Zper_b Zself_b
  /SCATTERPLOT=(*SDRESID ,*ZPRED)
  /RESIDUALS DURBIN HISTOGRAM(ZRESID).

*******************************************************.
***Serial Mediation Model***.
*******************************************************.

***Sample A***.
USE ALL.
COMPUTE filter_$=(Sample = 0).
VARIABLE LABELS filter_$ 'Sample = 0 (FILTER)'.
VALUE LABELS filter_$ 0 'Not Selected' 1 'Selected'.
FORMATS filter_$ (f1.0).
FILTER BY filter_$.
EXECUTE.

process vars = Zpub_b Zper_b Zself_b/y = ZSW_b/x = Zpub_b/m = Zper_b Zself_B/ cov= ZAGE ZSEX
ZPHQ/model = 6/total = 1
/effsize = 1/boot=10000.

***Sample B***.
```

```
USE ALL.

COMPUTE filter_$=(Sample = 1).

VARIABLE LABELS filter_$ 'Sample = 1 (FILTER)'.

VALUE LABELS filter_$ 0 'Not Selected' 1 'Selected'.

FORMATS filter_$ (f1.0).

FILTER BY filter_$.

EXECUTE.


***Total Sample***.

USE ALL.

process vars = Zpub_b Zper_b Zself_b/y = ZSW_b/x = Zpub_b/m = Zper_b Zself_B/ cov= ZAGE ZSEX
ZPHQ/model = 6/total = 1

/effsize = 1/boot=10000.



********************************************************.

********************************************************.

***Data for Trickle Down hypotheses***.

********************************************************.

********************************************************.


***Sample A***.

USE ALL.

COMPUTE filter_$=(Sample = 1).

VARIABLE LABELS filter_$ 'Sample = 1 (FILTER)'.

VALUE LABELS filter_$ 0 'Not Selected' 1 'Selected'.

FORMATS filter_$ (f1.0).

FILTER BY filter_$.
```

```
EXECUTE.

*Nonparametric Tests: Independent Samples.
NPTESTS
  /INDEPENDENT TEST (Means) GROUP (Var)
  /MISSING SCOPE=ANALYSIS USERMISSING=EXCLUDE
  /CRITERIA ALPHA=0.05  CILEVEL=95.

***Sample B***.
USE ALL.
COMPUTE filter_$=(Sample = 2).
VARIABLE LABELS filter_$ 'Sample = 2 (FILTER)'.
VALUE LABELS filter_$ 0 'Not Selected' 1 'Selected'.
FORMATS filter_$ (f1.0).
FILTER BY filter_$.
EXECUTE.

*Nonparametric Tests: Independent Samples.
NPTESTS
  /INDEPENDENT TEST (Means) GROUP (Var)
  /MISSING SCOPE=ANALYSIS USERMISSING=EXCLUDE
  /CRITERIA ALPHA=0.05  CILEVEL=95.

***Total Sample***.
USE ALL.

*Nonparametric Tests: Independent Samples.
NPTESTS
```

```
/INDEPENDENT TEST (Means) GROUP (Var)

/MISSING SCOPE=ANALYSIS USERMISSING=EXCLUDE

/CRITERIA ALPHA=0.05  CILEVEL=95.
```
