# Supplementary material for: Applying Corrigan’s progressive model of self-stigma to people with depression
Source: PLoS One. 2019 Oct 29;14(10):e0224418. doi: 10.1371/journal.pone.0224418 (PMC6818799; doi:10.1371/journal.pone.0224418)
Supplement: S4 File — (PDF) [file pone.0224418.s004.pdf]

Results of Serial Mediation Models

Sample A

PROCESS Procedure for SPSS Version 3.00 (Written by Andrew F. Hayes, Ph.D. www.afhayes.com)
Documentation available in Hayes (2018). www.guilford.com/p/hayes3

Model: 6
Y: self-esteem
X: stereotype awareness
M1: stereotype agreement
M2: self-concurrence
Covariates: age, gender depression
Sample Size: 550

Direct effects
Outcome variable: stereotype agreement
Model summary

R	R^2	MSE	F	df1	df2	*p*
0.2928	0.0858	0.9210	12.7799	4.0000	545.0000	0.0000

Model

	coeff	se	t	*p*	LLCI	ULCI
constant	0.0000	0.0409	0.0000	1.0000	-0.0804	0.0804
stereotype awareness	0.1822	0.0418	4.3558	0.0000	0.1000	0.2644
age	-0.0783	0.0415	-1.8869	0.0597	-0.1597	0.0032
gender	0.1214	0.0413	2.9358	0.0035	0.0402	0.2026
depression	0.1523	0.0419	3.6381	0.0003	0.0701	0.2346

Outcome variable: self-concurrence
Model summary

R	R^2	MSE	F	df1	df2	*p*
0.6801	0.4626	0.5424	93.6471	5.0000	544.0000	0.0000

Model

	coeff	se	t	*p*	LLCI	ULCI
constant	0.0000	0.0314	0.0000	1.0000	-0.0617	0.0617
stereotype awareness	0.0021	0.0327	0.0637	0.9492	-0.0621	0.0662
stereotype agreement	0.5055	0.0329	15.3785	0.0000	0.4410	0.5701
age	-0.1424	0.0319	-4.4586	0.0000	-0.2051	-0.0797
gender	0.0765	0.0320	2.3910	0.0171	0.0136	0.1393
depression	0.3034	0.0325	9.3286	0.0000	0.2395	0.3672

Outcome variable: self-esteem
Model summary

R	R^2	MSE	F	df1	df2	*p*
0.6818	0.4648	0.5411	78.6050	6.0000	543.0000	0.0000

Model

	coeff	se	t	*p*	LLCI	ULCI
constant	0.0000	0.0314	0.0000	1.0000	-0.0616	0.0616
stereotype awareness	-0.0671	0.0326	-2.0583	0.0400	-0.1312	-0.0031
stereotype agreement	0.0570	0.0393	1.4491	0.1479	-0.0203	0.1342
self-concurrence	-0.2370	0.0428	-5.5332	0.0000	-0.3211	-0.1528
age	0.1658	0.0325	5.1057	0.0000	0.1020	0.2296
gender	0.0441	0.0321	1.3734	0.1702	-0.0190	0.1072
depression	-0.4984	0.0350	-14.2472	0.0000	-0.5671	-0.4297

Total effect
Outcome variable: self-esteem
Model summary

R	R^2	MSE	F	df1	df2	*p*
0.6565	0.4310	0.5731	103.2259	4.0000	545.0000	0.0000

Model

	coeff	se	t	*p*	LLCI	ULCI
constant	0.0000	0.0323	0.0000	1.0000	-0.0634	0.0634
stereotype awareness	-0.0791	0.0330	-2.3961	0.0169	-0.1439	-0.0142
age	0.2045	0.0327	6.2485	0.0000	0.1402	0.2687
gender	0.0184	0.0326	0.5628	0.5738	-0.0457	0.0824
depression	-0.5798	0.0330	-17.5552	0.0000	-0.6447	-0.5150

Indirect effects

	Effect	BootSE	BootLLCI	BootULCI
total	-0.0119	0.0106	-0.0339	0.0079
stereotype awareness → stereotype agreement → self-esteem	0.0104	0.0080	-0.0031	0.0280
stereotype awareness → self-concurrence → self-esteem	-0.0005	0.0079	-0.0165	0.0149
stereotype awareness → stereotype agreement → self-concurrence → self-esteem	-0.0218	0.0073	-0.0380	-0.0096

Analysis notes:
Level of confidence for all confidence intervals in output: 95.0000
Number of bootstrap samples for percentile bootstrap confidence intervals: 10000

Sample B

PROCESS Procedure for SPSS Version 3.00 (Written by Andrew F. Hayes. Ph.D. www.afhayes.com)
Documentation available in Hayes (2018). www.guilford.com/p/hayes3

Model: 6
Y: self-esteem
X: stereotype awareness
M1: stereotype agreement
M2: self-concurrence
Covariates: age. gender depression
Sample Size: 180

Direct effects
Outcome variable: stereotype agreement
Model summary

R	R^2	MSE	F	df1	df2	*p*
0.2631	0.0692	0.9521	3.2530	4.0000	175.0000	0.0133

Model

	coeff	se	t	*p*	LLCI	ULCI
constant	0.0000	0.0727	0.0000	1.0000	-0.1435	0.1435
stereotype awareness	0.2374	0.0745	3.1855	0.0017	0.0903	0.3844
age	0.0726	0.0738	0.9835	0.3267	-0.0731	0.2182
gender	0.0064	0.0731	0.0871	0.9307	-0.1379	0.1506
depression	0.0852	0.0738	1.1545	0.2499	-0.0604	0.2307

Outcome variable: self-concurrence
Model summary

R	R^2	MSE	F	df1	df2	*p*
0.6129	0.3756	0.6423	20.9349	5.0000	174.0000	0.0000

Model

	coeff	se	t	*p*	LLCI	ULCI
constant	0.0000	0.0597	0.0000	1.0000	-0.1179	0.1179
stereotype awareness	0.0248	0.0630	0.3947	0.6936	-0.0994	0.1491
stereotype agreement	0.4174	0.0621	6.7222	0.0000	0.2948	0.5399
age	0.0253	0.0608	0.4167	0.6774	-0.0946	0.1453
gender	0.0136	0.0600	0.2262	0.8213	-0.1049	0.1321
depression	0.3923	0.0608	6.4503	0.0000	0.2723	0.5123

Outcome variable: self-esteem
Model summary

R	R^2	MSE	F	df1	df2	*p*
0.6681	0.4463	0.5729	23.2442	6.0000	173.0000	0.0000

Model

	coeff	se	t	*p*	LLCI	ULCI
constant	0.0000	0.0564	0.0000	1.0000	-0.1113	0.1113
stereotype awareness	-0.1216	0.0595	-2.0448	0.0424	-0.2390	-0.0042
stereotype agreement	-0.0048	0.0658	-0.0732	0.9417	-0.1347	0.1251
self-concurrence	-0.2078	0.0716	-2.9026	0.0042	-0.3491	-0.0665
age	0.1621	0.0574	2.8215	0.0053	0.0487	0.2754
gender	0.1072	0.0567	1.8905	0.0604	-0.0047	0.2191
depression	-0.4778	0.0639	-7.4734	0.0000	-0.6040	-0.3516

Total effect
Outcome variable: self-esteem
Model summary

R	R^2	MSE	F	df1	df2	*p*
0.6415	0.4116	0.6019	30.6007	4.0000	175.0000	0.0000

Model

	coeff	se	t	*p*	LLCI	ULCI
constant	0.0000	0.0578	0.0000	1.0000	-0.1141	0.1141
stereotype awareness	-0.1485	0.0592	-2.5068	0.0131	-0.2654	-0.0316
age	0.1501	0.0587	2.5587	0.0114	0.0343	0.2660
gender	0.1038	0.0581	1.7861	0.0758	-0.0109	0.2185
depression	-0.5671	0.0586	-9.6699	0.0000	-0.6829	-0.4514

Indirect effects

	Effect	BootSE	BootLLCI	BootULCI
total	-0.0269	0.0187	-0.0654	0.0081
stereotype awareness → stereotype agreement → self-esteem	-0.0011	0.0157	-0.0306	0.0336
stereotype awareness → self-concurrence → self-esteem	-0.0052	0.0136	-0.0354	0.0196
stereotype awareness → stereotype agreement → self-concurrence → self-esteem	-0.0206	0.0104	-0.0455	-0.0049

Analysis notes:
Level of confidence for all confidence intervals in output: 95.0000
Number of bootstrap samples for percentile bootstrap confidence intervals: 10000
