# Supplementary material for: Applying Corrigan’s progressive model of self-stigma to people with depression
Source: PLoS One. 2019 Oct 29;14(10):e0224418. doi: 10.1371/journal.pone.0224418 (PMC6818799; doi:10.1371/journal.pone.0224418)
Supplement: S5 File — (PDF) [file pone.0224418.s005.pdf]

Results of Total Sample

Tab. 1 Sample characteristics of total sample

sample size	730
age in years (mean, standard deviation)	37.7 (13.1)
gender (female) (%)	77.7
marital status (%)	
single	47.5
in partnership	26.2
others	26.3
level of education (%)	
< 12 years of school education	39.0
≥ 12 years of school education	61.0
current type of mental health care (%)	
outpatient	66.2
inpatient	14.9
not applicable	18.9
current severity of depression (mean, standard deviation)	14.7 (6.0)
stereotype awareness (mean, standard deviation)	51.1 (17.4)
personal agreement (mean, standard deviation)	24.3 (11.8)
self-concurrence (mean, standard deviation)	26.9 (13.9)
self-esteem (mean, standard deviation)	13.4 (7.1)

Tab. 2 Bivariate nonparametric Spearman correlations of model variables of total sample

	1	2	3	4	5	6	7
1 gender	--						
2 age	0.081*	--					
3 depression	-0.031	-0.091*	--				
4 awareness	-0.089*	-0.071	0.166***	--			
5 agreement	0.078*	-0.078*	0.176***	0.186***	--		
6 self-concurrence	0.079*	-0.155***	0.447***	0.154***	0.532***	--	
7 self-esteem	0.087*	0.263***	-0.603***	-0.205***	-0.201***	-0.465***	--

depression=current level of depression; awareness=stereotype awareness; agreement=personal agreement; ***p<0.001; **p<0.01; *p<0.05

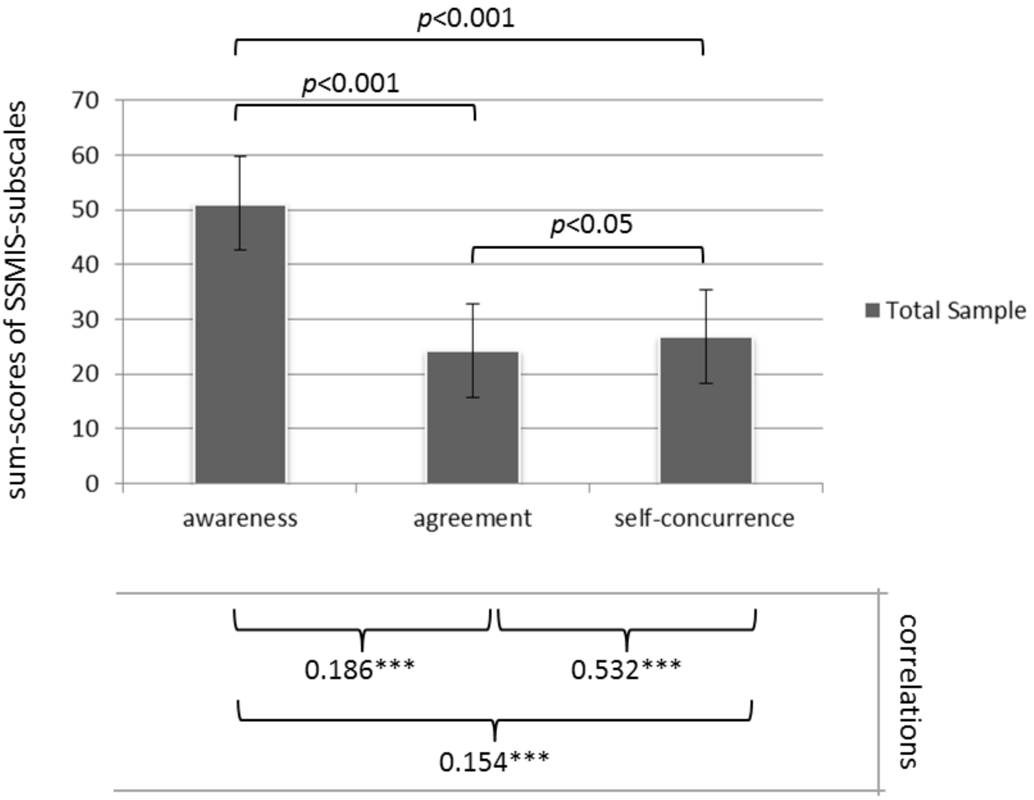

Fig 2. Summed scores and direct associations of stigma attitudes in total sample.

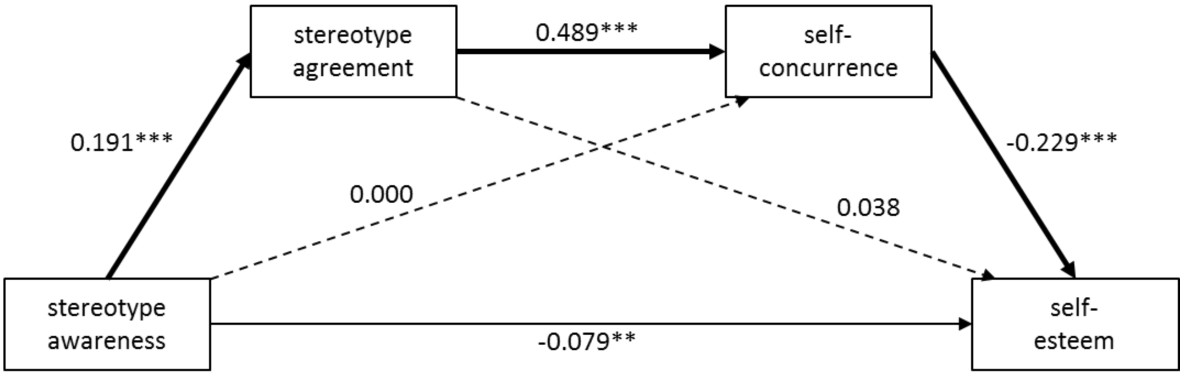

Fig 3. Serial mediation model of total sample. Solid lines indicate significant direct paths. Thick solid lines indicate significant indirect paths. Dotted lines represent non-significant direct paths. Standardized coefficients are presented. ***p<0.001; **p<0.01; *p<0.05.

Results of Serial Mediation Model of Total Sample

PROCESS Procedure for SPSS Version 3.00 (Written by Andrew F. Hayes. Ph.D. www.afhayes.com)
Documentation available in Hayes (2018). www.guilford.com/p/hayes3

Model: 6
Y: self-esteem
X: stereotype awareness
M1: stereotype agreement
M2: self-concurrence
Covariates: age gender depression
Sample Size: 730

Direct effects
Outcome variable: stereotype agreement
Model summary

R	R^2	MSE	F	df1	df2	*p*
0.2728	0.0744	0.9294	14.5786	4.0000	725.0000	0.0000

Model

	coeff	se	t	*p*	LLCI	ULCI
constant	0.0000	0.0357	0.0000	1.0000	-0.0701	0.0701
stereotype awareness	0.1905	0.0364	5.2345	0.0000	0.1190	0.2619
age	-0.0413	0.0361	-1.1446	0.2527	-0.1120	0.0295
gender	0.0918	0.0359	2.5587	0.0107	0.0214	0.1623
depression	0.1408	0.0363	3.8753	0.0001	0.0695	0.2121

Outcome variable: self-concurrence
Model summary

R	R^2	MSE	F	df1	df2	*p*
0.6581	0.4331	0.5700	110.6199	5.0000	724.0000	0.0000

Model

	coeff	se	t	*p*	LLCI	ULCI
constant	0.0000	0.0279	0.0000	1.0000	-0.0549	0.0549
stereotype awareness	0.0002	0.0290	0.0074	0.9941	-0.0568	0.0572
stereotype agreement	0.4887	0.0291	16.8026	0.0000	0.4316	0.5458
age	-0.1001	0.0283	-3.5418	0.0004	-0.1556	-0.0446
gender	0.0579	0.0282	2.0507	0.0407	0.0025	0.1134
depression	0.3311	0.0287	11.5177	0.0000	0.2747	0.3878

Outcome variable: self-esteem
Model summary

R	R^2	MSE	F	df1	df2	*p*
0.6770	0.4583	0.5455	101.9410	6.0000	723.0000	0.0000

Model

	coeff	se	t	*p*	LLCI	ULCI
constant	0.0000	0.0273	0.0000	1.0000	-0.0537	0.0537
stereotype awareness	-0.0792	0.0284	-2.7894	0.0054	-0.1350	-0.0235
stereotype agreement	0.0383	0.0335	1.1429	0.2535	-0.0275	0.1042
self-concurrence	-0.2285	0.0364	-6.2840	0.0000	-0.2998	-0.1571
age	0.1651	0.0279	5.9209	0.0000	0.1130	0.2198
gender	0.0589	0.0277	2.2169	0.0338	0.0045	0.1133
depression	-0.4928	0.0306	-16.1098	0.0000	-0.5529	-0.4328

Total effect
Outcome variable: self-esteem
Model summary

R	R^2	MSE	F	df1	df2	*p*
0.6509	0.4237	0.5787	133.2680	4.0000	725.0000	0.0000

Model

	coeff	se	t	*p*	LLCI	ULCI
constant	0.0000	0.0282	0.0000	1.0000	-0.0553	0.0553
stereotype awareness	-0.0932	0.0287	-3.2470	0.0012	-0.1496	-0.0369
age	0.1910	0.0284	6.7315	0.0000	0.1351	0.2468
gender	0.0390	0.0283	1.3755	0.1694	-0.0166	0.0946
depression	-0.5788	0.0287	-20.1874	0.0000	-0.6351	-0.5225

Indirect effects

	Effect	BootSE	BootLLCI	BootULCI
total	-0.0140	0.0090	-0.0322	0.0032
stereotype awareness → stereotype agreement → self-esteem	0.0073	0.0067	-0.0047	0.0218
stereotype awareness → self-concurrence → self-esteem	0.0000	0.0067	-0.0135	0.0132
stereotype awareness → stereotype agreement → self-concurrence → self-esteem	-0.0213	0.0060	-0.0345	-0.0108

Analysis notes:
Level of confidence for all confidence intervals in output: 95.0000
Number of bootstrap samples for percentile bootstrap confidence intervals: 10000
